# Dicarboxylate and dicarboxylic acid appended supramolecular self-associating amphiphiles as antimicrobial agents against high priority bacterial pathogens

**DOI:** 10.1039/d5ob01615k

**Published:** 2025-11-25

**Authors:** Lisa J. White, Bree Streather, J. Mark Sutton, Jennifer Rankin, Jennifer Baker, Charlotte Bennett, Hollie B. Wilson, Charlotte K. Hind, Jennifer R. Hiscock

**Affiliations:** a University of Kent Canterbury CT2 7NH UK J.R.Hiscock@Kent.ac.uk; b Countermeasures Development and Evaluation Preparedness, UKHSA Salisbury SP4 0JG UK Charlotte.Hind@UKHSA.gov.uk; c Cancer Research Horizons, Babraham Research Campus Cambridge CB22 3AT UK

## Abstract

Antimicrobial resistance remains a significant global health challenge. Supramolecular self-associating amphiphiles (SSAs) represent a promising class of compounds for development as antimicrobial agents, offering tuneable self-assembly properties alongside phospholipid membrane-targeting capabilities. Here we evaluate a series of 10 chiral SSAs, incorporating either urea-dicarboxylic acid or urea-dicarboxylate functional groups for the first time. This series of compounds has been designed to investigate how structural features including chirality, positioning of multiple polar functionalities, hydrophobicity, lipophobicity and hydrogen bond donor/acceptor activity effects not only physicochemical properties, such as self-associative aggregate formation, but also antimicrobial activity against the high threat priority pathogens *Pseudomonas aeruginosa*, *Klebsiella pneumoniae*, *Escherichia coli*, *Acinetobacter baumannii*, *Staphylococcus aureus*, *Enterococcus faecalis* and *Enterococcus faecium*. Finally we perform initial *in vitro* ADME studies to assess the potential for this sub-class of SSAs to be developed as antibiotics.

## Abstract 2

Chiral SSAs show antimicrobial activity, targeting key pathogens, with potential for development as antibiotics confirmed *via in vitro* DMPK studies.

## Graphical abstract

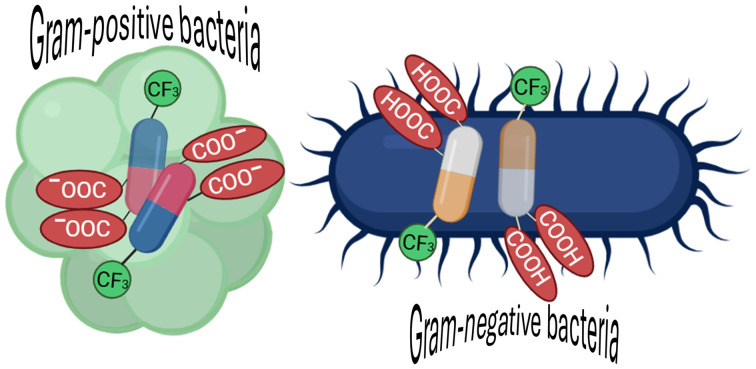

## Introduction

As certified by the United States Centre for Disease Control,^1^ the European Centre for Disease Control^2^ and the World Health Organisation (WHO),^3^ antimicrobial resistance (AMR) remains one of the major global health threats of the 21st century.^4^ With the number of worldwide deaths attributed to the primary effects of AMR infections forecast to rise to ten million per year by 2050. Furthermore, the cumulative number of deaths directly attributable to AMR from 2025 to 2050 is projected to reach approximately 39 million, with an additional ∼169 million deaths indirectly associated with AMR over the same time period.^5^ This estimate is inclusive of deaths attributed to direct infection and selected incidence estimates for different underlying conditions from the Global Burden of Diseases, Injuries, and Risk Factors Study 2021.^6^ In addition, the COVID-19 pandemic has also been shown to increase the impact of AMR,^7^ with a global 15% increase in death rates directly attributed to AMR infections from 2019 to 2020.^1^ This impact on human health is projected to significantly decrease GDP, with losses of up to $3.4 trillion by 2030,^8^ felt most by low-income countries.^9^

To curb the rise of and even reverse the impact of AMR, a combination of complementary approaches will be required. This includes slowing the emergence of resistant bacteria caused by overuse and misuse of antibiotics through effective stewardship,^10–12^ and the development of novel antibiotics alongside agents to enhance the efficacy of currently redundant antibiotics, to which there is large scale resistance, as suggested by the ‘One Health approach’.^13^ To meet the need associated with the development of novel antibiotic or antibiotic enhancement agents, a unified effort has resulted in the development of 27 novel antibiotics that target priority pathogens entering clinical development.^14^ Of these novel antibiotics, six meet at least one of the four WHO innovation criteria, used to evaluate the potential of an innovation to overcome existing mechanisms of AMR.^14^ However, the current rate of development is still not sufficient to meet current and future global need.^15–19^

The field of supramolecular chemistry has and continues to contribute many novel approaches to the development of novel antimicrobial agents/materials, alongside drug delivery vehicles and drug efficacy enhancement agents.^20–23^ Examples include the development of positively charged, self-assembling antibacterial nanoparticles that selectively disrupt negatively charged bacterial membranes;^24,25^ self-assembling hydrogels exhibiting antimicrobial and antiviral activity;^26–29^ short peptide and hybrid peptide–nanoparticle assemblies which are organised through optimisation of the supramolecular interactions present to enhance antimicrobial efficacy,^30–32^ and small-molecule adjuvants that potentiate the efficacy of antibiotics against both Gram-positive^33^ and Gram-negative pathogens.^34,35^ The use of supramolecular chemistry to enhance the therapeutic efficacy of antibacterial agents has also been demonstrated through the development of prodrugs,^36^ in combination therapies,^37,38^ and as drug delivery systems,^39–41^ addressing existing limitations.^42^

In our own work, we have developed a series of supramolecular self-associating amphiphiles (SSAs) that: demonstrate antimicrobial activity against multiple strains of clinically relevant, drug resistant and susceptible *Staphylococcus aureus* (*S. aureus*) and *Escherichia coli* (*E. coli*) bacterial strains;^43^ act as antibiotic efficacy enhancement and activation agents against the ESKAPE pathogen *Pseudomonas aeruginosa* (*P. aeruginosa*);^44^ and demonstrate antibiofilm activity against the bacterial and fungal pathogens of clinical relevance, methicillin-resistant *S. aureus* (MRSA),^43^*P. aeruginosa* and *Candida albicans*.^44,45^

We hypothesise SSAs achieve this biological activity through a combination of selective cell membrane association, permeation and disruption events, which may enhance the movement of molecules/ions across this biological barrier.^44,46^ SSAs are known to self-associate in aqueous conditions, producing spherical aggregates with a hydrodynamic diameter (*d*_H_) of approximately 100–550 nm,^43,47–49^ although the formation of fibres resulting in the production of hydrogel materials have also been observed upon the addition of salt.^50,51^ Studies to date have provided evidence that this process is mediated through the balance, activation and spatial positioning of hydrogen bond donating and accepting functionalities within the SSA anionic component.^43,47–49,52,53^

Upon arrival at the target cell surface, these spherical aggregates morph into a coating which enables the SSAs to interact with the cell surface landscape. Should preferential SSA: cell membrane interactions be achieved, and a critical SSA concentration be obtained, biological activity will be observed.^44,46^ To highlight the potential of this class of compounds to be developed as therapeutic drugs, therapeutic enhancement agents or drug delivery vehicles, a combination of *in vitro* and *in vivo* DMPK (drug metabolism and pharmacokinetic) studies have previously shown examples from this class of compounds to exhibit a drug-like profile.^46,54^

**Fig. 1:**
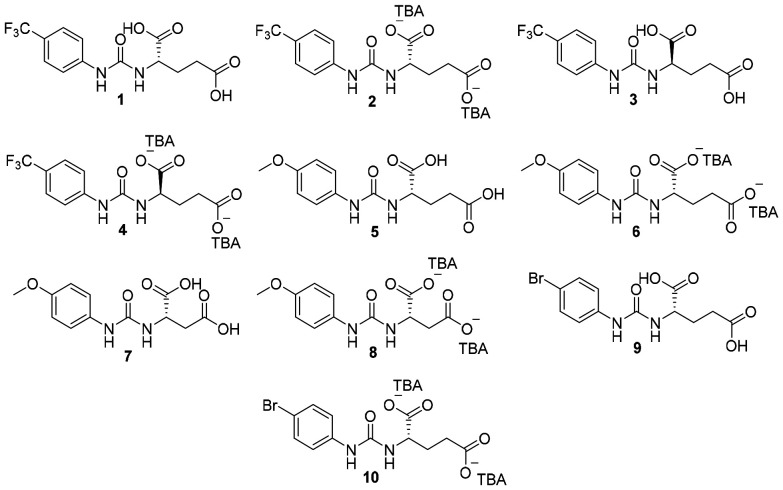
Chemical structure of 10 novel SSAs. TBA = tetrabutylammonium.

Chirality is widely recognized for its critical role in numerous fundamental biological processes,^55,56^ including enhanced *in vivo* stability^57^ and improved biological activity,^58^ boosting therapeutic potential.^59,60^ Herein we introduce chirality into the anionic component of the SSA, through the introduction of 10 novel compounds into our wider molecular library, Fig. 1. We determine the influence of chirality, enhanced hydrophilicity, alongside the introduction of a greater number of competitive hydrogen bond donating and accepting functionalities on SSA self-association properties, antibacterial activity, selectivity, biocompatibility and ADME (Absorption, Distribution, Metabolism and Excretion) properties.

## Results and discussion

SSAs 1, 3, 5, 7 and 9 (Fig. 1) represent a novel sub-class of chiral urea-di-carboxylic acid SSAs, while 2, 4, 6, 8 and 10 represent a novel sub-class of tetrabutylammonium (TBA) chiral urea-di-carboxylate SSAs. Each of these SSA sub-classes contain examples of enantiomers, to enable us to determine whether one isomer exhibits an enhanced therapeutic effect (eutomer) over the other (distomer). Alongside these structural differences, we also investigate the effects of altering the length of alkyl linking groups, and phenyl ring electron withdrawing and donating substituents on molecular self-association events and resultant antimicrobial activity.

### Synthesis

SSAs 1, 3, 5, 7 and 9 were synthesised through the reaction of the appropriate amino acid, supplied as the dimethyl ester hydrochloride salt, with the appropriate isocyanate, followed by deprotection using sodium hydroxide in a combination of isopropyl alcohol and methanol. SSAs 1, 3, 5, 7 and 9 were obtained as white solids in yields of 79%, 73%, 73%, 66% and 89% respectively. SSAs 2, 4, 6, 8, and 10 were obtained by the addition of two equivalents of TBAOH to 1, 3, 5, 7 and 9 in methanol, which afforded the final products as either clear or opaque white oils in a 100% yield.

### SSA self-association properties

**Fig. 2:**
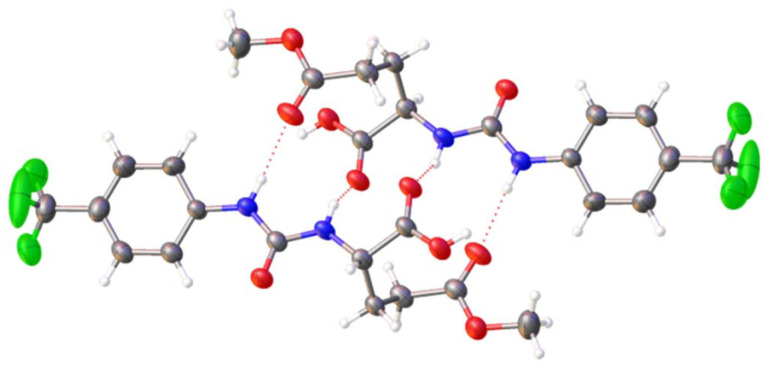
Single crystal X-ray structure obtained for the methyl ester of 3. TBA counter cations have been omitted for clarity. Grey = carbon, blue = nitrogen, red = oxygen, white = hydrogen, green = fluorine, red dashed lines = hydrogen bonds.

The self-associative properties of 1–10 were established in line with previously published methodologies.^47,48,52,61,62^ Single crystals of the methyl ester of 3, monoprotonated SSA 6 and SSA 10 were obtained through the slow evaporation of the methyl ester of 3, SSA 6 and SSA 10 from isopropyl or methanol in water. Analysis of the data obtained from single crystal X-ray diffraction studies confirmed the self-associative hydrogen bonding mode adopted within these samples. The structure obtained for the methyl ester of 3, shows this SSA intermediate to dimerise through the formation of four intermolecular hydrogen bonds, shown in Fig. 2. Here, the two urea NH's can be seen forming hydrogen bonds with two different hydrogen bond accepting functionalities, in this instance the carbonyl oxygen contained within the carboxylic acid and methyl ester functionalities.

**Fig. 3:**
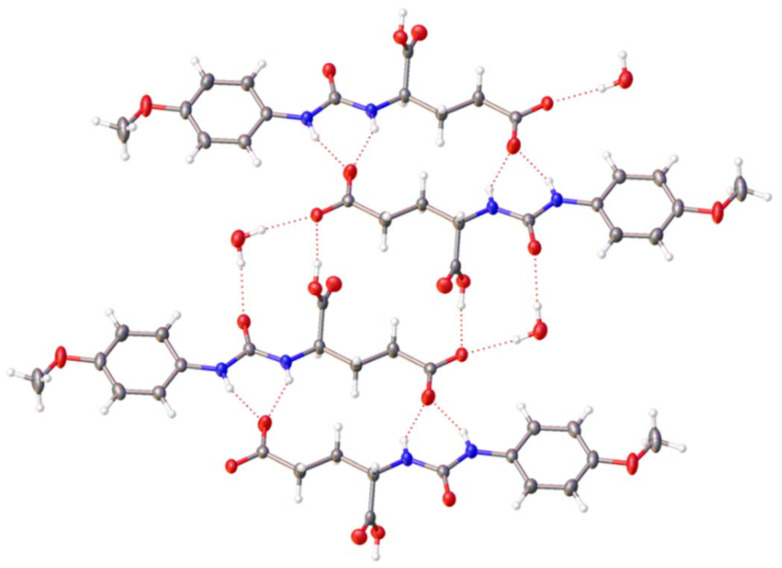
Single crystal X-ray structure obtained for monoprotonated 6. TBA counter cations have been omitted for clarity. Grey = carbon, blue = nitrogen, red = oxygen, white = hydrogen, red dashed lines = hydrogen bonds. Interior angle of dimerization = 180°.

Fig. 3 shows the hydrogen bond mediated, self-associative binding mode adopted by mono-protonated SSA 6 in the solid state, meaning each SSA anion carries a single negative charge. This protonation event was a result of the crystallisation process. Again, we see the formation of a hydrogen bonded dimer, stabilised through the formation of four hydrogen bonds. However, in this instance the two urea NH's form hydrogen bonds with the carboxylate functionality only, which we hypothesize to be due to the enhanced basicity of this functionality over the carboxylic acid functionality and crystal packing forces. The formation of hydrogen bonds between the carboxylate, carboxylic acid, urea oxygen and a bridging water molecule then facilitate the incorporation of these anionic dimers into a hydrogen bonded anionic tape, extending the hydrogen bond mediated, SSA self-association events within this single crystal.

**Fig. 4:**
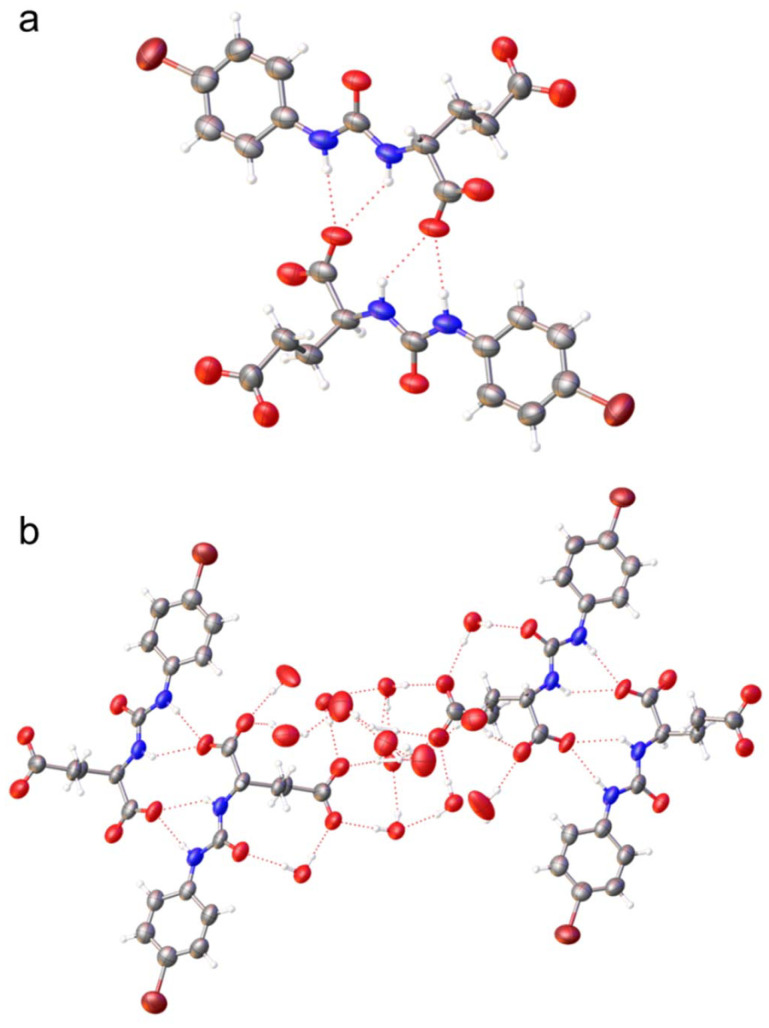
Single crystal X-ray structure obtained for 10. Grey = carbon, blue = nitrogen, red = oxygen, white = hydrogen, purple = bromine, red dashed lines = hydrogen bonds. Interior angle of dimerization = 180°. (a) TBA counter cations and water molecules have been omitted for clarity; (b) TBA counter cations have been omitted for clarity.

Fig. 4 shows an example of a single crystal X-ray structure obtained for an SSA, where the anionic portion bares two negatively charged carboxylate groups. As with 6 (Fig. 3), SSA 10 (Fig. 4a) contains the same arrangement of alkyl linking groups between the carboxylate (or carboxylic acid) groups and urea functionality. Although both SSA anions form a hydrogen bonded dimer, stabilised through the formation of four hydrogen bonds between the urea NH and carboxylate groups, the functionality which acts as the hydrogen bond acceptor in the formation of these complex changes due to the presence of the additional negative charge maintained within the structure of 10. Within this example the presence of a second carboxylate functionality acts as a hydrogen bond acceptor, facilitating interactions with water molecules incorporated within this structure (Fig. 4b). This provides evidence that the hydrogen bonding mode, involved in SSA anion self-association events may be controlled through processes as simple as selective protonation/deprotonation events.

Moving into the solution state, the self-association properties of 1–10 were characterised in an aqueous solution standardised with 5% EtOH, in line with our previous experimental procedures and to aid compound solubility across the full SSA library.^47,48^ To confirm the presence of higher order self-associative structures quantitative (*Q*) ^1^H NMR spectroscopy studies were undertaken at 5.6 mM in D_2_O 5% EtOH. Here the ^1^H NMR resonances attributed to the EtOH are used as an internal standard. Through comparative integration with the SSA signals, this enables the proportion of an SSA to be incorporated into larger aggregated species, which exhibit solid-like properties, and thus become NMR spectroscopy silent, to be determined.

As shown in Table 1, the percentage of 1–10 to be incorporated into higher-order aggregated species under these experimental conditions ranges from 34% to 71%. Where both an anionic and cationic component are present, they are found to exist in a 1 : 1 charge ratio within any higher order aggregated species. As expected, due to the achiral environment in which the behaviour of 1–10 is observed, the proportion of the different enantiomers to become incorporated into higher order structures under these environmental conditions remains within the limits of reproducibility for these SSA systems. Interestingly those SSAs which exist as the TBA salt (2, 4, 6, 8 and 10) all exhibit an approximate 40% SSA incorporation into those higher order structures which exhibit solid-like properties, regardless of any changes to the molecular structure. However, this is a trend that is not retained when those SSAs that contain the carboxylic acid residues – SSAs 1, 3, 5, 7 and 9. Here the exchange of the electron withdrawing CF_3_ (1 and 3) and Br (9) phenyl ring substituents with the OMe residue (5 and 7, see c log *P* values, Table 1) increasing the proportion of the SSA to be incorporated into the higher-order self-associated structures, this is further enhanced through the shortening of the alkyl linking groups between the carboxylic acid residues (5 = 56%; 7 = 71%).

**Table 1:** Overview of the results obtained from (*Q*) ^1^H NMR spectroscopy, CAC (tensiometry) determination studies, and calculated consensus (c) log *P* values for 1–10 in either a D_2_O 5.0% EtOH or H_2_O 5.0% EtOH solution respectively. (*Q*) ^1^H NMR values given in % represent the observed proportion of compound to become ^1^H NMR spectroscopy silent under these experimental conditions. All (*Q*) ^1^H NMR spectroscopy experiments were conducted at a concentration of 5.6 mM with a delay time (*d*1) of 60 s at 298 K. All samples underwent an annealing process where they were heated to approximately 313 K before being left to cool to the experimental temperature (*Q*) ^1^H NMR spectra = 298 K; tensiometry = 298 K

SSA	(*Q*) ^1^H NMR spectroscopy (%)		CAC (mM)	ST at CAC (mN m^−1^)	c log *P*
	Acid or anion	Cation			
1	39	a	19	39	1.70
2	40	36	20	37	1.17
3	34	a	20	37	1.73
4	41	43	22	38	1.18
5	56	a	b	58	0.65
6	42	42	c	38^d^	0.11
7	71	a	b	55	0.28
8	47	42	b	52	−0.21
9	41	a	c	54^d^	1.29
10	38	44	26	41	0.77

a No counter cation present.

b CAC above the limit of solubility.

c CAC is >100 mM.

d ST at the limit of solubility or 100 mM, whichever concentration is lower.

As the (*Q*) ^1^H NMR spectroscopy experiments conducted at 5.6 mM in a D_2_O 5.0% EtOH solution confirmed the presence of higher-order self-associated structures with solid-like properties, concentration dependent surface tension measurements, obtained through tensiometry, were used to calculate critical aggregate concentration (CAC). Here the CAC is defined as the point at which an increase in SSA concentration no longer decreases surface tension but instead results in the formation of higher-order self-associated structures. However, should a CAC remain undetermined, this does not mean that these larger self-associated aggregates do not exist within the solvent bulk, only that the formation of these higher-order aggregates is governed by dynamic equilibria between interfacial and bulk-phase aggregation processes.^63^

Interestingly, and unlike previous SSA examples,^43^ there is little difference in the surfactant properties or CAC values obtained for carboxylic acid and corresponding carboxylate based SSAs, except for 5 and 6, or 9 and 10. We hypothesise that this is due to the presence of a second polar carboxylate or carboxylic acid functionality, overriding the effects of SSA protonation state on global physicochemical properties in these instances. As expected, the surface tension (ST) properties and CAC of enantiomers 1, 3 and 2, 4 are comparable to one another. Bromine substituted 10 displays slightly higher CAC and ST at CAC values than those obtained for the CF_3_ substituted analogues 2 and 4, supporting the findings from (*Q*) ^1^H NMR spectroscopy experiments, that these two functionalities are almost interchangeable for carboxylate based SSAs (Table 1). Solubility prevented CAC determination for 6 and 9, while the presence of electron-donating MeO groups (5, 7, and 8) were found to comparatively increase aqueous solubility, while simultaneously decreasing surfactant properties. We hypothesise that this is because of the decreased c log *P* values associated with this substituent (Table 1), combined with a deactivation of the NH hydrogen bond donating groups, causing a destabilisation of any hydrogen bond mediated self-association events present.

Dynamic light scattering (DLS) and zeta potential measurements in H_2_O 5.0% EtOH were used to determine the hydrodynamic diameter (*d*_H_) and stability of the higher-order SSA aggregates detected by (*Q*) ^1^H NMR spectroscopy at 5.6 mM, Table 1. As detailed in Table 2, SSAs 2–10 exhibit a *d*_H_ < 350 nm (determined from intensity distribution peak maxima), a similar size to structures obtained at this same concentration for other members of the SSA library.^43,47^ However, we are unable to confidently report a *d*_H_ for 1 due to the instability of this system, which is supported by a zeta potential value of −26 mV, obtained for this SSA under the same experimental conditions. As expected, higher-order aggregates produced under these experimental conditions by carboxylate based SSAs 2, 4, 6, 8 and 10 were all found to demonstrate a greater stability than the corresponding carboxylic acid based SSAs 1, 3, 5, 7 and 9. We believe this to be due, at least in part, to the enhanced strength of SSA anion hydrogen bonded self-association events created by the presence of carboxylate groups which demonstrate enhanced hydrogen bond acceptor properties when compared to carboxylic acid residues. Interestingly, the polydispersity index (PI) values obtained from the DLS data for 2–10 shows a narrow size distribution, <0.5 in all cases, suggesting long term stability of these SSA aggregated systems.^64^ Due to the instability of these systems in the absence of solvent, attempts to further characterise these systems through SEM or AFM are not possible.^48^

**Table 2:** Overview of average DLS intensity particle size distribution peak maxima (aggregate *d*_H_ measurements), polydispersity index (PI) determined from DLS data (error <±1% for all data reported) and zeta potential values obtained for a H_2_O 5.0% EtOH solution of 1–10 (5.6 mM) at 298 K. Error = standard error of the mean. All samples underwent an annealing process where they were heated to approximately 313 K before being left to cool to 298 K

SSA	*d*_H_ (nm)	±Error (nm)	PI	Zeta potential (mV)	±Error (mV)
1	a	a	a	−26	*0.8*
2	140	*1*.*5*	0.33	−52	*0.9*
3	154	*6.6*	0.06	−25	*0.9*
4	255	*39.7*	0.07	−41	*0.6*
5	153	*5.9*	0.05	−28	*0.3*
6	164	*3.1*	0.05	−51	*0.3*
7	214	*3.4*	0.04	−13	*0.4*
8	313	*21.0*	0.07	−29	*0.7*
9	178	*4.5*	0.07	−18	*0.3*
10	307	*6.8*	0.07	−52	*0.7*

a Data quality prevents accurate reporting of *d*_H_ or PI values.

### SSA antimicrobial activity

With the self-associative properties of 1–10 established, the antimicrobial activity of these SSAs were determined against a panel of clinically relevant, high-priority pathogens including: *P. aeruginosa* PAO1; *Klebsiella pneumonia* (*K. pneumoniae*) M6; *E. coli* NCTC 12923; *Acinetobacter baumannii (A. baumannii*) ATCC 17978; *S. aureus* ATCC 9144; *Enterococcus faecalis* (*E. faecalis*) NCTC 775; and *Enterococcus faecium* (*E. faecium*) NCTC 12204. These pathogens were selected based on their role in hospital-acquired infections, (multi)drug resistance profiles, and inclusion in the WHO priority pathogen list (2024).^65^Table 3 shows the % growth inhibition for these bacteria in the presence of 1–10, after 20 hours when incubated with the appropriate SSA in a 2.5% EtOH solution. However, it should be noted that the SSA stock solution was prepared in a H_2_O 5% EtOH solution, to ensure that the SSA aggregates formed were in line with those characterised within the scope of the SSA self-association studies.

**Table 3:** Percentage inhibition of bacterial growth after 20 hours incubation with the appropriate SSA (5.0 mM) in a 2.5% ethanol solution. Negative values represent enhanced % bacterial growth

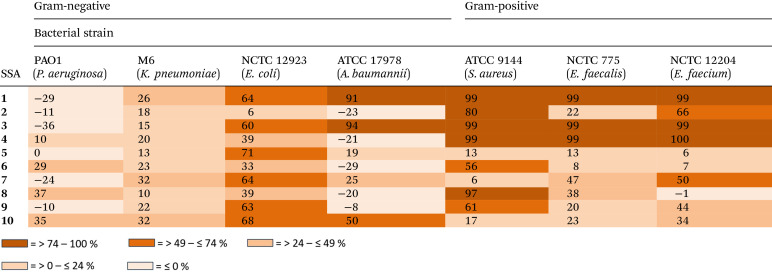

**Fig. 5:**
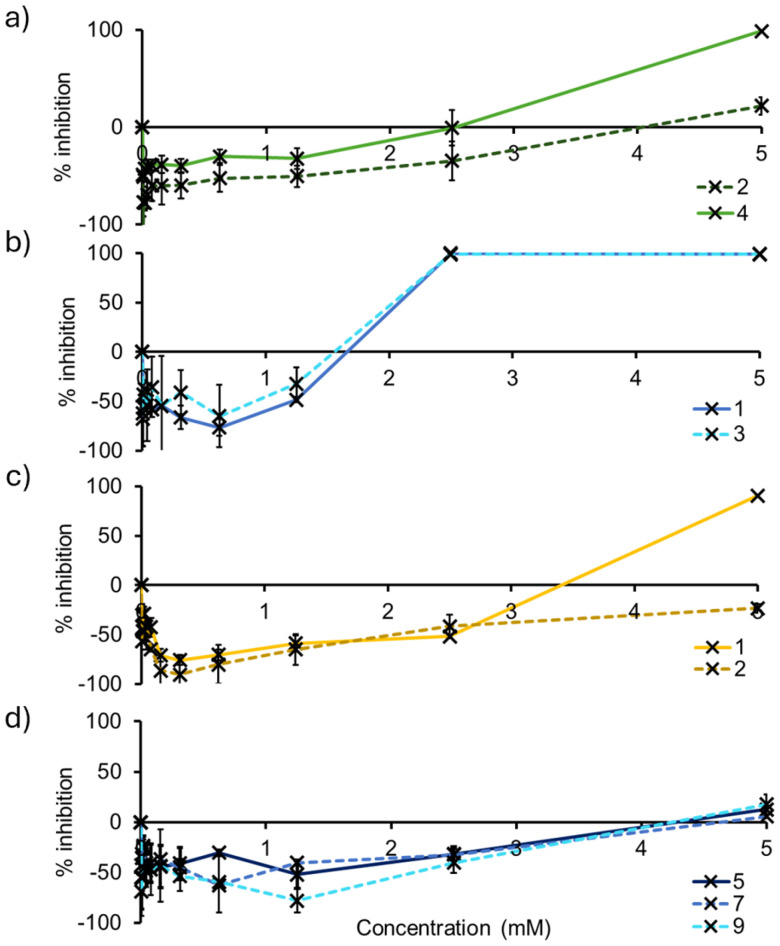
Percentage inhibition of bacterial growth after 20 hours incubation with the appropriate SSA supplied in a H_2_O 5.0% EtOH solution: (a) 2 (dashed dark green line) and 4 (solid light green line) against *E. faecalis* 775; (b) 1 (solid dark blue line) and 3 (dashed light blue line) against *S. aureus* ATCC 9144; (c) 1 (solid light yellow line) and 2 (dashed dark yellow line) against *A. baumannii ATCC 17978*; (d) 5 (solid dark blue line), 7 (dashed dark blue line) and 9 (dashed light blue line) against *S. aureus* ATCC 9144.

Chirality was found to influence antimicrobial activity for the carboxylate based SSAs only. Here SSA 4 (99%–100% inhibition) was found to demonstrate an enhanced antimicrobial activity over SSA 2 (22%–80% inhibition) against all Gram-positive bacteria tested (Table 3 and Fig. 5a), alongside Gram-negative *P. aeruginosa* (10% inhibition *vs.* −10% inhibition) and *E. coli* (39% inhibition *vs.* 6% inhibition). However, although the carboxylic acid analogues 1 and 3 demonstrate a loss in enantiomer dependent antimicrobial activity (Table 3 and Fig. 5b), these SSAs also demonstrate an enhanced broad-spectrum activity when compared to 2 and 4, most notably showing an enhanced antimicrobial activity against the Gram-negative pathogen *A. baumannii* (Fig. 5c). These differences in antimicrobial activity between 1–4 support the hypothesis that the carboxylic acid and carboxylate SSAs demonstrate differences in their mode of antimicrobial activity, that could include differences in membrane disruption processes, cellular delivery or biological target interaction.

When substituting the CF_3_ functionality within SSA 1 for the OMe (5 and 7) or Br (9) group, there is a marked decrease in antimicrobial activity observed against most bacterial strains tested, except for *E. coli* (Table 3). As shown in Fig. 5b and d, this loss in activity is clearly demonstrated against *S. aureus*, where the inhibition of bacterial growth is recorded at 99% for 1, 13% for 5, 6% for 7 and 61% for 9, after 20 hours of incubation with the SSA at 5 mM. This loss in antimicrobial activity we hypothesise to be due to the combined decrease in hydrophobicity, lipophilicity and electron withdrawing properties, resulting in the deactivation of the NH hydrogen bond donating groups – as we move from CF_3_ > Br > OMe. This hypothesis is further supported by the self-associative data obtained for these SSAs and summarised within Tables 1 and 2.

Interestingly when considering SSAs 3–10, the carboxylate SSAs demonstrate enhanced antimicrobial activity against *P. aeruginosa*, over the corresponding carboxylic acid compounds. This trend is reversed when considering the activity of SSAs 1–8 against *E. coli*. However, when considering the antimicrobial activity of SSAs 1–10 against *A. baumannii*, activity is largely confined to CF_3_ substituted carboxylic acid based SSAs 1 (91% inhibition) and 3 (94% inhibition), while a similar level of antimicrobial activity is observed for all SSAs against *K. pneumoniae* – ranging between 10–32% inhibition of bacterial growth. These data highlight the potential for this class of compounds to be developed for enhanced antimicrobial activity and tailored to either demonstrate board spectrum or targeted antimicrobial activity against high threat bacterial pathogens.

### *In vitro* absorption, distribution, metabolism and excretion (ADME) studies

To understand the potential for 1–10 to be developed as antimicrobial agents, a range of *in vitro* ADME studies were performed with a sub-set of structurally diverse SSAs including 1, 2, 3 and 9. This SSA sub-set were prioritised to include those molecules which demonstrated the greatest antimicrobial activity against the broad range of bacteria tested.

Metabolic stability was assessed in mouse and rat liver microsomes. Microsomes are subcellular fractions of hepatocytes and contain CYP450s, the predominant enzymes involved in first-pass metabolism.^66^ All SSAs tested demonstrated a low intrinsic clearance (<7.5 µL min^−1^ mg^−1^) in both mouse and rat liver microsomes. This suggests resistance to metabolism by cytochrome P450 enzymes. A plasma protein binding assay was performed to assess the extent of compound binding to plasma proteins. This is key as only unbound compound is free to distribute and exert a pharmacological effect. When incubated with human plasma over 6 hours these SSAs showed low to moderate binding, correlating to a moderate to high fraction unbound (Fu). To assess the potential for this sub-class of SSAs to be developed as drugs for oral administration, Caco-2 permeability assays were also performed, as a cellular model indicative of human intestinal permeability. The results of which showed the compounds to have low permeability suggesting poor oral absorption and therefore further optimisation would be required for oral administration of these SSAs.

## Conclusions

We have synthesised a novel sub-class of 10 SSAs, that are unique in their structure due to the presence of the two carboxylic acid or carboxylate groups present on the same side of the SSA urea functionality, which also introduces chirality into these systems. Within the solid state we have shown that the introduction of this second carboxylic acid or carboxylate group, combined with the protonation state of these amphiphiles can be used to control self-associative hydrogen bonded modes.

Within a H_2_O 5.0% EtOH solution, the presence of higher order self-associated aggregates is confirmed for 1–10 at 5.6 mM by (*Q*) ^1^H NMR spectroscopy studies, interestingly when comparing the (*Q*) ^1^H NMR spectroscopy and tensiometry data obtained for 1–4, the carboxylate and carboxylic acid based SSAs demonstrate similar self-associative properties. However, when considering the zeta potential data for the higher-order self-associative structures formed at 5.6 mM under these experimental conditions, the carboxylate based SSAs produce structures that demonstrate an enhanced stability when compared to the carboxylic acid based SSAs.

Antimicrobial testing of 1–10 against a panel of high threat Gram-positive and Gram-negative pathogenic bacteria confirmed CF_3_ substituted SSAs to demonstrate the broadest activity. However, alterations in the SSA structure, such as chirality, presence of TBA, carboxylate or carboxylic acid and modulation of hydrophobic, lipophilic and hydrogen bond donor activity was found to substantially impact the specificity and efficacy of this class of compounds. This enables us to confirm that through an understanding of SSA structure activity relationships, antimicrobial efficacy and specificity/broad rage activity may be obtained through effective molecular design. Finally, the *in vitro* ADME results, and antimicrobial activity indicate the potential of this sub-class of SSAs, though additional *in vitro* and *in vivo* studies are required to confirm their suitability as antibiotics.

## Author contributions

L. J. W.: investigation, validation and writing – original draft, review and editing. J. R., J. B., C. B. and H. B. W.: investigation and validation. B. S.: investigation and writing – review and editing. J. M. S. and C. K. H.: supervision, validation, writing – review & editing and funding acquisition. J. R. H.: conceptualization, funding acquisition, project administration, supervision and writing – original draft, review & editing.

## Conflicts of interest

There are no conflicts to declare.

## Supplementary Material

OB-023-D5OB01615K-s001

OB-023-D5OB01615K-s002

## Data Availability

The data supporting this article have been included as part of the supplementary information (SI).††A suitable crystal was selected and mounted on a Rigaku Oxford Diffraction Supernova diffractometer. Data were collected using Cu Kα radiation at 100 K. Structures were solved with the ShelXT^67^ or ShelXS structure solution programs *via* Direct Methods and refined with ShelXL^68^ on Least Squares minimisation. Olex2^69^ was used as an interface to all ShelX programs. CCDC deposition number for the single crystal X-ray structure shown in Fig. 2 = 2388585, Fig. 3 = 2388584 and Fig. 4 = 2388587. Supplementary information: experimental details and computational modelling, DLS, zeta potential, tensiometry, mass spectrometry, NMR spectroscopy, crystallography, molecular characterisation, and biological data. See DOI: https://doi.org/10.1039/d5ob01615k. A suitable crystal was selected and mounted on a Rigaku Oxford Diffraction Supernova diffractometer. Data were collected using Cu Kα radiation at 100 K. Structures were solved with the ShelXT^67^ or ShelXS structure solution programs *via* Direct Methods and refined with ShelXL^68^ on Least Squares minimisation. Olex2^69^ was used as an interface to all ShelX programs. CCDC deposition number for the single crystal X-ray structure shown in Fig. 2 = 2388585, Fig. 3 = 2388584 and Fig. 4 = 2388587. CCDC 2388585, 2388586, 2388584 and 2388587 contain the supplementary crystallographic data for this paper.^70*a*–*d*^
