# Supplementary material for: Dicarboxylate and dicarboxylic acid appended supramolecular self-associating amphiphiles as antimicrobial agents against high priority bacterial pathogens
Source: Org Biomol Chem. 2025 Nov 25;23(48):10913–21. doi: 10.1039/d5ob01615k (PMC12645191; doi:10.1039/d5ob01615k)
Supplement: OB-023-D5OB01615K-s001 [file OB-023-D5OB01615K-s001.pdf]

Supplementary Information (SI) for Organic & Biomolecular Chemistry.
This journal is © The Royal Society of Chemistry 2025

Dicarboxylate and dicarboxylic acid appended supramolecular self-associating amphiphiles as antimicrobial agents against high priority bacterial pathogens

Lisa J. White,[a] Bree Streather, [a] J. Mark Sutton,[b] Jennifer Rankin,[c] Jennifer Baker,[c] Charlotte Bennett,[c] Hollie B. Wilson,[c] Charlotte K. Hind *[b] and Jennifer R. Hiscock*[a]

Contents

Section 1: General remarks 2
Section 2: Physicochemical methods 2
Section 3: Biological methods 4
Microbial Methods 4
In vitro Absorption, Distribution and Metabolism 4
Section 4: Chemical structures 6
Section 5: Chemical synthesis 6
Section 6: Characterisation NMR spectra 10
Section 7: Mass Spectrum data 22
Section 8: Quantitative ^{1}H NMR studies 26
Summary 36
Section 9: ^{1}H NMR DOSY studies 37
Summary 42
Section 10: Dynamic Light Scattering (DLS) studies 43
Summary 52
Section 11: Zeta potential studies 53
Zeta potential studies in H_2O/5.0 % EtOH 53
Summary 58
Section 12: Surface tension measurements and critical aggregate concentration (CAC) determination 58
Summary 63
Section 13: Single crystal X-ray structures 64
Section 14: Molecular modelling and simulation 70
Calculation of LogP values using Swiss ADME 75
Section 15: *In vitro* Drug Metabolism and Pharmokinetics (DMPK) studies 76
Protein Binding Measurements in Human Plasma by Using Equilibrium Dialysis 76
Kinetic Solubility Determination in PBS pH 7.4 76

Metabolic Stability in Rat and Mouse Liver Microsomes 76
Bidirectional Permeability in Caco-2 Cell Line 78
Section 16: Antimicrobial activity 78
Summary 149
Section 17: References 149

Section 1: General remarks

A positive pressure of nitrogen and oven dried glassware were used for all reactions. All solvents and starting materials were purchased from known chemical suppliers or available stores and used without any further purification unless specifically stipulated. The NMR spectra were obtained using a Bruker AV2 400 MHz or AVNEO 400 MHz spectrometer. The data was processed using TopSpin 4.1.4. software. NMR chemical shift values are reported in parts per million (ppm) and calibrated to the centre of the residual solvent peak set (s = singlet, br = broad, d = doublet, t = triplet, q = quartet, m = multiplet). Tensiometry measurements were undertaken using the Biolin Scientific Theta Attension optical tensiometer. The data was processed using Biolin OneAttension software. A Hamilton (309) syringe was used for these measurements. The melting point for each SSA was measured using Stuart SMP10 melting point apparatus. High resolution mass spectrometry was performed using a Waters Acquity H-Class (Quaternary Solvent Manager(QSM) and Flow Through Needle(FTN)), configured to allow direct injection into a Waters Vion. Infrared spectra were obtained using Shimadzu IR-Affinity-1 model Infrared spectrometer. The data was analysed in wavenumbers (cm^{-1}) using IRsolution software. DLS and Zeta Potential studies were carried out using an Anton Paar Litesizer™ 500 and processed using Kalliope™ professional software.

Section 2: Physicochemical methods

^{1}H NMR Diffusion Ordered Spectroscopy (DOSY): The hydrodynamic diameter was derived from diffusion rates obtained from ^{1}H NMR DOSY measurements using the Stokes-Einstein equation. The viscosity value used for the calculation was 0.00199 mPa (DMSO).

Quantitative (Q) ^{1}H NMR studies: A ^{1}H NMR spectrum was obtained with a delay (d_1 = 60 s) for SSAs (112 mM) in DMSO-d_6/ 1.0 % DCM or 5.56 mM in D_2O/ 5.0 % EtOH. Through comparative integration of the anionic and cationic component signals against the internal standard signals (DCM/EtOH), the proportion of these molecular components to become 'lost' from solution, through the adoption of solid-like characteristics can be calculated.

Tensiometry studies: All the samples were prepared in an H_2O/ 5.0 % EtOH solution. All samples underwent an annealing process where they were heated to approximately 313 K before being left to cool to RT, enabling each sample to reach a thermodynamic minimum. All samples were prepared through serial dilution of the most concentrated sample. Three surface tension measurements were obtained for each sample at a given concentration using

the pendant drop method. The average values were then used to calculate the critical aggregate concentration (CAC).

Mass spectrometry: Ammonium Acetate (10 mM, Supleco Lichropur, Lot AM 1890034413) was prepared in H_2O/ 5.0 % EtOH solution (Fischer Chemicals, Lot 2361141): Methanol (Sigma-Aldrich, Lot STBK7695). The QSM was configured to deliver 0.02 mL/min using direct injection. The FTN delivered 10 uL per injection and samples were analysed in triplicate. The parameters for the mass spectrometry are as follows: Source Temperature = 121 °C; Desolvation Temperature = 350 °C; Cone Gas = 49 L/h; Desolvation Gas = 597 L/h; Capillary = 2.54 kV; Mode = negative mode, High Definition MSE; Scan Time = 1.000 secs.

Mass Spectrometry Standards: Lock mass spray consisted of a Leucine Enkephalin (ex. Waters, Lot W27072310) (100 pg/µL) and was prepared according to Waters procedure but adjusted to 100 pg/µL; Mass spec calibration performed using Major Mix (ex. Waters, Lot W07022424) and prepared according to Waters procedure 715005131. Rev A. Sample Preparation: Approximately 1 mg of each SSA was dissolved in 1 mL of methanol (Sigma-Aldrich, Lot STBK7695). This solution was further diluted 10 µL in 1000 µL H_2O/ 5.0 % EtOH solution (Fischer 8 Chemicals, Lot 2361141): methanol (Sigma-Aldrich, Lot STBK7695). Samples directly injected into a flow of 10 mM ammonium acetate in H_2O/ 5.0 % methanol at 0.2 mL/min.

Dynamic light scattering (DLS) studies: All solvents used were filtered to remove any particulates that may interfere with the results obtained. All samples underwent an annealing process, in which they were heated to 313 K before being left to cool to 298 K to allow each sample to reach a thermodynamic minimum. A series of 10 runs were recorded at 298 K.

Zeta potential studies: All solvents used were filtered to remove any particulates that may interfere with the results obtained. All samples underwent an annealing process in which they were heated to 313 K before being left to cool to 298 K to allow each sample to reach a thermodynamic minimum. The final zeta potential value given is an average of the number of experiments conducted at 298 K.

Crystal X-ray studies: Single crystals were produced through selective precipitation using ethanol and isopropanol. A suitable crystal was selected and mounted on a Rigaku Oxford Diffraction Supernova diffractometer. Data were collected using Cu Kα radiation at 100 K. Structures were solved with the ShelXT[1] or ShelXS[2] structure solution programs via Direct Methods and refined with ShelXL[3] by least Squares minimisation. Olex2[4] was used as an interface to all ShelX programs (CCDC 2388585, 2388586, 2388584 and 2388587).

Molecular modelling and simulation: Structures of the SSAs were drawn in ChemDraw (version 22.0.0) and were imported into Chem3D (version 22.2.0). MM2 energy minimisations were run, giving the dipole-dipole energy and total energy in kcal/mol. The length of the SSA was obtained by measuring the distance between the two atoms furthest away from each other, given in Å and converted to nm.

Section 3: Biological methods

Microbial Methods

These methods were taken directly from UK Health and Security Office (UKHSA) standard operating procedures.

Preparation of Luria Broth media (LB): Yeast extract (5.0 g), tryptone (10.0 g) and sodium chloride (10.0 g) were dissolved in dH_2O (1.0 L) then divided into bottles and autoclaved.

Preparation of Luria Broth (LB) agar plates: Agar (6.0 g) was added to LB (400 mL) and autoclaved. Once cool, the LB agar was poured into sterile petri dishes under sterile conditions and allowed to set. LB plates were stored at 4 °C until use.

Preparation of bacterial plates: Sterile LB agar plates were streaked using streaked using the desired bacteria i) *Pseudomonas aeruginosa* (*P.aeruginosa*) PAO1, ii) *Klebsiella pneumoniae* (*K. pneumoniae*) M6 iii) *E. coli* NCTC 12923 and iv) *Acinetobacter baumannii* (*A. baumannii*) ATCC 17978 and Gram-positive i) *Staphylococcus aureus* (*S. aureus*) ATCC 9144 ii) *Enterococcus faecalis* (*E. faecalis*) NCTC 775 and iii) *Enterococcus faecium* (*E. faecium*) NCTC 12204, then incubated at 37 °C overnight.

Preparation of Inoculum: An initial culture was made up by inoculating LB media (5 mL) with a single colony of bacteria under sterile conditions and incubated at 37 °C with shaking overnight. The following day, overnight bacterial cultures were subcultured into fresh LB medium to a starting OD_{600} of 0.01.

Preparation of 96 well microplate for MIC_{50}: The 1:100 cell suspension (150 µL) was dispensed into individual wells under sterile conditions. SSAs (30 µL) were added to the wells to equal a total volume of 180 µL in the wells. Six repeats of each concentration for each SSA were created each plate. The plates were sealed using parafilm, then incubated at 37 °C in a microplate reader for 20 hours. OD_{600} endpoint data was used to generate growth curves.

In vitro Absorption, Distribution and Metabolism

These studies were performed by Contract Research Organisation (CRO) Pharmaron. The methodology provided within the ESI is published within the express permission of this organisation. The percentage of a compound recovered during the analysis process should be 100 %; any deviation from this value indicates experimental limitations such as unintended potential non-specific binding events to experimental equipment, solubility issues, etc.

Metabolic Stability in Rat and Mouse Liver Microsomes:

The metabolic stability of compounds at a concentration of 1 µM, in rat and mouse liver microsomes was measured following incubation with hepatic microsomes (0.5 mg of protein / mL) in the presence and absence of cofactor (NADPH) at 37 °C. Aliquots of each incubation were removed at 0.5, 5, 15, 30 and 60 minutes. The reaction was stopped by the addition of 5 volumes of cold acetonitrile containing analytical internal standard (IS).

Samples were then centrifuged at 3 °C, 220 g for 40 minutes to remove cell debris. The supernatant was aliquoted and added to ultra-pure H_2O in a 1:1 ratio in preparation for LC-MS/MS analysis.

Samples were analysed using a Shimadzu liquid chromatography system coupled to a Triple Quad 6500+ mass spectrometry (AB Inc.) Separation of compounds was achieved using an HSS T3 column (50 x 2.1 mm) at a temperature of 40 °C and a binary mobile phase gradient at a flow rate of 0.7 mL/min. Initial LC conditions comprised of 95 % solvent A (0.1 % formic acid in water) and 5 % solvent B (0.1 % formic acid in acetonitrile). This was ramped to 95 % solvent B at 0.7 minutes, held for 0.5 minutes and then immediately returned to initial conditions. Sample analysis was by electrospray ionisation.

Protein Binding Measurements in Human Plasma by Using Equilibrium Dialysis Method:

The fraction unbound in human plasma (P-KB, Ltd People's hospital of Shandong) were measured using 96-well Equilibrium Dialysis Plate (HTDialysis LLC, Gales Ferry, CT). Human plasma containing 5 µM test compound were dialysed against dialysis buffer (PBS) with initial samples taken to allow for assessment of plasma stability and recovery before the plate was incubated for 6 hours at 37 °C at 5 % CO_2. Samples were removed at 6 hours, and all samples were matrix matched and quenched with four volumes of acetonitrile containing an appropriate analytical internal standard. Samples were centrifuged at 3,220 g for 30 minutes (Eppendorf 5810R) to precipitate protein and enable sampling of supernatant prior to appropriate dilution for liquid chromatography mass-spectrometry (LC-MS/MS) analysis. Samples were analysed using a Shimadzu coupled to a triple Quad 6500+ (AB Inc., Illinois). Separation of analytes was achieved using a Xselect Hss T3 (2.1 x 50 mm) column at a temperature of 40 °C and a binary mobile phase gradient at a flow rate of 0.8 mL/min. Initial LC conditions comprised of 95 % solvent A (0.1 % formic acid in water), 5 % solvent B (0.1 % formic acid in acetonitrile); this was ramped to 98 % B at 0.5 min, held for 0.4 min and then immediately returned to initial conditions. Sample analysis was by electrospray ionisation in positive ion mode.

Bi-directional Permeability in Caco-2 Cell Line:

The bi-directional permeability in Caco-2 cell lines was determined by seeding cells onto an HTS Transwell plate. Cells were cultivated for 14-18 days in a cell culture incubator at 37 °C, 5 % CO_2, 95 % relative humidity. Cell culture medium was replaced every other day. To assess monolayer integrity the transepithelial electrical resistance (TEER) across the monolayer was measured using Millicell Epithelial volt-ohm meter prior to assay and Lucifer yellow (LY) fluorescence measurement post-assay.

Test and control compounds were prepared in DMSO and diluted in Hank's Balanced Saline Solution (HBSS, pH 7.4) to a final concentration of 5 or 10 µM. Compound was added to the donor compartment of the transwell plate for both apical to basolateral (A>B) and basolateral to apical (B>A) measurements, with blank HBSS being added to the receiver compartment. The transwell plate was then incubated at 37 °C, 5 % CO_2 for 2 hours.

Samples were taken at T=0 and T=2 hours determine the concentration of initial donor sample, and the concentration of receiver and donor wells post-incubation, respectively. The samples were quenched with solvent containing analytical internal standard (IS). The supernatant was mixed with an appropriate volume of ultra-pure water before LC-MS/MS analysis.

Samples were analysed using a Shimadzu liquid chromatography system coupled to a Triple Quad 6500+ (AB Inc., Illinois). Separation of compounds was achieved using an XSelect HSS T3 XP (2.1x50 mm) (SSA **9**) or Halo 90 Å Biphemyl (2.1x50mm) (SSA **1**, **2** and **3**) column at a temperature of 40 °C.A binary mobile phase gradient at a flow rate of 0.7 mL/min was used. Initial LC conditions comprised of 95 % solvent A (0.1 % formic acid in water) and 5 % solvent B (0.1 % formic acid in acetonitrile). This was ramped to 95 % solvent B at 0.7, held for 0.5 minutes and then immediately returned to initial conditions. Sample analysis was by electrospray ionisation.

Section 4: Chemical structures

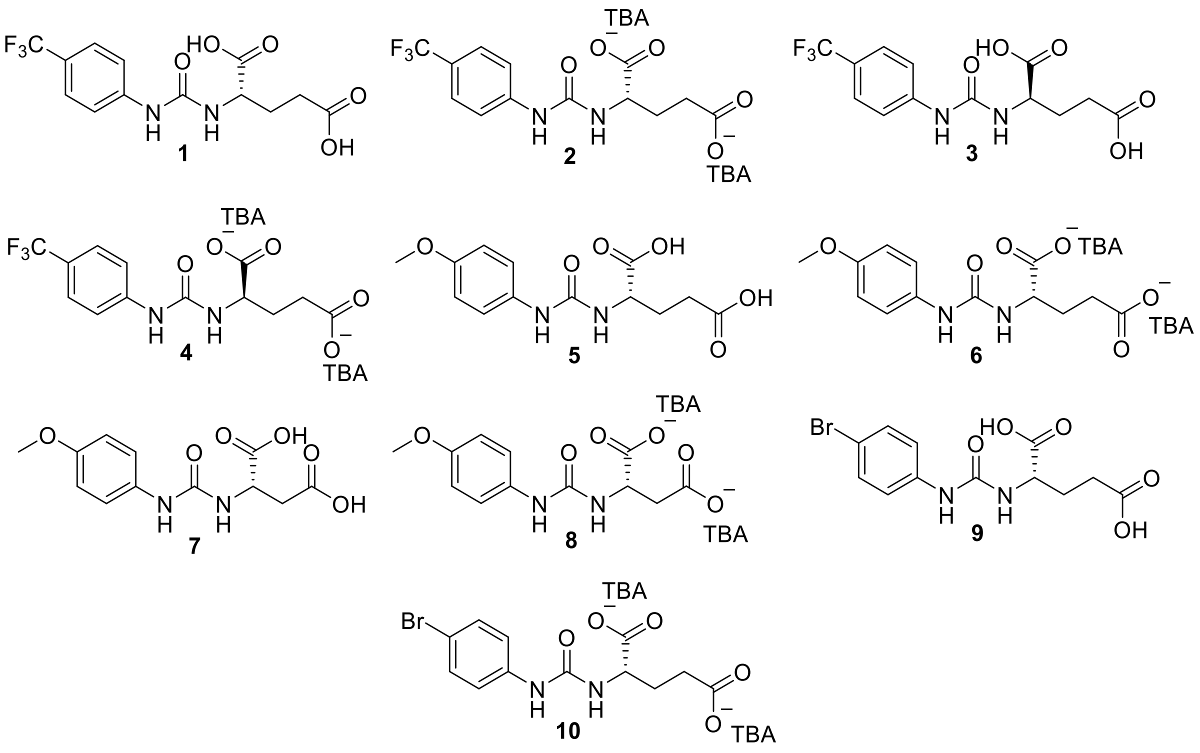

Figure S1 - Chemical structures of **1** - **10**. TBA = tetrabutylammonium.

Section 5: Chemical synthesis

SSA 1: 1-Isocyanato-4-(trifluoromethyl) benzene (0.57 mL, 4.00 mmol) was added to L-glutamic acid dimethyl ester hydrochloride (0.82 g, 4.15 mmol) and triethylamine (0.72 mL,

5.20 mmol) in acetonitrile (20 mL) and stirred at room temperature overnight. The mixture was then taken to complete dryness and dissolved in chloroform (5 mL), followed by dropwise additions of hexane (> 5 mL) resulting in precipitation. The precipitate was collected via filtration. The precipitate was dissolved in isopropanol (5 mL) and sodium hydroxide (2 mL, 2M) and stirred at room temperature for 4 hours. Hydrochloric acid (1M) was added dropwise until precipitation (~pH 5) and collected by filtration. The pure product was identified using NMR spectroscopy and collected as a white solid with a yield of 79 % (1.14 g, 3.41 mmol); melting point: > 200 °C; ^{1}H NMR (400 MHz, 298 K, DMSO-d_6): δ: 12.47 (bs, 1H), 9.03 (s, 1H), 7.58 (s, 4H), 6.62 - 6.61 (d, J = 7.32 Hz, 1H), 4.23 – 4.22 (q, J = 5.32 Hz, 1H), 2.36 – 2.23 (m, 2H), 2.10 – 2.00 (m, 1H), 1.98 – 1.77 (m, 1H); ^{13}C{^{1}H} NMR (100 MHz, 298 K, DMSO-d_6): δ: 173.9 (CO), 155.0 (CO), 144.4 (ArC), 126.3 (ArCH), 129.1 – 121.0 (q, J = 268.7 Hz, ArC), 122.0 – 119.9 (q, J = 31.8 Hz, CF_3), 121.0 (ArC), 118.0 (ArCH), 52.4 (CH), 30.7 (CH_2), 27.8 (CH_2); IR (film): ν = 3001 (NH stretch), 1662, 1471, 1304, 844; HRMS for the carboxylic acid-urea ($C_{13}H_{13}F_3N_2O_5$) (ESI$^-$): m/z: act = 333.0690 [M]$^-$, cal = 333.0777 [M]$^-$.

SSA 2: Tetrabutylammonium hydroxide (1.50 mL, 1.50 mmol) was added to **1** (0.50 g, 1.50 mmol) in methanol. The mixture was then taken to complete dryness. The pure product was identified using NMR spectroscopy and collected as a clear oil with a yield of 100 % (1.23 g, 1.50 mmol); melting point: oil; ^{1}H NMR (400 MHz, 343 K, DMSO-d_6): δ: 10.08 (s, 1H), 7.81 (s, 2H), 7.45 – 7.43 (d, J = 6.36 Hz, 2H), 6.23 (s, 1H), 3.82 – 3.76 (d, J = 19.24 Hz, 1H), 2.23 – 2.13 (m, 1H), 2.02 – 2.01 (m, 1H), 1.69 – 1.58 (m, 18H), 1.38 – 1.35(m, 16H), 0.98 – 0.96 (t, J = 7.28 Hz, 24H); ^{13}C{^{1}H} NMR (100 MHz, 343 K, DMSO-d_6): δ: 176.0 (CO), 173.8 (CO), 155.6 (CO), 146.5 (ArC), 125.6 (ArCH), 129.5 – 121.4 (q, J = 266.6 Hz, ArC), 120.3 - 199.0 (q, J = 32.1 Hz, CF_3), 118.0 (ArCH), 58.5 (CH_2), 55.2 (CH), 34.5 (CH_2), 24.0 (CH_2), 24.0 (CH_2), 19.7 (CH_2), 13.7 (CH_3); IR (film): ν = 2995 (NH stretch), 1669, 1437, 1300, 874; HRMS for the carboxylate-urea ($C_{13}H_{11}F_3N_2O_5^{2-}$) (ESI$^-$): m/z: act = 333.0692 [M]$^-$, cal = 333.0631 [M]$^-$.

SSA 3: 1-Isocyanato-4-(trifluoromethyl) benzene (0.57 mL, 4.00 mmol) was added to D-glutamic acid dimethyl ester hydrochloride (0.82 g, 4.15 mmol) and triethylamine (0.72 mL, 5.20 mmol) in acetonitrile (20 mL) and stirred at room temperature overnight. The mixture was then taken to complete dryness and dissolved in chloroform (5 mL), followed by dropwise additions of hexane (> 5 mL) resulting in precipitation. The precipitate was collected via filtration. The precipitate was dissolved in isopropanol (5 mL) and sodium hydroxide (2 mL, 2M) and stirred at room temperature for 4 hours. Hydrochloric acid (1M) was added dropwise until precipitation (~pH 5) and collected by filtration. The pure product was identified using NMR spectroscopy and collected as a white solid with a yield of 73 % (0.98 g, 2.93 mmol); melting point: > 200 °C; ^{1}H NMR (400 MHz, 298 K, DMSO-d_6): δ: 12.25 (bs, 1H), 9.04 (s, 1H), 7.58 (s, 4H), 6.63 - 6.61 (d, J = 8.12 Hz, 1H), 4.24 – 4.19 (q, J = 8.20 Hz, 1H), 2.36 – 2.24 (m, 2H), 2.06 – 2.01 (m, 1H), 2.00 – 1.77 (m, 1H); ^{13}C{^{1}H} NMR (100 MHz, 298 K, DMSO-d_6): δ: 173.9 (CO), 155.0 (CO), 144.4 (ArC), 126.3 (ArCH), 129.1 – 121.0 (q, J = 268.7 Hz, ArC), 122.5 – 121.5 (q, J = 31.9 Hz, CF_3), 118.0 (ArCH), 52.3 (CH), 30.5 (CH_2), 27.9 (CH_2); IR (film): ν = 2999 (NH stretch), 1669, 1469, 1314, 840; HRMS for the carboxylic acid-urea ($C_{13}H_{13}F_3N_2O_5$) (ESI$^-$): m/z: act = 333.0689 [M]$^-$, cal = 333.0777 [M]$^-$.

SSA 4: Tetrabutylammonium hydroxide (1.50 mL, 1.50 mmol) was added to **3** (0.50 g, 1.50 mmol) in methanol. The mixture was then taken to complete dryness. The pure product was identified using NMR spectroscopy and collected as a clear oil with a yield of 100 % (1.23 g, 1.50 mmol); melting point: oil; ^{1}H NMR (400 MHz, 343 K, DMSO-d_6): δ: 10.08 (s, 1H), 7.81 (s, 2H), 7.48 – 7.46 (d, *J* = 6.36 Hz, 2H), 6.23 (s, 1H), 3.80 - 3.77 (d, *J* = 19.24 Hz, 1H), 2.23 – 2.13 (m, 1H), 2.02 – 2.01 (m, 1H), 1.69 – 1.58 (m, 18H), 1.38 – 1.35 (m, 16H), 0.97 – 0.93 (t, *J* = 7.40 Hz, 24H); ^{13}C{^{1}H} NMR (100 MHz, 343 K, DMSO-d_6): δ: 176.4 (CO), 173.9 (CO), 155.8 (CO), 146.6 (ArC), 125.5 (ArCH), 129.5 – 121.4 (q, *J* = 269.9 Hz, ArC), 120.8 – 119.9 (q, *J* = 31.7 Hz, CF_3), 118.2 (ArCH), 58.5 (CH_2), 55.6 (CH), 35.3 (CH_2), 30.1 (CH_2), 23.6 (CH_2), 19.5 (CH_2), 13.7 (CH_3); IR (film): ν = 3008 (NH stretch), 1710, 1449, 1312, 881; HRMS for the carboxylate-urea ($C_{13}H_{11}F_3N_2O_5^{2-}$) (ESI$^-$): m/z: act = 333.0691 [M]$^-$, cal = 333.0631 [M]$^-$.

SSA 5: 4-Methoxyphenyl isocyanate (0.89 g, 6.00 mmol) was added to L-glutamic acid dimethyl ester hydrochloride (1.31 g, 6.20 mmol) and triethylamine (1.08 mL, 7.80 mmol) in acetonitrile (30 mL) and stirred at room temperature overnight. The mixture was then taken to complete dryness and dissolved in chloroform (5 mL), followed by dropwise additions of hexane (> 5 mL) resulting in precipitation. The precipitate was collected via filtration. The precipitate was dissolved in isopropanol (5 mL) and sodium hydroxide (2 mL, 2M) and stirred at room temperature for 4 hours. Hydrochloric acid (1M) was added dropwise until precipitation (~pH 5) and collected by filtration. The pure product was identified using NMR spectroscopy and collected as a white solid with a yield of 73 % (1.61 g, 5.45 mmol); melting point: > 200 °C; ^{1}H NMR (400 MHz, 298 K, DMSO-d_6): δ: 12.37 (bs, 1H), 8.39 (s, 1H), 7.28 – 7.26 (d, *J* = 9.04 Hz, 2H), 6.82 – 6.80 (d, *J* = 9.04 Hz, 2H), 6.33 - 6.31 (d, *J* = 8.04 Hz, 1H), 4.19 – 4.17 (q, *J* = 5.04 Hz, 1H), 3.69 (s, 3H), 2.50 – 2.21 (m, 2H), 2.04 – 1.98 (m, 1H), 1.96 – 1.73 (m, 1H); ^{13}C{^{1}H} NMR (100 MHz, 298 K, DMSO-d_6): δ: 174.2 (CO), 173.9 (CO), 155.6 (CO), 154.9 (CO), 133.9 (ArC), 120.2 (ArCH), 114.6 (ArCH), 55.8 (CH_3), 52.3 (CH), 30.6 (CH_2), 28.0 (CH_2); IR (film): ν = 3002 (NH stretch), 1701, 1441, 1398, 888; HRMS for the carboxylic acid-urea ($C_{13}H_{16}N_2O_6$) (ESI$^-$): m/z: act = 295.0923 [M]$^-$, cal = 295.1008 [M]$^-$.

SSA 6: Tetrabutylammonium hydroxide (3.00 mL, 3.00 mmol) was added to **5** (0.89 g, 3.00 mmol) in methanol. The mixture was then taken to complete dryness. The pure product was identified using NMR spectroscopy and collected as a clear oil with a yield of 100 % (2.34 g, 3.00 mmol); melting point: oil; ^{1}H NMR (400 MHz, 343 K, DMSO-d_6): δ: 9.65 (s, 1H), 7.46 – 7.44 (d, *J* = 7.06 Hz, 2H), 6.76 – 7.73 (d, *J* = 9.64 Hz, 2H), 5.98 (s, 1H), 3.78 – 3.74 (d, *J* = 5.80 Hz, 1H), 3.69 (s, 3H), 2.25 – 2.17 (m, 1H), 2.04 – 1.96 (m, 1H), 1.69 – 1.58 (m, 18H), 1.38 – 1.35 (m, 16H), 0.97 – 0.93 (t, *J* = 7.28 Hz, 24H); ^{13}C{^{1}H} NMR (100 MHz, 343 K, DMSO-d_6): δ: 176.1 (CO), 174.2 (CO), 156.1 (CO), 153.9 (CO), 136.0 (ArC), 119.9 (ArCH), 114.1 (ArCH), 58.5 (CH_2), 55.8 (CH_3), 55.1 (CH), 34.9 (CH_2), 31.8 (CH_2), 23.9 (CH_2), 19.5 (CH_2), 13.8 (CH_3); IR (film): ν = 2981 (NH stretch), 1787, 1449, 1388, 901; HRMS for the carboxylate-urea ($C_{13}H_{14}N_2O_6^{2-}$) (ESI$^-$): m/z: act = 295.0923 [M]$^-$, cal = 295.0863 [M]$^-$.

SSA 7: 4-Methoxyphenyl isocyanate (0.89 g, 6.00 mmol) was added to L-aspartic acid dimethyl ester hydrochloride (1.23 g, 6.20 mmol) and triethylamine (1.08 mL, 7.80 mmol) in acetonitrile (30 mL) and stirred at room temperature overnight. The mixture was then taken to complete dryness and dissolved in chloroform (5 mL), followed by dropwise additions of hexane (> 5

mL) resulting in precipitation. The precipitate was collected via filtration. The precipitate was dissolved in isopropanol (5 mL) and sodium hydroxide (2 mL, 2M) and stirred at room temperature for 4 hours. Hydrochloric acid (1M) was added dropwise until precipitation (~pH 5) and collected by filtration. The pure product was identified using NMR spectroscopy and collected as a white solid with a yield of 66 % (1.12 g, 3.96 mmol); melting point: > 194 °C; ^{1}H NMR (400 MHz, 298 K, DMSO-d_6): δ: 12.42 (bs, 1H), 8.63 (s, 1H), 7.28 – 7.26 (d, *J* = 9.48 Hz, 2H), 6.82 – 6.80 (d, *J* = 8.40 Hz, 2H), 6.42 - 6.34 (d, *J* = 7.36 Hz, 1H), 4.50 – 4.46 (q, *J* = 5.24 Hz, 1H), 3.69 (s,3H), 2.79 – 2.64 (m, 2H); ^{13}C{^{1}H} NMR (100 MHz, 298 K, DMSO-d_6): δ: 173.3 (CO), 172.3 (CO), 155.4 (CO), 154.9 (CO), 133.9 (ArC), 120.1 (ArCH), 114.6 (ArCH), 55.8 (CH_3), 49.6 (CH), 37.5 (CH_2); IR (film): ν = 3011 (NH stretch), 1714, 1461, 1402, 826; HRMS for the carboxylic acid-urea ($C_{12}H_{14}N_2O_6$) (ESI$^-$): m/z: act = 281.0773 [M]$^-$, cal = 281.0852 [M]$^-$.

SSA 8: Tetrabutylammonium hydroxide (3.00 mL, 3.00 mmol) was added to **7** (0.85 g, 3.00 mmol) in methanol. The mixture was then taken to complete dryness. The pure product was identified using NMR spectroscopy and collected as a clear oil with a yield of 100 % (2.30 g, 3.00 mmol); melting point: oil; ^{1}H NMR (400 MHz, 298 K, DMSO-d_6): δ: 11.88 (s, 1H), 7.43 – 7.41 (d, *J* = 10.08 Hz, 2H), 6.74 – 6.72 (d, *J* = 8.80 Hz, 2H), 5.79 (s, 1H), 4.05 – 3.72 (m, 4H), 3.68 - 3.15 (m, 16H), 2.24 – 2.28 (m, 1H), 1.85 – 1.82 (m, 1H), 1.60 – 1.53 (m, 16H), 1.35 – 1.26 (m, 16H), 0.95 – 0.91 (m, 24H); ^{13}C{^{1}H} NMR (100 MHz, 298 K, DMSO-d_6): δ: 176.2 (CO), 173.9 (CO), 157.6 (CO), 153.5 (CO), 136.1 (ArC), 119.7 (ArCH), 113.8 (ArCH), 58.0 (CH_2), 56.0 (CH_3), 55.1 (CH), 46.8 (CH_2), 23.5 (CH_2), 19.7 (CH_2), 19.5 (CH_2), 13.8 (CH_3); IR (film): ν = 3003 (NH stretch), 1695, 1500, 1412, 832; HRMS for the carboxylate-urea ($C_{12}H_{12}N_2O_6^{2-}$) (ESI$^-$): m/z: act = 281.0769 [M]$^-$, cal = 281.0706[M]$^-$.

SSA 9: 4-Bromophenyl isocyanate (1.19 g, 6.00 mmol) was added to L-glutamic acid dimethyl ester hydrochloride (1.31 g, 6.20 mmol) and triethylamine (1.08 mL, 7.80 mmol) in acetonitrile (30 mL) and stirred at room temperature overnight. The mixture was then taken to complete dryness and dissolved in chloroform (5 mL), followed by dropwise additions of hexane (> 5 mL) resulting in precipitation. The precipitate was collected via filtration. The precipitate was dissolved in isopropanol (5 mL) and sodium hydroxide (2 mL, 2M) and stirred at room temperature for 4 hours. Hydrochloric acid (1M) was added dropwise until precipitation (~pH 5) and collected by filtration. The pure product was identified using NMR spectroscopy and collected as a white solid with a yield of 89 % (1.78 g, 5.39 mmol); melting point: > 198°C; ^{1}H NMR (400 MHz, 298 K, DMSO-d_6): δ: 12.45 (bs, 1H), 8.76 (s, 1H), 7.41 – 7.35 (dd, *J* = 9.12 Hz, 4H), 6.52 – 6.50 (d, *J* = 8.16 Hz, 1H), 4.24 – 4.18 (q, *J* = 8.24 Hz, 1H), 2.35 – 2.23 (m, 2H), 2.06 – 2.20 (m, 1H), 1.99 – 1.82 (m, 1H); ^{13}C{^{1}H} NMR (100 MHz, 298 K, DMSO-d_6): δ: 174.0 (CO), 173.9 (CO), 155.1 (CO), 140.1 (ArC), 131.8 (ArCH), 120.3 (ArCH), 113.2 (ArC), 52.3 (CH), 30.5 (CH_2), 27.9 (CH_2); IR (film): ν = 3000 (NH stretch), 1741, 1440, 1392, 836; HRMS for the carboxylic acid-urea ($C_{12}H_{13}BrN_2O_5$) (ESI$^-$): m/z: act = 344.0006 [M]$^-$, cal = 344.0008 [M]$^-$.

SSA 10: Tetrabutylammonium hydroxide (1.50 mL, 1.50 mmol) was added to **9** (0.49 g, 1.50 mmol) in methanol. The mixture was then taken to complete dryness. The pure product was identified using NMR spectroscopy and collected as a white oil with a yield of 100 % (1.22 g, 1.50 mmol); melting point: oil; ^{1}H NMR (400 MHz, 343 K, DMSO-d_6): δ: 10.16 (s, 1H), 7.56 (bs, 2H), 7.29 – 7.27 (d, *J* = 8.80 Hz, 2H), 6.17 (s, 1H), 3.76 (bs, 1H), 2.28 – 2.20 (m, 1H), 2.04 – 1.98

(m, 1H), 1.72 – 1.56 (m, 18H), 1.39 – 1.29 (m, 16H), 0.97 – 0.93 (t, *J* = 7.32 Hz, 24H); $^{13}C\{^{1}H\}$ NMR (100 MHz, 343 K, DMSO-d_6): δ: 176.0 (CO), 174.0 (CO), 156.1 (CO), 142.0 (ArC), 131.0 (ArC), 120.2 (ArCH), 111.6 (ArCH), 58.5 (CH_2), 54.8 (CH), 34.2 (CH_2), 31.0 (CH_2), 23.7 (CH_2), 19.7 (CH_2), 13.8 (CH_3); IR (film): ν = 3006 (NH stretch), 1777, 1495, 1400, 886; HRMS for the carboxylate-urea ($C_{12}H_{11}BrN_2O_5^{2-}$) (ESI$^-$): m/z: act = 343.9935 $[M]^-$, cal = 343.9962 $[M]^-$.

Section 6: Characterisation NMR spectra

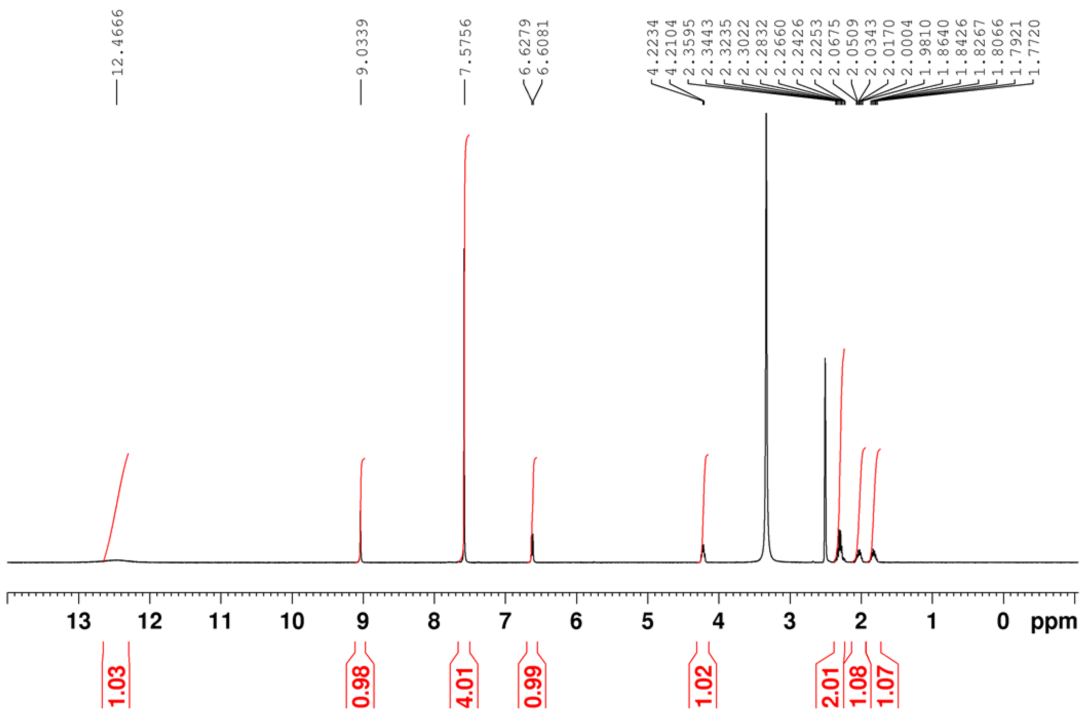

Figure S2 - ^{1}H NMR spectrum of **1** in DMSO-d_6 conducted at 298 K.

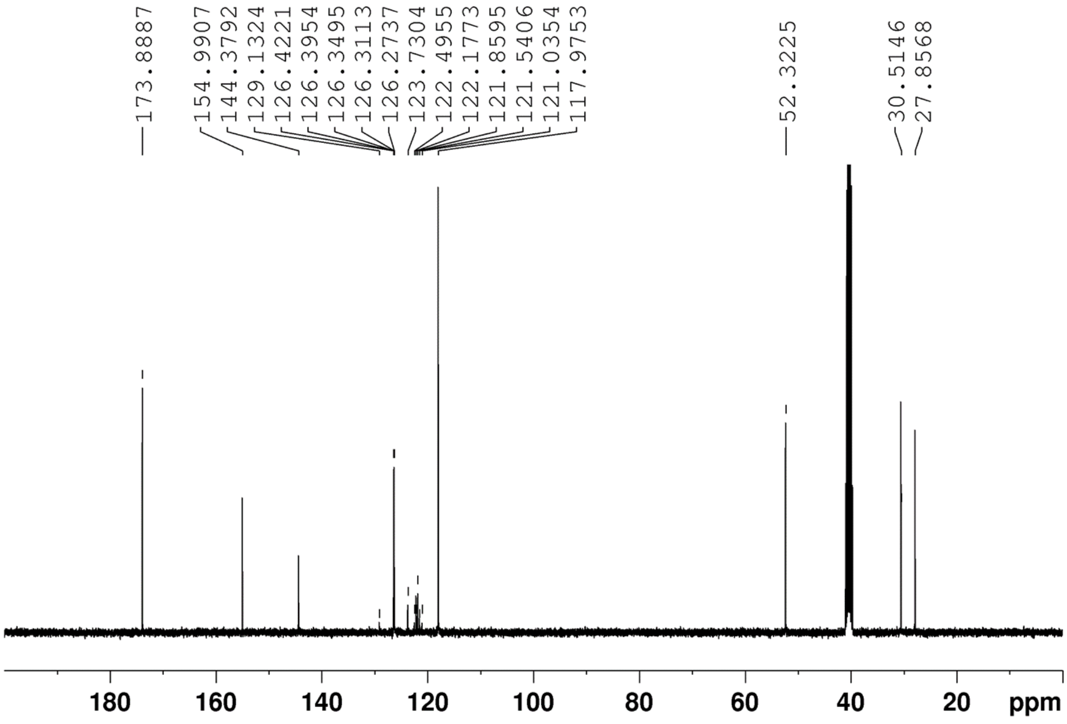

Figure S3 – ^{13}C {^{1}H} NMR spectrum of **1** in DMSO-d_6 conducted at 298 K.

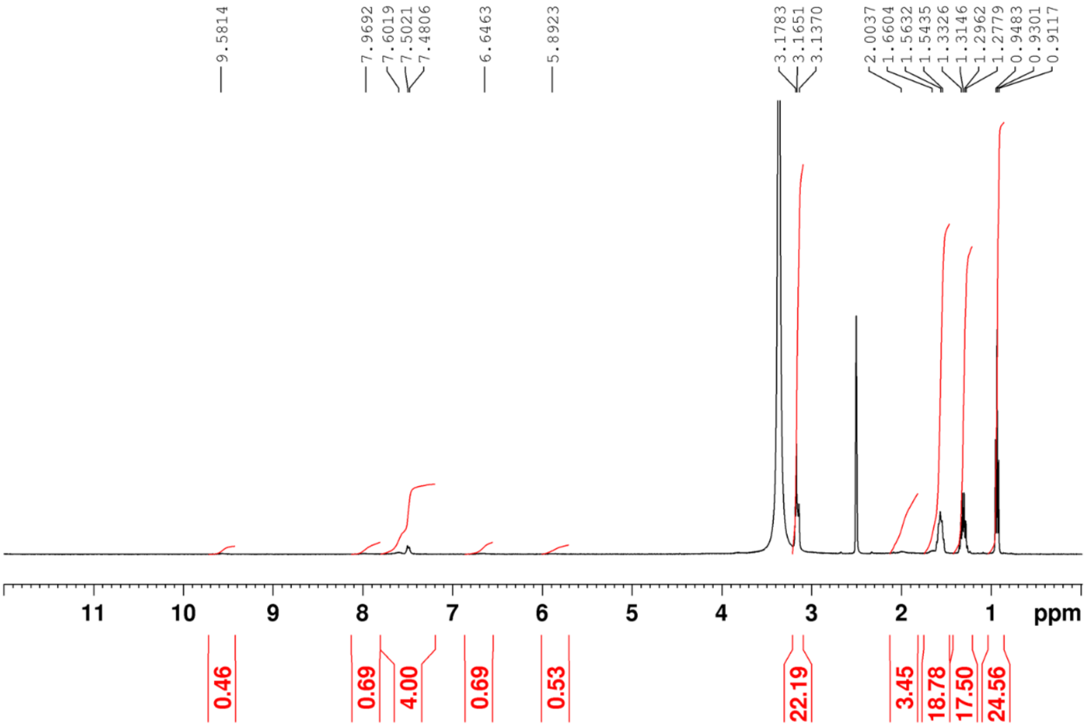

Figure S4 - ^{1}H NMR spectrum of **2** in DMSO-d_6 conducted at 298 K.

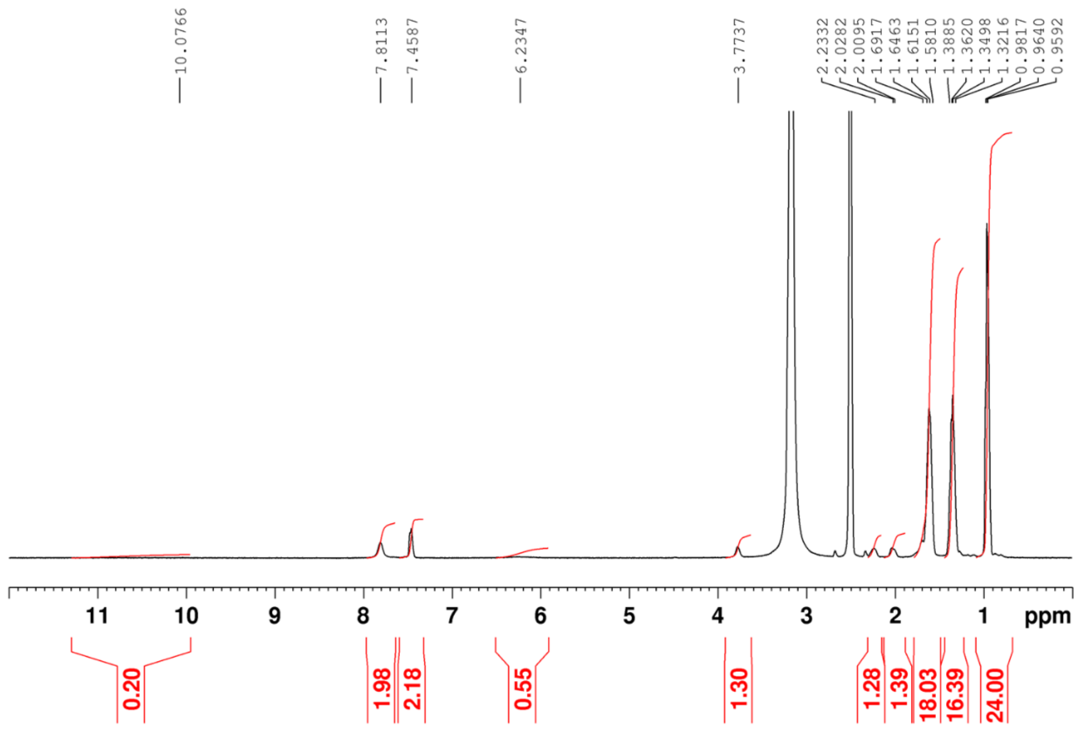

Figure S5 - ^{1}H NMR spectrum of **2** in DMSO-d_6 conducted at 343 K.

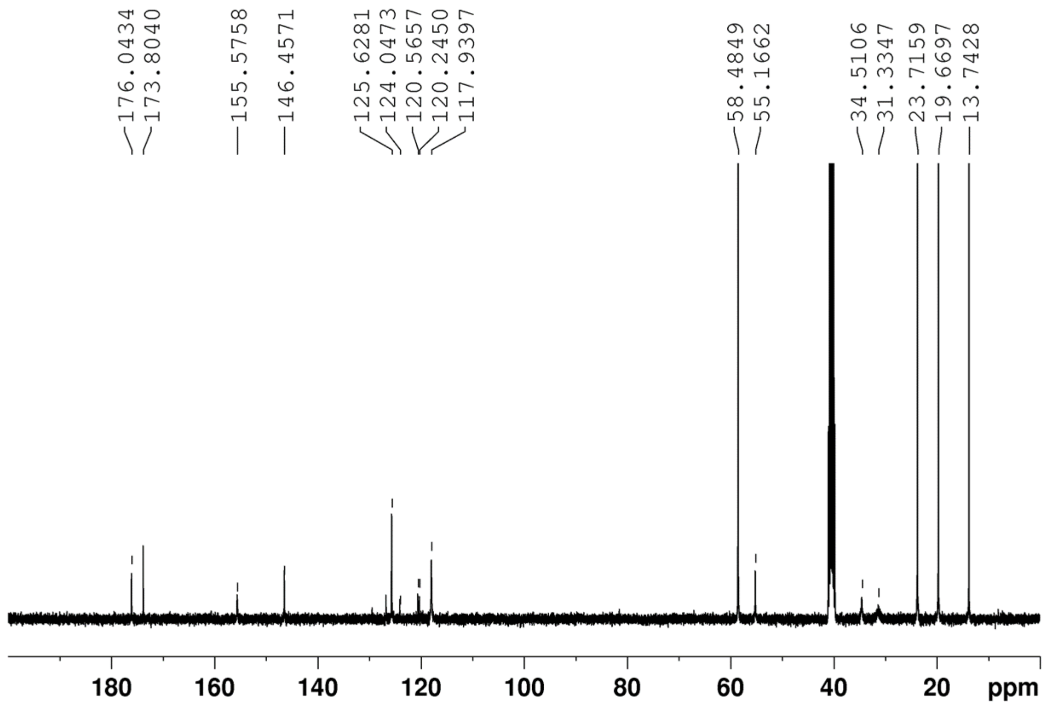

Figure S6 - ^{13}C {^{1}H} NMR spectrum of **2** in DMSO-d_6 conducted at 343 K.

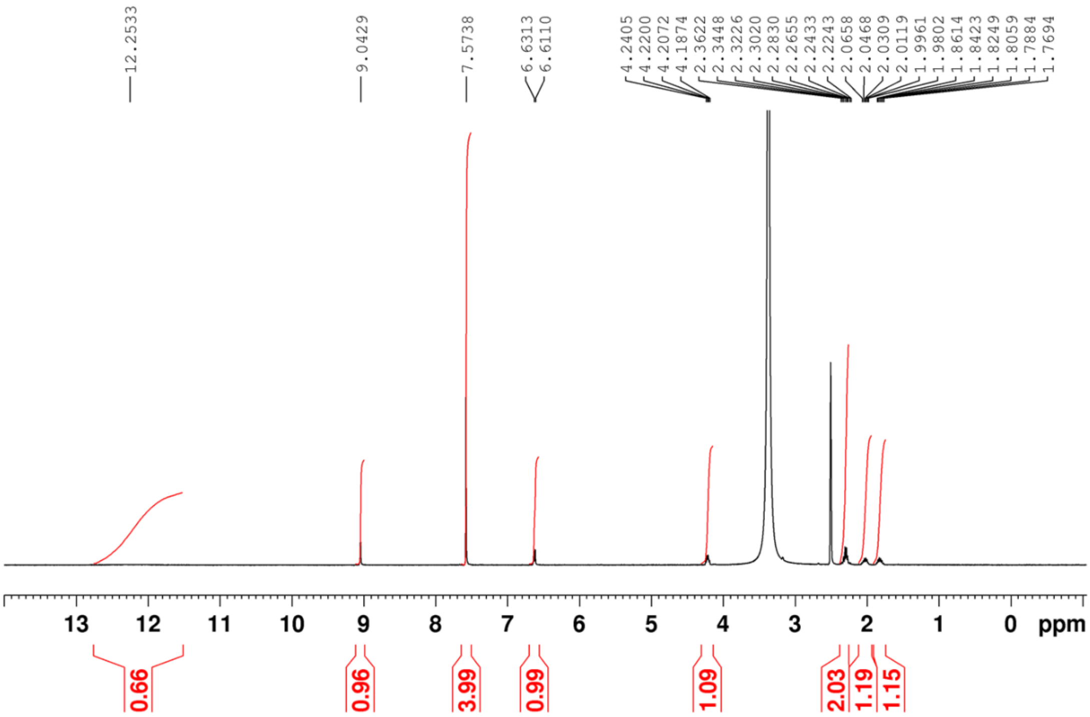

Figure S7 - ^{1}H NMR spectrum of **3** in DMSO-d_6 conducted at 298 K.

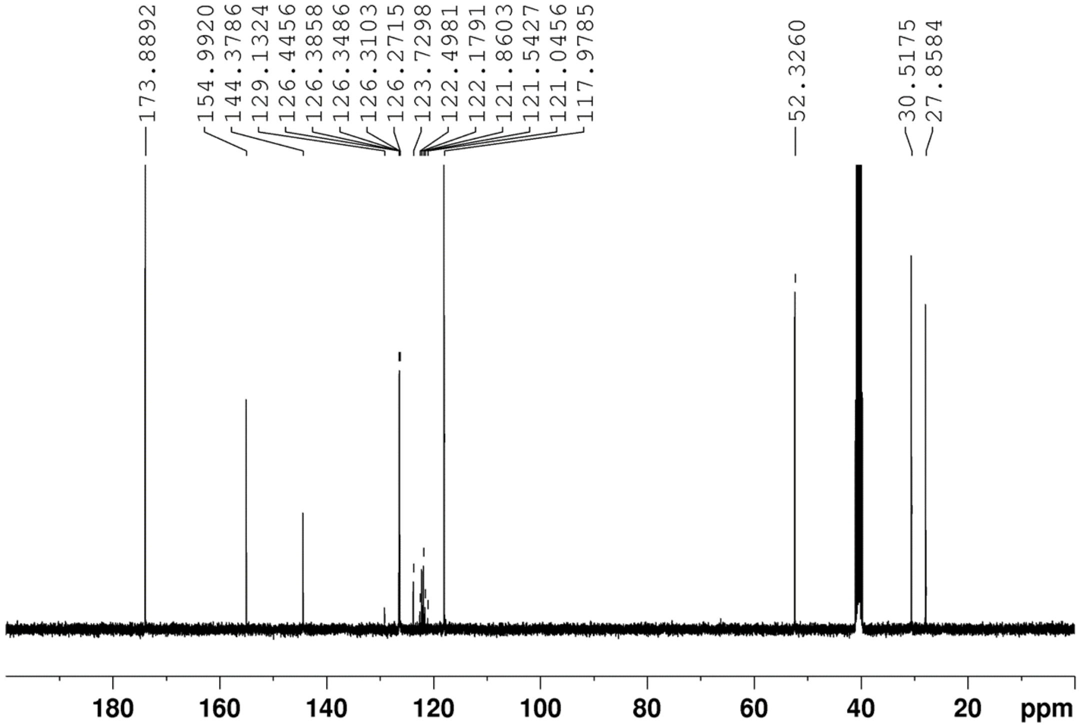

Figure S8 - ^{13}C {^{1}H} NMR spectrum of **3** in DMSO-d_6 conducted at 298 K.

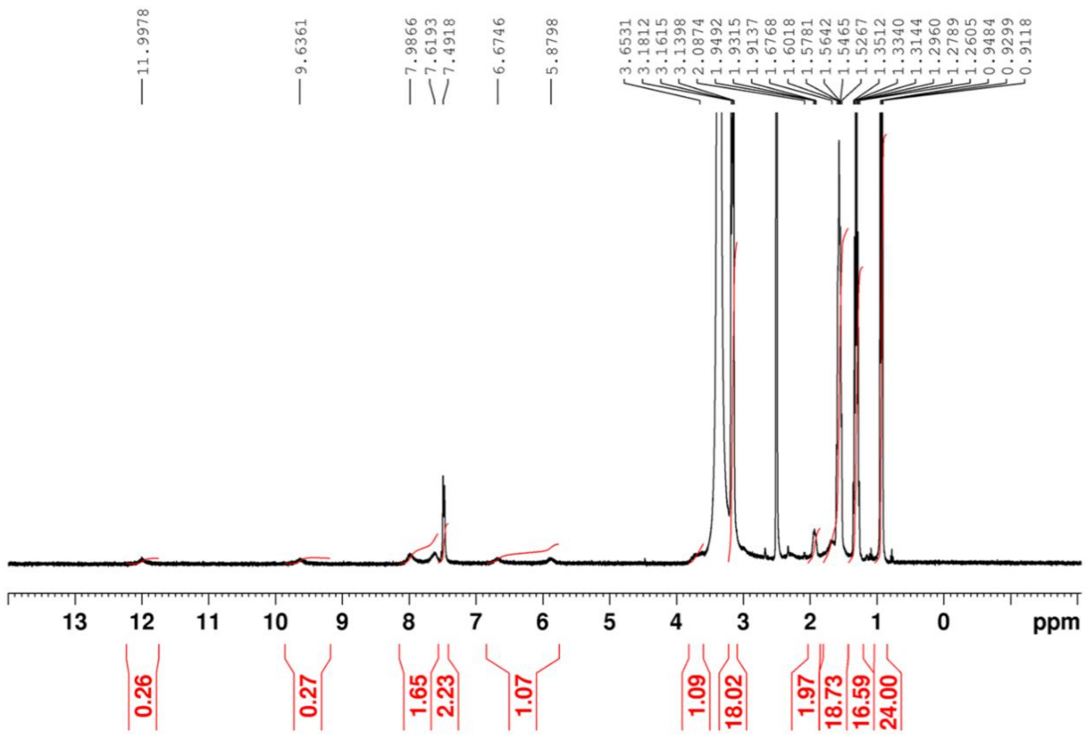

Figure S9 - ^{1}H NMR spectrum of **4** in DMSO-d_6 conducted at 298 K.

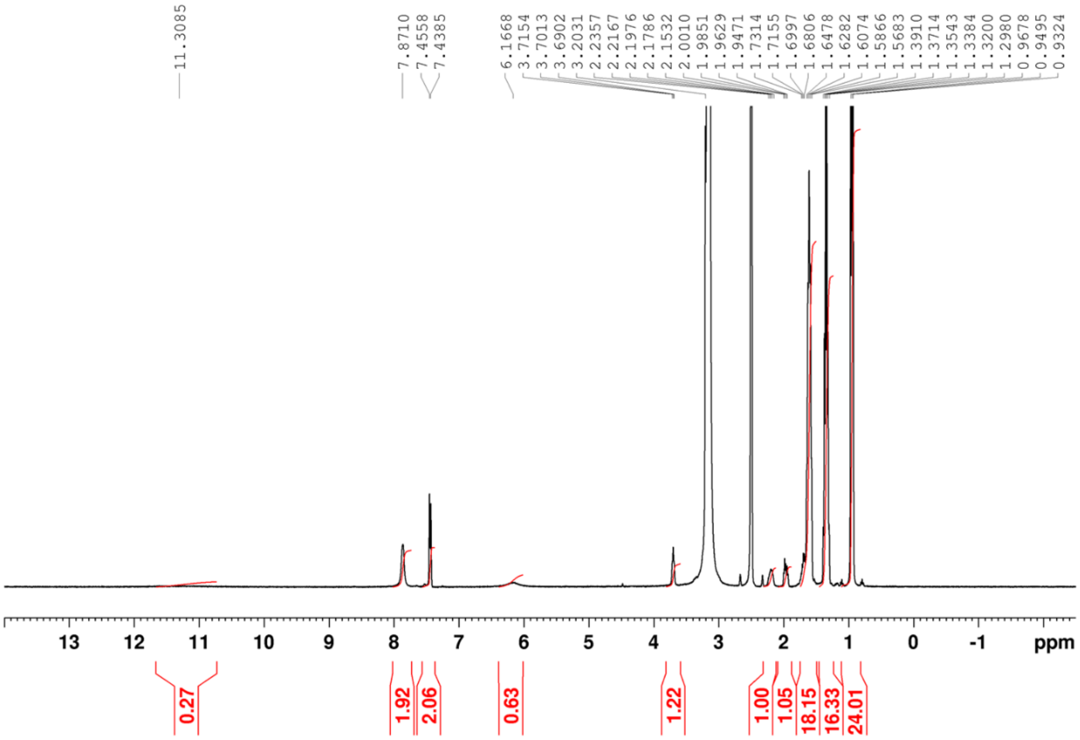

Figure S10 - ^{1}H NMR spectrum of **4** in DMSO-d_6 conducted at 343 K.

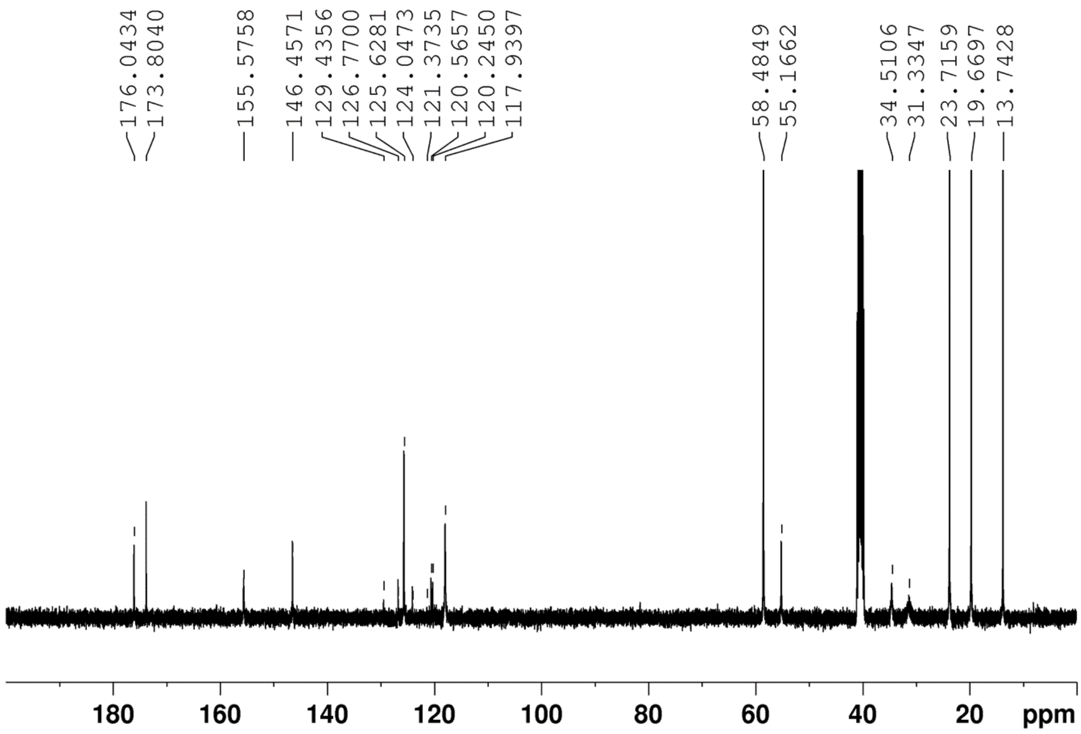

Figure S11 - ^{13}C {^{1}H} NMR spectrum of **4** in DMSO-d_6 conducted at 343 K.

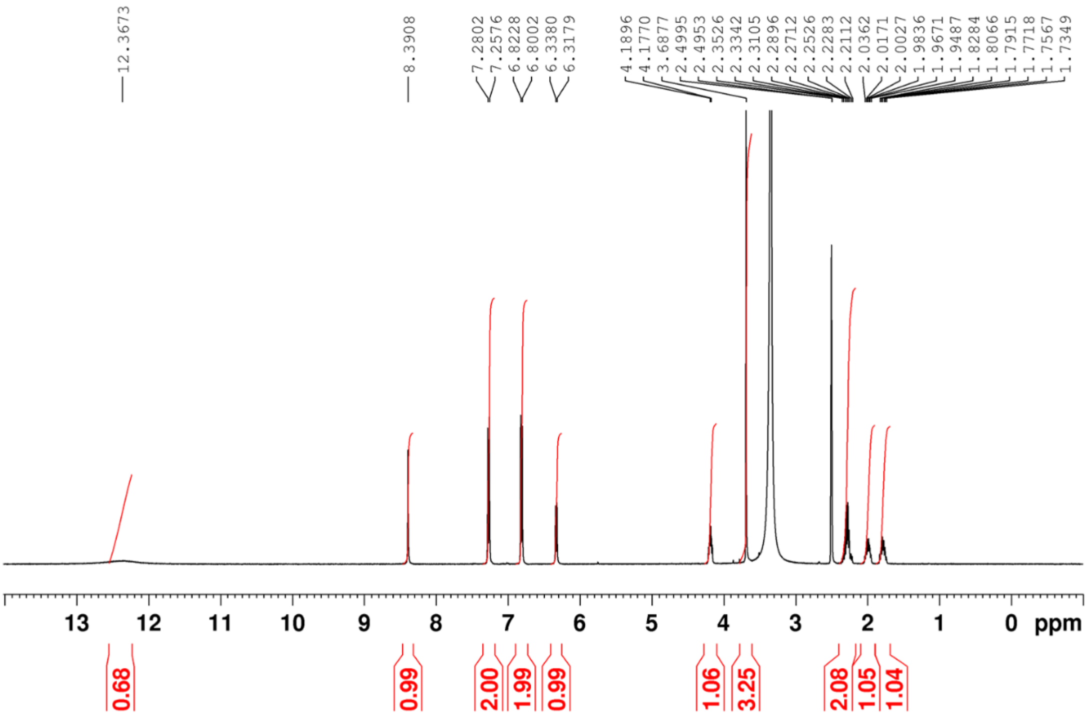

Figure S12 - ^{1}H NMR spectrum of **5** in DMSO-d_6 conducted at 298 K.

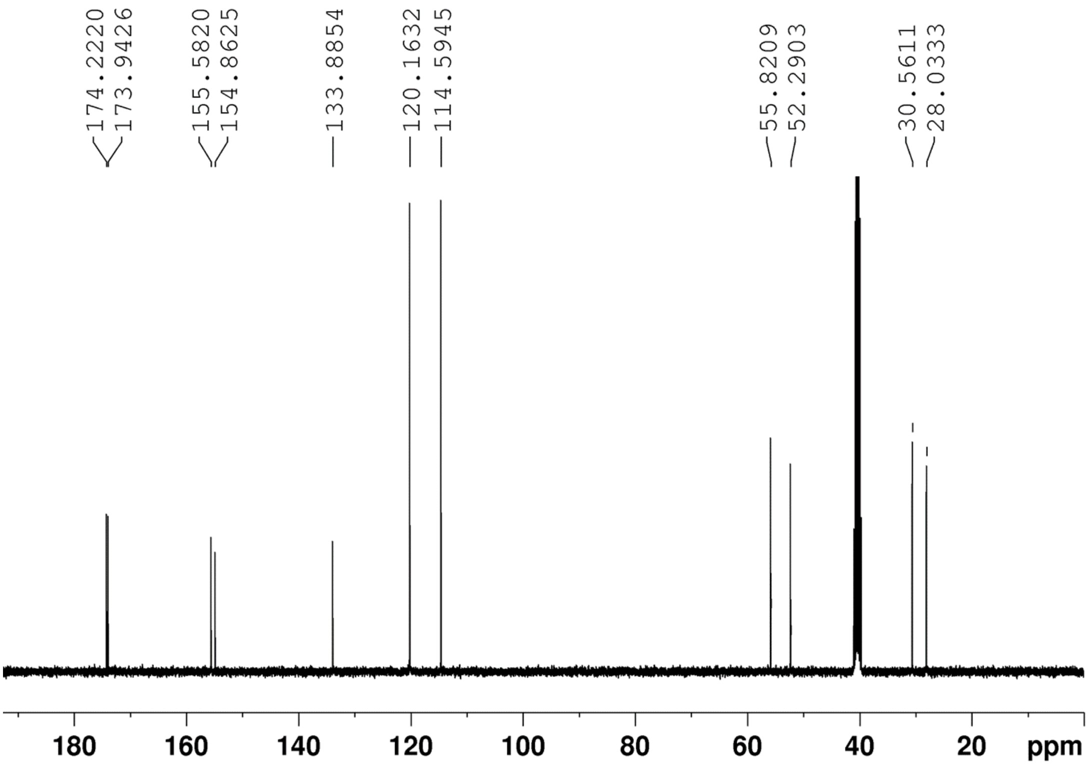

Figure S13 - ^{13}C {^{1}H} NMR spectrum of **5** in DMSO-d_6 conducted at 298 K.

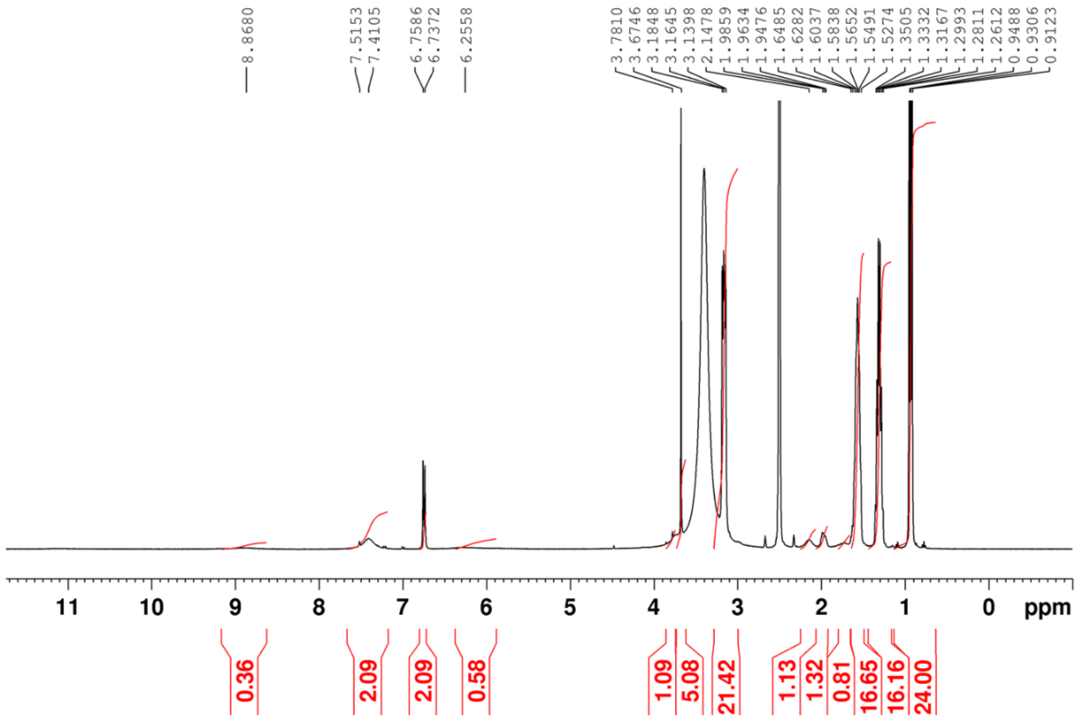

Figure S14 - ^{1}H NMR spectrum of **6** in DMSO-d_6 conducted at 298 K.

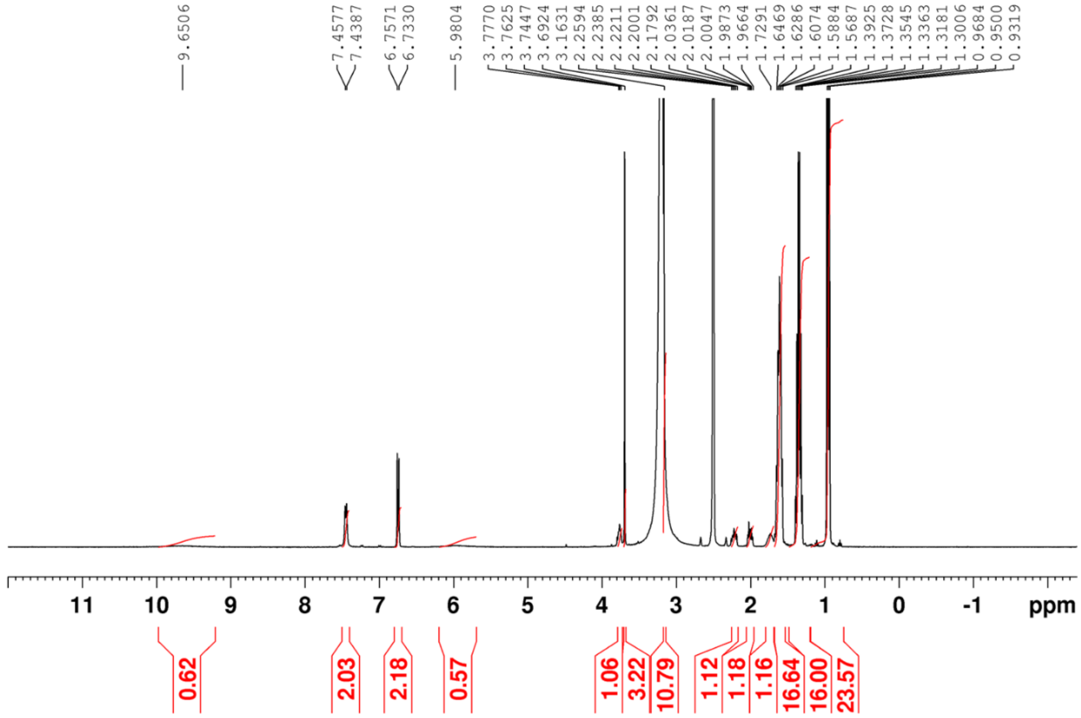

Figure S15 - ^{1}H NMR spectrum of **6** in DMSO-d_6 conducted at 343 K.

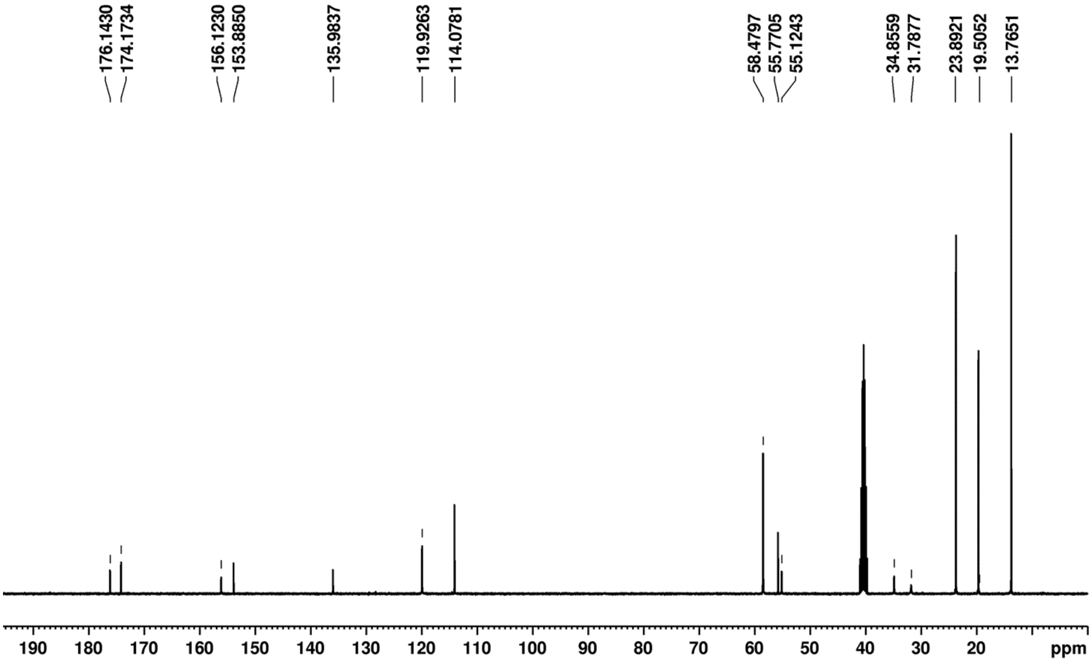

Figure S16 - ^{13}C {^{1}H} NMR spectrum of **6** in DMSO-d_6 conducted at 343 K.

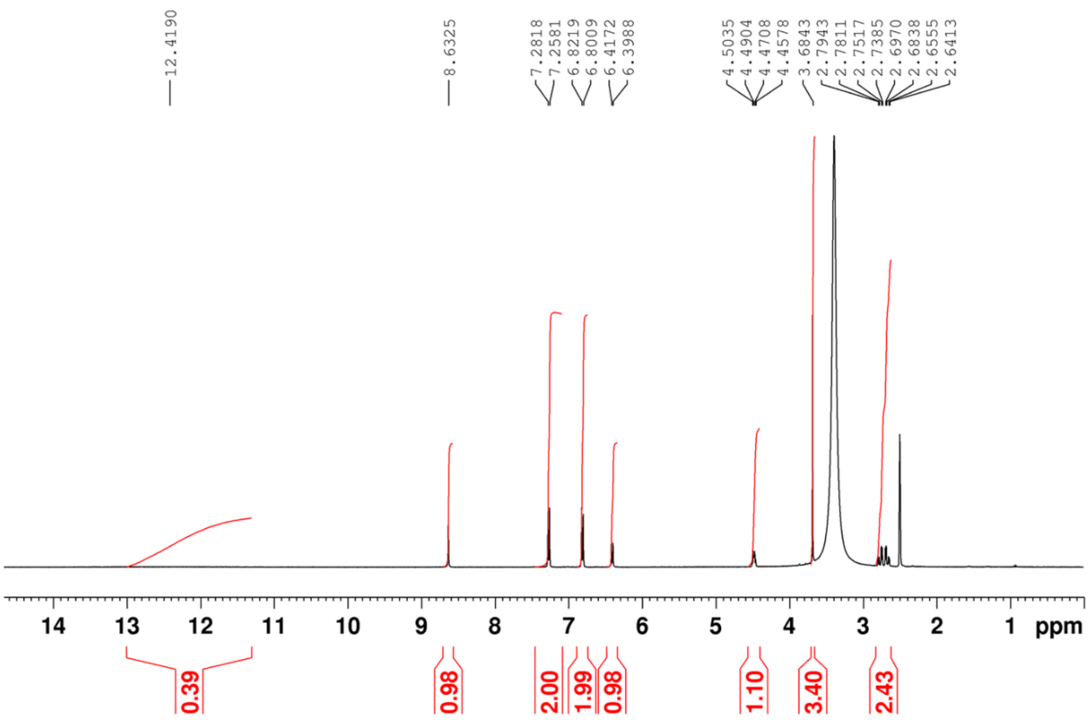

Figure S17 - ^{1}H NMR spectrum of **7** in DMSO-d_6 conducted at 298 K.

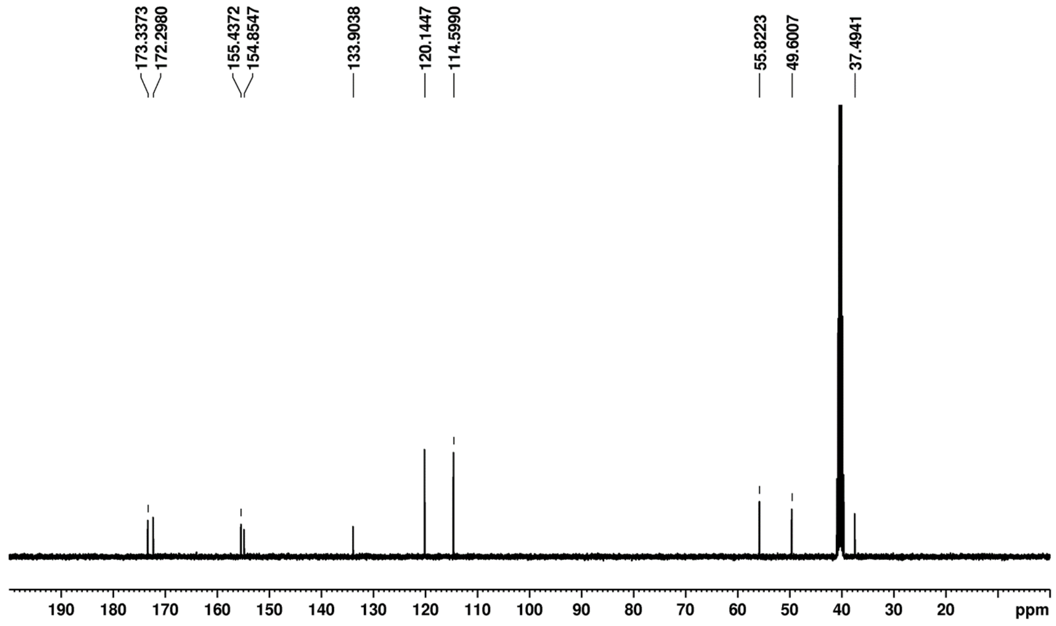

Figure S18 - ^{13}C {^{1}H} NMR spectrum of **7** in DMSO-d_6 conducted at 298 K.

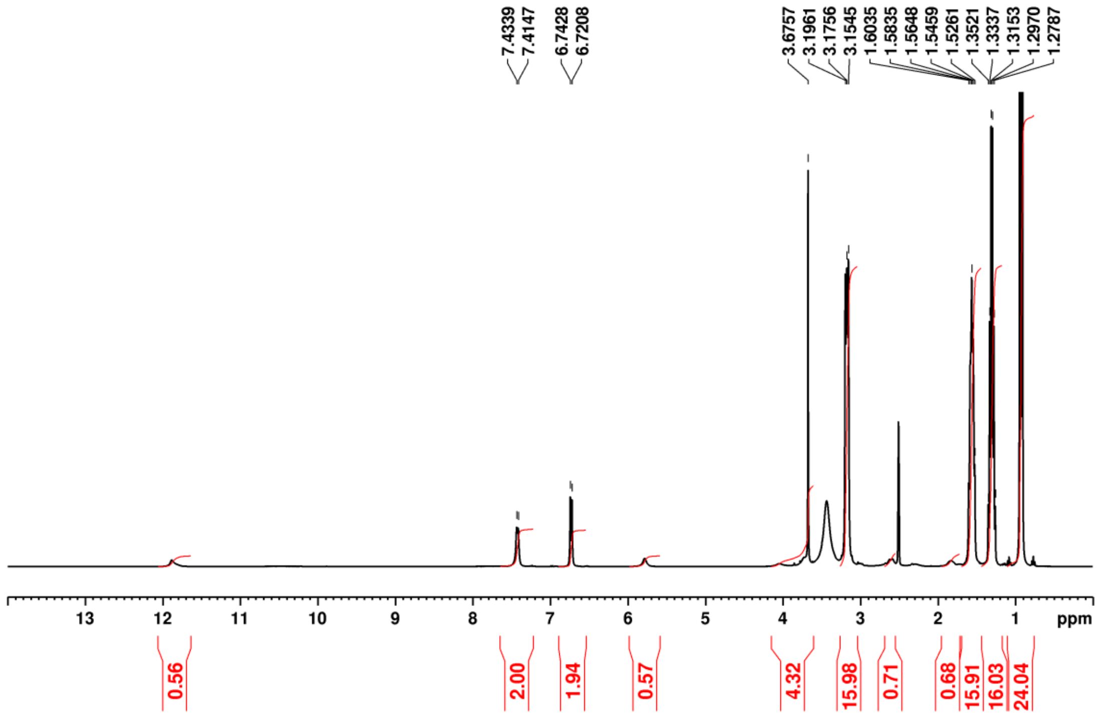

Figure S19 - ^{1}H NMR spectrum of **8** in DMSO-d_6 conducted at 298 K.

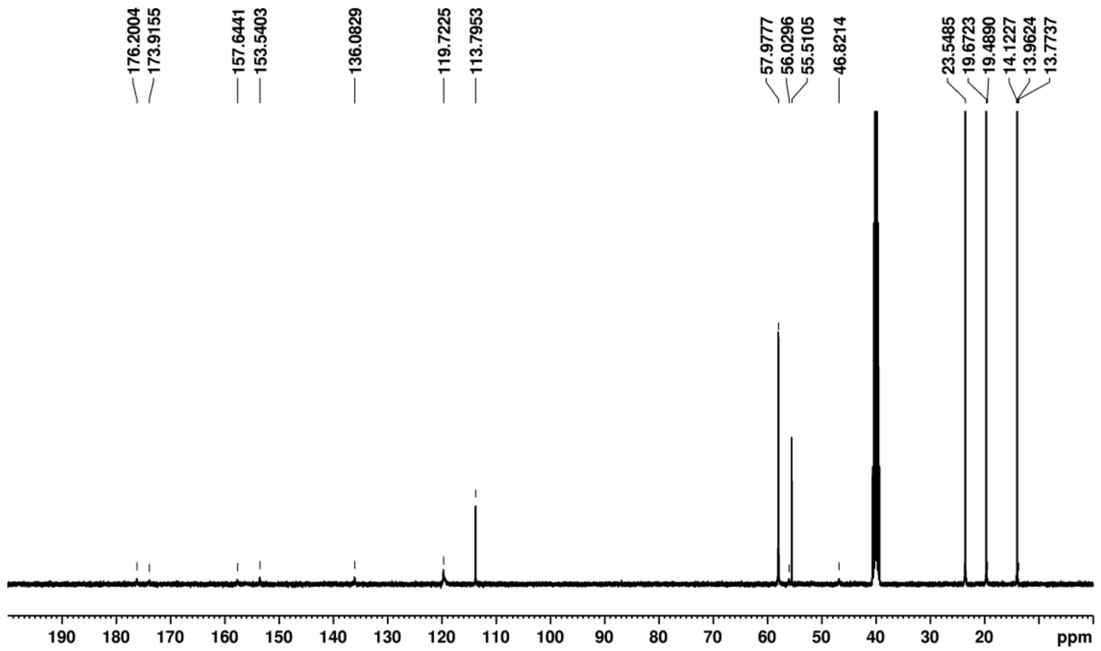

Figure S20 - ^{13}C {^{1}H} NMR spectrum of **8** in DMSO-d_6 conducted at 298 K.

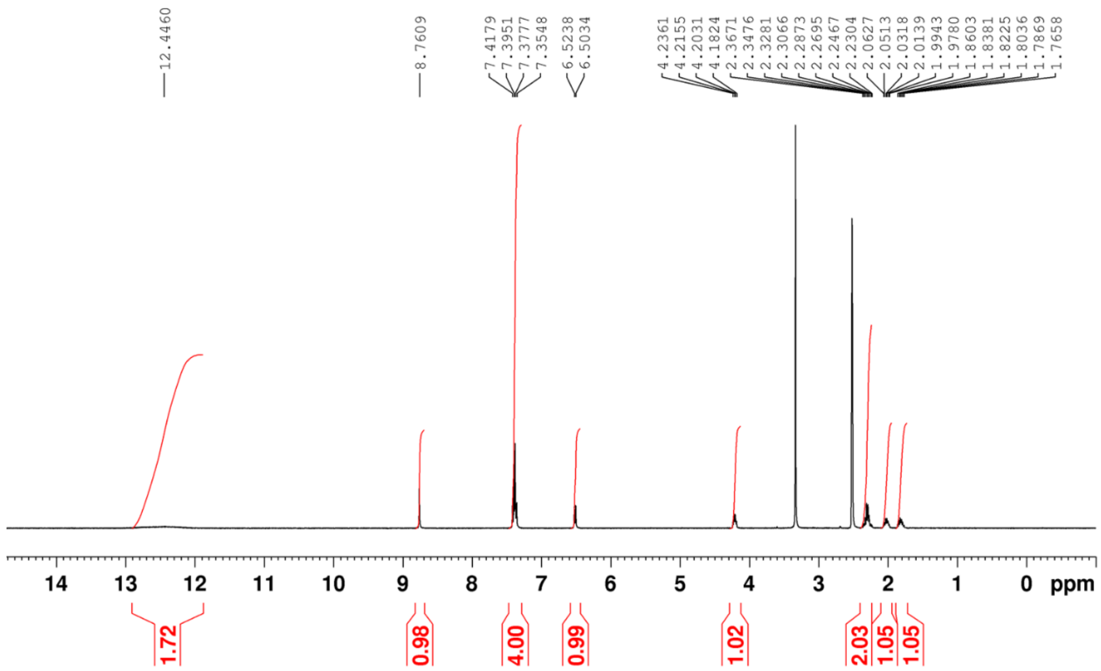

Figure S21 - ^{1}H NMR spectrum of **9** in DMSO-d_6 conducted at 298 K.

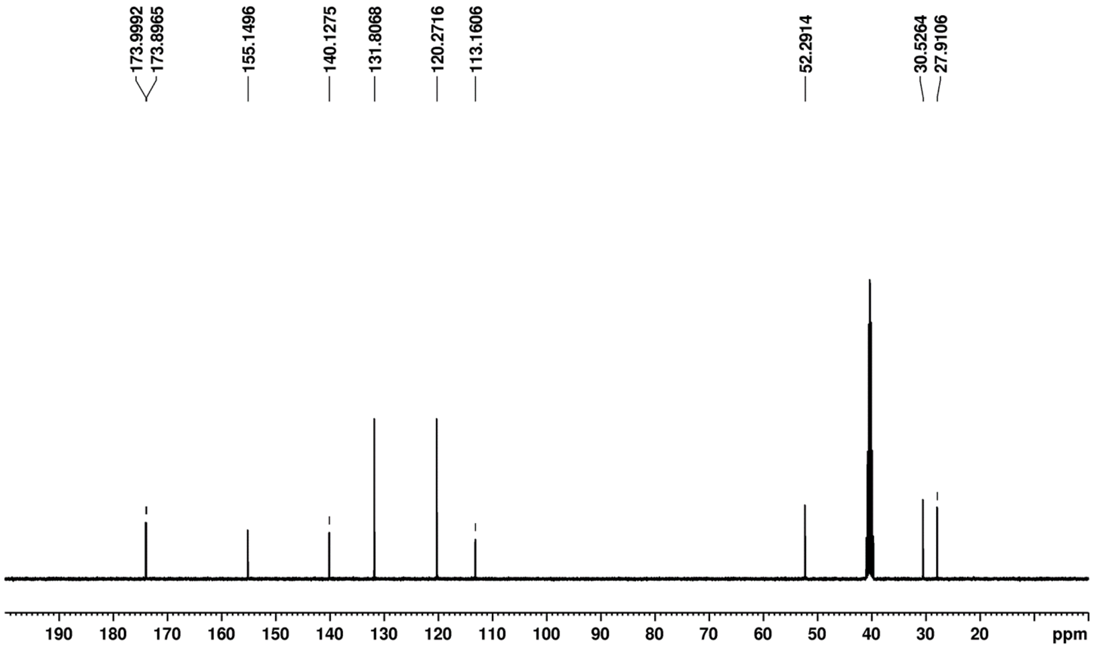

Figure S22 - ^{13}C {^{1}H} NMR spectrum of **9** in DMSO-d_6 conducted at 298 K.

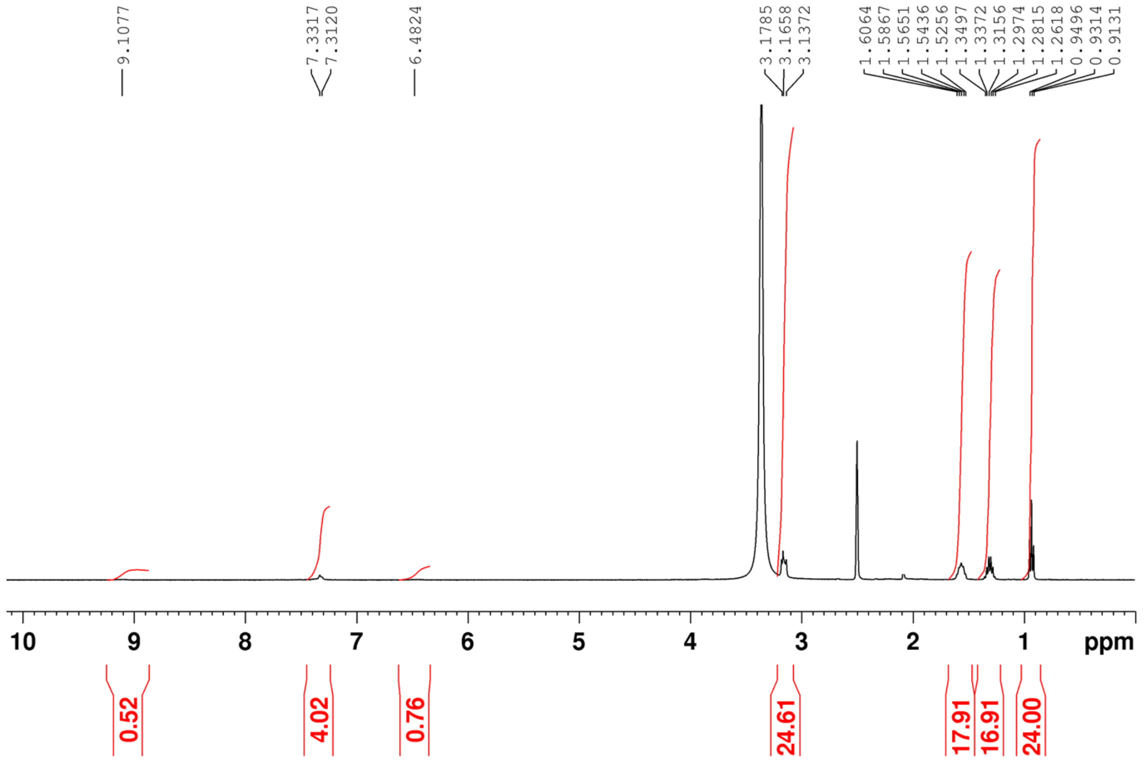

Figure S23 - ^{1}H NMR spectrum of **10** in DMSO-d_6 conducted at 298 K.

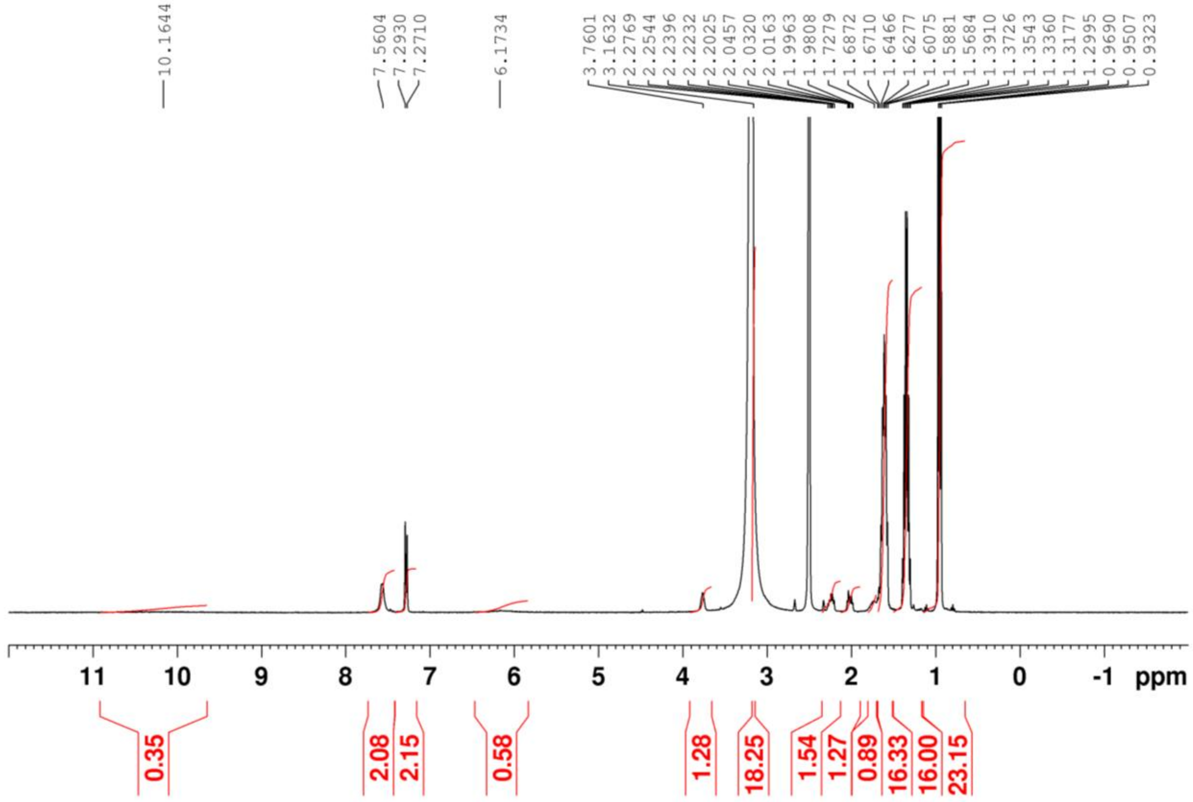

Figure S24 - ^{1}H NMR spectrum of **10** in DMSO-d_6 conducted at 343 K.

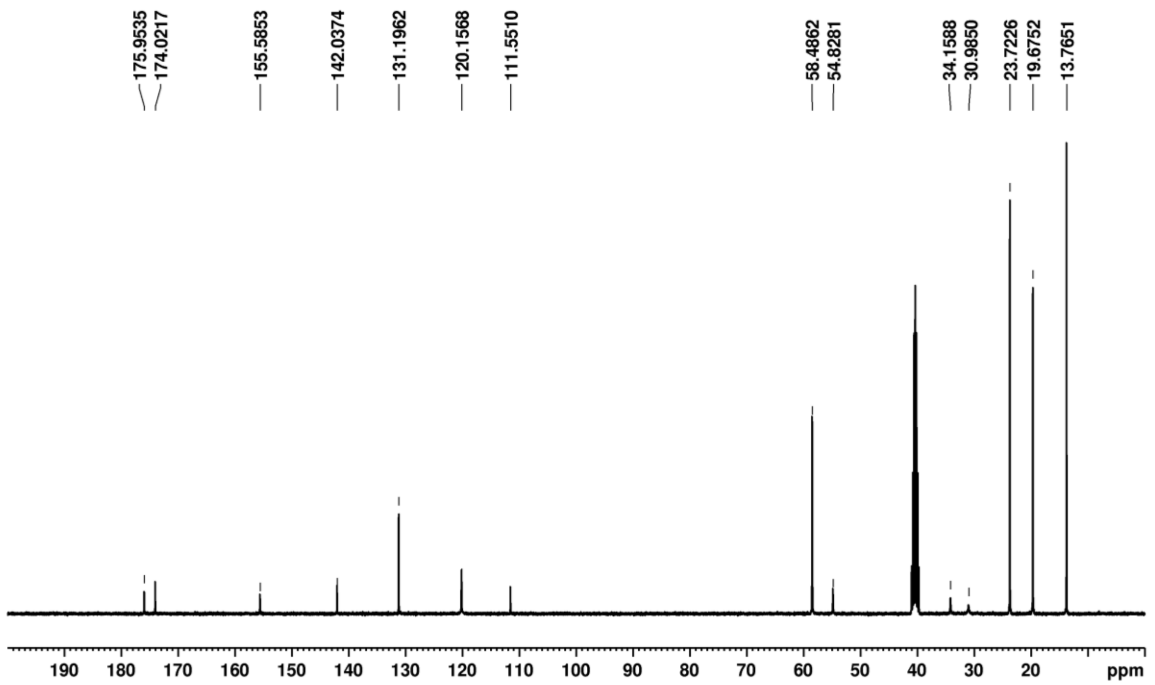

Figure S25 - $^{13}C\ \{^{1}H\}$ NMR spectrum of **10** in DMSO-d_6 conducted at 343 K.

Section 7: Mass Spectrum data

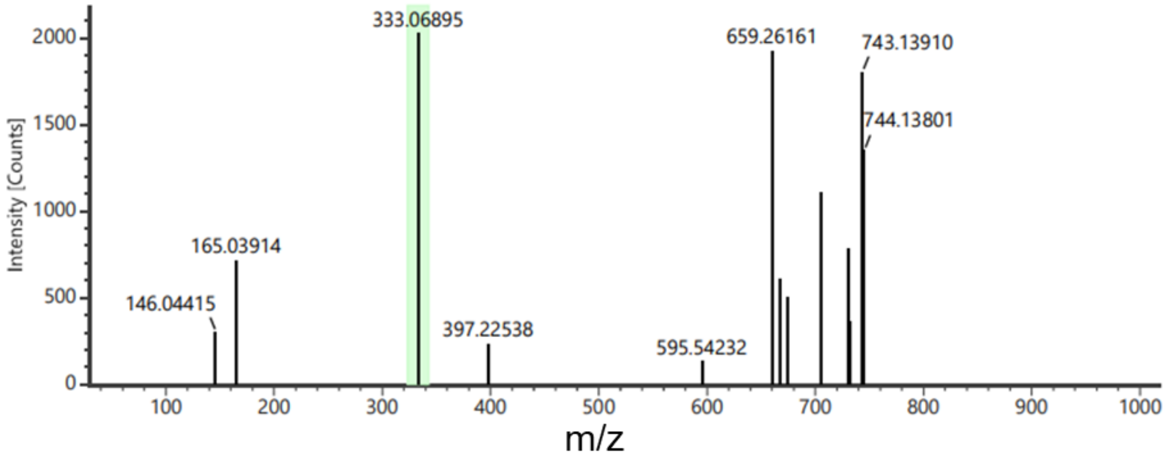

Figure S26 - A high-resolution mass spectrum (ESI$^-$) obtained for **1** in methanol.

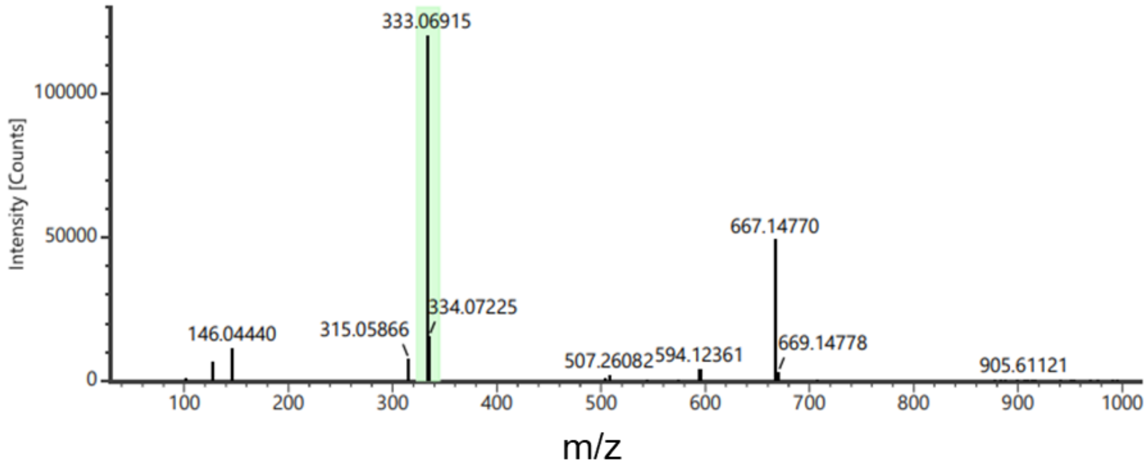

Figure S27 - A high-resolution mass spectrum (ESI⁻) obtained for **2** in methanol.

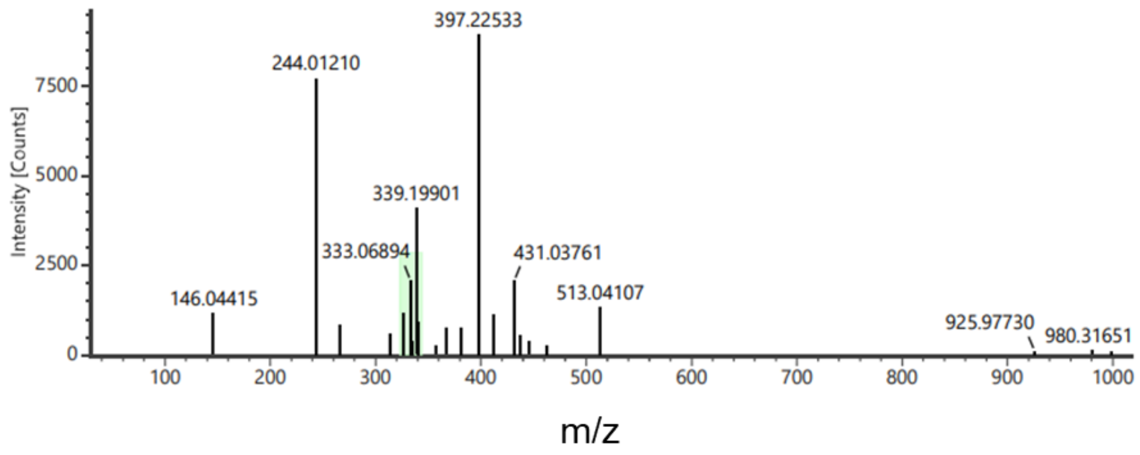

Figure S28 - A high-resolution mass spectrum (ESI⁻) obtained for **3** in methanol.

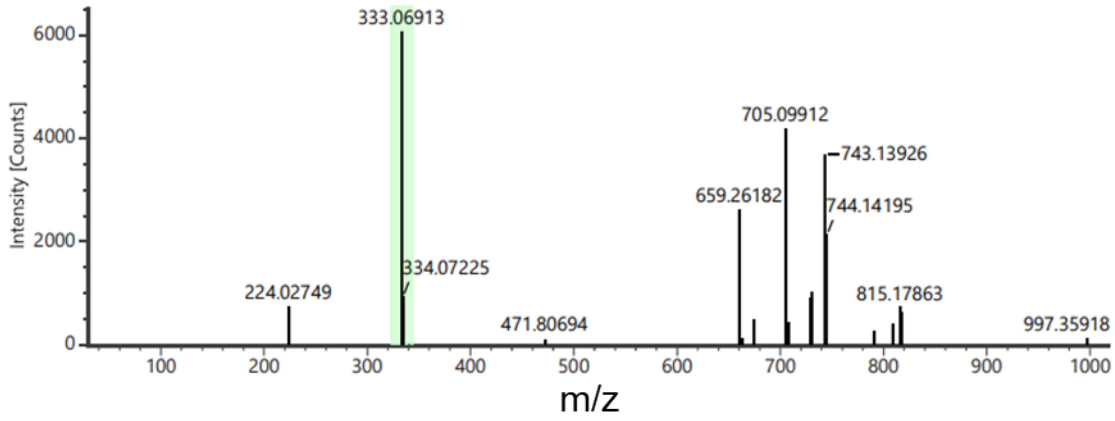

Figure S29 - A high-resolution mass spectrum (ESI⁻) obtained for **4** in methanol.

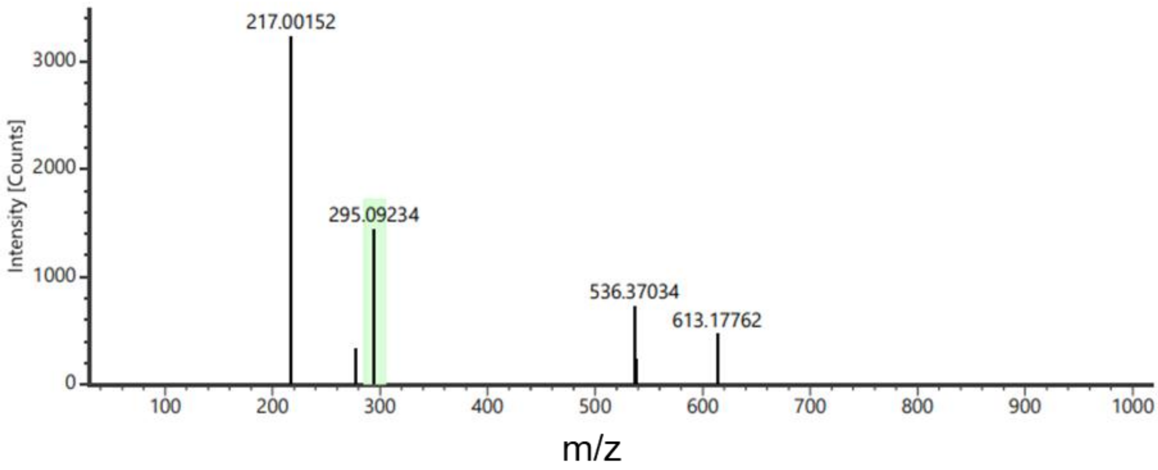

Figure S30 - A high-resolution mass spectrum (ESI$^-$) obtained for **5** in methanol.

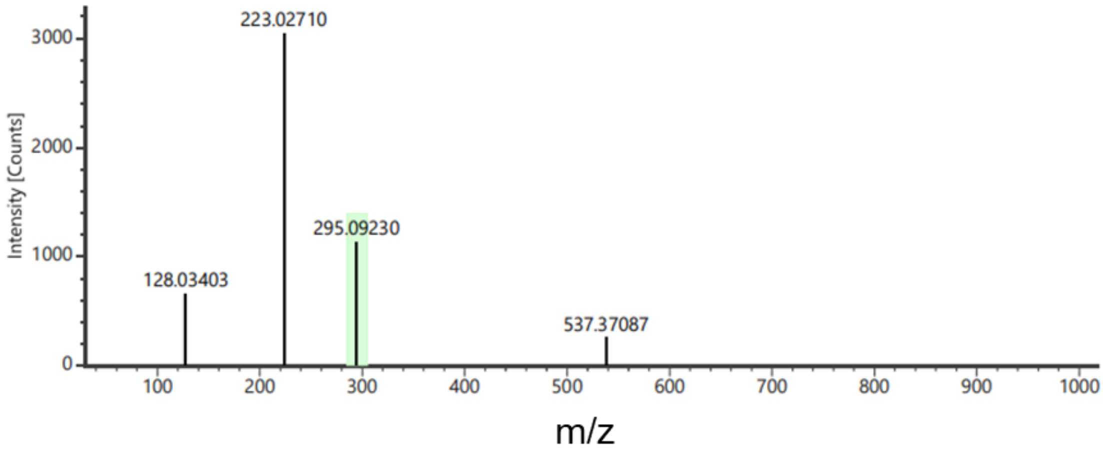

Figure S31 - A high-resolution mass spectrum (ESI$^-$) obtained for **6** in methanol.

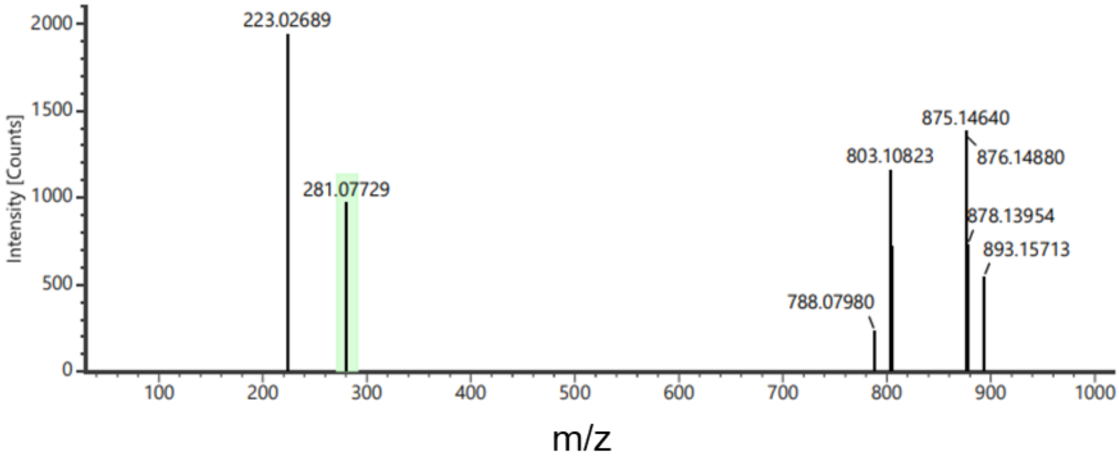

Figure S32 - A high-resolution mass spectrum (ESI$^-$) obtained for **7** in methanol.

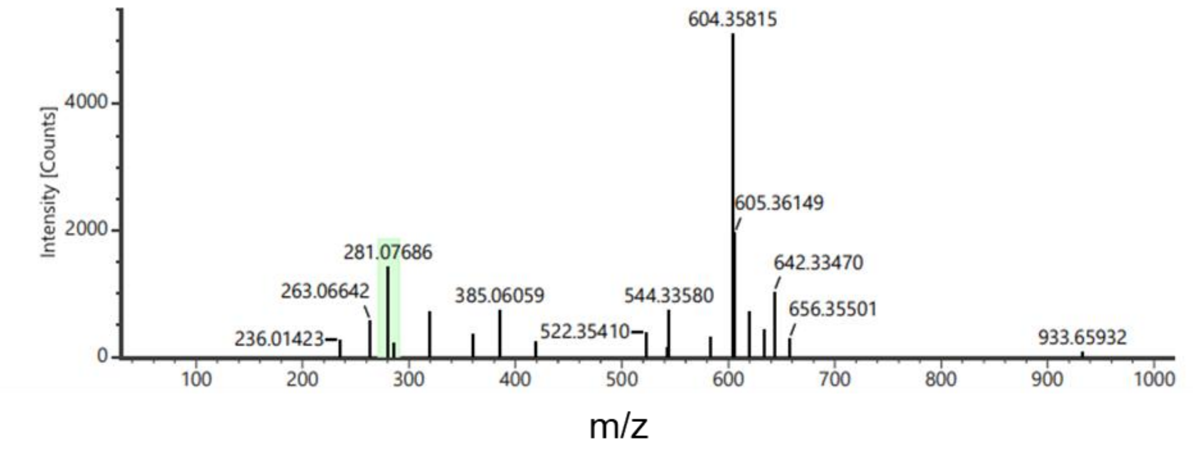

Figure S33 - A high-resolution mass spectrum (ESI^{-}) obtained for **8** in methanol.

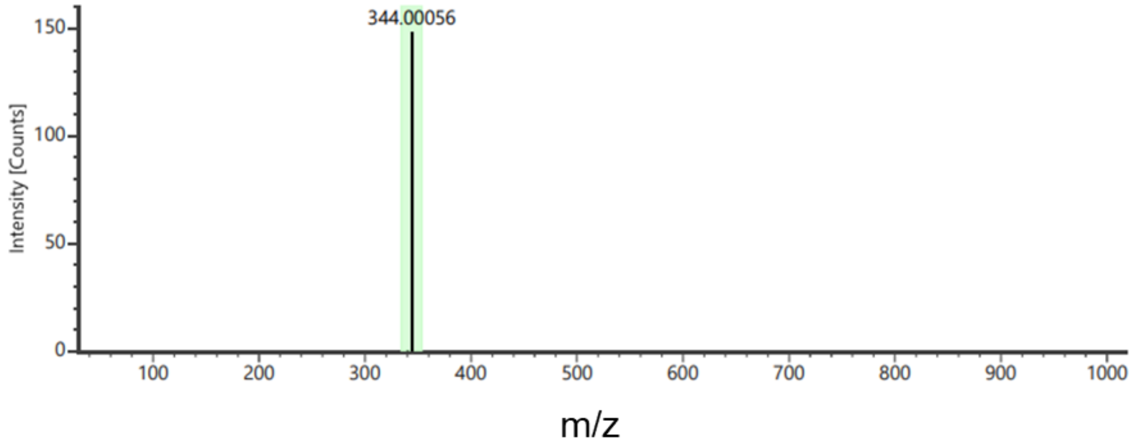

Figure S34 - A high-resolution mass spectrum (ESI^{-}) obtained for **9** in methanol.

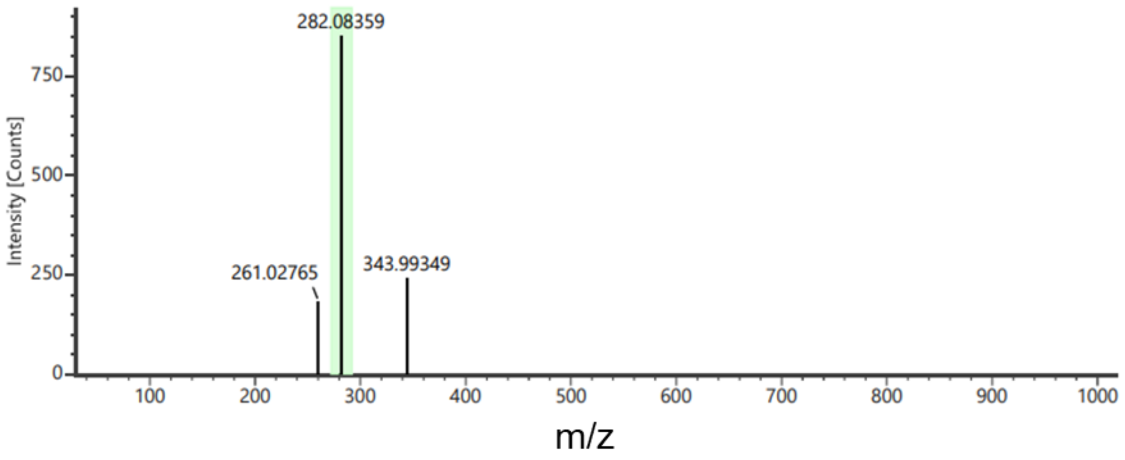

Figure S35 - A high-resolution mass spectrum (ESI^{-}) obtained for **10** in methanol.

Section 8: Quantitative ^{1}H NMR studies

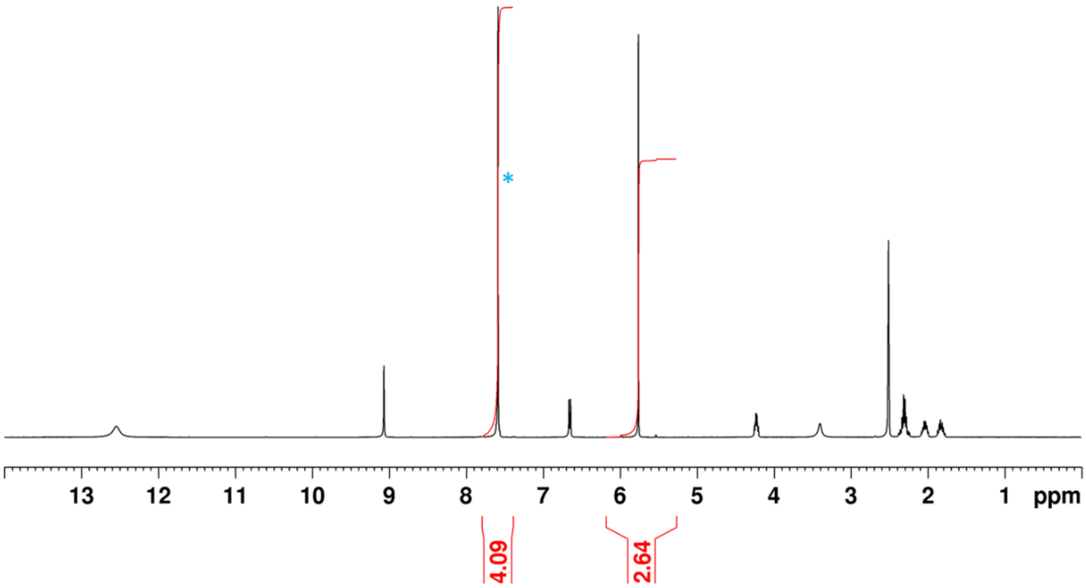

Figure S36 - Quantitative ^{1}H NMR spectrum (d_1 = 60 s) of **1** (56.28 mmol) in DMSO-d_6/ 1.0 % DCM. Comparative integration indicates 0 % of the SSA* has become NMR silent.

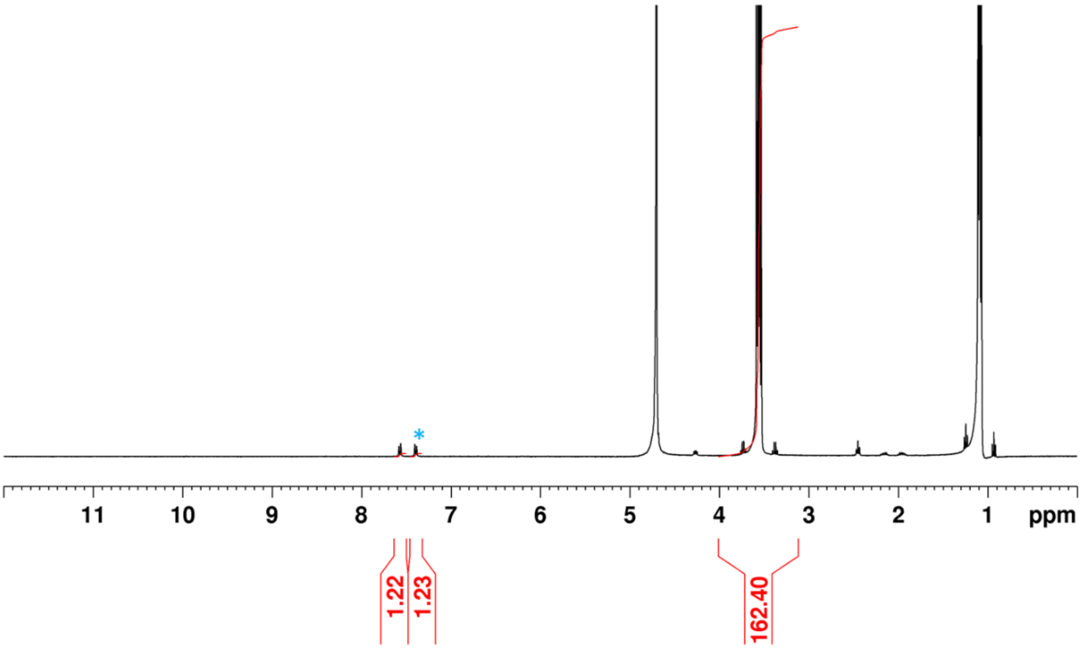

Figure S37 - Quantitative ^{1}H NMR spectrum (d_1 = 60 s) of **1** (5.56 mmol) in D_2O/ 5.0 % EtOH. Comparative integration indicates 38.5 % of the SSA* has become NMR silent.

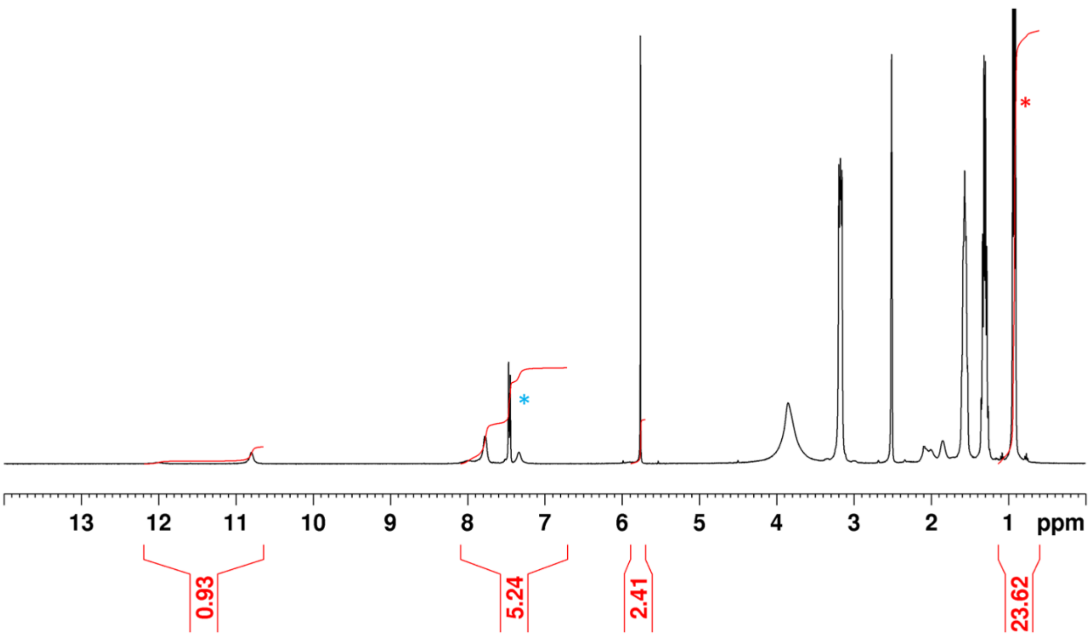

Figure S38 - Quantitative ^{1}H NMR spectrum (d_1 = 60 s) of **2** (56.55 mmol) in DMSO-d_6/ 1.0 % DCM. Comparative integration indicates 0 % of the anionic component of the SSA and 0 % of TBA counter cation has become NMR silent (anion*, TBA*).

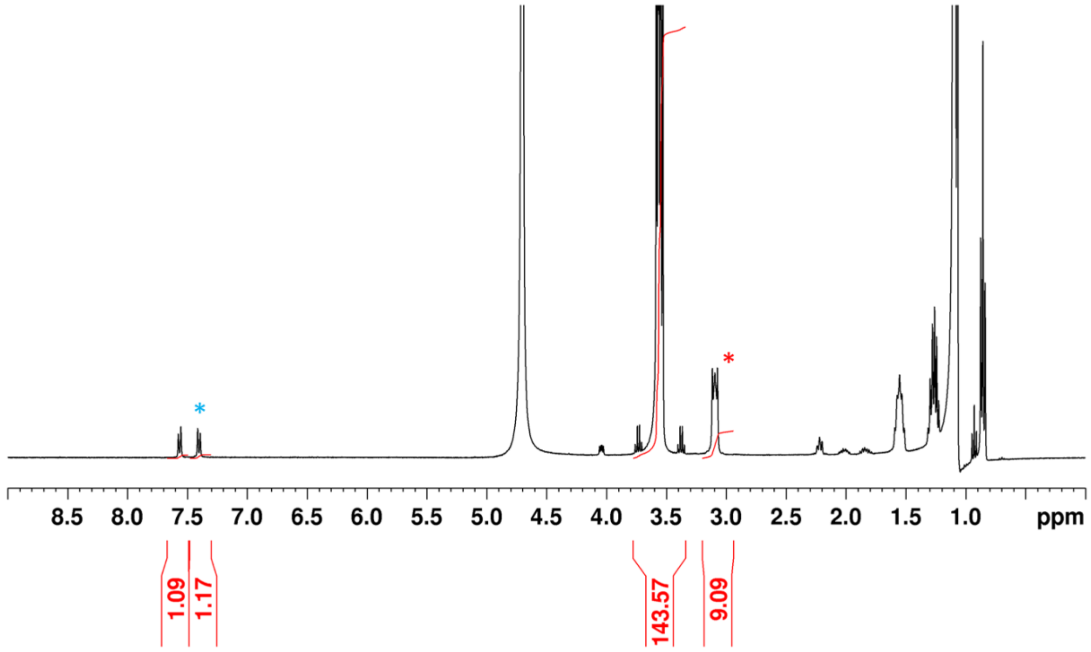

Figure S39 - Quantitative ^{1}H NMR spectrum (d_1 = 60 s) of **2** (6.00 mmol) in D_2O/ 5.0 % EtOH. Comparative integration indicates 41.3 % of the anionic component of the SSA and 43.2 % of TBA counter cation has become NMR silent (anion*, TBA*).

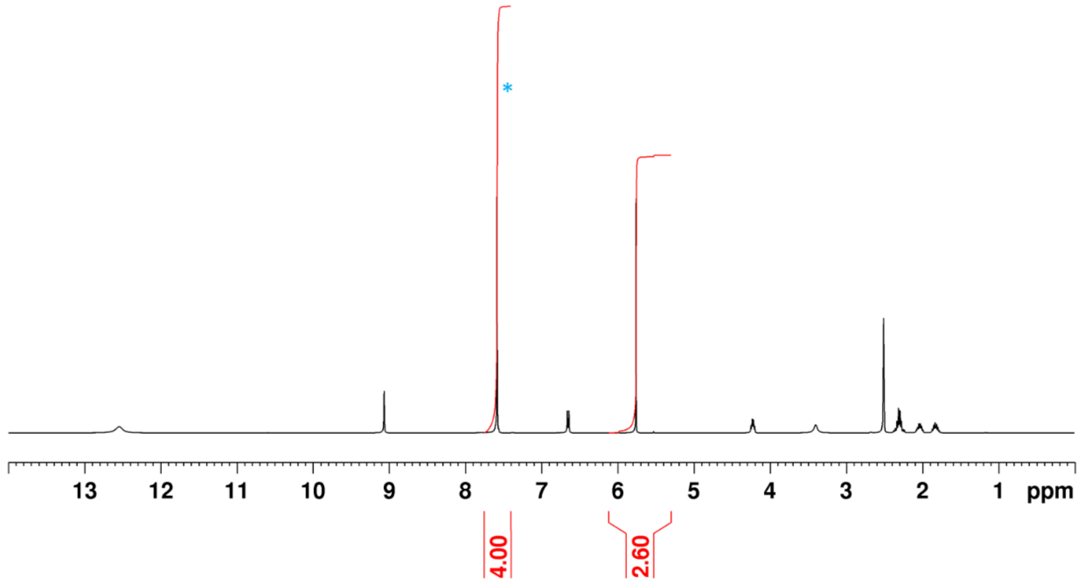

Figure S40 - Quantitative ^{1}H NMR spectrum (d_1 = 60 s) of **3** (54.87 mmol) in DMSO-d_6/ 1.0 % DCM. Comparative integration indicates 0 % of the SSA* has become NMR silent.

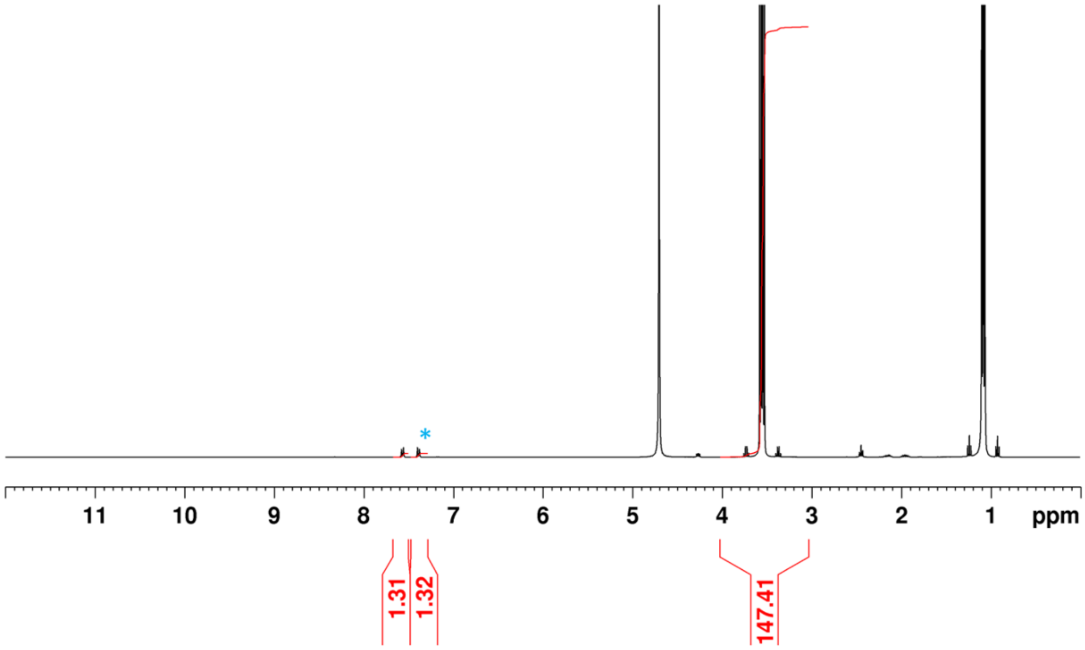

Figure S41 - Quantitative ^{1}H NMR spectrum (d_1 = 60 s) of **3** (5.83 mmol) in D_2O/ 5.0 % EtOH. Comparative integration indicates 34.0 % of the SSA* has become NMR silent.

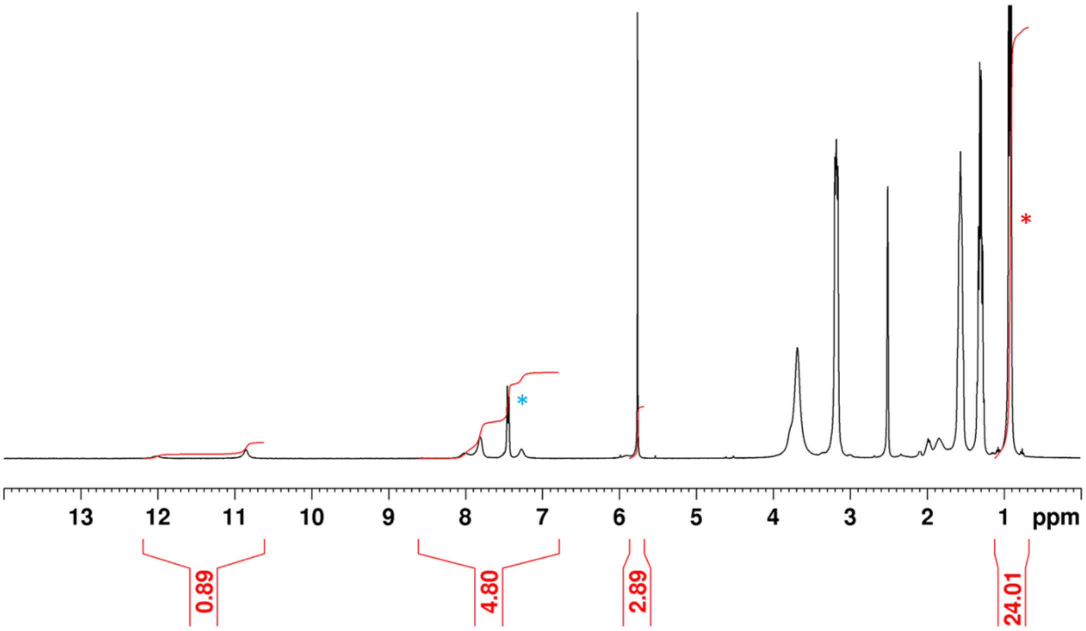

Figure S42 - Quantitative ^{1}H NMR spectrum (d_1 = 60 s) of **4** (55.72 mmol) in DMSO-d_6/ 1.0 % DCM. Comparative integration indicates 0 % of the anionic component of the SSA and 0 % of TBA counter cation has become NMR silent (anion*, TBA*).

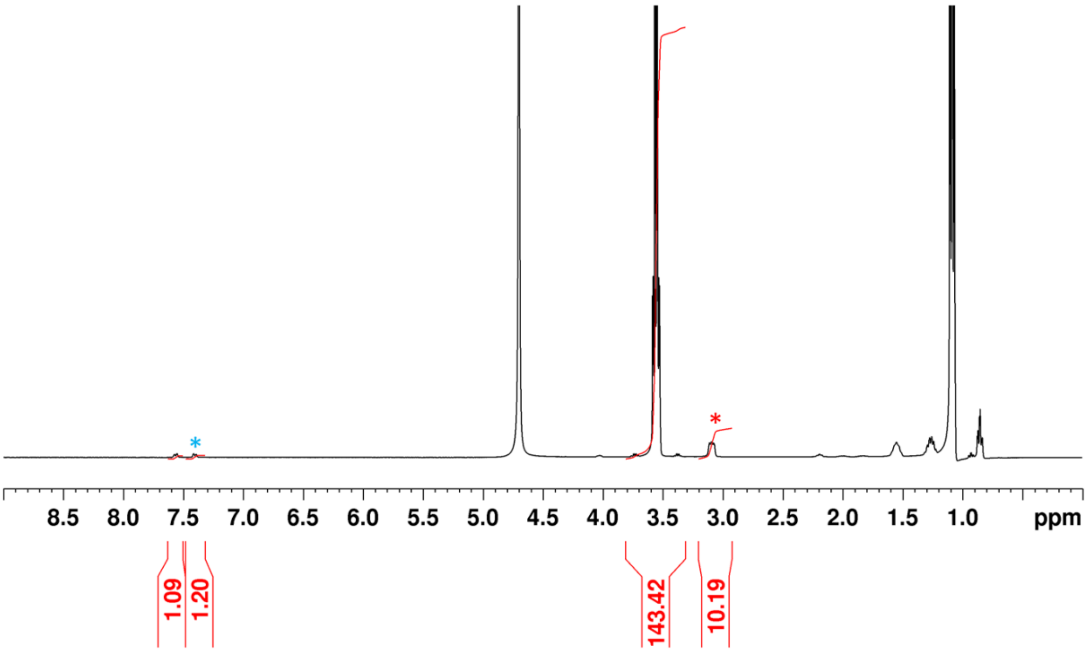

Figure S43 - Quantitative ^{1}H NMR spectrum (d_1 = 60 s) of **4** (5.74 mmol) in D_2O/ 5.0 % EtOH. Comparative integration indicates 39.8 % of the anionic component of the SSA and 36.3 % of TBA counter cation has become NMR silent (anion*, TBA*).

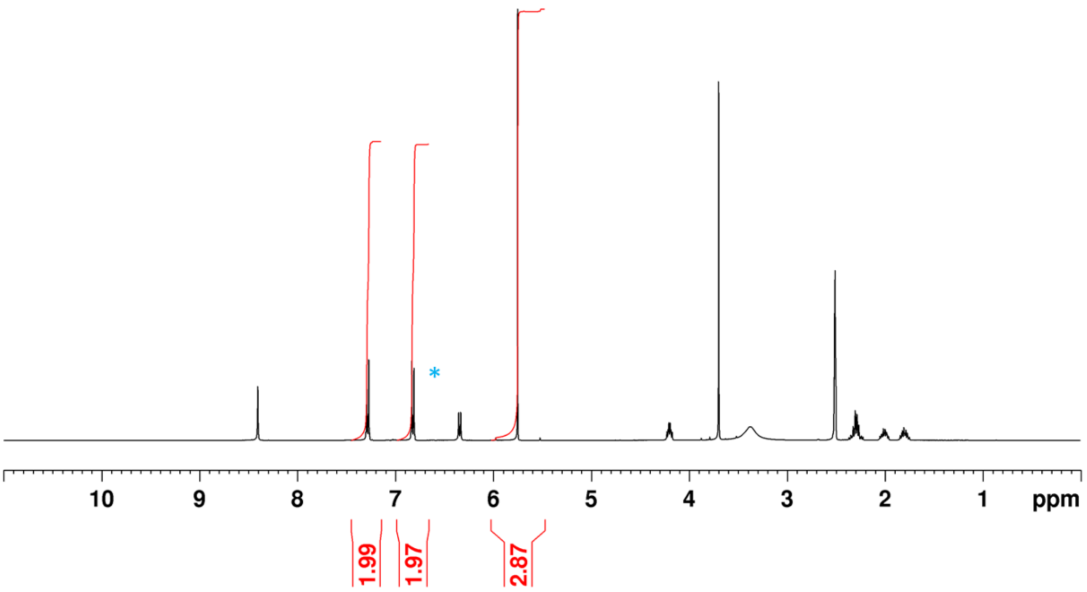

Figure S44 - Quantitative ^{1}H NMR spectrum (d_1 = 60 s) of **5** (55.79 mmol) in DMSO-d_6/ 1.0 % DCM. Comparative integration indicates 0 % of the SSA* has become NMR silent.

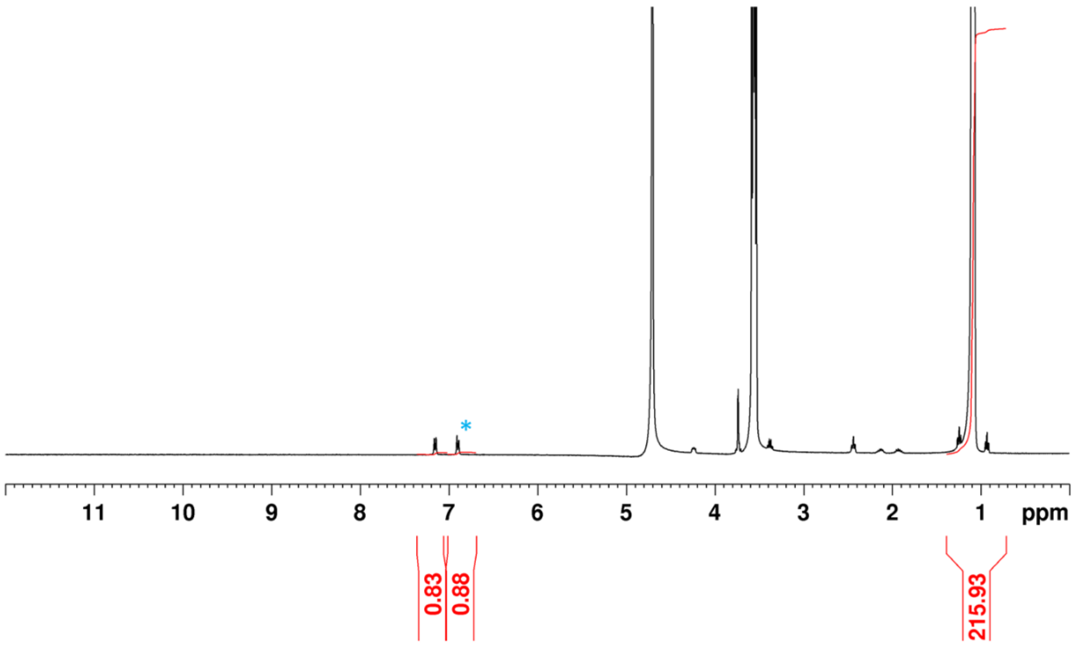

Figure S45 - Quantitative ^{1}H NMR spectrum (d_1 = 60 s) of **5** (5.56 mmol) in D_2O/ 5.0 % EtOH. Comparative integration indicates 56.0 % of the SSA* has become NMR silent.

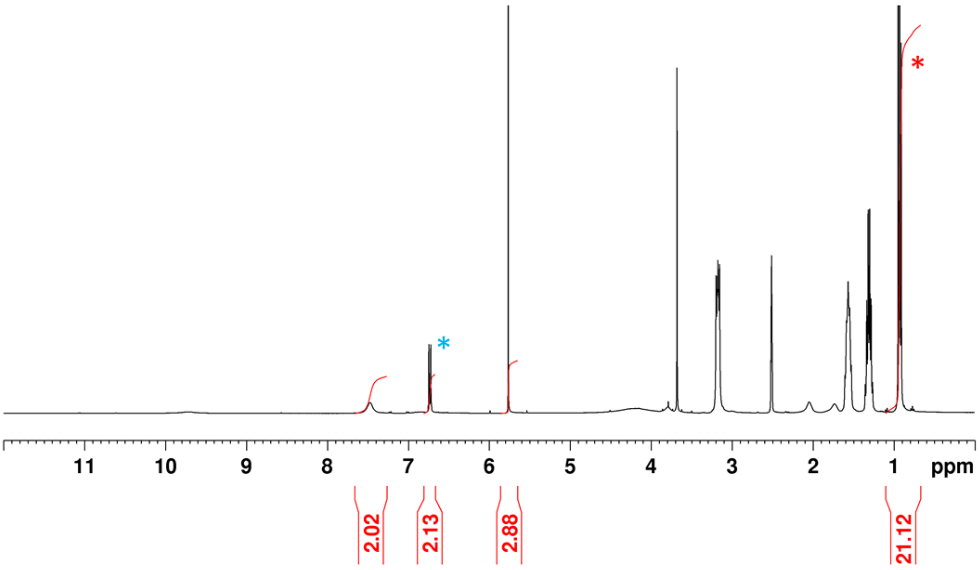

Figure S46 - Quantitative ^{1}H NMR spectrum (d_1 = 60 s) of **6** (56.42 mmol) in DMSO-d_6/ 1.0 % DCM. Comparative integration indicates 0 % of the anionic component of the SSA and 0 % of TBA counter cation has become NMR silent (anion*, TBA*).

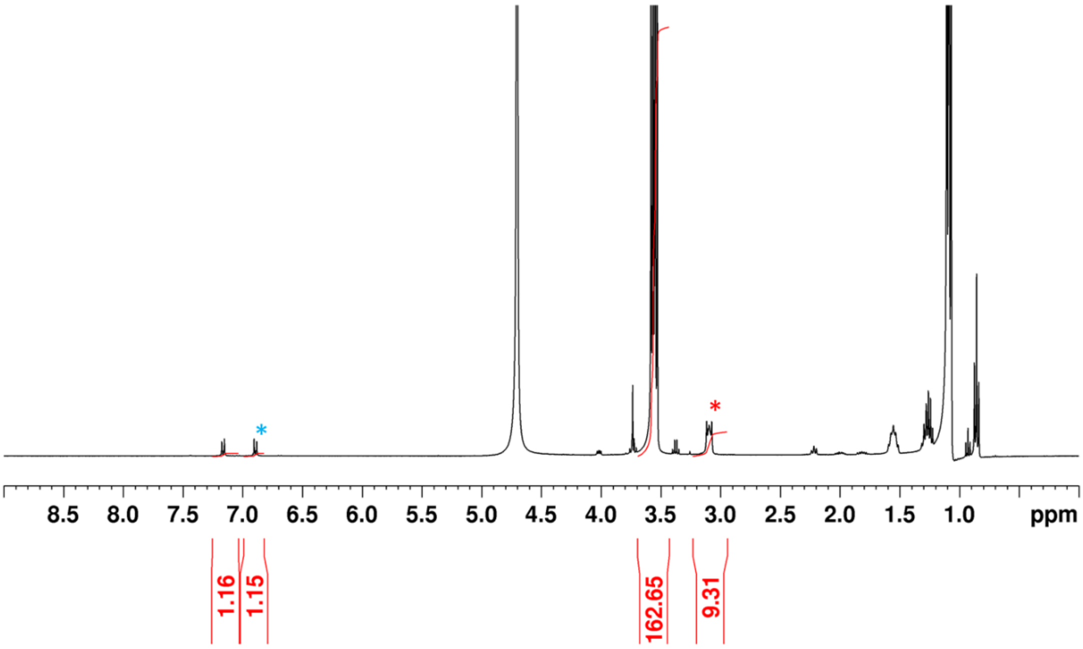

Figure S47 - Quantitative ^{1}H NMR spectrum (d_1 = 60 s) of **6** (5.29 mmol) in D_2O/ 5.0 % EtOH. Comparative integration indicates 42.3 % of the anionic component of the SSA and 41.8 % of TBA counter cation has become NMR silent (anion*, TBA*).

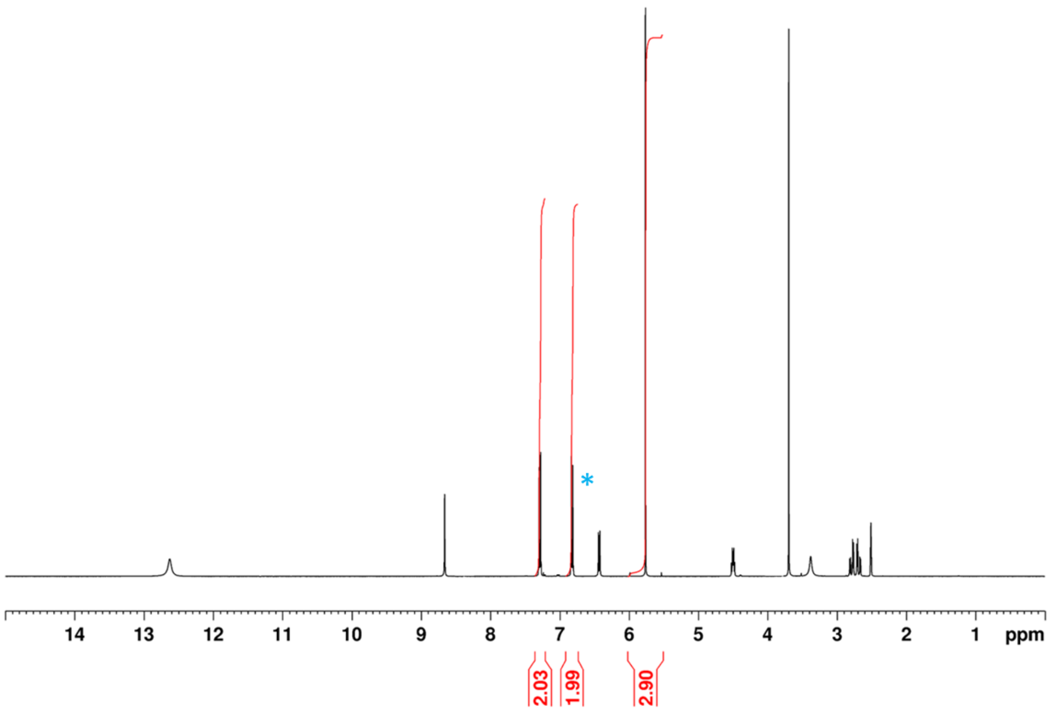

Figure S48 - Quantitative ^{1}H NMR spectrum (d_1 = 60 s) of **7** (55.20 mmol) in DMSO-d_6/ 1.0 % DCM. Comparative integration indicates 0 % of the SSA* has become NMR silent.

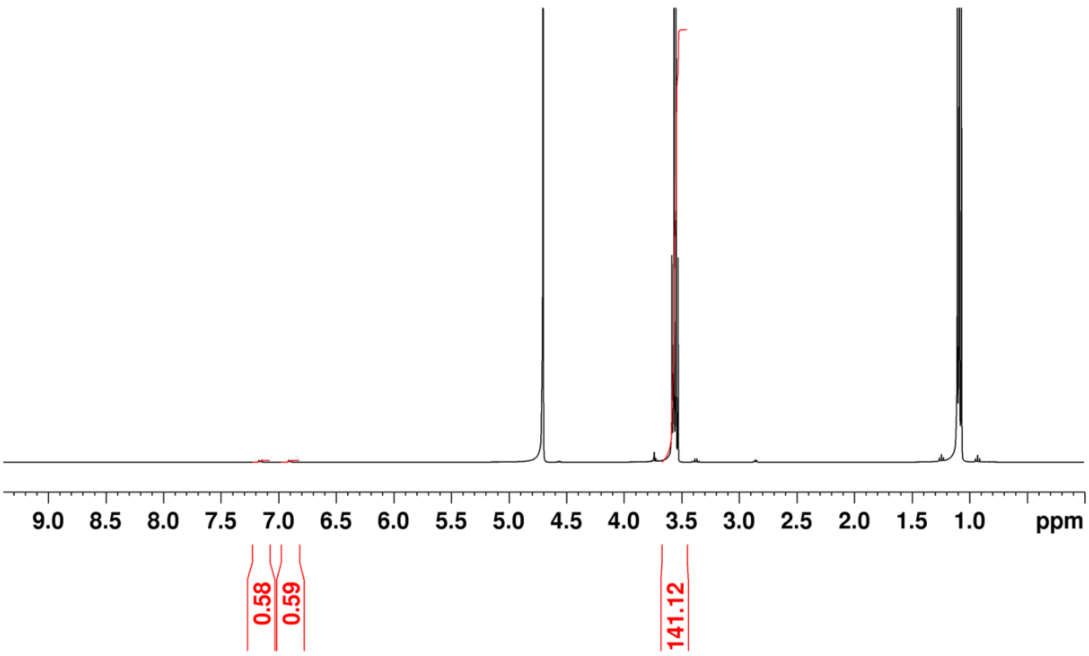

Figure S49 - Quantitative ^{1}H NMR spectrum (d_1 = 60 s) of **7** (6.09 mmol) in D_2O/ 5.0 % EtOH. Comparative integration indicates 70.5 % of the SSA* has become NMR silent.

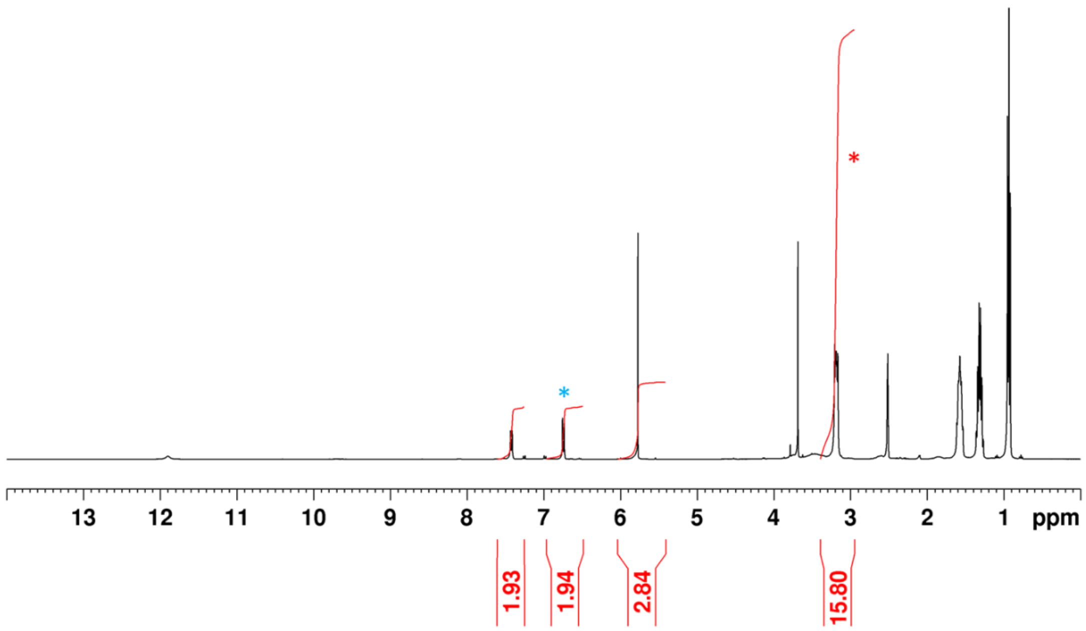

Figure S50 - Quantitative ^{1}H NMR spectrum (d_1 = 60 s) of **8** (56.34 mmol) in DMSO-d_6/ 1.0 % DCM. Comparative integration indicates 0 % of the anionic component of the SSA and 0 % of TBA counter cation has become NMR silent (anion*, TBA*).

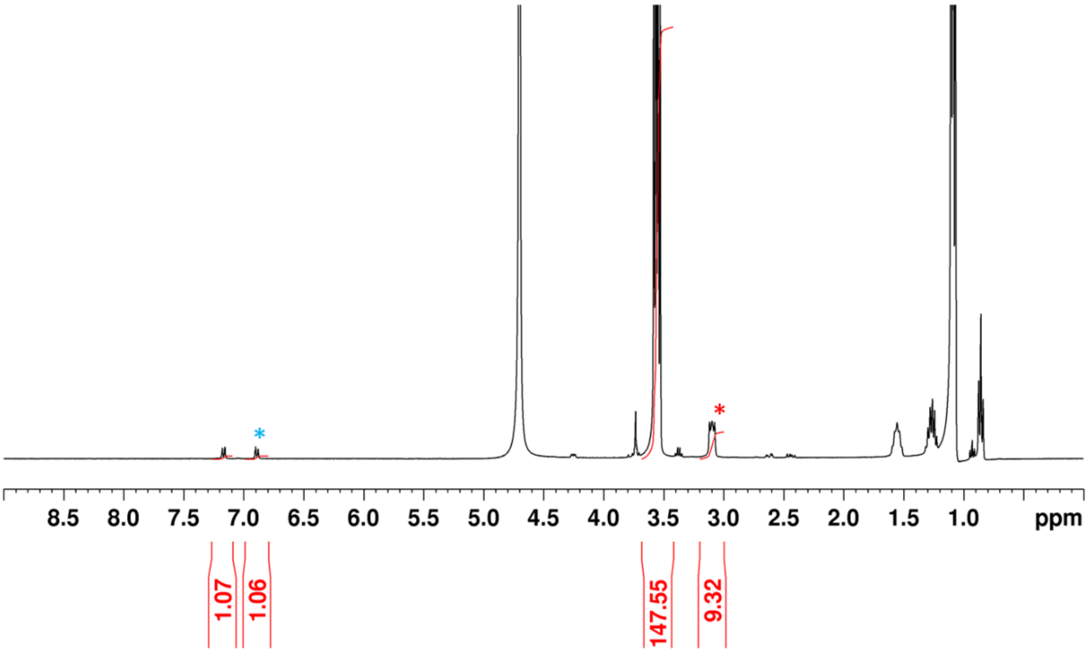

Figure S51 - Quantitative ^{1}H NMR spectrum (d_1 = 60 s) of **8** (5.83 mmol) in D_2O/ 5.0 % EtOH. Comparative integration indicates 46.6 % of the anionic component of the SSA and 41.8 % of TBA counter cation has become NMR silent (anion*, TBA*).

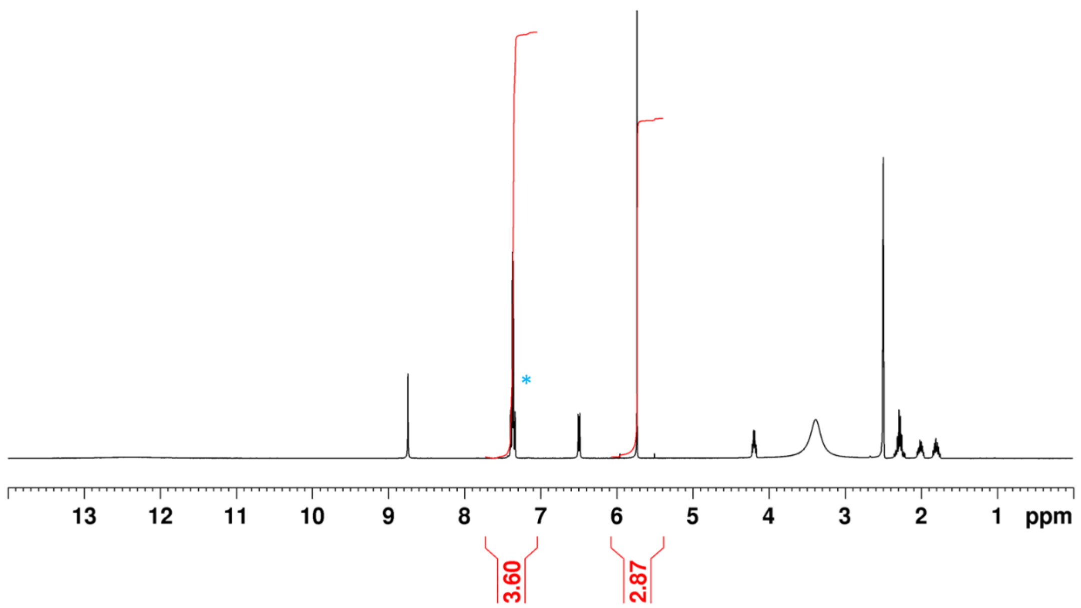

Figure S52 - Quantitative ^{1}H NMR spectrum (d_1 = 60 s) of **9** (55.77 mmol) in DMSO-d_6/ 1.0 % DCM. Comparative integration indicates 0 % of the SSA* has become NMR silent.

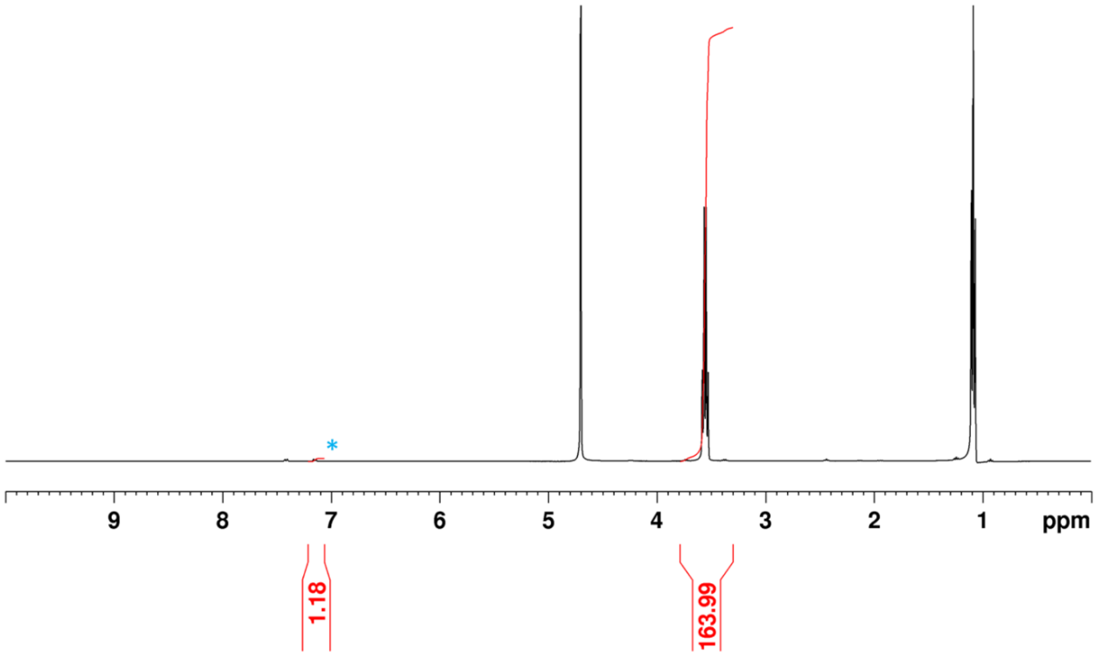

Figure S53 - Quantitative ^{1}H NMR spectrum (d_1 = 60 s) of **9** (5.24 mmol) in D_2O/ 5.0 % EtOH. Comparative integration indicates 40.9 % of the SSA* has become NMR silent.

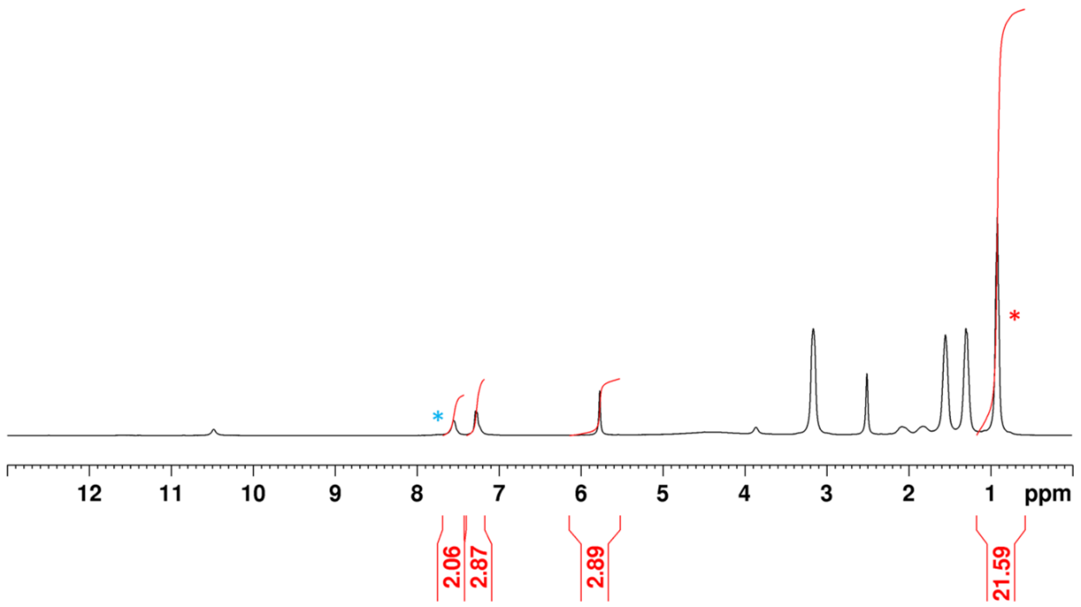

Figure S54 - Quantitative ^{1}H NMR spectrum (d_1 = 60 s) of **10** (55.30 mmol) in DMSO-d_6/ 1.0 % DCM. Comparative integration indicates 0 % of the anionic component of the SSA and 0 % of TBA counter cation has become NMR silent (anion*, TBA*).

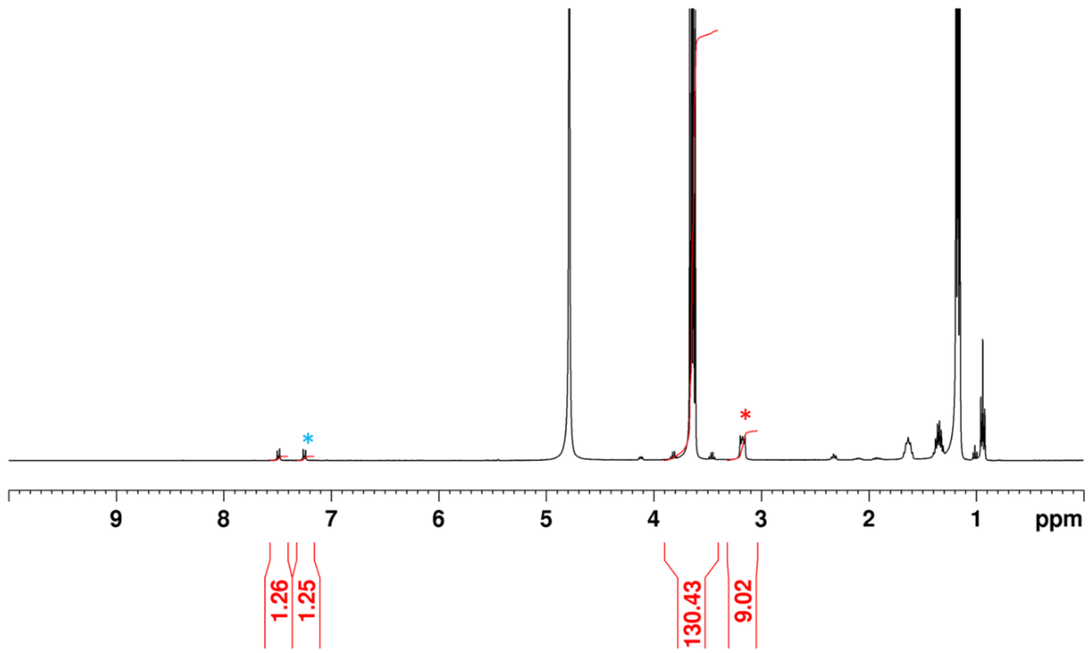

Figure S55 - Quantitative ^{1}H NMR spectrum (d_1 = 60 s) of **10** (6.59 mmol) in D_2O/ 5.0 % EtOH. Comparative integration indicates 37.5 % of the anionic component of the SSA and 43.6 % of TBA counter cation has become NMR silent (anion*, TBA*).

Summary

Table S1 – Overview of results of the quantitative (Q) ^{1}H NMR studies for **1**, **3**, **5**, **7** and **9** performed at concentrations of ~ 112 mM in DMSO-d_6/ 1.0 % DCM and ~ 5.56 mM in D_2O/ 5.0 % EtOH. Values given in % represent the observed proportion of the SSA that has become NMR silent.

SSA	D_2O 5 % EtOH (%)	DMSO-d_6 1 % DCM (%)
**1**	38.5	0
**3**	34.0	0
**5**	56.0	0
**7**	70.5	0
**9**	40.9	0

Table S2 – Overview of results of the quantitative (Q) ^{1}H NMR studies for **2**, **4**, **6**, **8** and **10** performed at concentrations of ~ 112 mM in DMSO-d_6/ 1.0 % DCM and ~ 5.56 mM in D_2O/ 5.0 % EtOH. Values given in % represent the observed proportion of anion and cation that has become NMR silent.

SSA		D_2O 5 % EtOH (%)	DMSO-d_6 1 % DCM (%)
**2**	Anion	39.8	0
	Cation	36.3	0
**4**	Anion	41.3	0
	Cation	43.2	0
**6**	Anion	42.3	0
	Cation	41.8	0
**8**	Anion	46.6	0
	Cation	41.8	0
**10**	Anion	37.5	0
	Cation	43.6	0

Section 9: ^{1}H NMR DOSY studies

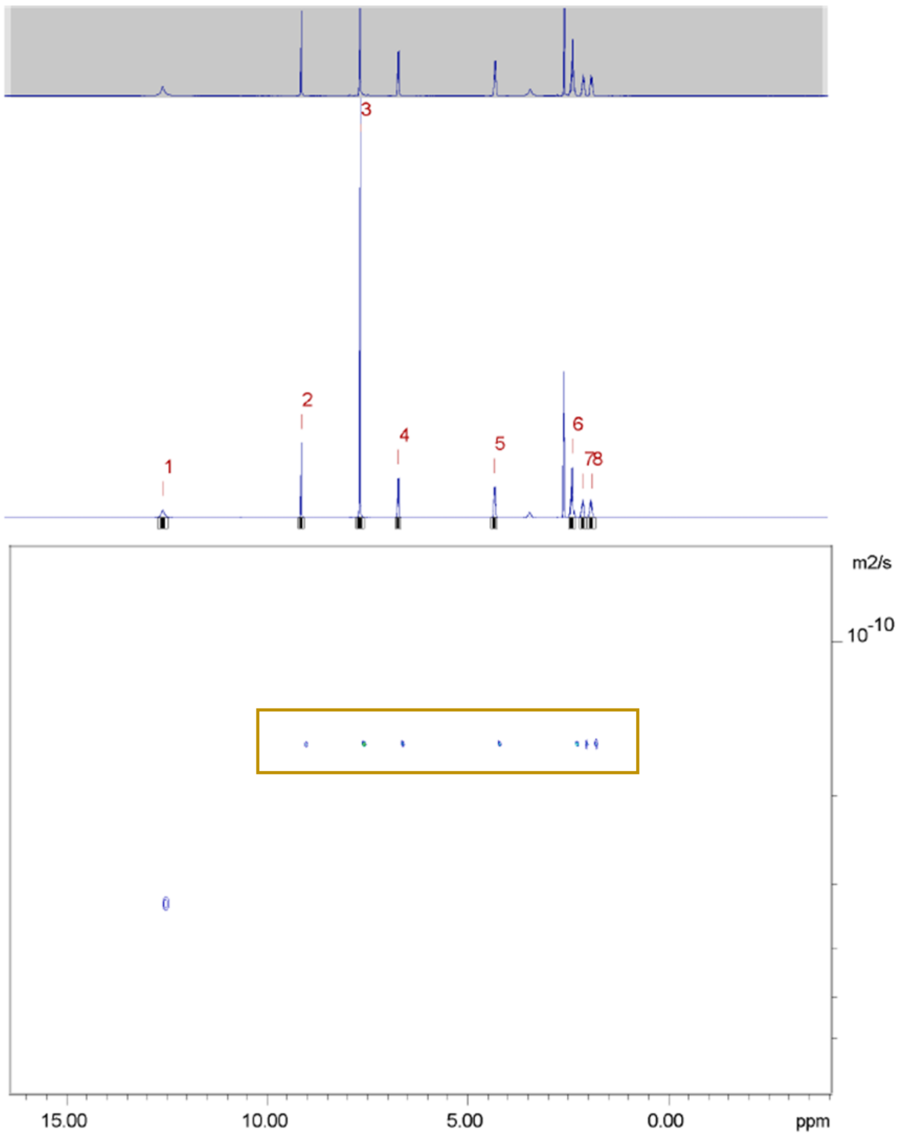

Peak name	F2 [ppm]	Io	error	D [m2/s]	error	fitInfo
1	12.497	2.23e+09	3.352e+05	3.27e-10	1.039e-13	Done
2	9.052	2.71e+09	1.997e+05	1.58e-10	2.584e-14	Done
3	7.584	1.32e+10	2.331e+05	1.58e-10	6.203e-15	Done
4	6.632	2.72e+09	2.000e+05	1.59e-10	2.581e-14	Done
5	4.245	3.15e+09	2.087e+05	1.58e-10	2.319e-14	Done
6	2.308	5.54e+09	2.176e+05	1.60e-10	1.387e-14	Done
7	2.030	2.59e+09	2.173e+05	1.59e-10	2.939e-14	Done
8	1.825	2.55e+09	2.409e+05	1.59e-10	3.308e-14	Done

Figure S56 - ^{1}H DOSY NMR of **1** (112.6 mM) in a DMSO-d_6/0.5 % H_2O solution conducted at 298 K and a table reporting the diffusion constants calculated for each peak used to determine the hydrodynamic diameter (d_H) of **1** (d_H = 1.38 nm).

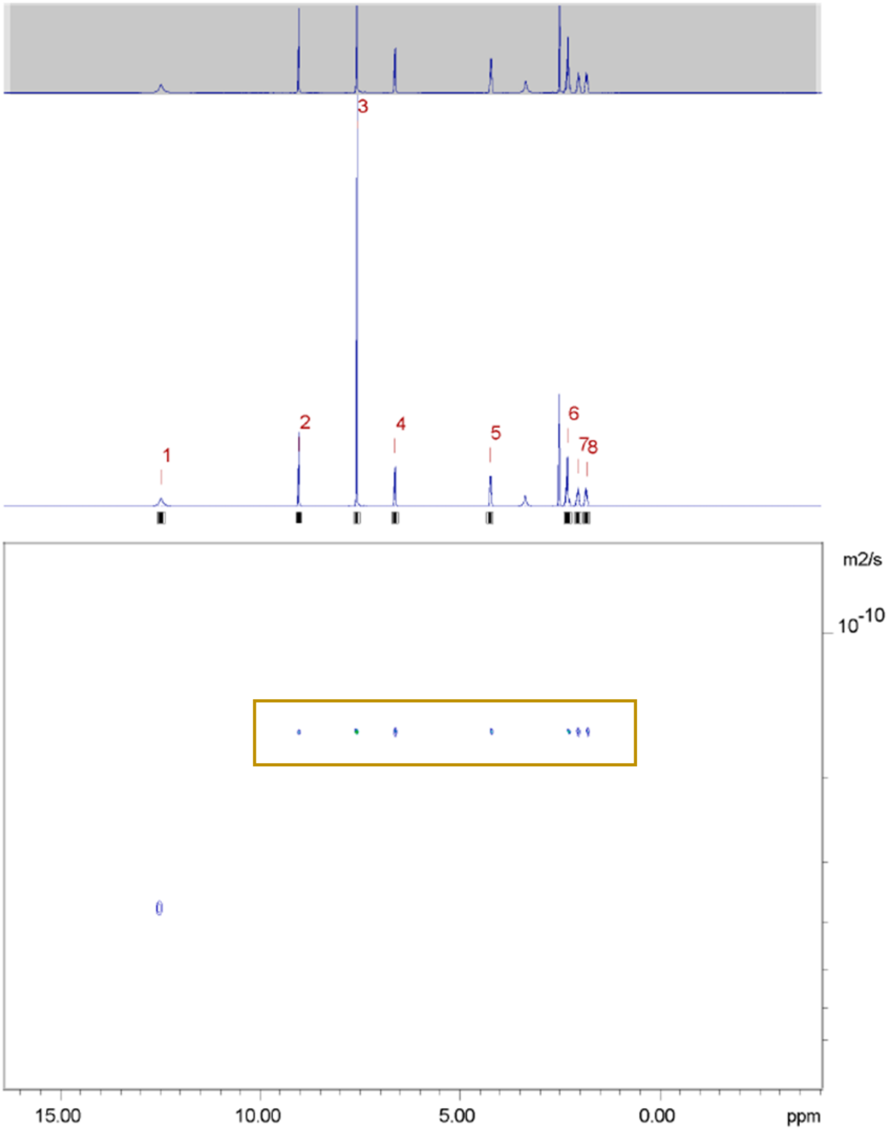

Peak name	F2 [ppm]	D [m2/s]	error	fitInfo
1	12.497	3.74e-10	1.267e-13	Done
2	9.032	1.60e-10	2.636e-14	Done
3	7.584	1.60e-10	5.756e-15	Done
4	6.632	1.60e-10	2.978e-14	Done
5	4.245	1.60e-10	2.474e-14	Done
6	2.294	1.61e-10	1.591e-14	Done
7	2.037	1.60e-10	3.272e-14	Done
8	1.818	1.60e-10	3.230e-14	Done

Figure S57 - ^{1}H DOSY NMR of **3** (109.7 mM) in a DMSO-d_6/0.5 % H_2O solution conducted at 298 K and a table reporting the diffusion constants calculated for each peak used to determine the hydrodynamic diameter (d_H) of **3** (d_H = 1.37 nm).

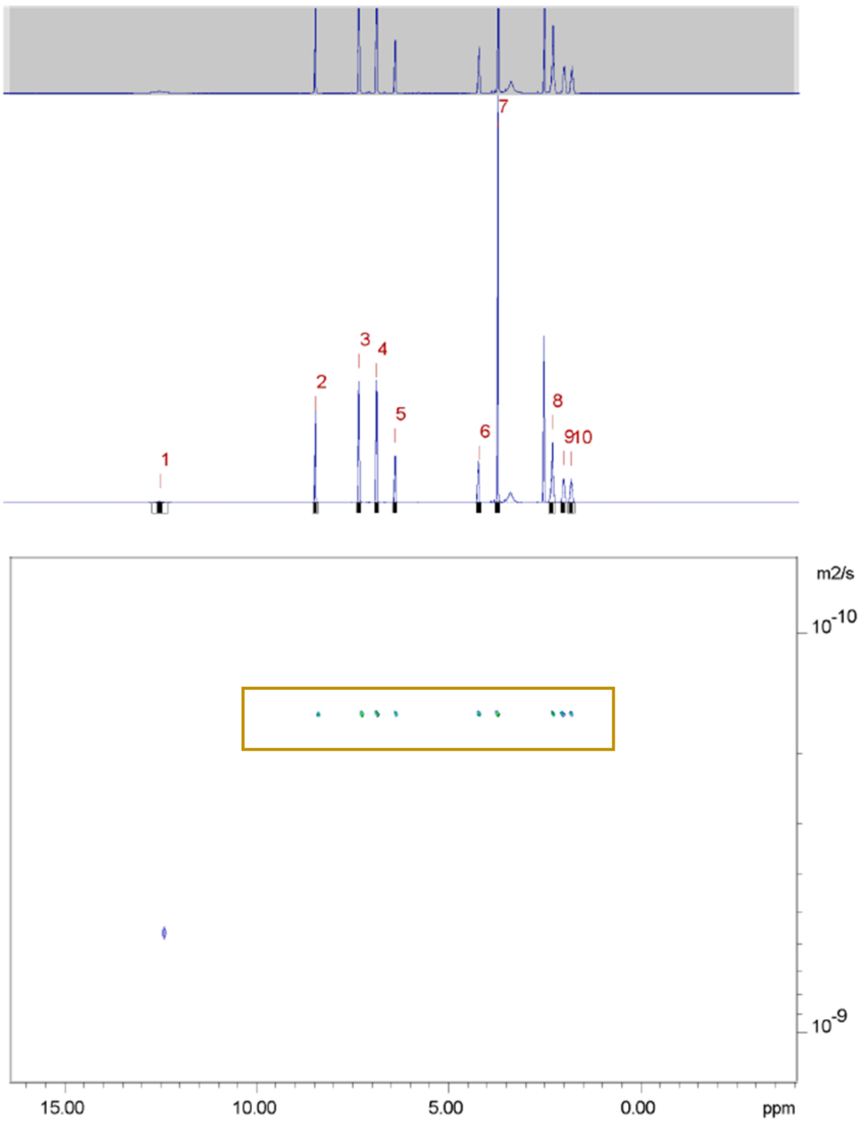

Peak name	F2 [ppm]	Io	error	D [m2/s]	error	fitInfo
1	12.411	1.27e+09	9.970e+05	5.64e-10	9.289e-13	Done
2	8.404	4.81e+09	3.992e+05	1.59e-10	2.947e-14	Done
3	7.280	1.11e+10	3.610e+05	1.59e-10	1.154e-14	Done
4	6.824	1.10e+10	3.409e+05	1.59e-10	1.099e-14	Done
5	6.347	4.60e+09	3.413e+05	1.60e-10	2.639e-14	Done
6	4.185	5.45e+09	3.617e+05	1.60e-10	2.364e-14	Done
7	3.709	1.70e+10	3.618e+05	1.60e-10	7.584e-15	Done
8	2.308	9.40e+09	4.348e+05	1.60e-10	1.651e-14	Done
9	2.017	4.29e+09	3.617e+05	1.60e-10	3.002e-14	Done
10	1.805	4.30e+09	4.343e+05	1.59e-10	3.588e-14	Done

Figure S58 - ^{1}H DOSY NMR of **5** (111.6 mM) in a DMSO-d_6/0.5 % H_2O solution conducted at 298 K and a table reporting the diffusion constants calculated for each peak used to determine the hydrodynamic diameter (d_H) of **5** (d_H = 1.37 nm).

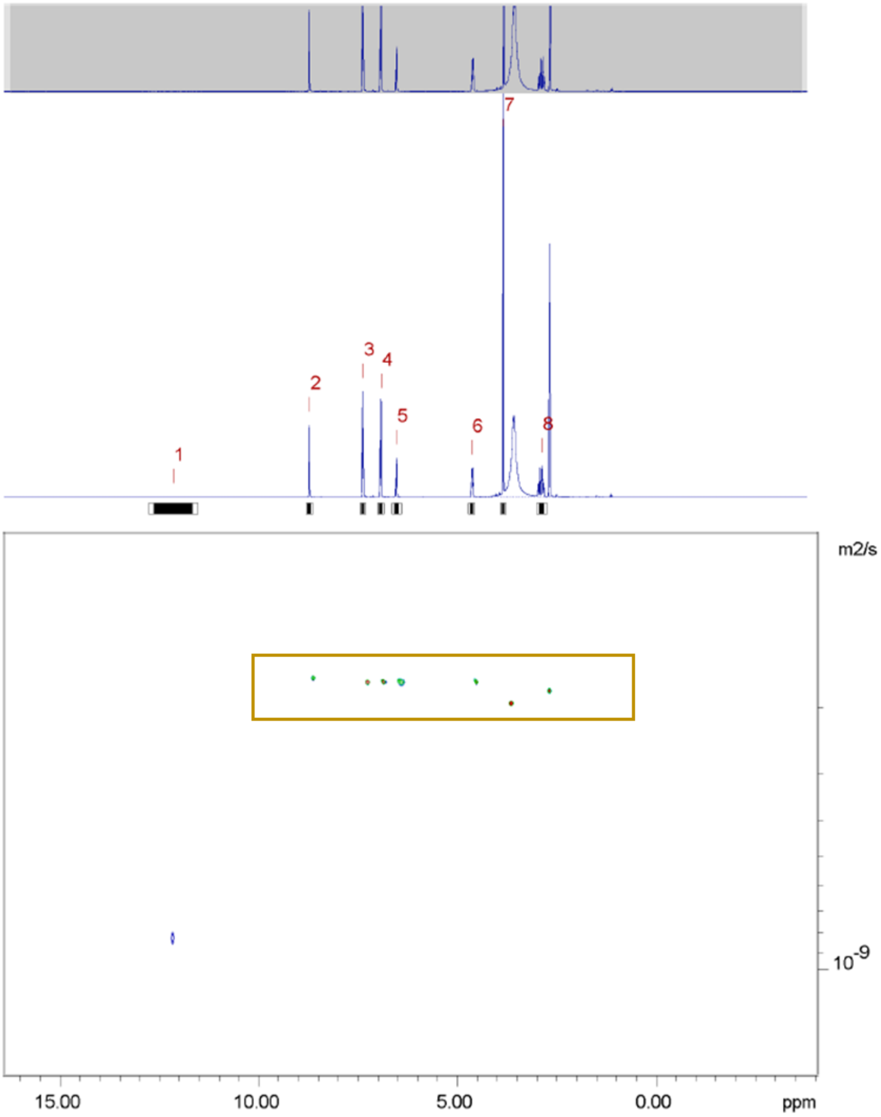

Peak name	F2 [ppm]	Io	error	D [m2/s]	error	fitInfo
1	12.118	3.11e+08	3.971e+06	8.14e-10	2.084e-11	Done
2	8.648	3.69e+09	8.831e+05	1.69e-10	9.009e-14	Done
3	7.275	8.68e+09	8.491e+05	1.70e-10	3.695e-14	Done
4	6.819	8.64e+09	8.486e+05	1.70e-10	3.704e-14	Done
5	6.417	3.82e+09	1.040e+06	1.70e-10	1.027e-13	Done
6	4.501	4.34e+09	8.539e+05	1.73e-10	7.562e-14	Done
7	3.691	1.54e+10	8.463e+05	1.95e-10	2.359e-14	Done
8	2.706	7.68e+09	1.118e+06	1.82e-10	5.861e-14	Done

Figure S59 - ^{1}H DOSY NMR of **7** (110.4 mM) in a DMSO-d_6/0.5 % H_2O solution conducted at 298 K and a table reporting the diffusion constants calculated for each peak used to determine the hydrodynamic diameter (d_H) of **7** (d_H = 1.27 nm).

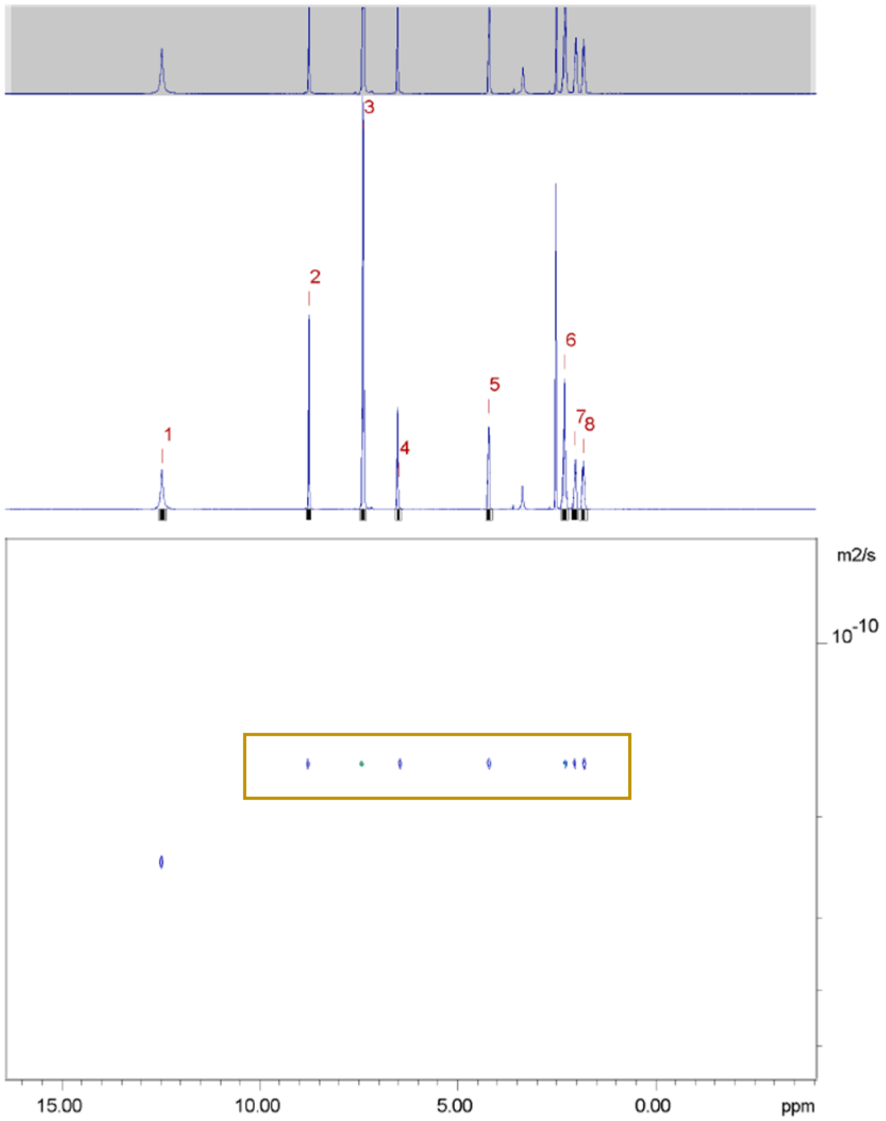

Peak name	F2 [ppm]	Io	error	D [m2/s]	error	fitInfo
1	12.457	9.50e+09	1.082e+06	2.42e-10	5.958e-14	Done
2	8.748	9.34e+09	7.235e+05	1.62e-10	2.781e-14	Done
3	7.386	4.43e+10	8.688e+05	1.62e-10	7.041e-15	Done
4	6.487	9.26e+09	7.981e+05	1.61e-10	3.084e-14	Done
5	4.205	1.08e+10	8.343e+05	1.61e-10	2.769e-14	Done
6	2.288	1.89e+10	9.035e+05	1.63e-10	1.729e-14	Done
7	2.030	8.70e+09	7.988e+05	1.62e-10	3.295e-14	Done
8	1.812	8.65e+09	9.633e+05	1.61e-10	3.991e-14	Done

Figure S60 - ^{1}H DOSY NMR of **9** (109.7 mM) in a DMSO-d_6/0.5 % H_2O solution conducted at 298 K and a table reporting the diffusion constants calculated for each peak used to determine the hydrodynamic diameter (d_H) of **9** (d_H = 1.35 nm).

Summary

Table S3 - Overview of diffusion coefficients (m^2s^{-1}) and hydrodynamic diameters (nm) of **1**, **3**, **5**, **7** and **9** in DMSO-d_6/0.5 % H_2O solution at 298 K. Errors for diffusion constants are no greater than ± 1 x 10^{-13} m^2s^{-1}.

SSA	Diffusion coefficients (m^2s^{-1})	Hydrodynamic diameter (nm)
**1**	1.59 x 10^{-10}	1.38
**3**	1.60 x 10^{-10}	1.37
**5**	1.60 x 10^{-10}	1.37
**7**	1.76 x 10^{-10}	1.27
**9**	1.62 x 10^{-10}	1.35

Section 10: Dynamic Light Scattering (DLS) studies

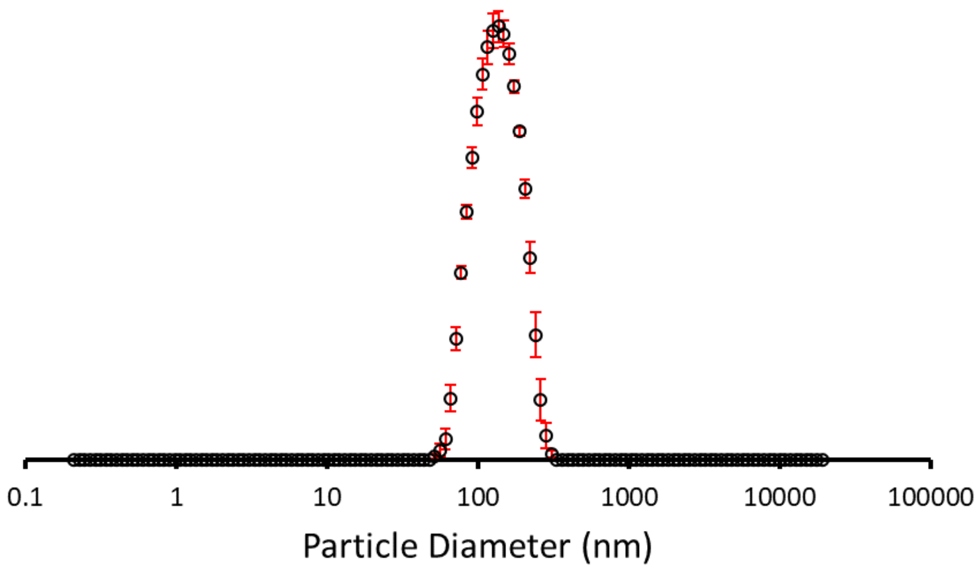

Figure S61 – The average intensity particle size distribution calculated using 10 DLS runs of **2** (5.56 mM) in H_2O/5.0 % EtOH at 298 K, with a peak maxima of 140 nm.

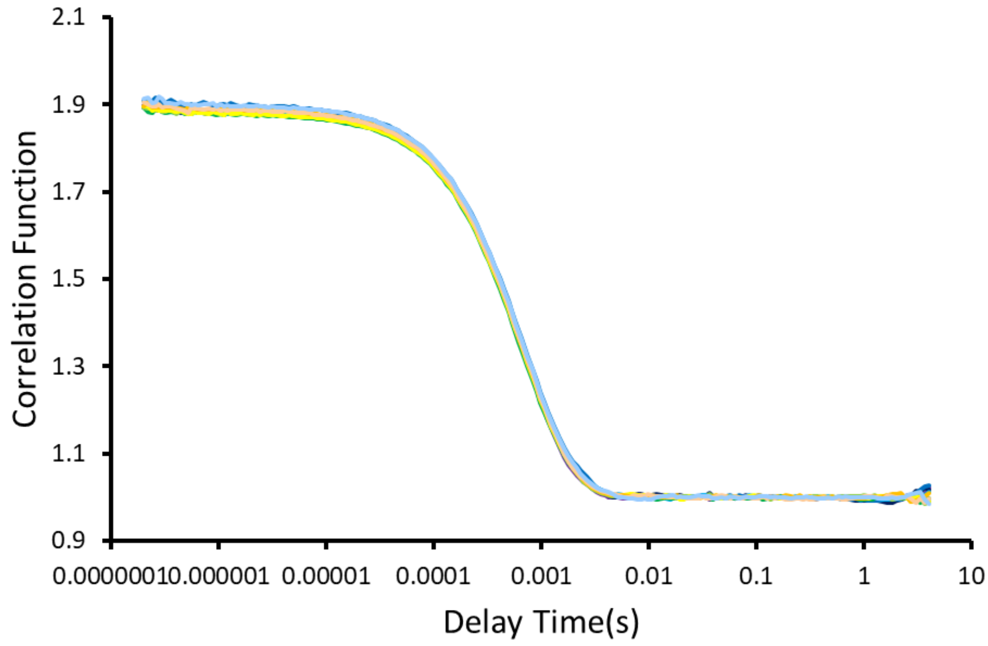

Figure S62 – The correlation function data for 10 DLS runs of **2** (5.56 mM) in H_2O/5.0 % EtOH at 298 K.

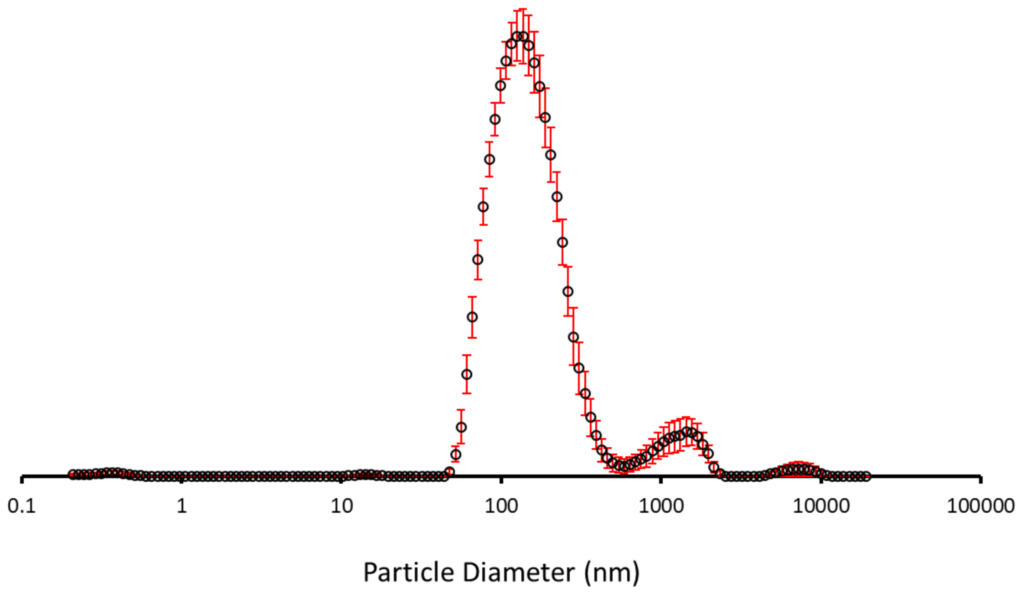

Figure S63 – The average intensity particle size distribution calculated using 10 DLS runs of **3** (5.56 mM) in H_2O/5.0 % EtOH at 298 K, with a peak 1 maxima of 154 nm and a peak 2 maxima of 1410 nm.

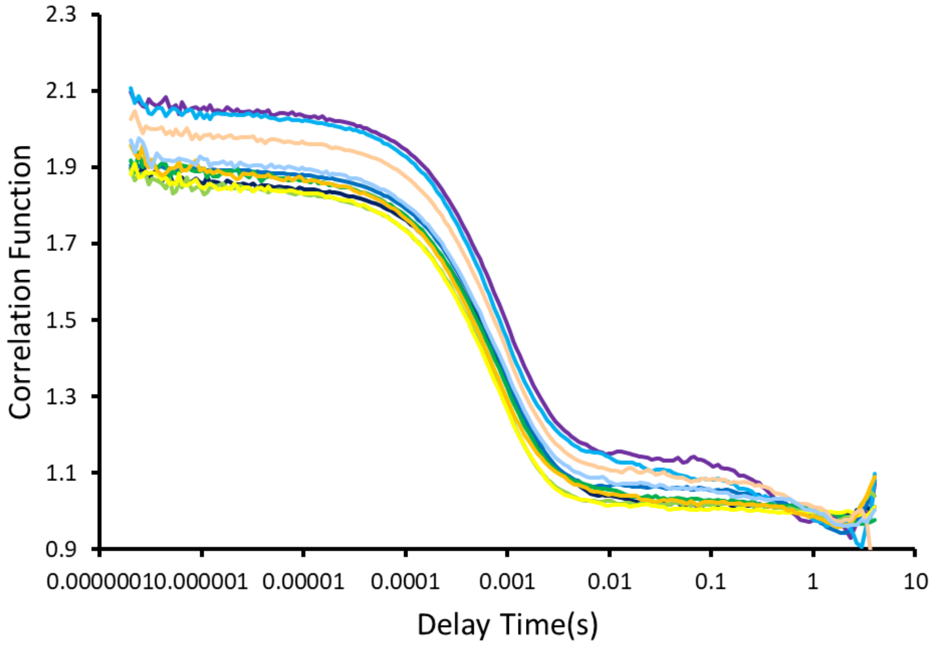

Figure S64 – The correlation function data for 10 DLS runs of **3** (5.56 mM) in H_2O/5.0 % EtOH at 298 K.

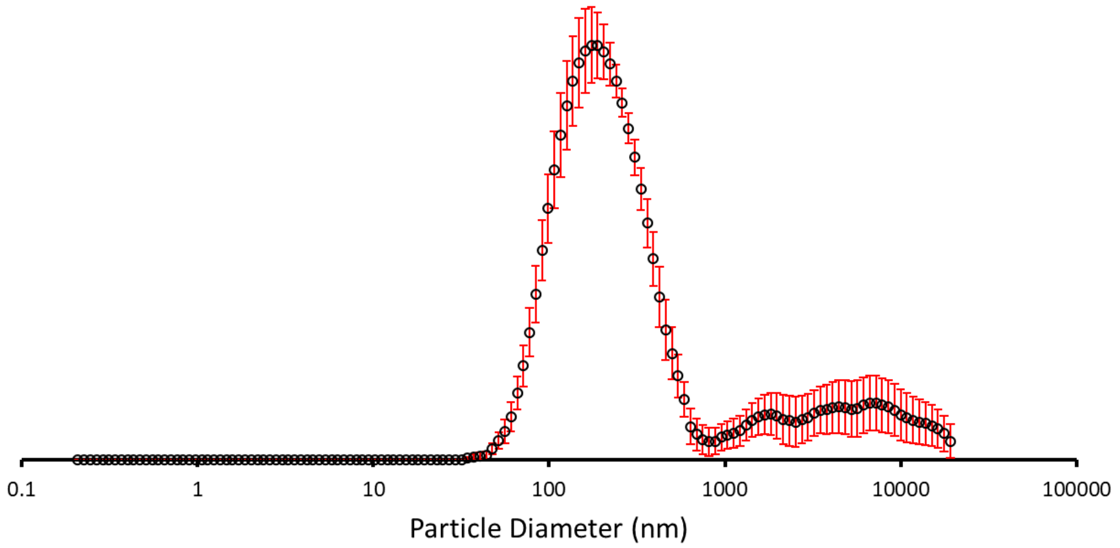

Figure S65 – The average intensity particle size distribution calculated using 10 DLS runs of **4** (5.56 mM) in H_2O/5.0 % EtOH at 298 K, with a peak 1 maxima of 255 nm and a peak 2 maxima of 5118 nm.

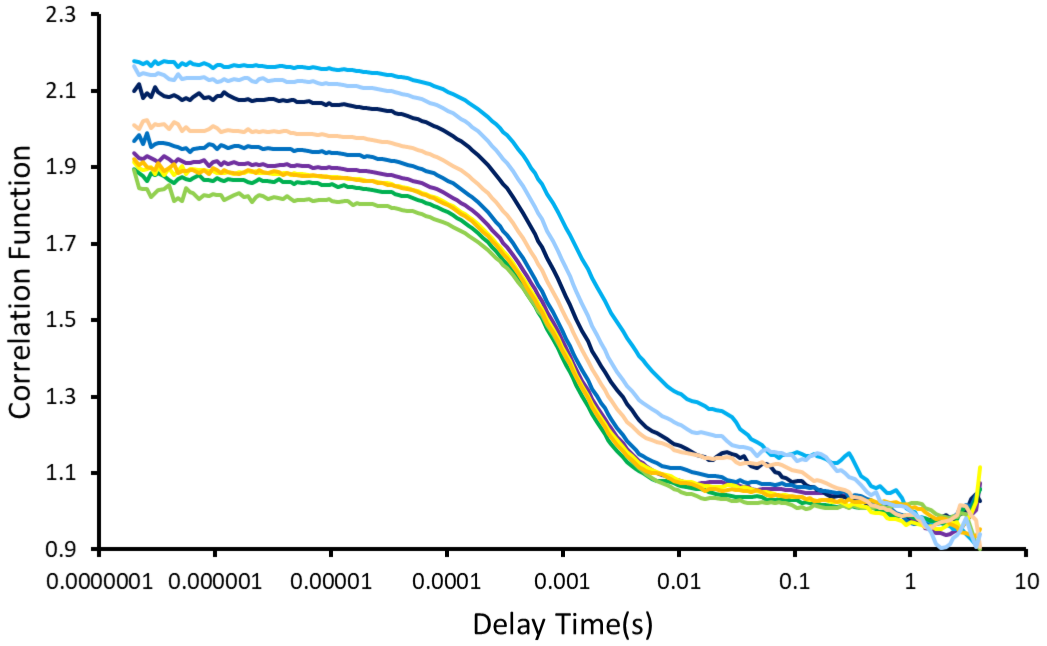

Figure S66 – The correlation function data for 10 DLS runs of **4** (5.56 mM) in H_2O/5.0 % EtOH at 298 K.

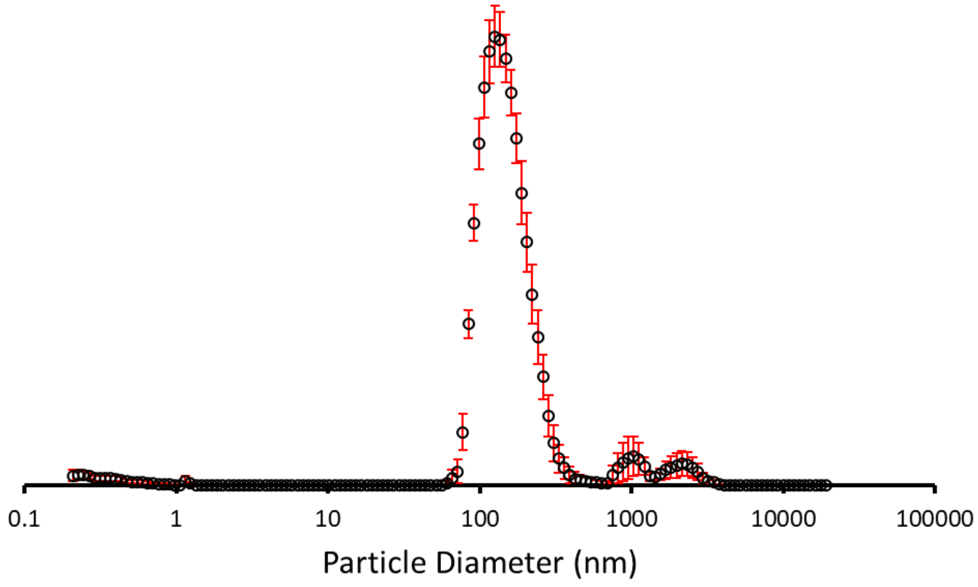

Figure S67 – The average intensity particle size distribution calculated using 10 DLS runs of **5** (5.56 mM) in H_2O/5.0 % EtOH at 298 K, with a peak 1 maxima of 153 nm and a peak 2 maxima of 1328 nm.

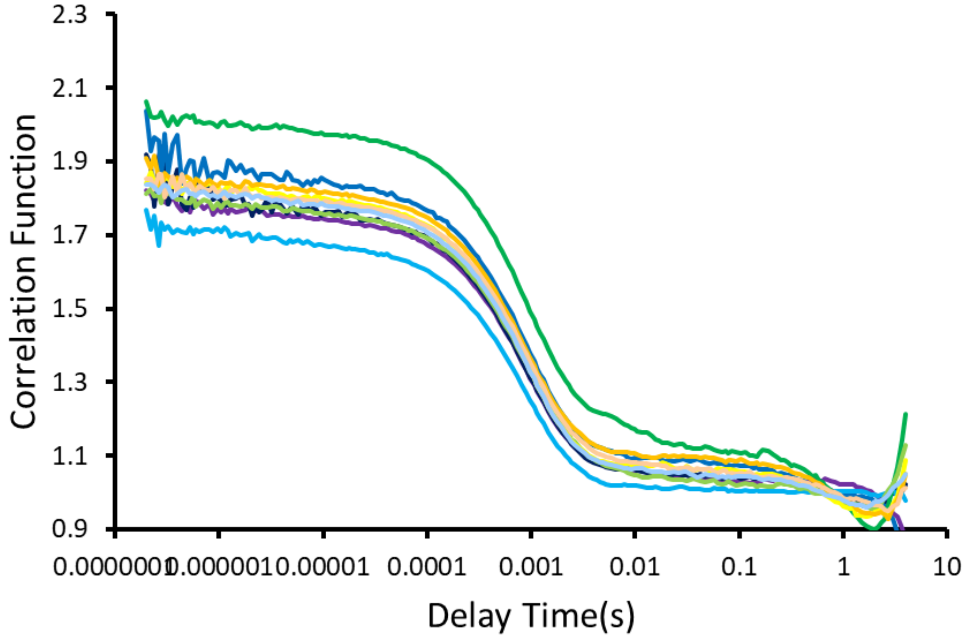

Figure S68 – The correlation function data for 10 DLS runs of **5** (5.56 mM) in H_2O/5.0 % EtOH at 298 K.

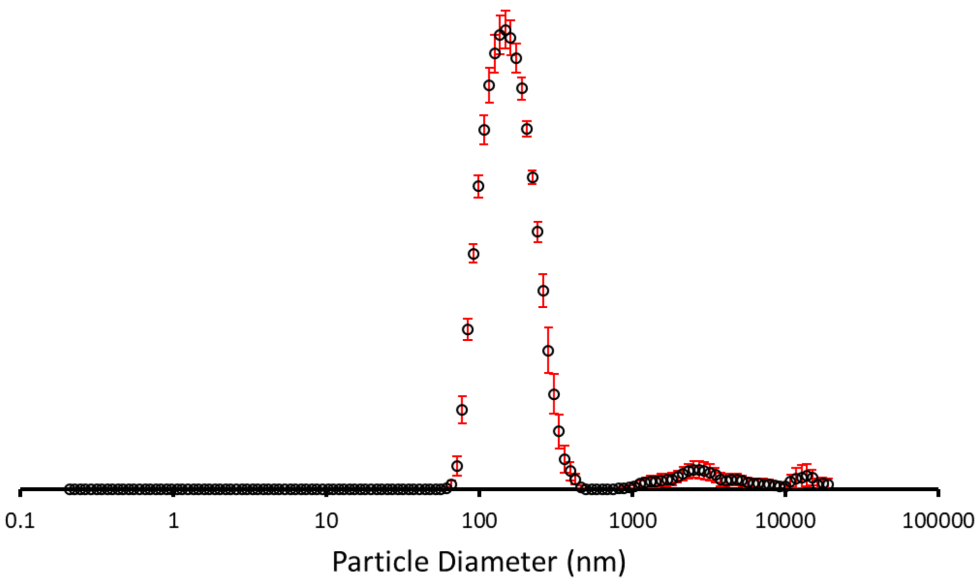

Figure S69 – The average intensity particle size distribution calculated using 10 DLS runs of **6** (5.56 mM) in H_2O/5.0 % EtOH at 298 K, with a peak 1 maxima of 164 nm and a peak 2 maxima of 5868 nm.

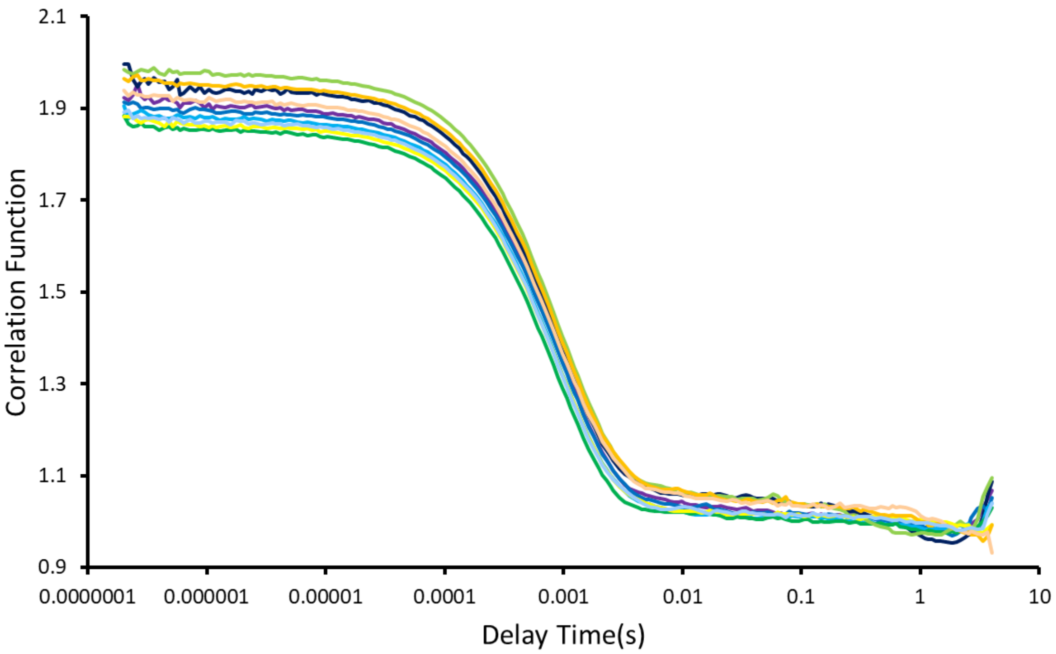

Figure S70 – The correlation function data for 10 DLS runs of **6** (5.56 mM) in H_2O/5.0 % EtOH at 298 K.

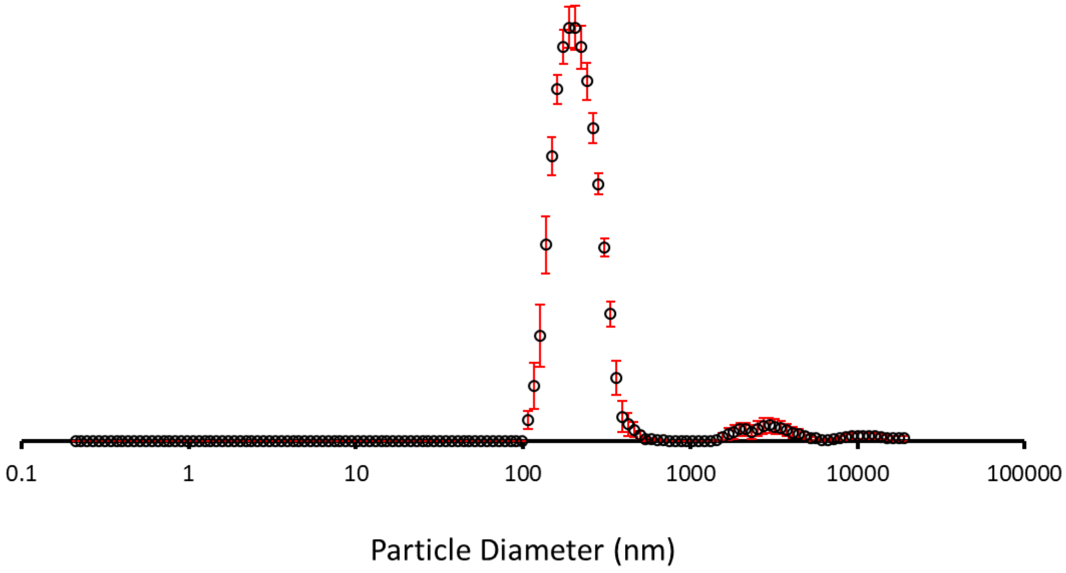

Figure S71 – The average intensity particle size distribution calculated using 10 DLS runs of **7** (5.56 mM) in H_2O/5.0 % EtOH at 298 K, with a peak 1 maxima of 214 nm and a peak 2 maxima of 5177 nm.

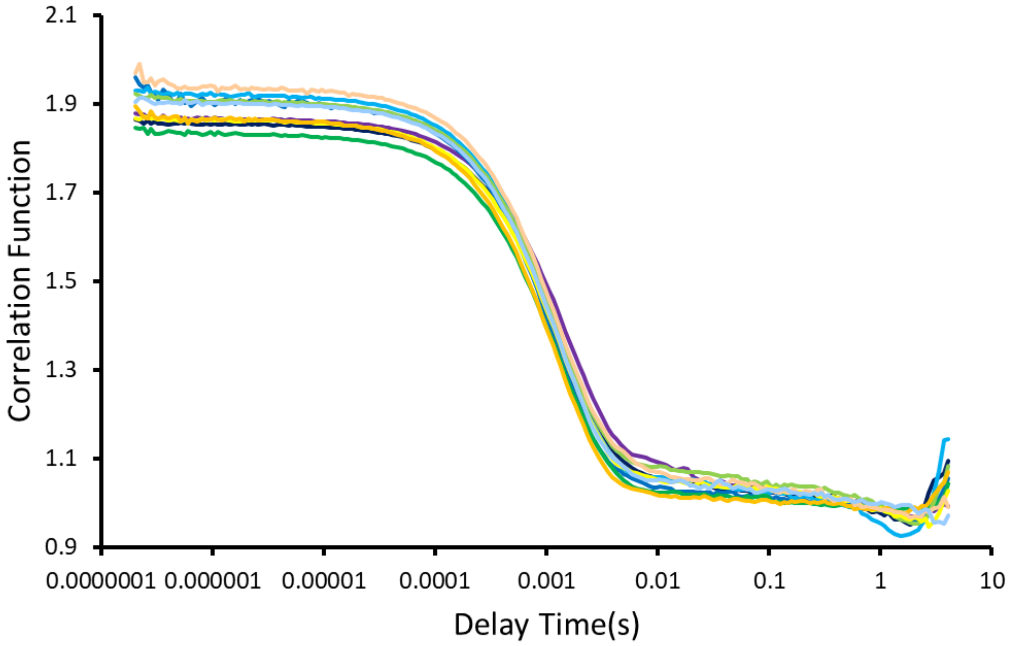

Figure S72 – The correlation function data for 10 DLS runs of **7** (5.56 mM) in H_2O/5.0 % EtOH at 298 K.

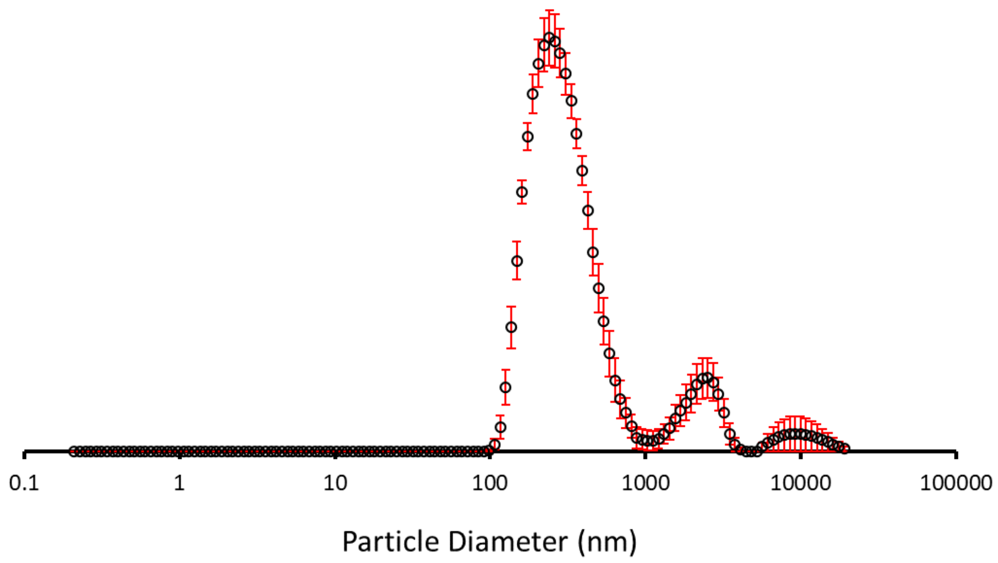

Figure S73 – The average intensity particle size distribution calculated using 10 DLS runs of **8** (5.56 mM) in H_2O/5.0 % EtOH at 298 K, with a peak 1 maxima of 313 nm and a peak 2 maxima of 3417 nm.

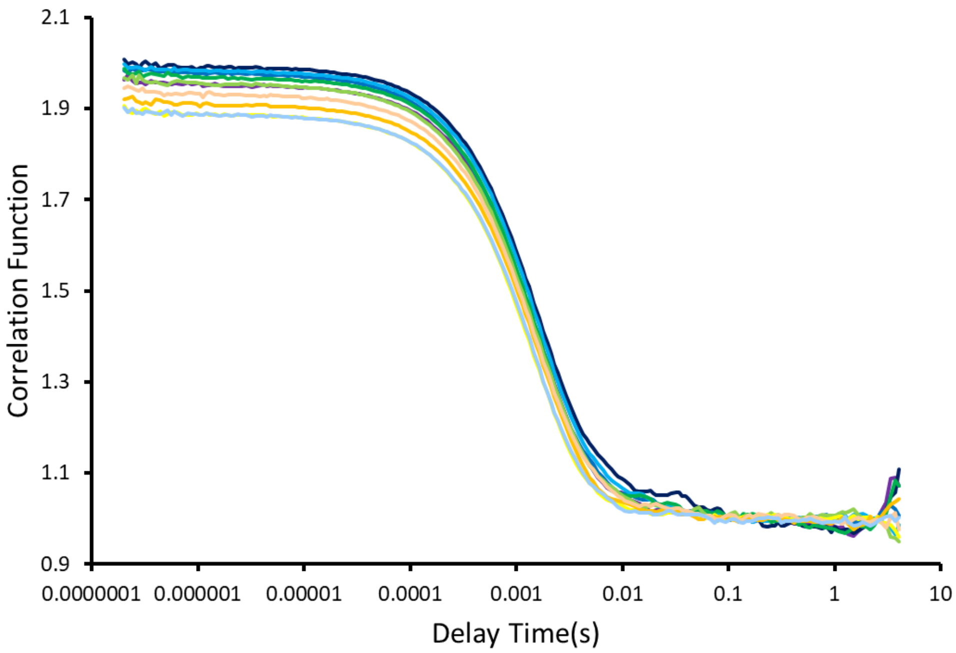

Figure S74 – The correlation function data for 10 DLS runs of **8** (5.56 mM) in H_2O/5.0 % EtOH at 298 K.

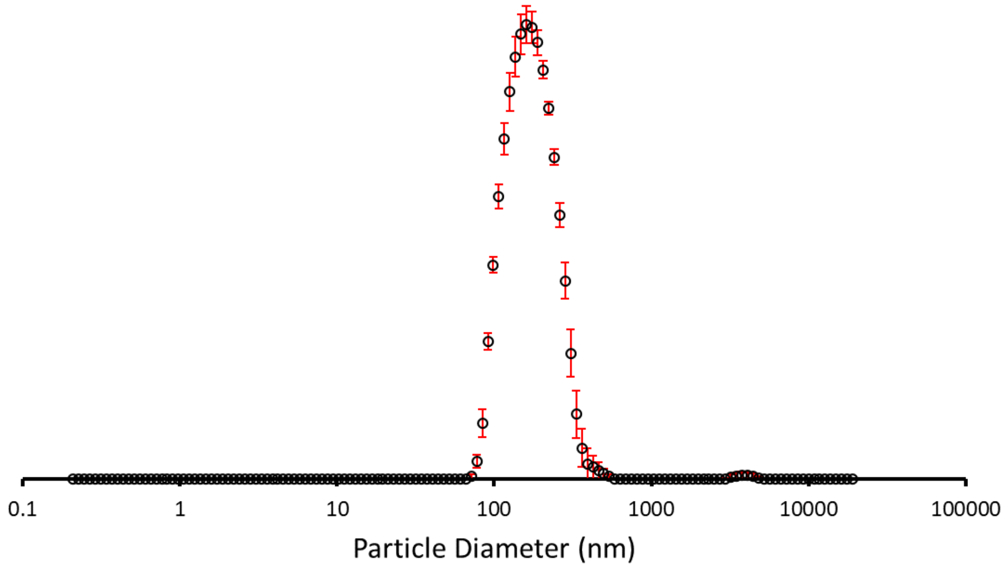

Figure S75 – The average intensity particle size distribution calculated using 10 DLS runs of **9** (5.56 mM) in H_2O/5.0 % EtOH at 298 K, with a peak 1 maxima of 178 nm and a peak 2 maxima of 3866 nm.

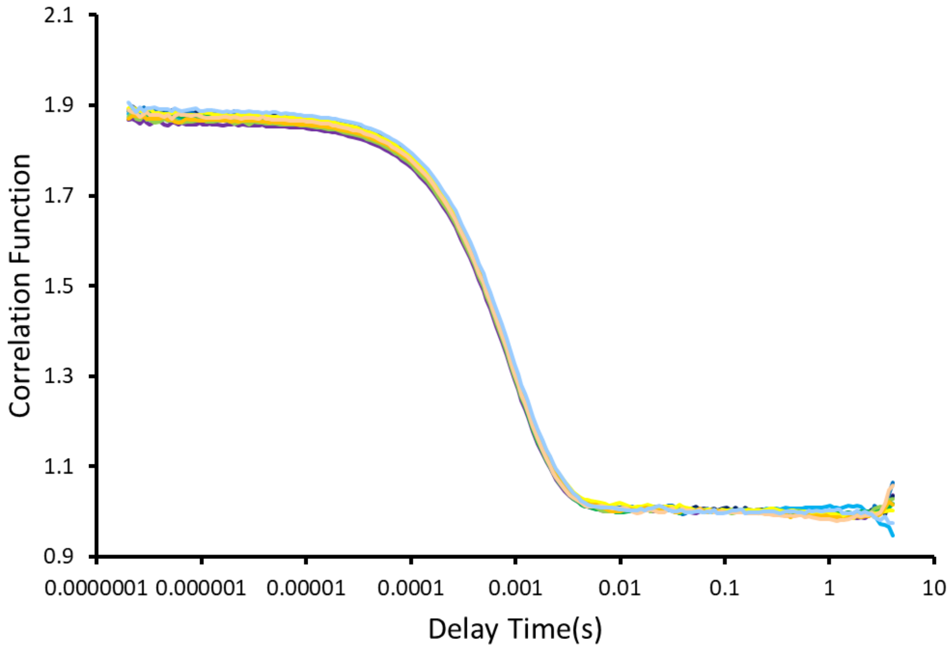

Figure S76 – The correlation function data for 10 DLS runs of **9** (5.56 mM) in H_2O/5.0 % EtOH at 298 K.

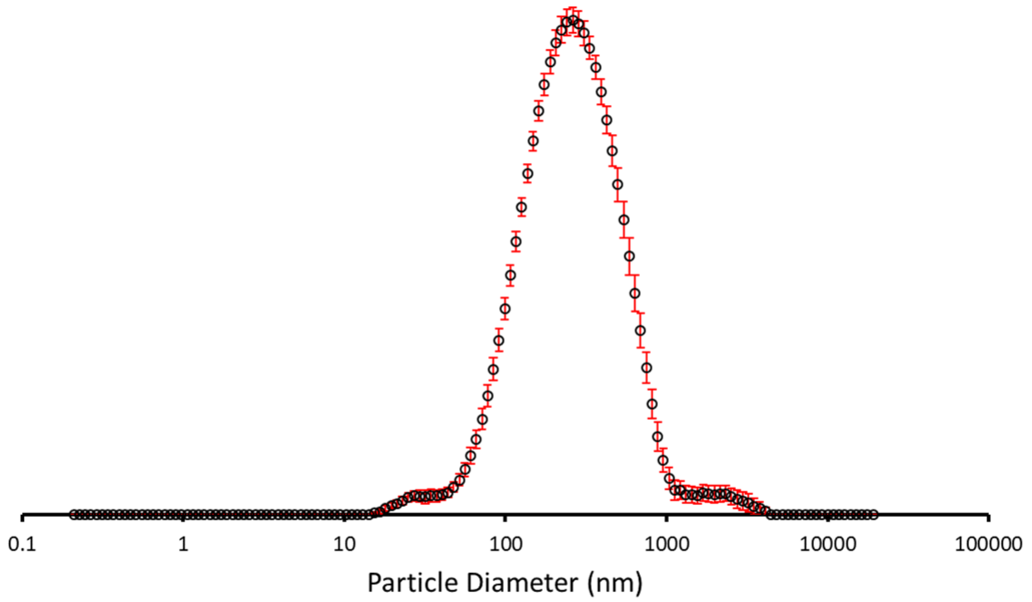

Figure S77 – The average intensity particle size distribution calculated using 10 DLS runs of **10** (5.56 mM) in H_2O/5.0 % EtOH at 298 K, with a peak maxima of 307 nm.

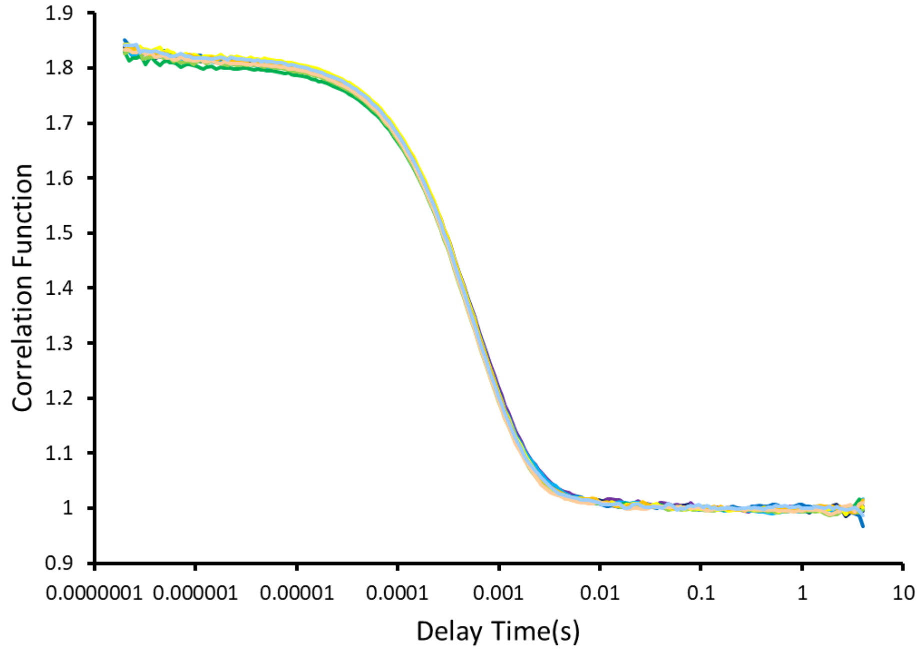

Figure S78 – The correlation function data for 10 DLS runs of **10** (5.56 mM) in H_2O/5.0 % EtOH at 298 K.

Summary

Table S4 - Overview of average DLS intensity particle size distribution peak maxima (aggregate d_H measurements), polydispersity index (PI) determined from DLS data (error < ± 1 % for all data reported) obtained for a H_2O/ 5.0 % EtOH solution of **1** - **10** (5.6 mM) at 298 K. Error = standard error of the mean. All samples underwent an annealing process where they were heated to approximately 313 K before being left to cool to 298 K.

SSA	Peak 1 maxima (nm)	*± Error (nm)*	Peak 2 maxima (nm)	*± Error (nm)*	Polydispersity index (%)	Polydispersity index	*± Error*
**1**	*a*	*a*	*a*	*a*	*a*	*a*	*a*
**2**	140	*1.54*	n/a		18.2 (*± 1.1)*	0.33	*0.00012*
**3**	154	*6.59*	1410	*92.88*	20.2 (*± 0.7)*	0.06	*0.00005*
**4**	255	*39.74*	5118	*1192.20*	27.4 (*± 0.8)*	0.07	*0.00006*
**5**	153	*5.93*	1328	*317.68*	22.7 (*± 1.5)*	0.05	*0.00024*
**6**	164	*3.08*	5868	*1701.87*	21.5 (*± 1.3)*	0.05	*0.00017*
**7**	214	*3.35*	5177	*1316.96*	19.6 (*± 1.7)*	0.04	*0.00028*
**8**	313	*20.99*	3417	*887.21*	25.6 (*± 1.0)*	0.07	*0.00011*
**9**	178	*4.51*	n/a		25.6 (*± 1.0)*	0.07	*0.00019*
**10**	307	*6.77*	n/a		26.0 (*± 0.5)*	0.07	*0.00002*

a - Data quality prevents accurate reporting of d_H or PI values.

Section 11: Zeta potential studies

Zeta potential studies in H_2O/5.0 % EtOH

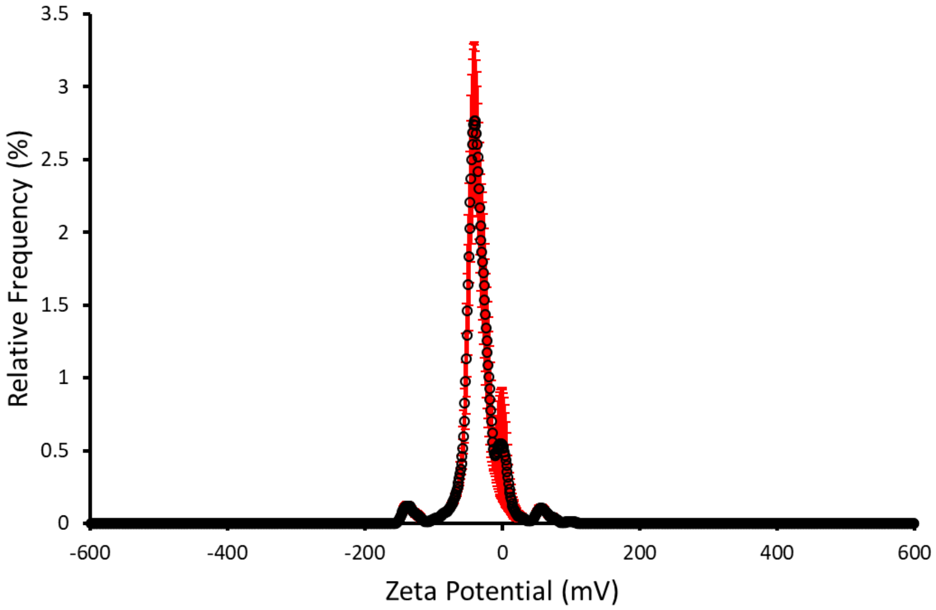

Figure S79 – The average zeta potential distribution for **1** (5.56 mM) in H_2O/5.0 % EtOH calculated using 10 runs at 298 K. Average measurement value = -26 mV.

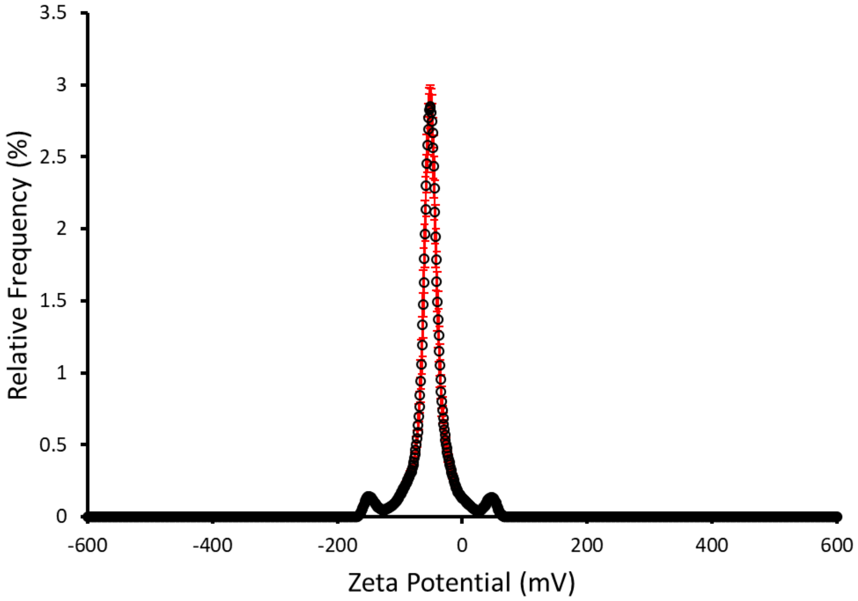

Figure S80 – The average zeta potential distribution for **2** (5.56 mM) in H_2O/5.0 % EtOH calculated using 10 runs at 298 K. Average measurement value = -52 mV.

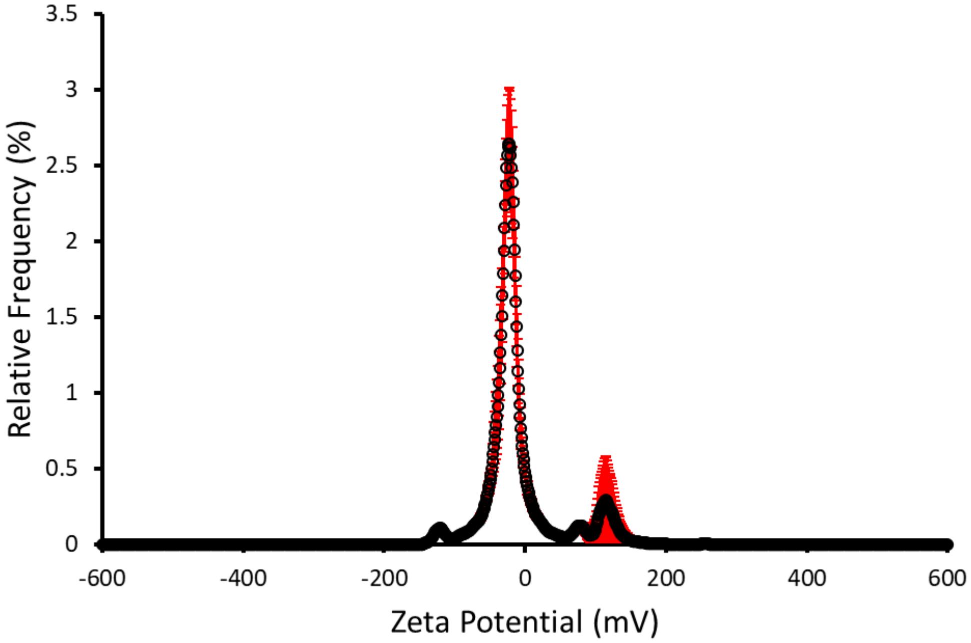

Figure S81 – The average zeta potential distribution for **3** (5.56 mM) in H_2O/5.0 % EtOH calculated using 10 runs at 298 K. Average measurement value = -25 mV.

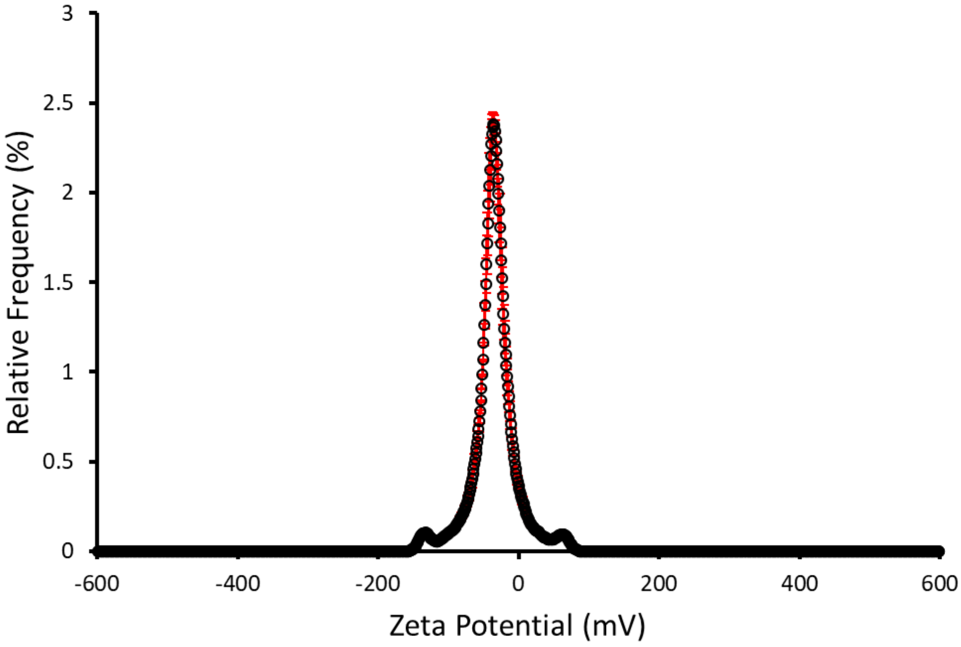

Figure S82 – The average zeta potential distribution for **4** (5.56 mM) in H_2O/5.0 % EtOH calculated using 10 runs at 298 K. Average measurement value = -40 mV.

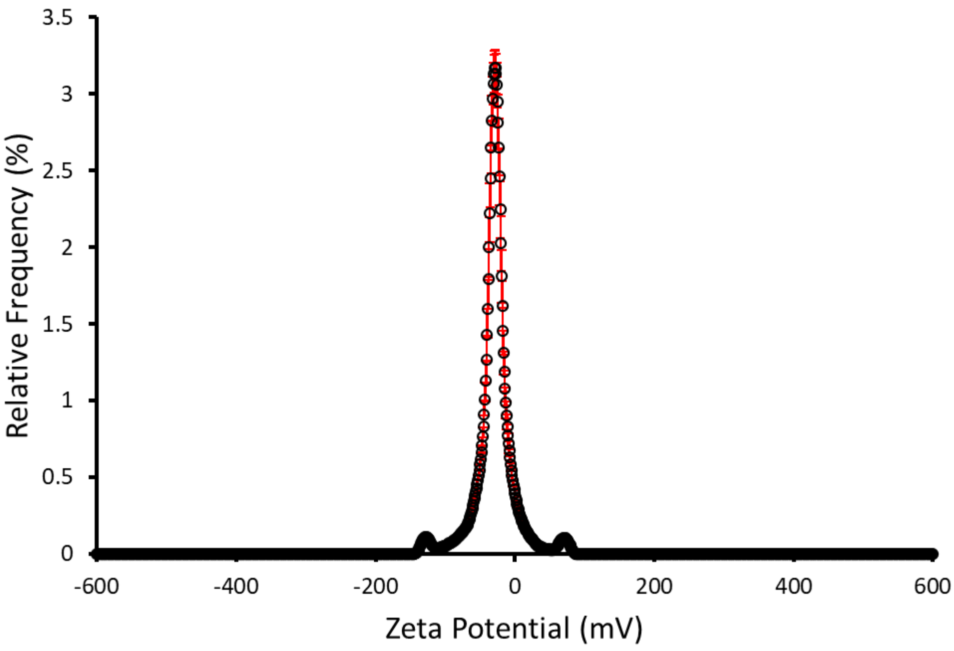

Figure S83 – The average zeta potential distribution for **5** (5.56 mM) in H_2O/5.0 % EtOH calculated using 10 runs at 298 K. Average measurement value = -28 mV.

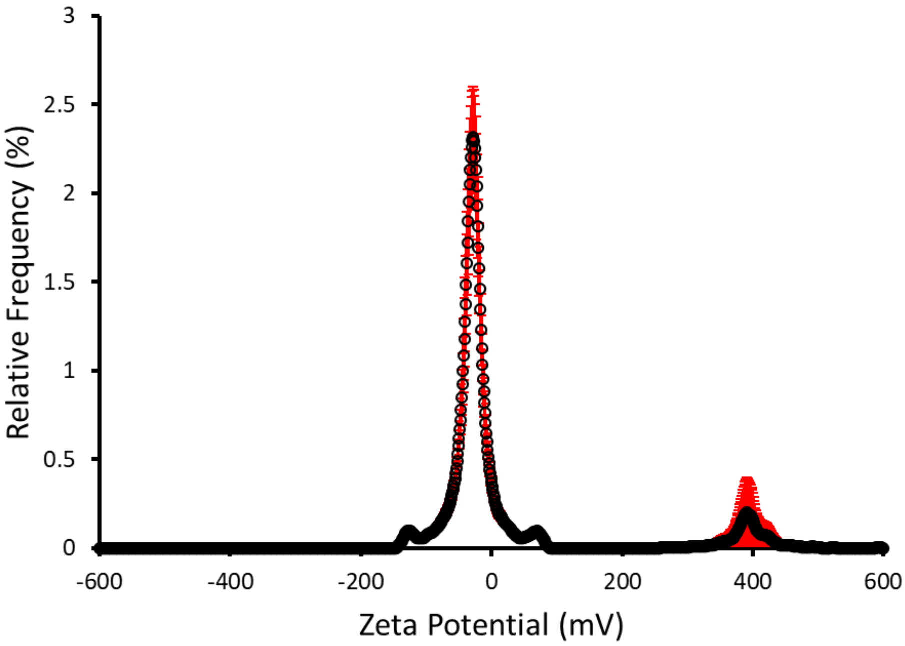

Figure S84 – The average zeta potential distribution for **6** (5.56 mM) in H_2O/5.0 % EtOH calculated using 10 runs at 298 K. Average measurement value = -51 mV.

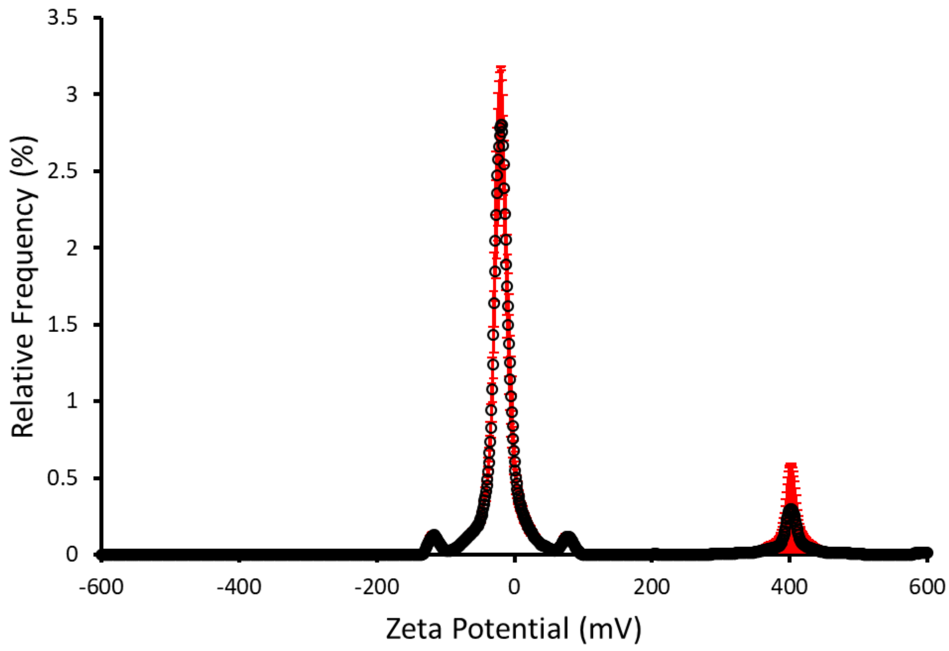

Figure S85 – The average zeta potential distribution for **7** (5.56 mM) in H_2O/5.0 % EtOH calculated using 10 runs at 298 K. Average measurement value = -12 mV.

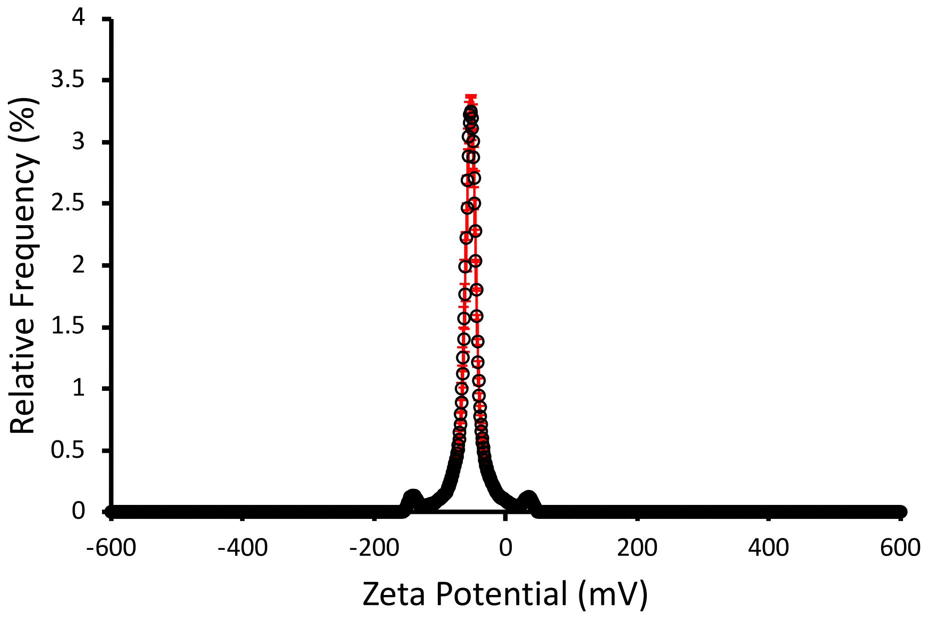

Figure S86 – The average zeta potential distribution for **8** (5.56 mM) in H_2O/5.0 % EtOH calculated using 10 runs at 298 K. Average measurement value = -29 mV.

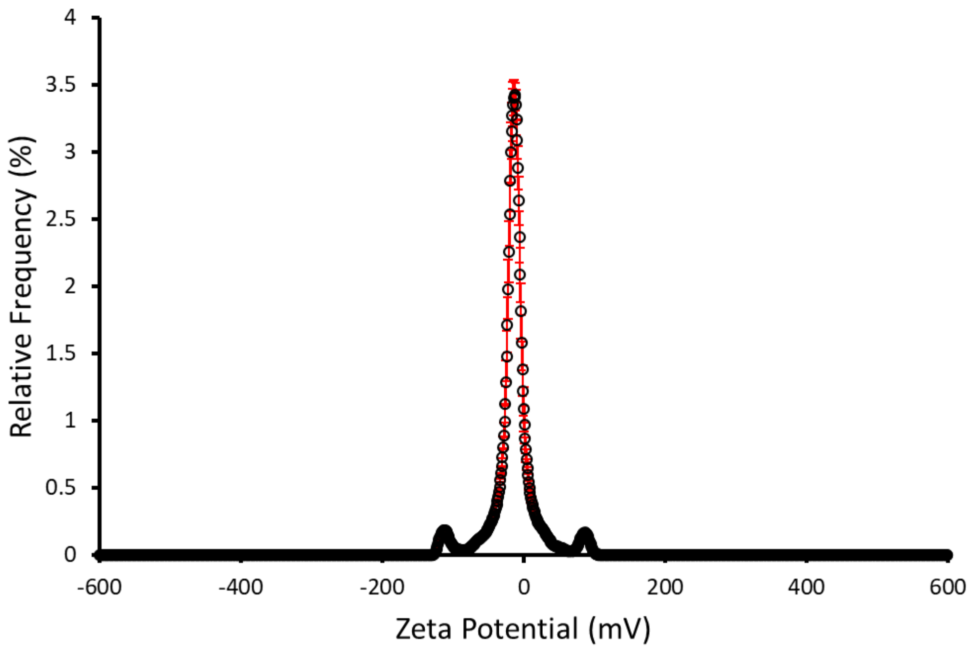

Figure S87 – The average zeta potential distribution for **9** (5.56 mM) in H_2O/5.0 % EtOH calculated using 10 runs at 298 K. Average measurement value = -17 mV.

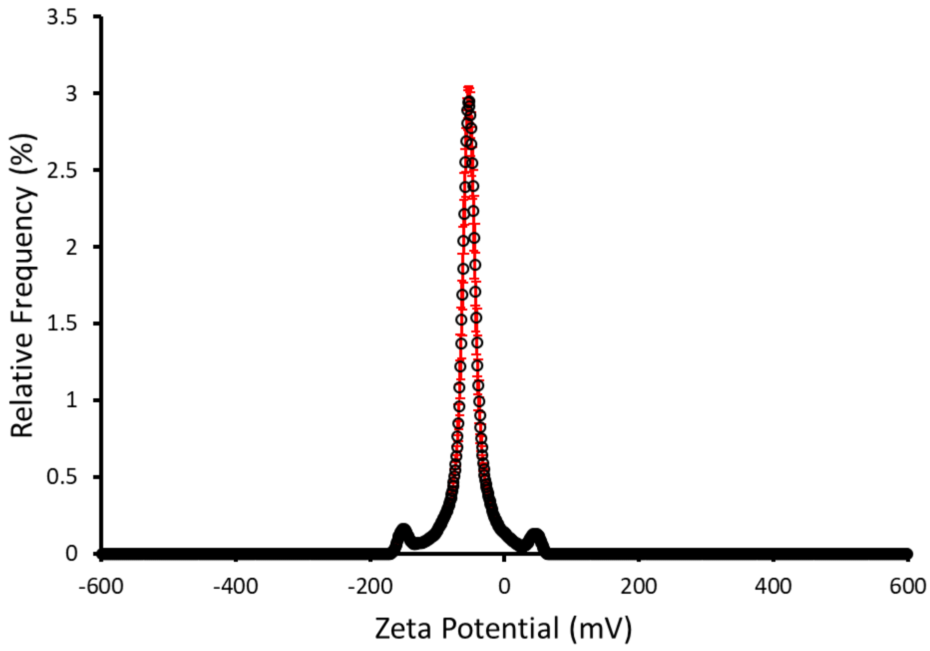

Figure S88 – The average zeta potential distribution for **10** (5.56 mM) in H_2O/5.0 % EtOH calculated using 10 runs at 298 K. Average measurement value = -51 mV.

Summary

Table S5 – Summary of the average zeta potential for **1**, **2**, **3**, **4**, **5**, **6**, **7**, **8**, **9** and **10** at 5.56 mM in H_2O/5.0 % EtOH.

SSA	Zeta Potential (mV)	*± Error (mV)*
**1**	-25.60	0.77
**2**	-51.83	0.92
**3**	-25.07	0.86
**4**	-40.48	0.57
**5**	-28.32	0.31
**6**	-51.02	0.33
**7**	-12.45	0.39
**8**	-28.71	0.65
**9**	-17.48	0.33
**10**	-51.47	0.65

Section 12: Surface tension measurements and critical aggregate concentration (CAC) determination

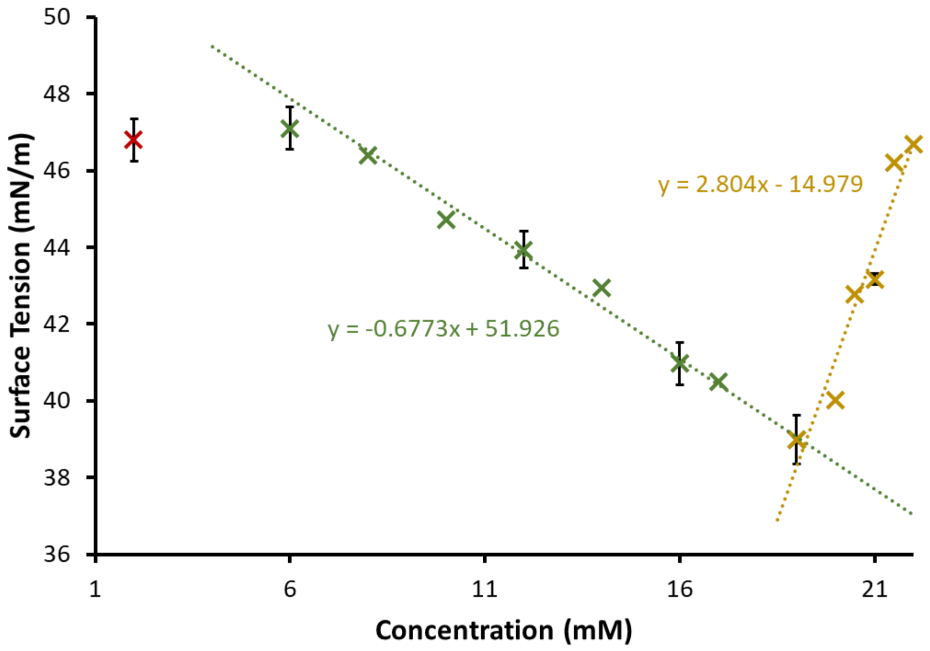

Figure S89 – Determination of the critical aggregate concentration of **1,** 19.23 mM at surface tension 38.90 mN/m in H_2O/5.0 % EtOH.

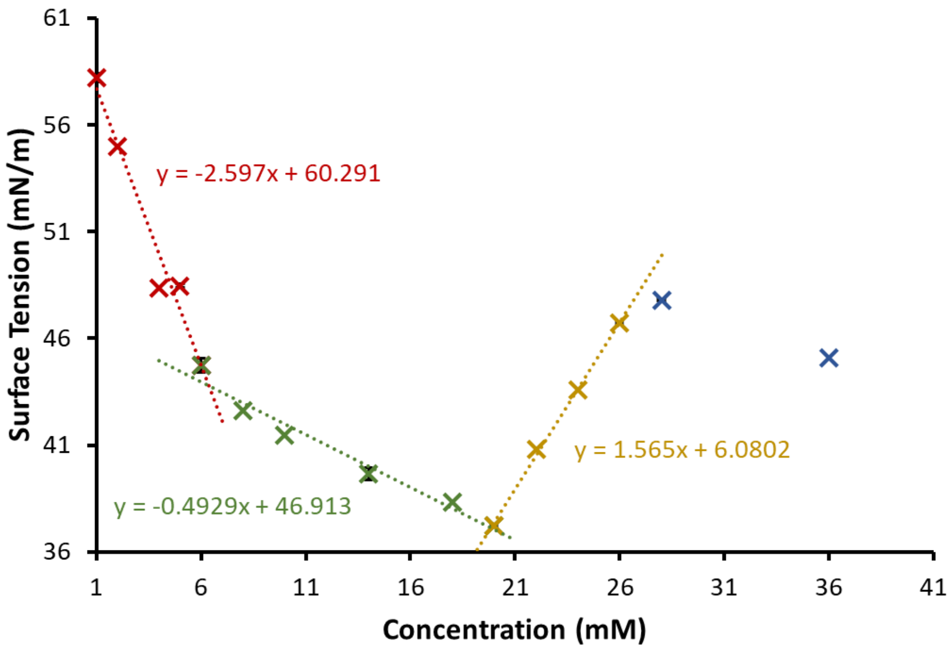

Figure S90 – Determination of the critical aggregate concentration of **2,** 19.84 mM at surface tension 37.13 mN/m in H_2O/5.0 % EtOH.

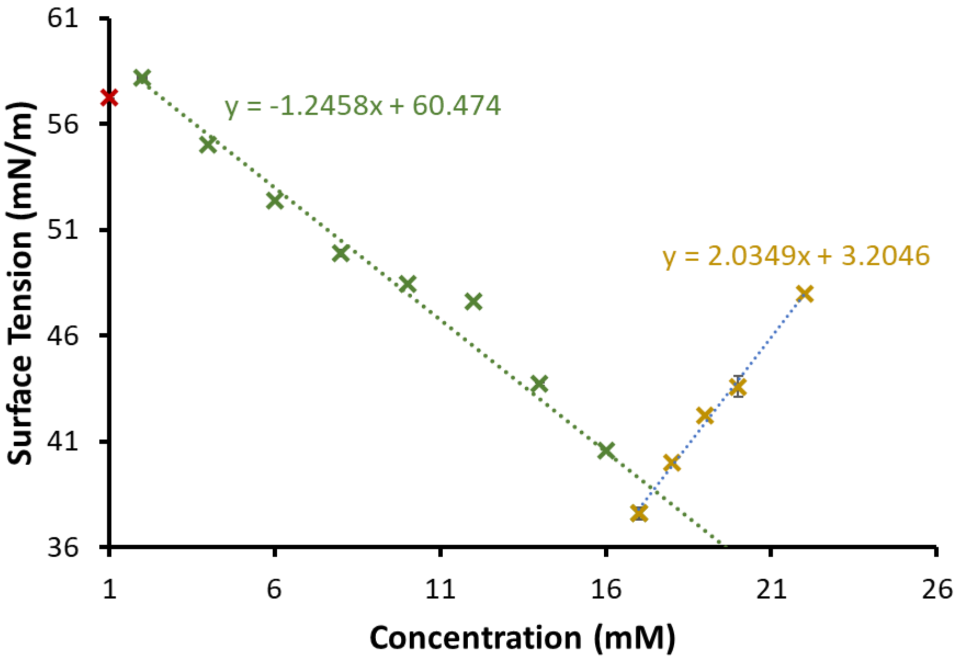

Figure S91 – Determination of the critical aggregate concentration of **3,** 17.46 mM at surface tension 38.73 mN/m in H_2O/5.0 % EtOH.

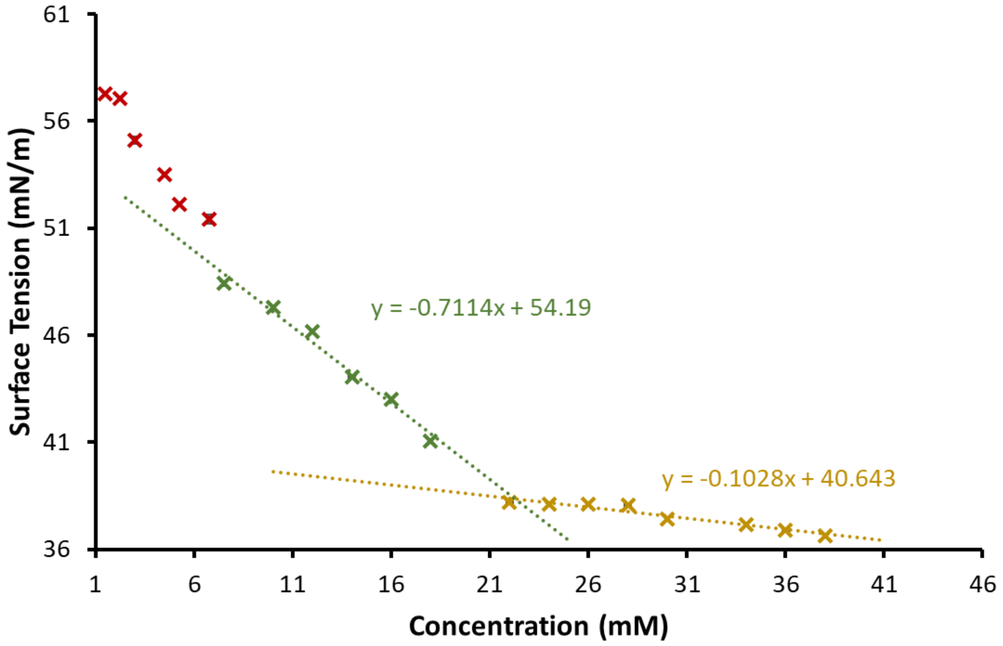

Figure S92 – Determination of the critical aggregate concentration of **4,** 22.26 mM at surface tension 38.35 mN/m in H_2O/5.0 % EtOH.

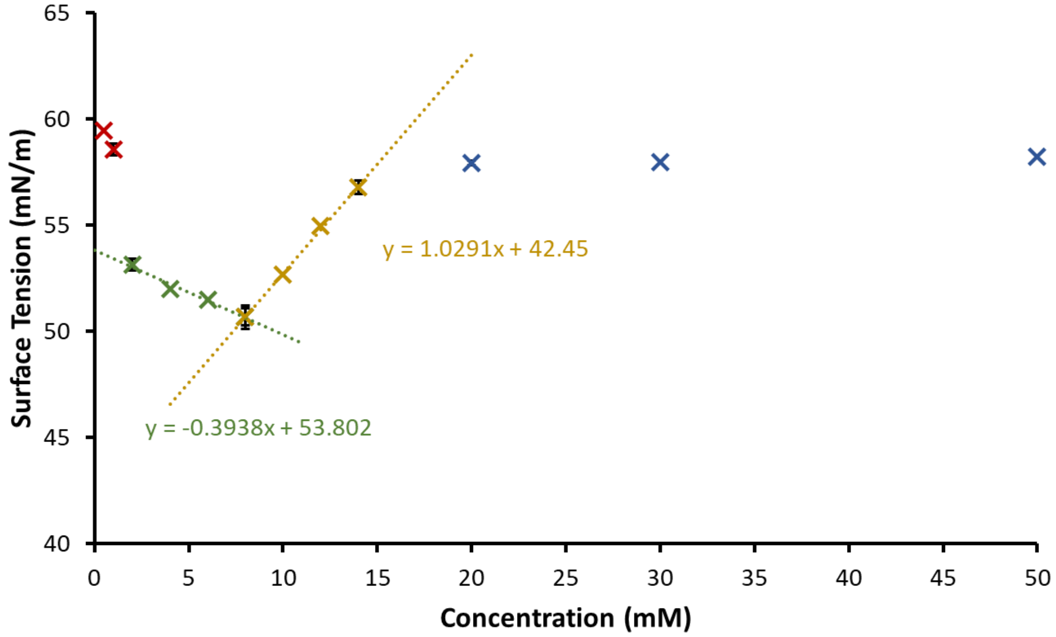

Figure S93 - Determination of the critical aggregate concentration of **5** in H_2O/5.0 % EtOH using surface tension measurements could not be determined at surface tension 58.22 mN/m

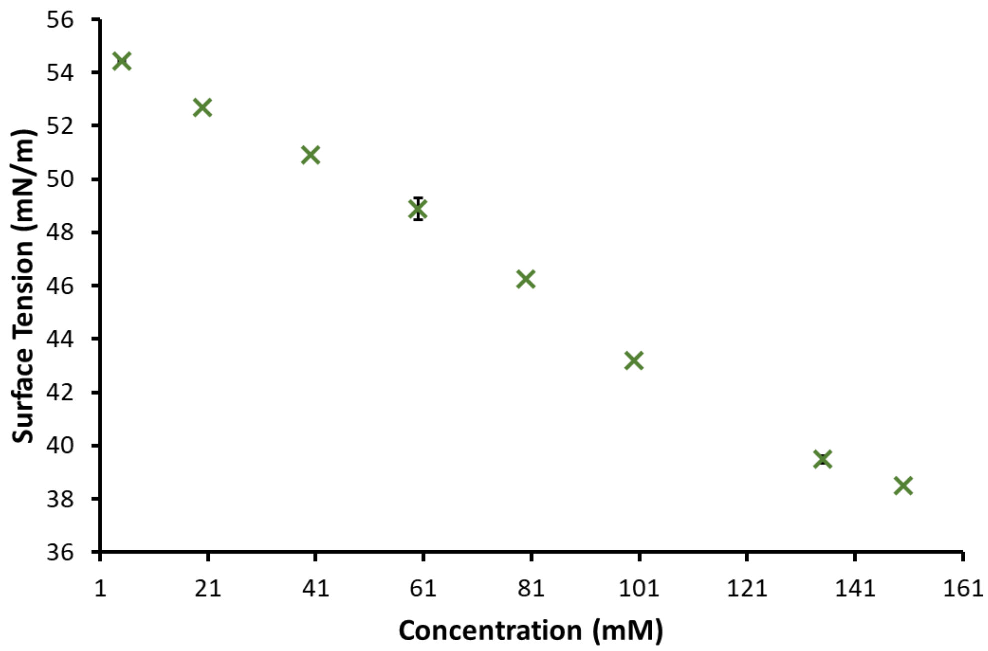

Figure S94 - Determination of the critical aggregate concentration of **6** in H_2O/5.0 % EtOH using surface tension measurements. The CAC is above the limit of solubility (151.0 mM), at which the surface tension is 38.24 mN/m.

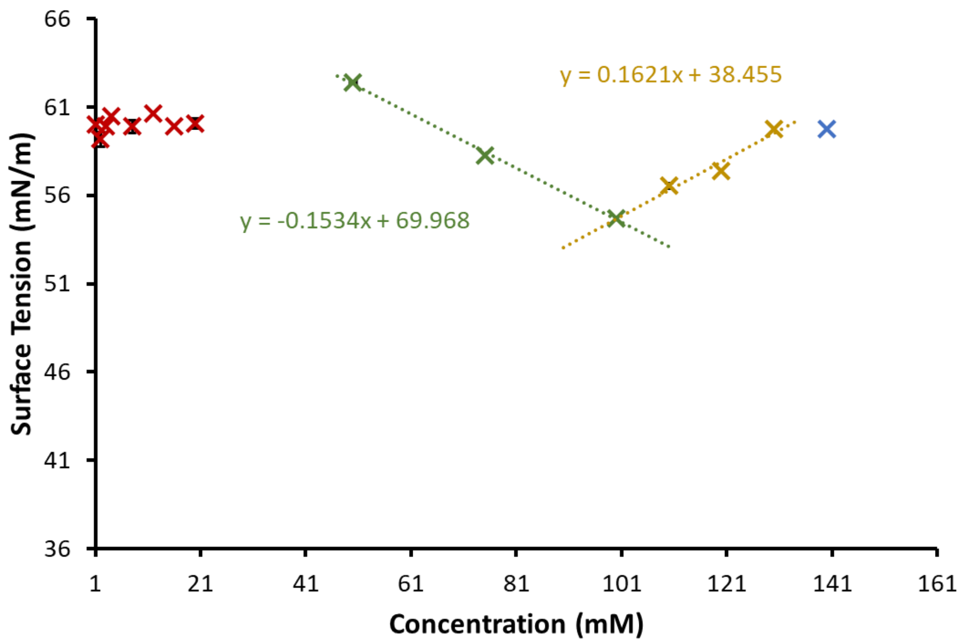

Figure S95 - Determination of the critical aggregate concentration of **7** in H_2O/5.0 % EtOH using surface tension measurements could not be determined at surface tension 54.72 mN/m

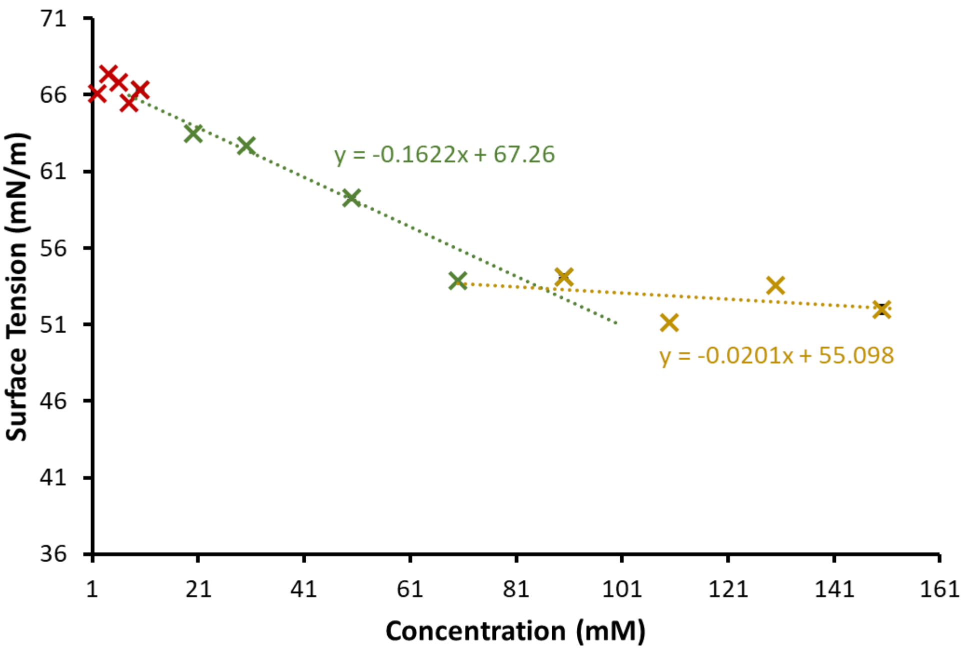

Figure S96 - Determination of the critical aggregate concentration of **8** in H_2O/5.0 % EtOH using surface tension measurements could not be determined at surface tension 51.98 mN/m

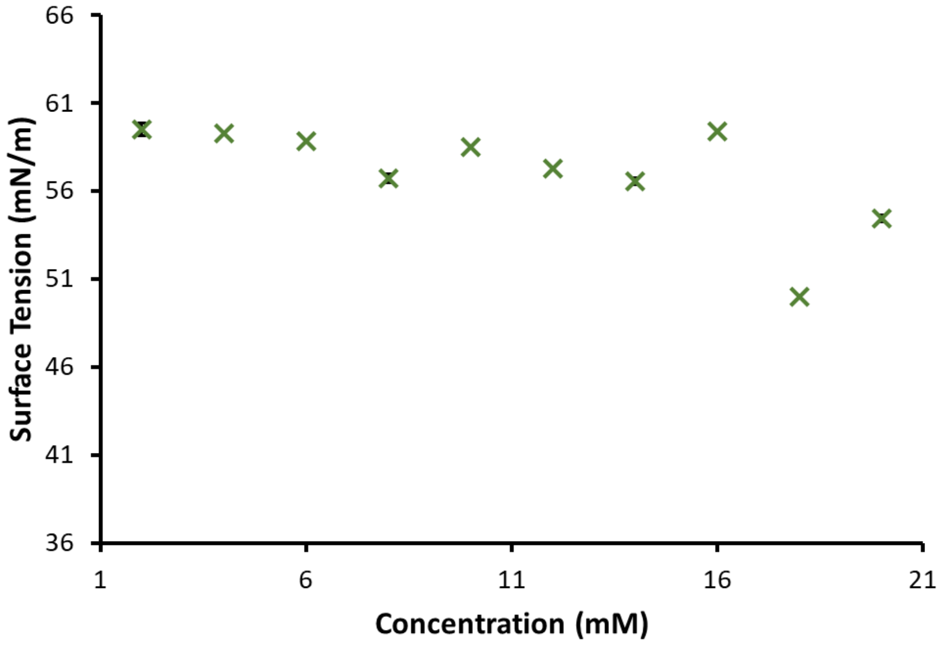

Figure S97 - Determination of the critical aggregate concentration of **9** in H_2O/5 % EtOH using surface tension measurements. The CAC is above the limit of solubility (20.00 mM), at which the surface tension is 54.44 mN/m.

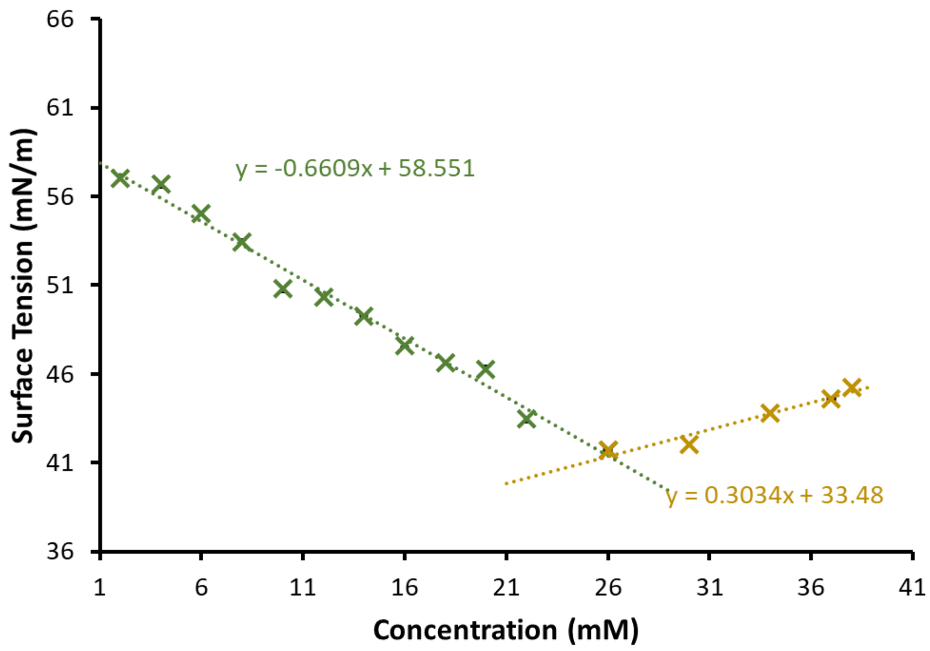

Figure S98 – Determination of the critical aggregate concentration of **10,** 26.00 mM at surface tension 41.37 mN/m in H_2O/5.0 % EtOH.

Summary

Table S6 – Summary of the critical aggregate concentration (CAC) and surface tension at CAC for **1**, **2**, **3**, **4**, **5**, **6**, **7**, **8**, **9** and **10** in H_2O/5.0 % EtOH.

SSA	CAC (mM)	Surface tension (mN/m)
**1**	19.23	38.90
**2**	19.84	37.13
**3**	17.46	38.73
**4**	22.26	38.35
**5**	*b*	58.22
**6**	*a*	38.24
**7**	*b*	54.72
**8**	*b*	51.98
**9**	*a*	54.44
**10**	26.00	41.37

a = Limit of solubility
b = CAC could not be determined

Section 13: Single crystal X-ray structures

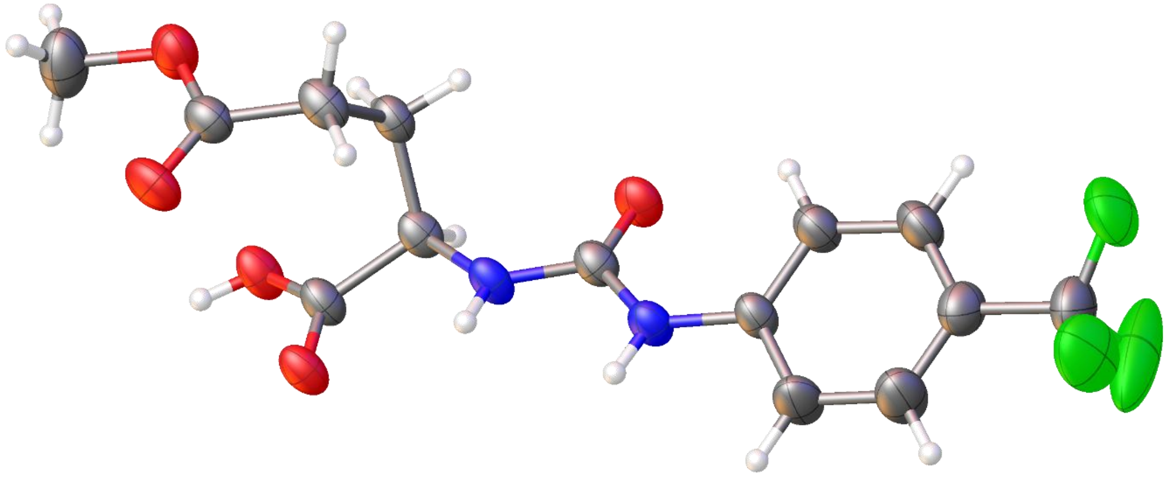

Figure S99 – Single crystal X-ray structure of methyl ester of **3**: red = oxygen; green = fluorine; blue = nitrogen; white = hydrogen; grey = carbon. CCDC 2388585, $C_{14}H_{15}F_3N_2O_5$ (M = 348.24): monoclinic, space group C 1 2/c 1, a = 27.781(3) Å, b = 10.9013(10) Å, c = 10.04616(7) Å, α = 90°, β = 93.477(3)°, γ = 90°, V = 3035.5(5) Å^3, Z = 8, T = 150(1) K, CuK\α = 1.5418 Å, Dcalc = 1.524 g/cm^3, 11086 reflections measured (8.708 ≤ 2Θ ≤ 144.946), 2932 unique (R_{int} = 0.0912**,** R_{sigma} = 0.0734) which were used in all calculations. The final R_1 was 0.1299 (I > 2σ(I)) and wR_2 was 0.4072 (all data).

Table S7 – Hydrogen bond distances and angles observed for methyl ester of **3**, calculated from the single crystal X-ray structure shown in Figure S99.

Hydrogen bond donor	Hydrogen atom	Hydrogen bond acceptor	Hydrogen bond length (D•••A) (Å)	Hydrogen bond angle (D-H•••A) (°)
N1	H1	O4	3.139 (7)	141.6 (4)
N2	H2	O2	2.847 (8)	167.7 (4)
O3	H3	O5	2.620(7)	163.1 (4)

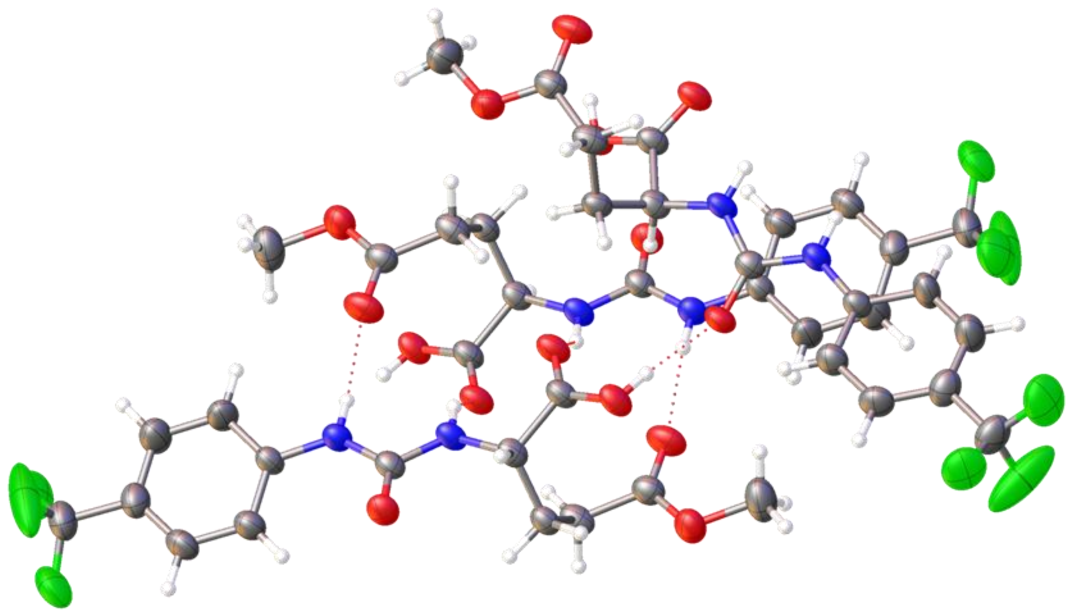

Figure S100 – Single crystal X-ray showing the extended hydrogen bonded network of the methyl ester of **3**: red = oxygen; green = fluorine; blue = nitrogen; white = hydrogen; grey = carbon. TBA counter cations have been omitted for clarity.

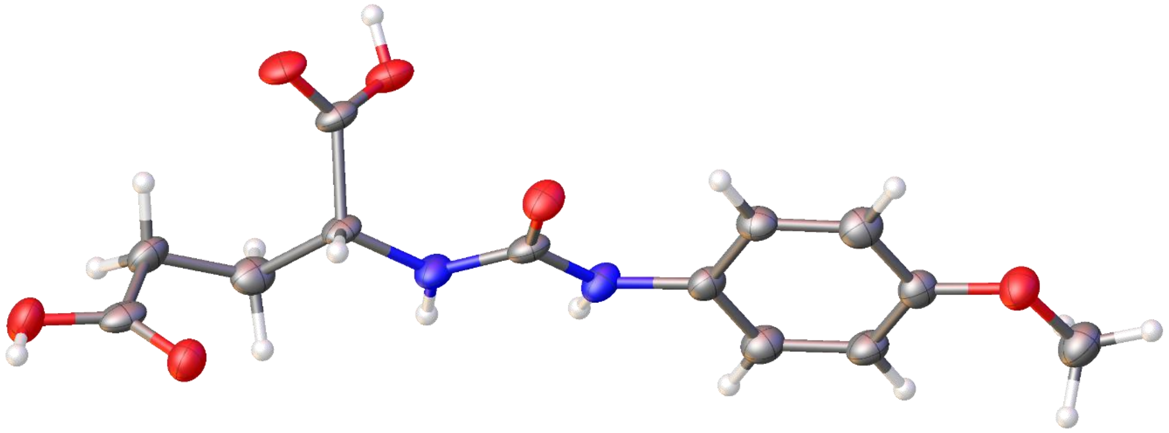

Figure S101 – Single crystal X-ray structure of **5**: red = oxygen; blue = nitrogen; white = hydrogen; grey = carbon. CCDC 2388586, $C_{13}H_{16}N_2O_6$ (M = 296.28): monoclinic, space group P 1 21/C 1, a = 27.123 (3) Å, b = 6.2205 (5) Å, c = 7.7852 (7) Å, α = 27.123 (3)°, β = 90.642 (10)°, γ = 90°, V = 1313.4(2) Å^3, Z = 4, T = 150(1) K, CuK\α = 1.5418 Å, Dcalc = 1.498 g/cm^3, 8232 reflections measured (9.784 ≤ 2Θ ≤ 143.906), 2539 unique (R_{int} = 0.0941, R_{sigma} = 0.0913) which were used in all calculations. The final R_1 was 0.1379 (I > 2σ(I)) and wR_2 was 0.4113 (all data).

Table S8 – Hydrogen bond distances and angles observed for **5**, calculated from the single crystal X-ray structure shown in Figure S101.

Hydrogen bond donor	Hydrogen atom	Hydrogen bond acceptor	Hydrogen bond length (D•••A) (Å)	Hydrogen bond angle (D-H•••A) (°)
N1	H1	O2	3.026 (8)	148.1 (5)
N1	H1	O3	3.889 (9)	155.5 (4)
N2	H2	O2	3.327(9)	123.3 (5)
N2	H2	O4	2.978 (8)	149.3 (5)
O3	H3	O2	2.610 (8)	161.0 (5)
O6	H6	O5	2.652 (9)	172.6 (5)

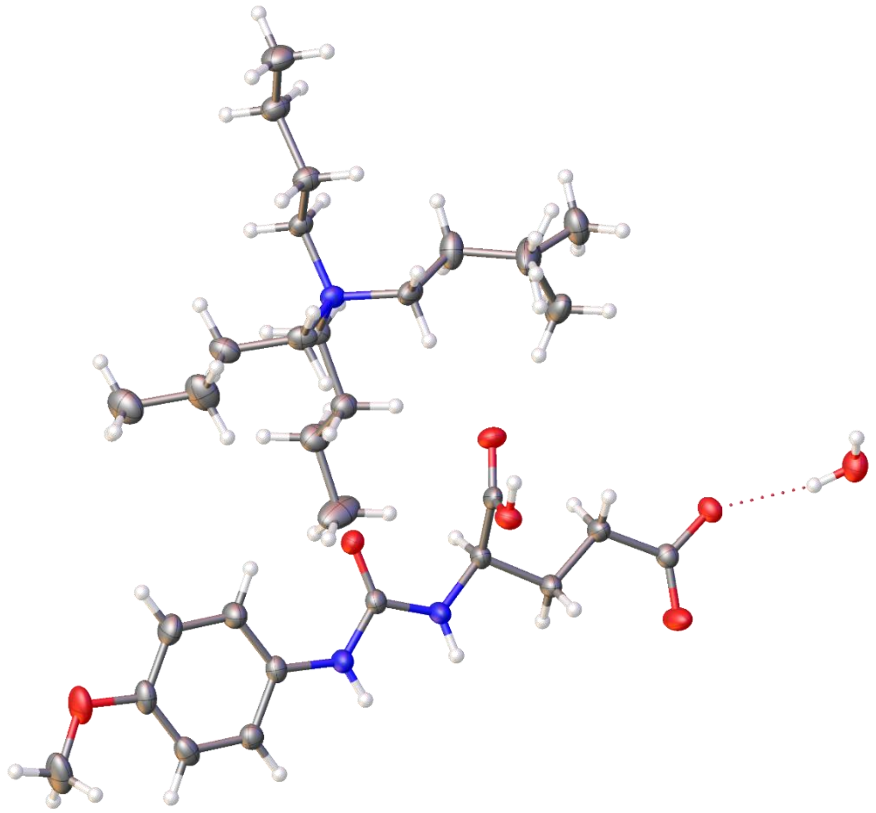

Figure S102 – Single crystal X-ray structure of mono-deprotonated of **6**: red = oxygen; blue = nitrogen; white = hydrogen; grey = carbon. CCDC 2388584, $C_{29}H_{53}N_3O_7$ (M = 555.74): triclinic, space group P -1, a = 8.6519(3) Å, b = 9.5272(3) Å, c = 19.0975(7) Å, α = 93.880(3)°, β = 95.670(3)°, γ = 100.995(3)°, V = 1521.68(9) Å^3, Z = 2, T = 150(1) K, CuK\α = 1.5418 Å, Dcalc = 1.205 g/cm^3, 10263 reflections measured (9.344 ≤ 2Θ ≤ 133.184), 5412 unique (R_{int} = 0.0294, R_{sigma} = 0.0366) which were used in all calculations. The final R_1 was 0.0410 (I > 2σ(I)) and wR_2 was 0.1101 (all data). Internal angle of dimerization = 180.00 (18) °.

Table S9 – Hydrogen bond distances and angles observed for mono-deprotonated of **6**, calculated from the single crystal X-ray structure shown in Figure S102.

Hydrogen bond donor	Hydrogen atom	Hydrogen bond acceptor	Hydrogen bond length (D•••A) (Å)	Hydrogen bond angle (D-H•••A) (°)
N1	H1	O6	2.7828 (16)	159.93 (8)
N2	H2	O6	2.8720 (16)	148.46 (8)
O3	H3	O5	2.5465 (14)	172.51 (8)
O7	H7A	O5	2.9165 (15)	167.40 (8)
O7	H7B	O2	2.9780 (16)	172.63 (8)

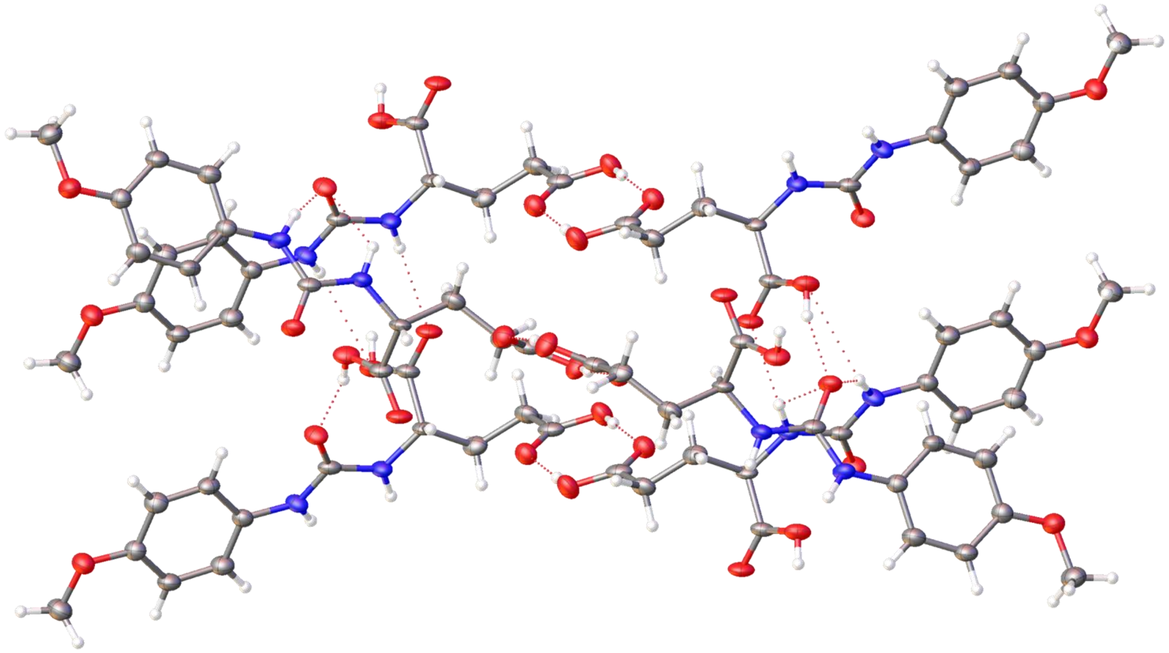

Figure S103 – Single crystal X-ray showing the extended hydrogen bonded network of mono-deprotonated **6**: red = oxygen; blue = nitrogen; white = hydrogen; grey = carbon. TBA counter cations have been omitted for clarity.

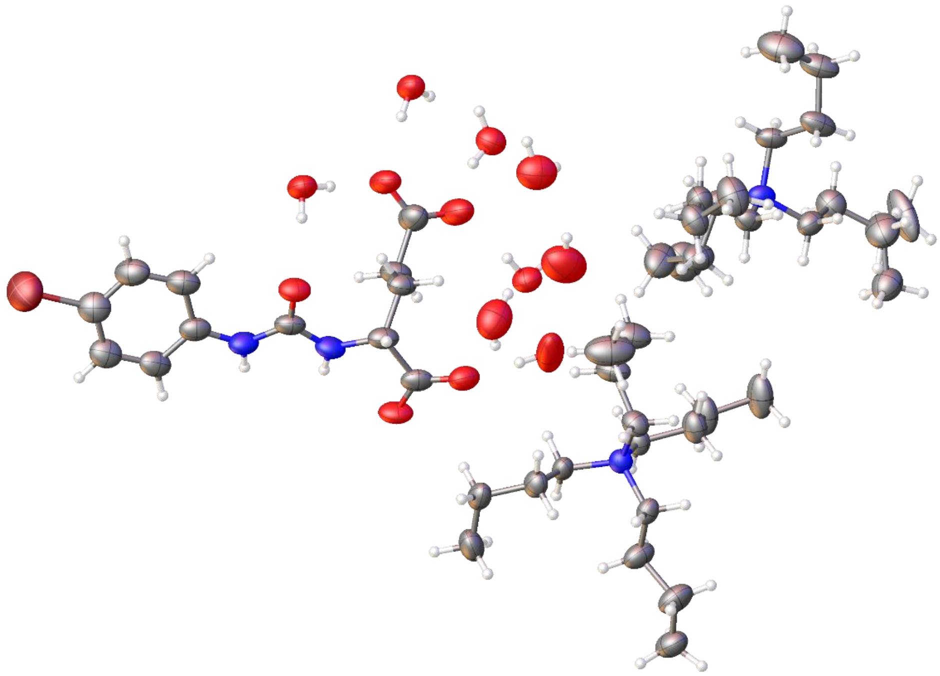

Figure S104 – Single crystal X-ray structure of **10**: red = oxygen; purple = bromine; blue = nitrogen; white = hydrogen; grey = carbon. CCDC 2388587, $C_{44}H_{97}BrN_4O_{12}$ (M = 954.16): orthorhombic, space group p b c n, a = 19.3063(13) Å, b = 16.619(2) Å, c = 33.619(2) Å, α = 90°, β = 90°, γ = 90°, V = 10780.2(13) $Å^3$, Z = 8, T = 150(1) K, CuK\α = 1.5418 Å, Dcalc = 1.176 g/cm^3, 28580 reflections measured (6.972 ≤ 2Θ ≤ 133.176), 9503 unique (R_{int} = 0.0575, R_{sigma} = 0.0537) which were used in all calculations. The final R_1 was 0.0867 (I > 2σ(I)) and wR_2 was 0.2843 (all data). Internal angle of dimerization = 180.0 (7)°.

Table S10 – Hydrogen bond distances and angles observed for **10**, calculated from the single crystal X-ray structure shown in Figure S104.

Hydrogen bond donor	Hydrogen atom	Hydrogen bond acceptor	Hydrogen bond length (D•••A) (Å)	Hydrogen bond angle (D-H•••A) (°)
N1	H1	O2	2.775 (5)	160.8 (3)
N2	H2	O2	3.094 (5)	142.0 (3)
O6	H6B	O1	2.909 (5)	175.2 (3)
O6	H6A	O5	2.781 (6)	172.3 (3)
O7	H7A	O5	2.767 (5)	175.9 (5)
O7	H7B	O8	2.981 (4)	152.5 (3)
O8	H8B	O4	2.753 (6)	159.2 (4)
O9	H9B	O5	2.767 (5)	175.9 (3)
O11	H11D	O3	2.834 (7)	146.9 (4)
O11	H11C	O10	2.803 (10)	150.8 (4)
O12	H12A	O4	2.754 (6)	169.2 (3)
O13	H13C	O3	2.717 (7)	158.1 (5)

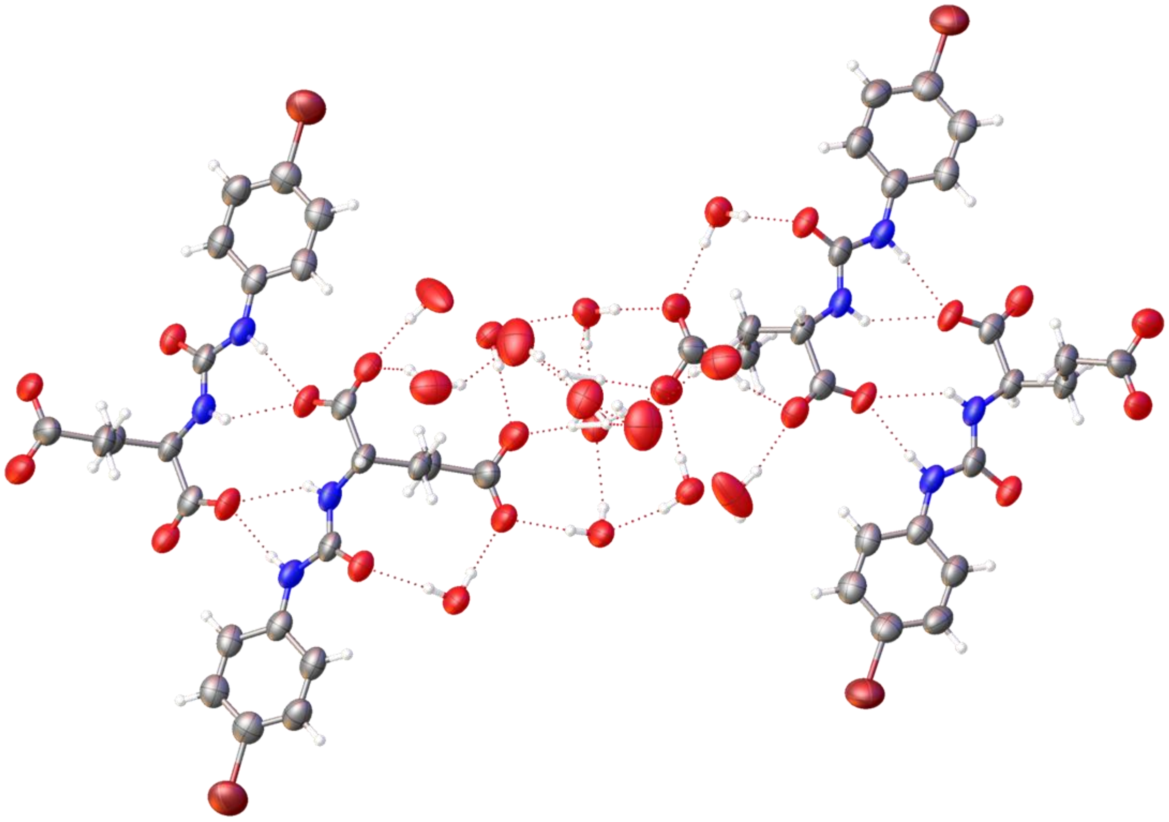

Figure S105 – Single crystal X-ray showing the extended hydrogen bonded network of **10**: red = oxygen; purple = bromine; blue = nitrogen; white = hydrogen; grey = carbon. TBA counter cations have been omitted for clarity.

Section 14: Molecular modelling and simulation

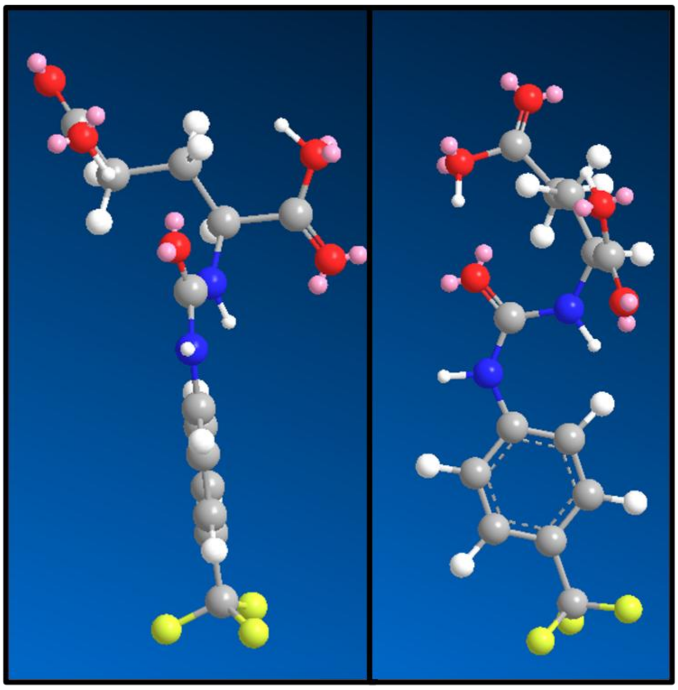

Figure S106 – MM2 energy minimised ball and stick 3D model used to calculate the molecular properties of **1** (Table 11).

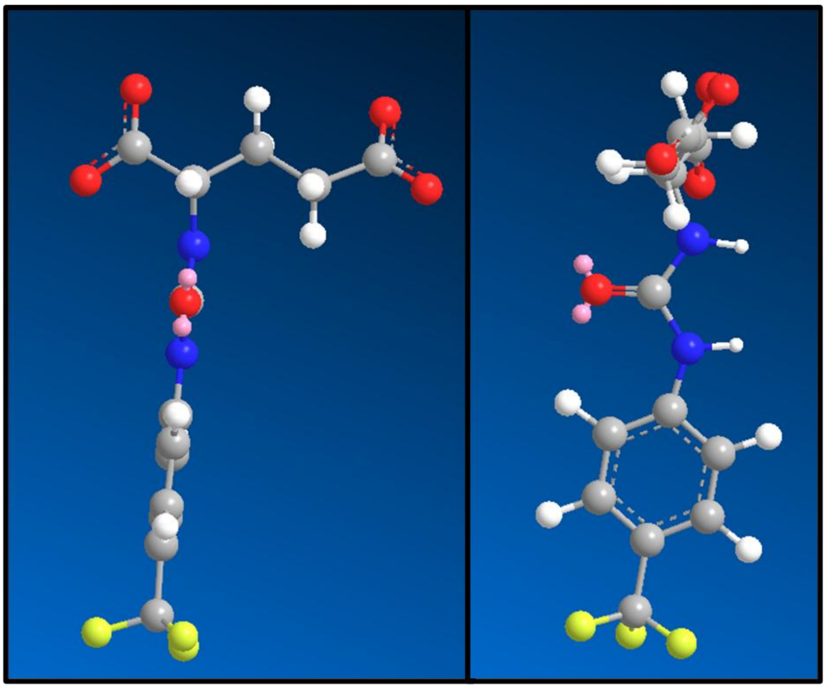

Figure S107 – MM2 energy minimised ball and stick 3D model used to calculate the molecular properties of **2** (Table 11).

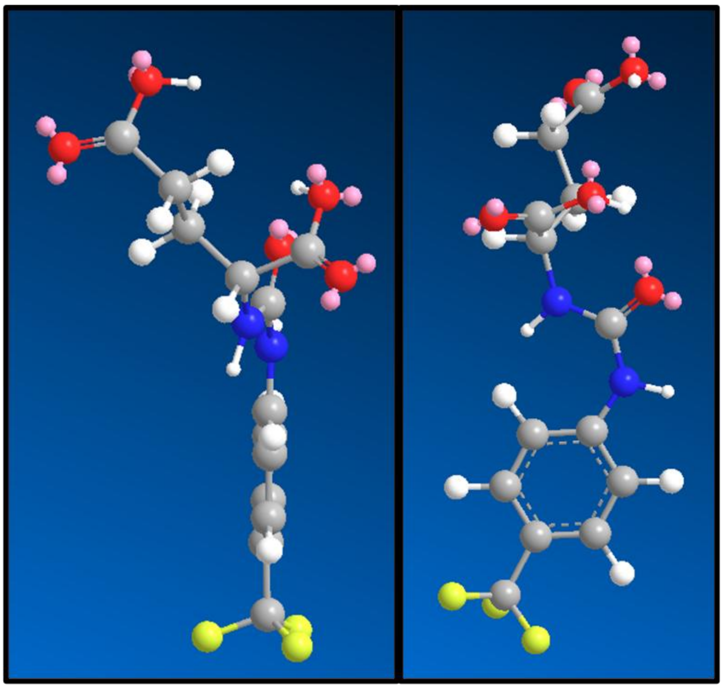

Figure S108 – MM2 energy minimised ball and stick 3D model used to calculate the molecular properties of **3** (Table 11).

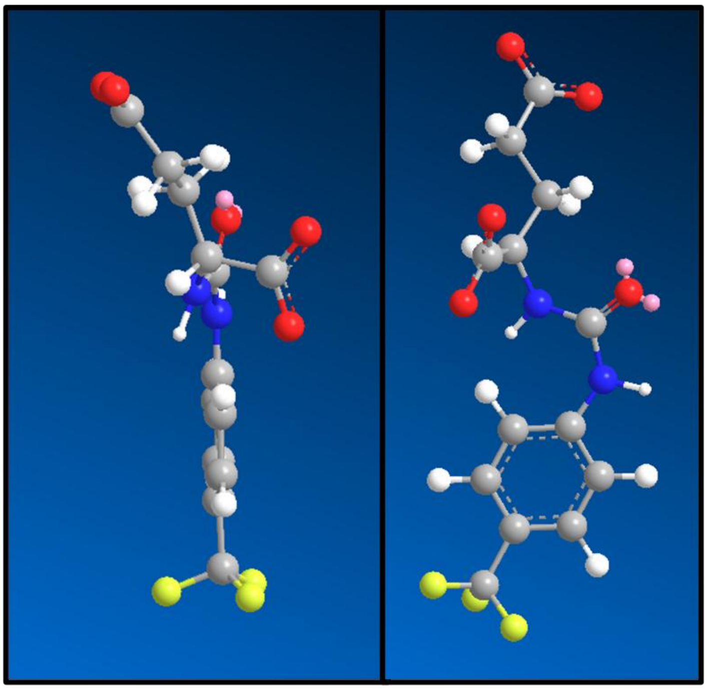

Figure S109 – MM2 energy minimised ball and stick 3D model used to calculate the molecular properties of **4** (Table 11).

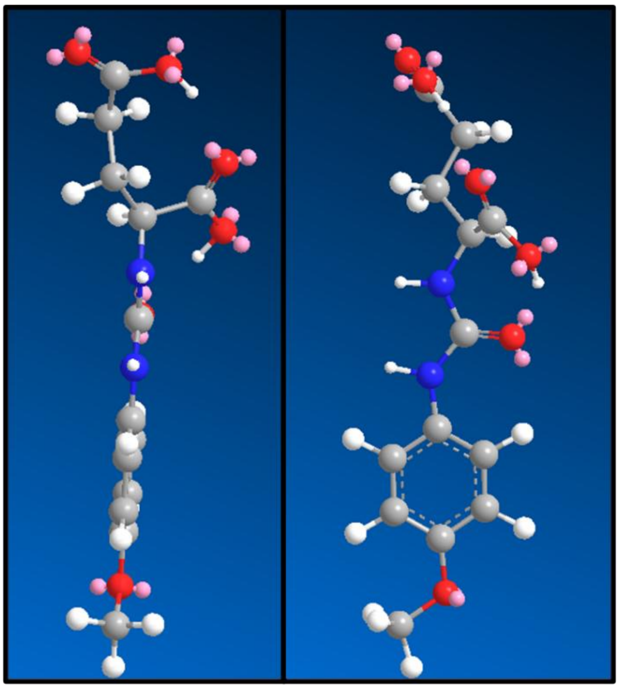

Figure S110 – MM2 energy minimised ball and stick 3D model used to calculate the molecular properties of **5** (Table 11).

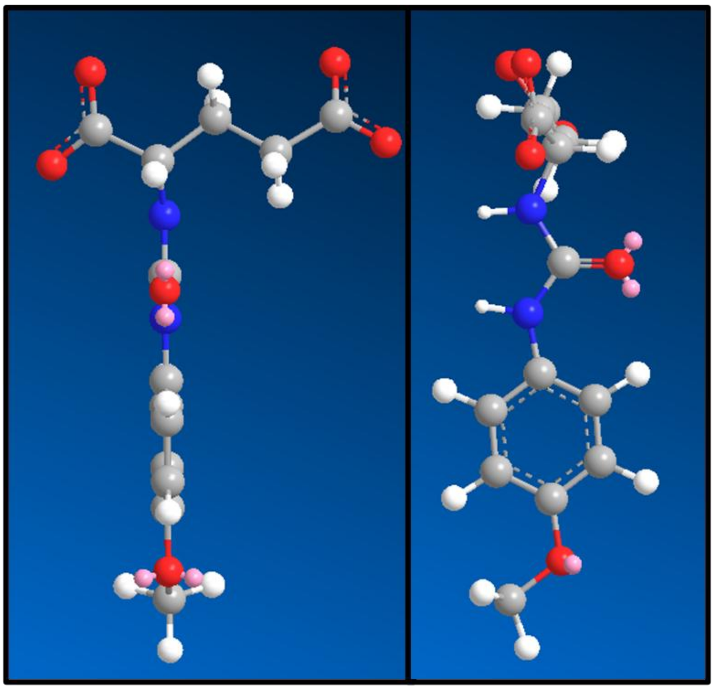

Figure S111 – MM2 energy minimised ball and stick 3D model used to calculate the molecular properties of **6** (Table 11).

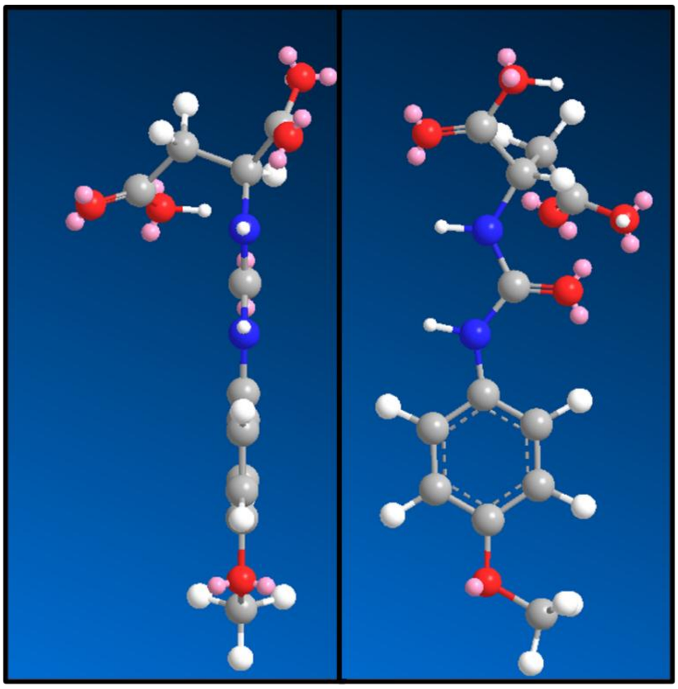

Figure S112 – MM2 energy minimised ball and stick 3D model used to calculate the molecular properties of **7** (Table 11).

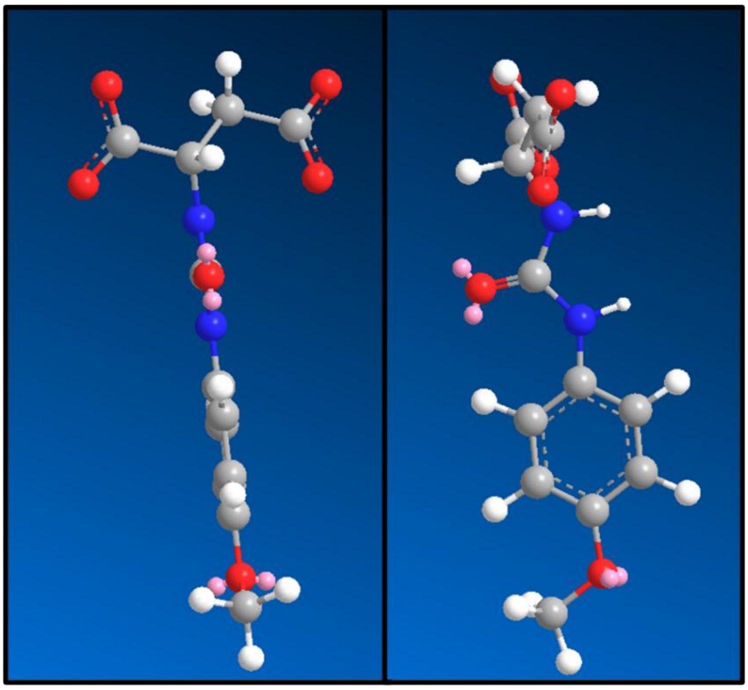

Figure S113 – MM2 energy minimised ball and stick 3D model used to calculate the molecular properties of **8** (Table 11).

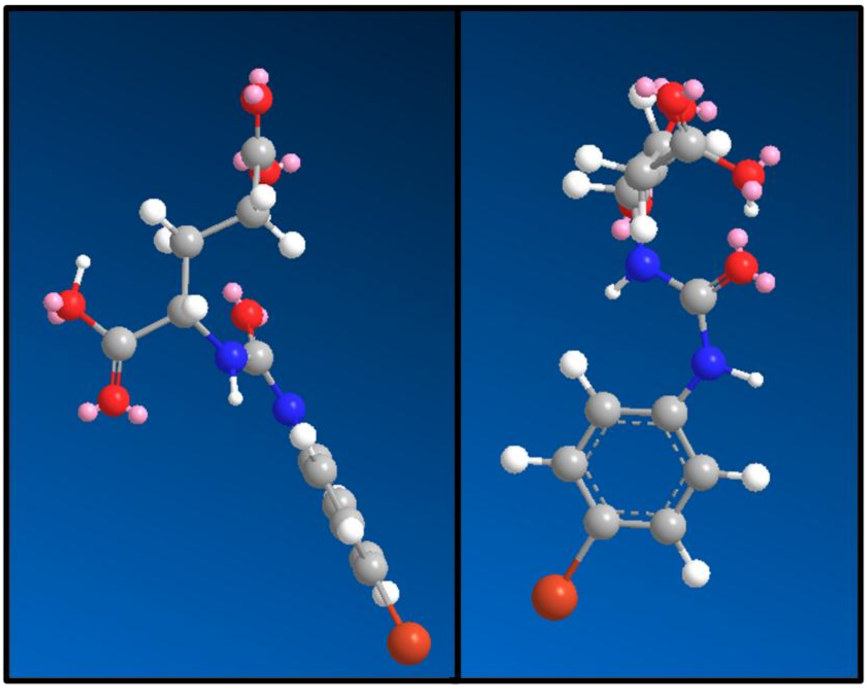

Figure S114 – MM2 energy minimised ball and stick 3D model used to calculate the molecular properties of **9** (Table 11).

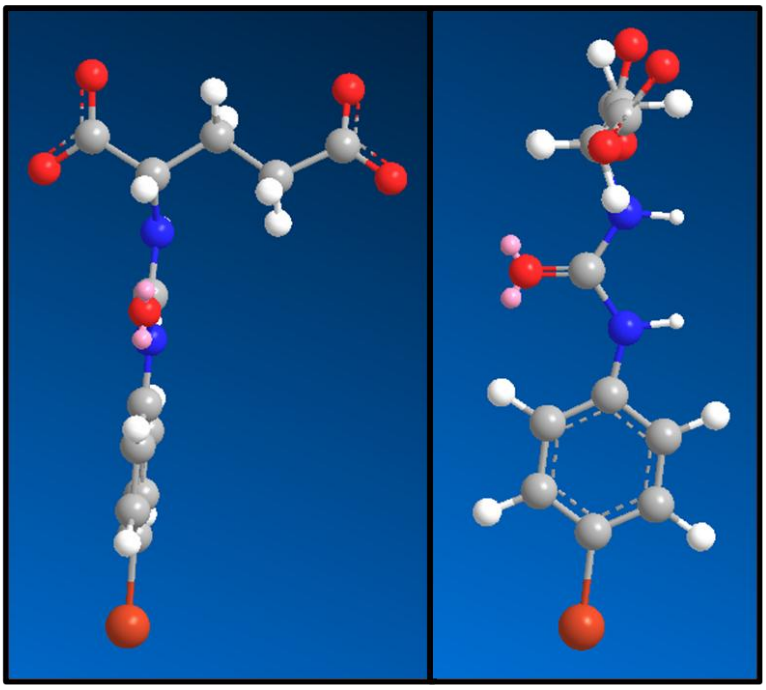

Figure S115 – MM2 energy minimised ball and stick 3D model used to calculate the molecular properties of **10** (Table 11).

Table S11 - Summary of molecular properties generated using MM2 energy minimised ball and stick 3D models developed using Chem3D (version 22.2.0) for 1, **3**, **5**, **7** and **9** the anionic component of **2**, **4**, **6**, **8** and **10**.

SSA	Length (Å)	Length (nm)	Dipole-dipole energy (kcal/mol)	Total energy (kcal/mol)
**1**	12.308	1.2308	-8.6390	8.9924
**2**	12.595	1.2595	-10.5635	28.1223
**3**	13.097	1.3097	-7.7741	7.7305
**4**	13.668	1.3668	-10.0452	35.5194
**5**	15.184	1.5184	-11.3716	4.4756
**6**	11.575	1.1575	-10.7255	23.9925
**7**	11.807	1.1807	-5.9275	9.6035
**8**	12.823	1.2823	-9.8860	33.5566
**9**	11.899	1.1899	-8.4910	4.3176
**10**	11.470	1.1470	-10.4641	23.5809

Calculation of LogP values using Swiss ADME

Table S12 – Summary of LogP values calculated for **1**, **3**, **5**, **7** and **9** using Swiss ADME.[5]

SSA	iLOGP	XLOGP3	WLOGP	MLOGP	Silicos-IT Log P	Consensus Log P
**1**	1.33	1.29	3.11	1.75	1.02	1.70
**3**	1.47	1.29	3.11	1.75	1.02	1.73
**5**	1.44	0.38	0.94	0.57	-0.06	0.65
**7**	1.00	0.02	0.55	0.29	-0.45	0.28
**9**	1.59	1.10	1.70	1.49	0.56	1.29

Table S13 – Summary of LogP values calculated for the anionic component of **2**, **4**, **6**, **8** and **10** using Swiss ADME.[5]

SSA	iLOGP	XLOGP3	WLOGP	MLOGP	Silicos-IT Log P	Consensus Log P
**2**	1.35	1.29	0.44	1.75	1.02	1.17
**4**	1.39	1.29	0.44	1.75	1.02	1.18
**6**	1.42	0.38	-1.73	0.57	-0.06	0.11
**8**	1.21	0.02	-2.12	0.29	-0.45	-0.21
**10**	1.67	1.10	-0.97	1.49	0.56	0.77

Section 15: *In vitro* Drug Metabolism and Pharmokinetics (DMPK) studies

Protein Binding Measurements in Human Plasma by Using Equilibrium Dialysis

Table S14 - Protein binding results of **1**, **2**, **3**, **9** and control compound in human plasma.

Compound	Species	% Bound	% Unbound	% Recovery	% Remaining at 6 hr
Ketoconazole	Human	99.14	0.86	96.60	101.17
SSA **1**	Human	78.31	21.69	97.28	104.11
SSA **2**	Human	78.73	21.27	97.50	108.64
SSA **3**	Human	76.97	23.03	99.61	101.82
SSA **9**	Human	83.37	16.63	94.88	105.81

Kinetic Solubility Determination in PBS pH 7.4

Table S15 - The solubility data of **1**, **2**, **3**, **9** and control compounds in PBS pH 7.4. The upper limit was set at 300 µM. Any value close to or above 300 µM indicates that the compound may have a solubility at or above 300 µM.

Compound	Solubility in PBS pH 7.4 (µM)
Progesterone	14.52
Diclofenac	280.79
SSA **1**	303.17
SSA **2**	297.29
SSA **3**	309.81
SSA **9**	304.92

Metabolic Stability in Rat and Mouse Liver Microsomes

Table S16 - Metabolic stability of **1**, **2**, **3**, **9** and control compounds in rat and mouse liver microsomes.

**Compound**	**Species**	***in vitro*** **$t_{1/2}$ (min)**	***in vitro*** **CL_{int} (µL/min/mg)**	**Scale-up CL_{int} (mL/min/Kg)**	**Predicted Hepatic CL_H (mL/min/kg)**	**Hepatic Extraction Ratio (ER)**
Verapamil	Rat	2.40	577.23	1408.44	64.87	0.95
	Mouse	1.96	708.93	2932.13	87.32	0.97
SSA **1**	Rat	> 184.78	< 7.50	< 18.30	< 14.42	< 0.21
	Mouse	> 184.78	< 7.50	< 31.02	< 23.07	< 0.26
SSA **2**	Rat	> 184.78	< 7.50	< 18.30	< 14.42	< 0.21
	Mouse	> 184.78	< 7.50	< 31.02	< 23.07	< 0.26
SSA **3**	Rat	> 184.78	< 7.50	< 18.30	< 14.42	< 0.21
	Mouse	> 184.78	< 7.50	< 31.02	< 23.07	< 0.26
SSA **9**	Rat	> 184.78	< 7.50	< 18.30	< 14.42	< 0.21
	Mouse	> 184.78	< 7.50	< 31.02	< 23.07	< 0.26

Table S17 - Remaining percentage of **1**, **2**, **3**, **9** and control compounds in rat and mouse liver microsomes

**Compound**	**Species**	**Assay format**	**Remaining percentage (%)**				
			**0.5 min**	**5 min**	**15 min**	**30 min**	**60 min**
Verapamil	Rat	+Cofactors	100.00	27.30	3.04	BLOD	BLOD
		-Cofactors	100.00	-	-	-	99.51
	Mouse	+Cofactors	100.00	20.30	2.54	BLOD	BLOD
		-Cofactors	100.00	-	-	-	103.42
SSA **1**	Rat	+Cofactors	100.00	96.56	99.83	96.48	95.66
		-Cofactors	100.00	-	-	-	102.14
	Mouse	+Cofactors	100.00	100.09	98.69	98.11	105.07
		-Cofactors	100.00	-	-	-	103.10
SSA **2**	Rat	+Cofactors	100.00	95.53	94.28	97.57	96.64
		-Cofactors	100.00	-	-	-	97.74
	Mouse	+Cofactors	100.00	101.97	102.46	101.55	102.31
		-Cofactors	100.00	-	-	-	102.89
SSA **3**	Rat	+Cofactors	100.00	95.07	99.07	97.86	98.31
		-Cofactors	100.00	-	-	-	104.13
	Mouse	+Cofactors	100.00	99.16	100.05	98.24	98.27
		-Cofactors	100.00	-	-	-	93.98
SSA **9**	Rat	+Cofactors	100.00	97.95	96.49	99.04	100.06
		-Cofactors	100.00	-	-	-	102.63
	Mouse	+Cofactors	100.00	101.88	100.09	97.61	103.16
		-Cofactors	100.00	-	-	-	103.83

Bidirectional Permeability in Caco-2 Cell Line

Table S18 - Permeability results of **1**, **2**, **3**, **9** and control compounds in the Caco-2 cell line.

Compound	$P_{app\ (A\text{-}B)}$ (10^{-6}, cm/s)	$P_{app\ (B\text{-}A)}$ (10^{-6}, cm/s)	Efflux Ratio	Recovery (%) AP-BL	Recovery (%) BL-AP
Metoprolol	28.43	26.41	0.93	107.79	98.96
Digoxin	0.42	18.74	45.59	95.92	99.20
SSA **1**	0.19	0.23	1.23	107.47	89.73
SSA **2**	0.14	0.24	1.71	99.71	80.26
SSA **3**	0.10	0.24	2.31	102.70	87.42
SSA **9**	0.17	0.32	1.93	115.47	106.39

Table S19 - The assessment of Caco-2 cell monolayer integrity of **1**, **2**, **3**, **9** and control compounds.

Compound	TEER A-B (Ω × cm^2)	TEER B-A (Ω × cm^2)	LY Leakage A-B (%)	LY Leakage B-A (%)
Metoprolol	751.82	821.75	0.13	0.15
Digoxin	732.02	768.48	0.14	0.15
SSA **1**	668.24	744.74	0.14	0.13
SSA **2**	674.67	784.93	0.14	0.16
SSA **3**	731.59	732.88	0.13	0.14
SSA **9**	759.97	796.87	0.14	0.15

Section 16: Antimicrobial activity

Table S20 - The type and strain of bacteria used to test the antimicrobial efficacy of SSAs **1** - **10**.

Type of bacteria	Bacterial strain
Gram negative	PAO1 (*P.aeruginosa*)
	M6 (*K. pneumoniae*)
	NCTC 12923 (*E. coli*)
	ATCC 17978 (*A. baumannii*)
Gram positive	ATCC 9144 (*S. aureus*)
	NCTC 775 (*E. faecalis*)
	NCTC 12204 (*E. faecium*)

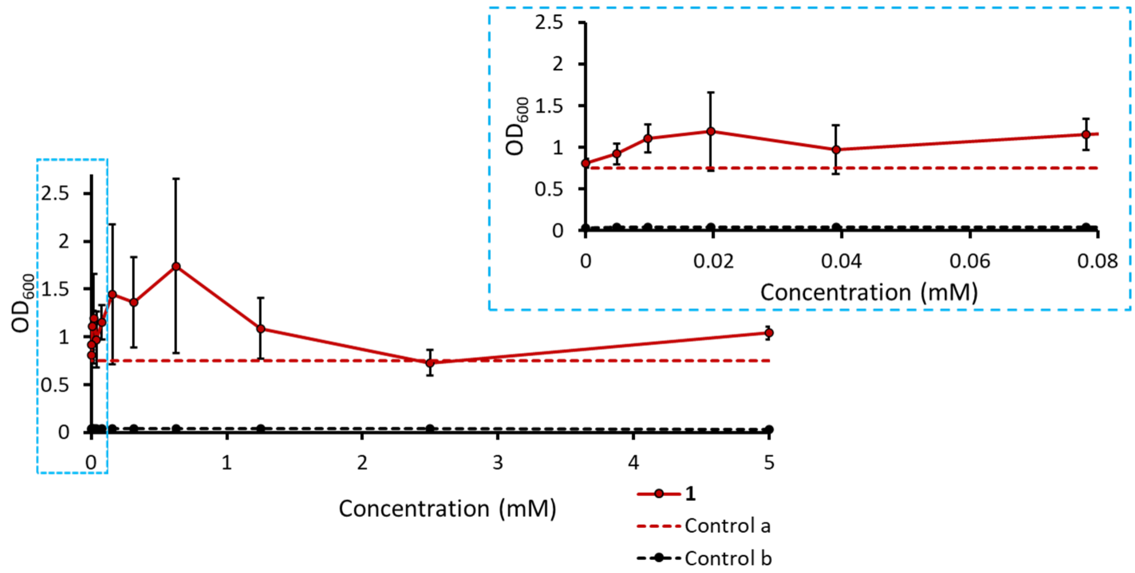

Figure S116 – OD_{600} readings of **1** (solid red line) at increasing concentrations in the presence of PAO1 (*P.aeruginosa*), created from an average of two biological repeats, each containing three technical repeats. Control a (red dashed line) = absence of SSA, Control b (black dashed line) = absence of bacteria, outlined in blue dashed line = enlarged area.

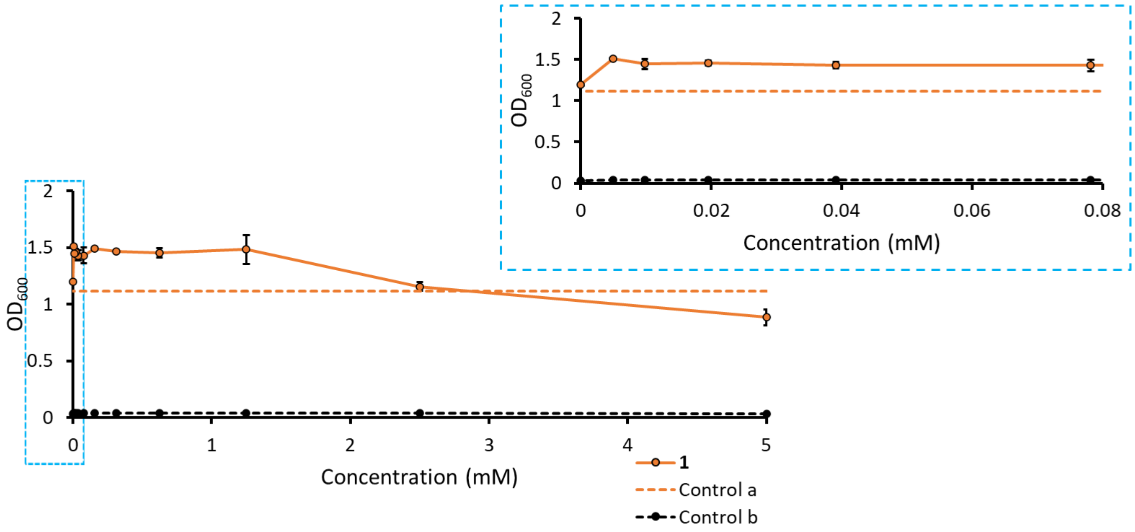

Figure S117 – OD_{600} readings of **1** (solid orange line) at increasing concentrations in the presence of M6 (*K. pneumoniae*), created from an average of two biological repeats, each containing three technical repeats. Control a (orange dashed line) = absence of SSA, Control b (black dashed line) = absence of bacteria, outlined in blue dashed line = enlarged area.

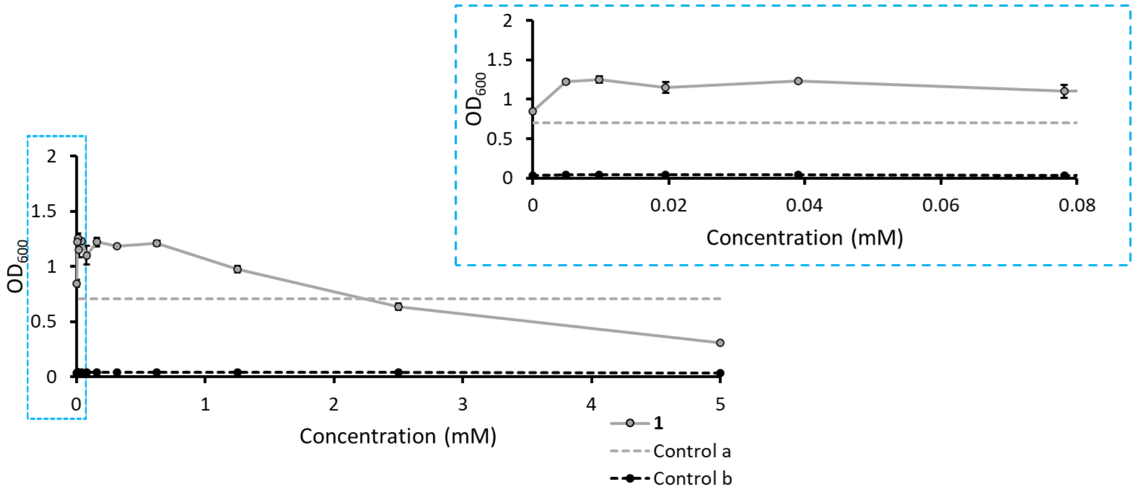

Figure S118 – OD600 readings of **1** (solid grey line) at increasing concentrations in the presence of NCTC 12923 (*E. coli*), created from an average of two biological repeats, each containing three technical repeats. Control a (grey dashed line) = absence of SSA, Control b (black dashed line) = absence of bacteria, outlined in blue dashed line = enlarged area.

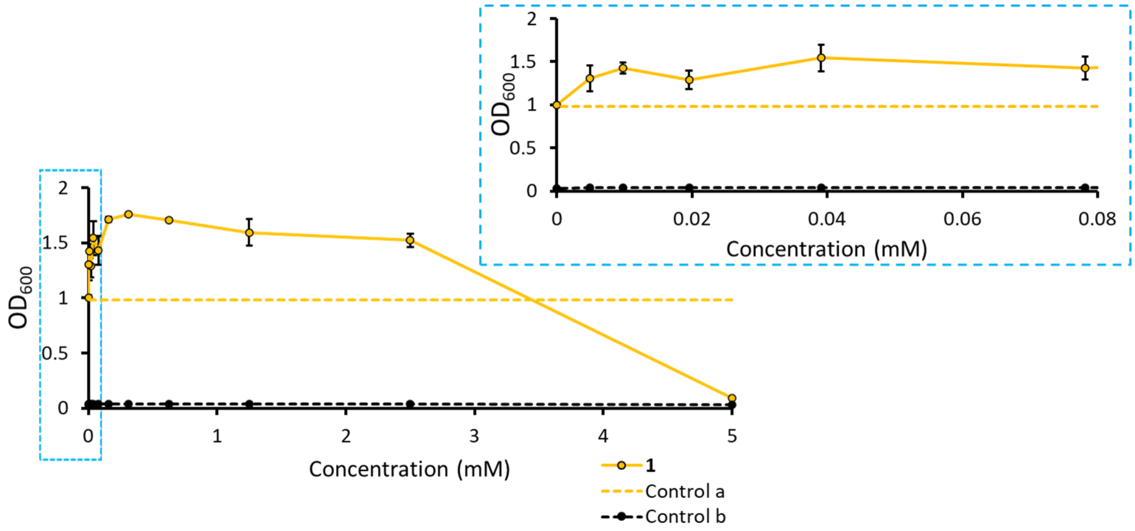

Figure S119 – OD600 readings of **1** (solid yellow line) at increasing concentrations in the presence of ATCC 17978 (*A. baumannii*), created from an average of two biological repeats, each containing three technical repeats. Control a (yellow dashed line) = absence of SSA, Control b (black dashed line) = absence of bacteria, outlined in blue dashed line = enlarged area.

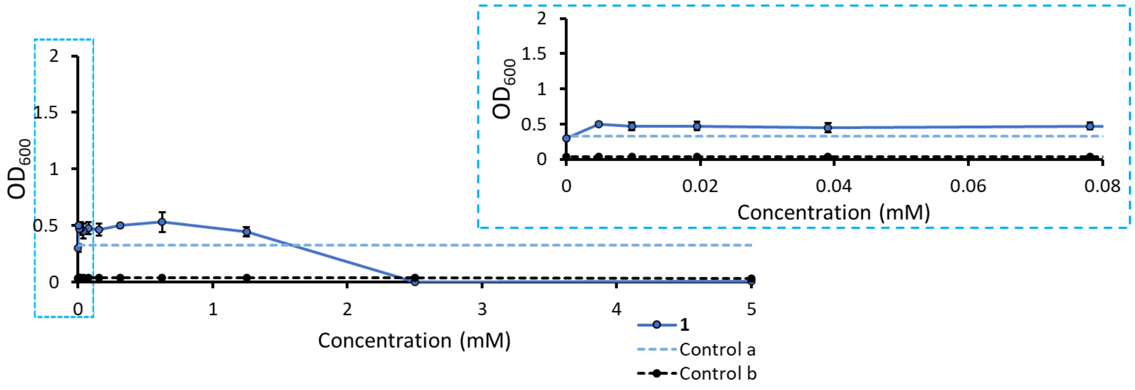

Figure S120 – OD_{600} readings of **1** (solid blue line) at increasing concentrations in the presence of ATCC 9144 (*S. aureus*), created from an average of two biological repeats, each containing three technical repeats. Control a (blue dashed line) = absence of SSA, Control b (black dashed line) = absence of bacteria, outlined in light blue dashed line = enlarged area.

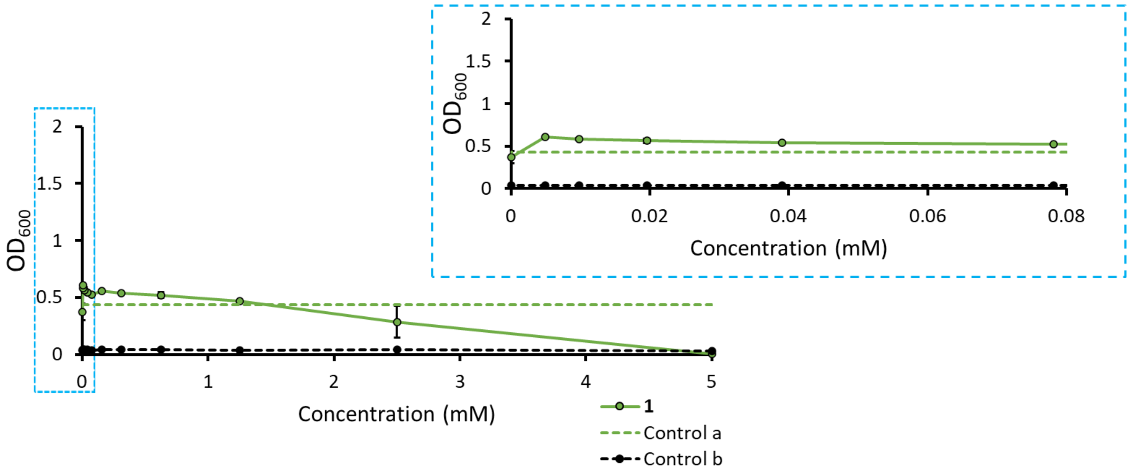

Figure S121 – OD_{600} readings of **1** (solid green line) at increasing concentrations in the presence of NCTC 775 (*E. faecalis*), created from an average of two biological repeats, each containing three technical repeats. Control a (green dashed line) = absence of SSA, Control b (black dashed line) = absence of bacteria, outlined in blue dashed line = enlarged area.

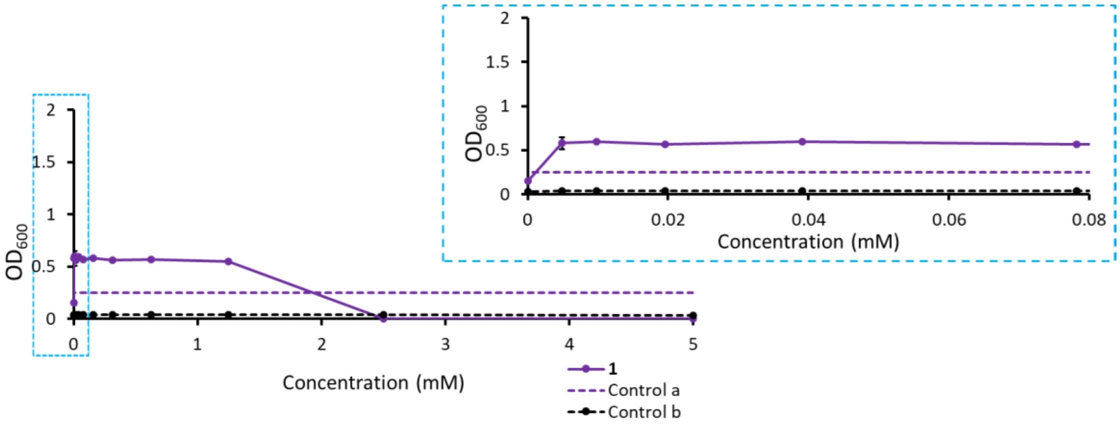

Figure S122 – OD_{600} readings of **1** (solid purple line) at increasing concentrations in the presence of NCTC 12204 (*E. faecium*), created from an average of two biological repeats, each containing three technical repeats. Control a (purple dashed line) = absence of SSA, Control b (black dashed line) = absence of bacteria, outlined in blue dashed line = enlarged area.

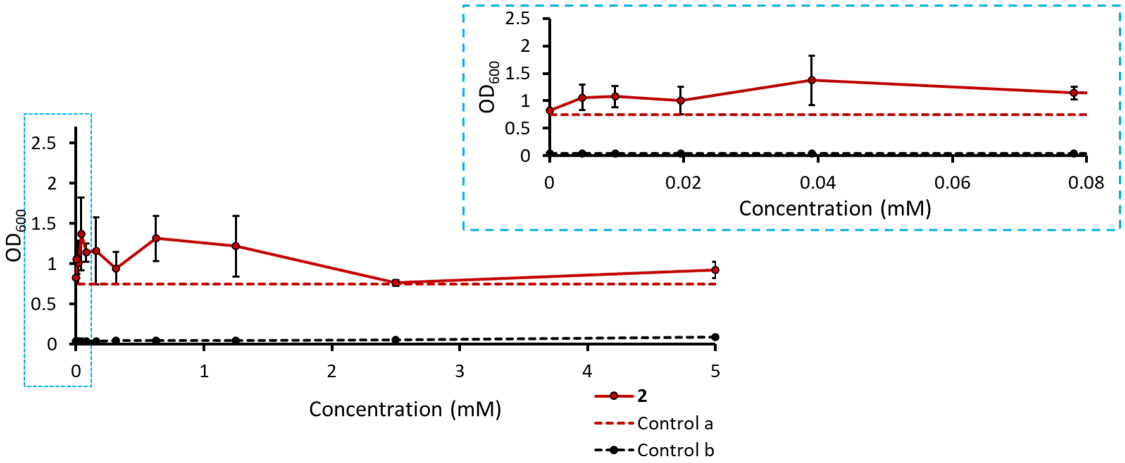

Figure S123 – OD_{600} readings of **2** (solid red line) at increasing concentrations in the presence of PAO1 (*P.aeruginosa*), created from an average of two biological repeats, each containing three technical repeats. Control a (red dashed line) = absence of SSA, Control b (black dashed line) = absence of bacteria, outlined in blue dashed line = enlarged area.

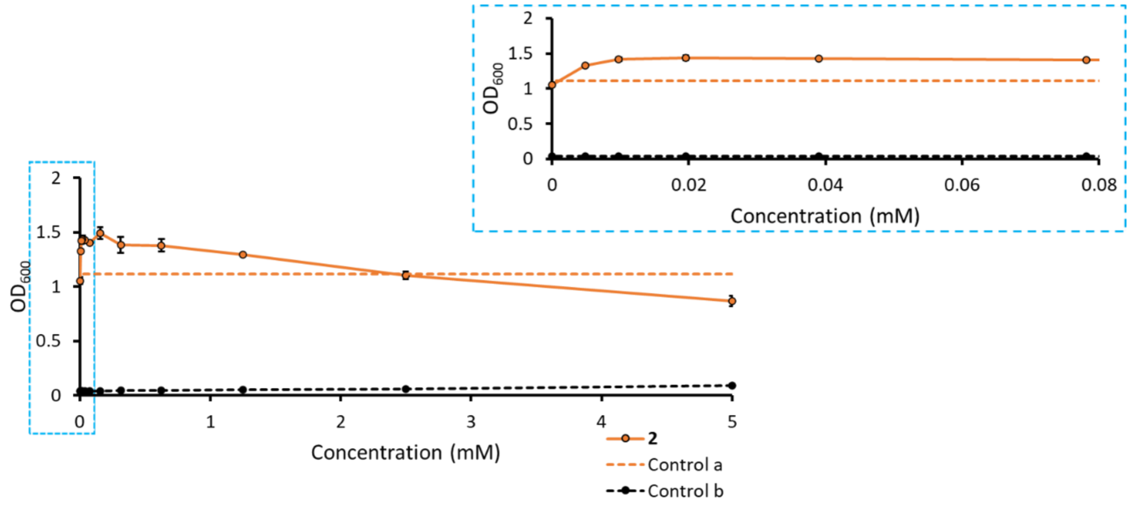

Figure S124 – OD_{600} readings of **2** (solid orange line) at increasing concentrations in the presence of M6 (*K. pneumoniae*), created from an average of two biological repeats, each containing three technical repeats. Control a (orange dashed line) = absence of SSA, Control b (black dashed line) = absence of bacteria, outlined in blue dashed line = enlarged area.

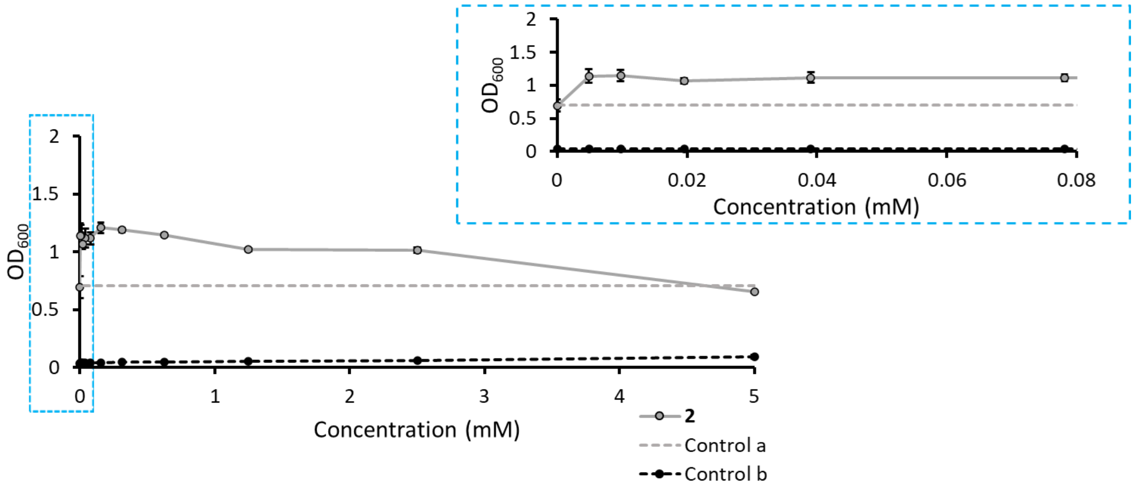

Figure S125 – OD_{600} readings of **2** (solid grey line) at increasing concentrations in the presence of NCTC 12923 (*E. coli*), created from an average of two biological repeats, each containing three technical repeats. Control a (grey dashed line) = absence of SSA, Control b (black dashed line) = absence of bacteria, outlined in blue dashed line = enlarged area.

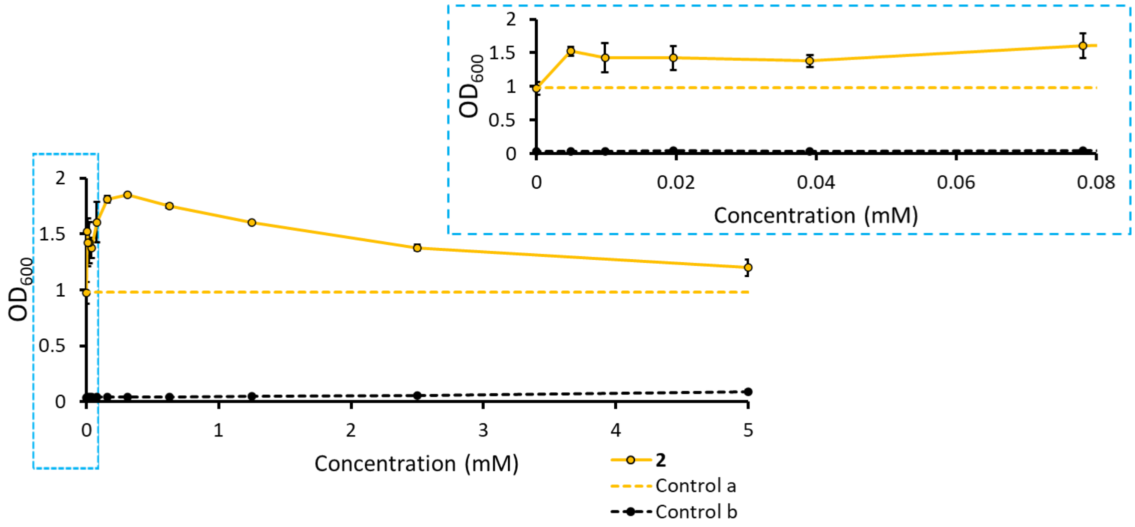

Figure S126 – OD_{600} readings of **2** (solid yellow line) at increasing concentrations in the presence of ATCC 17978 (*A. baumannii*), created from an average of two biological repeats, each containing three technical repeats. Control a (yellow dashed line) = absence of SSA, Control b (black dashed line) = absence of bacteria, outlined in blue dashed line = enlarged area.

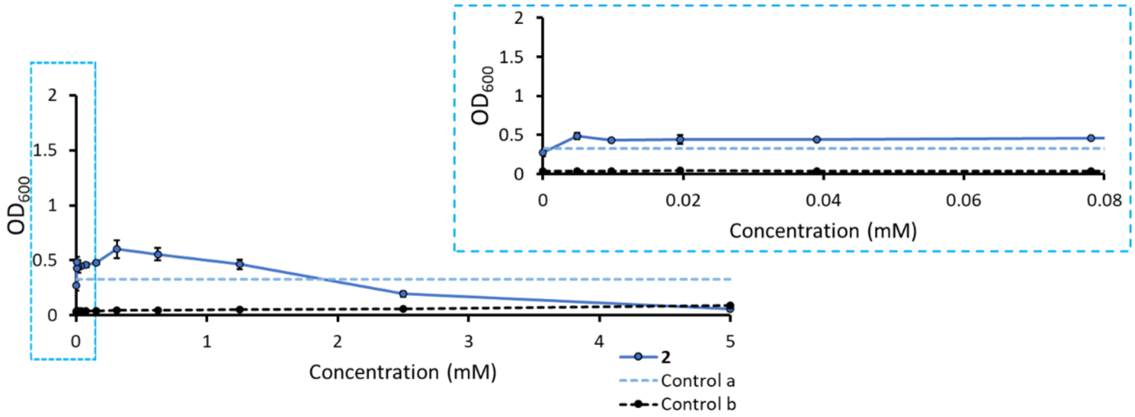

Figure S127 – OD_{600} readings of 2 (solid blue line) at increasing concentrations in the presence of ATCC 9144 (*S. aureus*), created from an average of two biological repeats, each containing three technical repeats. Control a (blue dashed line) = absence of SSA, Control b (black dashed line) = absence of bacteria, outlined in light blue dashed line = enlarged area.

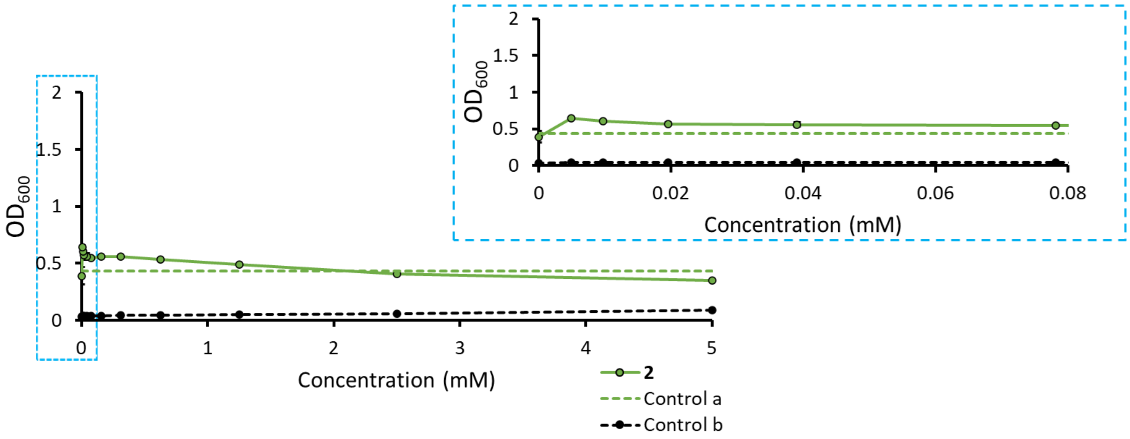

Figure S128 – OD_{600} readings of **2** (solid green line) at increasing concentrations in the presence of NCTC 775 (*E. faecalis*), created from an average of two biological repeats, each containing three technical repeats. Control a (green dashed line) = absence of SSA, Control b (black dashed line) = absence of bacteria, outlined in blue dashed line = enlarged area.

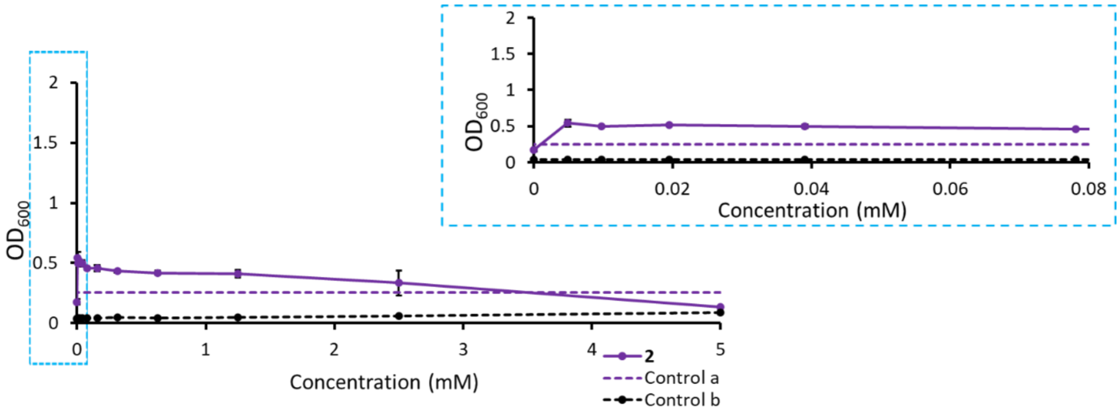

Figure S129 – OD_{600} readings of **2** (solid purple line) at increasing concentrations in the presence of NCTC 12204 (*E. faecium*), created from an average of two biological repeats, each containing three technical repeats. Control a (purple dashed line) = absence of SSA, Control b (black dashed line) = absence of bacteria, outlined in blue dashed line = enlarged area.

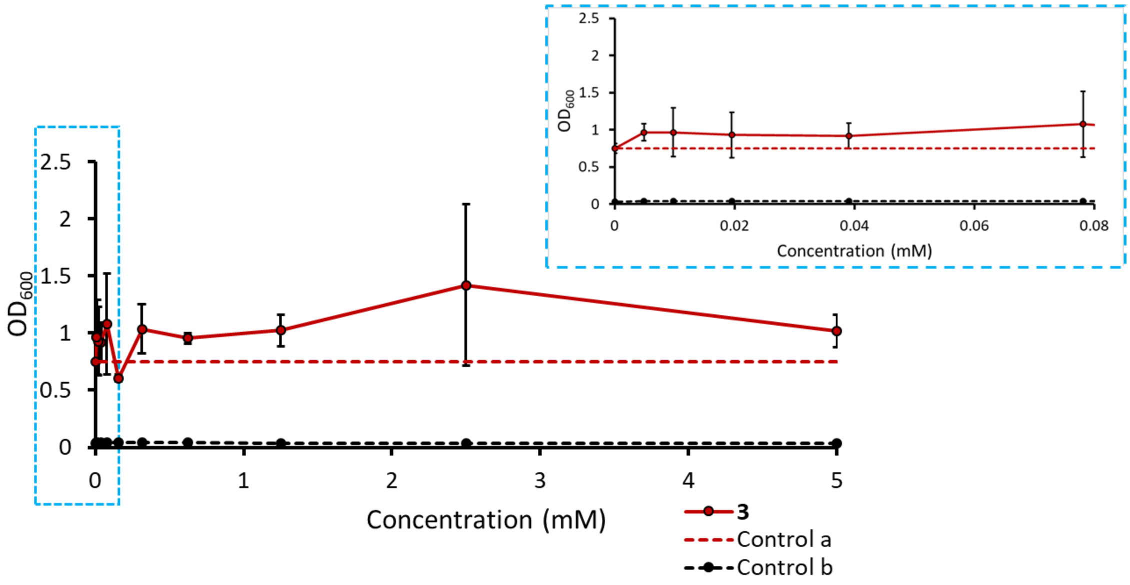

Figure S130 – OD_{600} readings of **3** (solid red line) at increasing concentrations in the presence of PAO1 (*P.aeruginosa*), created from an average of two biological repeats, each containing three technical repeats. Control a (red dashed line) = absence of SSA, Control b (black dashed line) = absence of bacteria, outlined in blue dashed line = enlarged area.

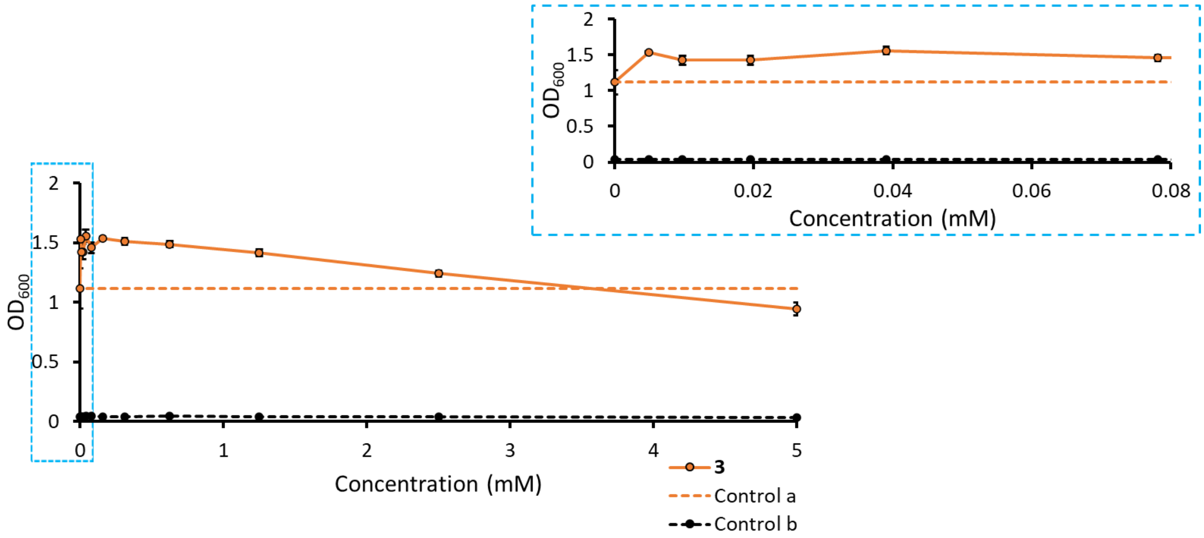

Figure S131 – OD_{600} readings of **3** (solid orange line) at increasing concentrations in the presence of M6 (*K. pneumoniae*), created from an average of two biological repeats, each containing three technical repeats. Control a (orange dashed line) = absence of SSA, Control b (black dashed line) = absence of bacteria, outlined in blue dashed line = enlarged area.

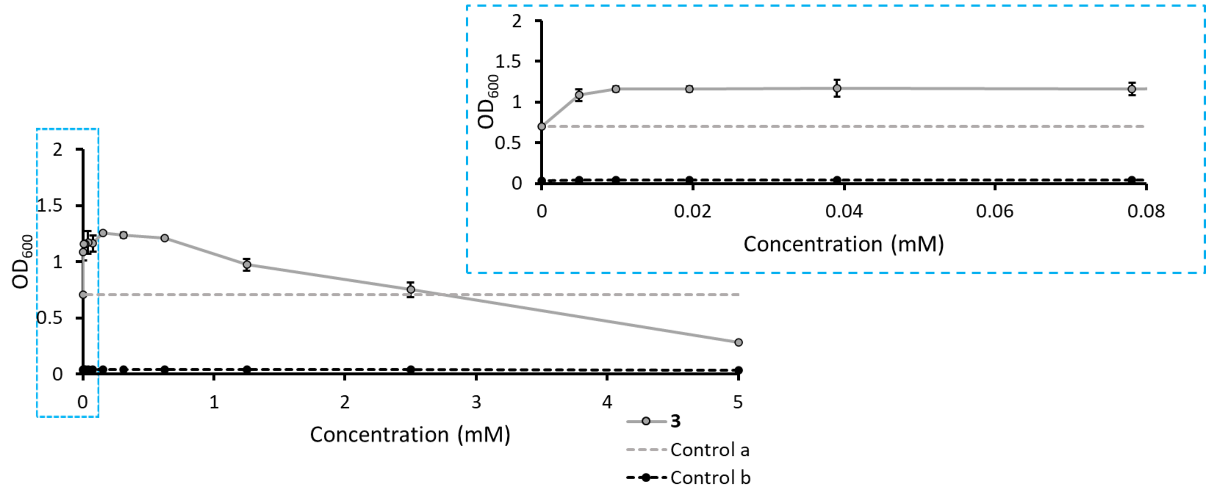

Figure S132 – OD_{600} readings of **3** (solid grey line) at increasing concentrations in the presence of NCTC 12923 (*E. coli*), created from an average of two biological repeats, each containing three technical repeats. Control a (grey dashed line) = absence of SSA Control b (black dashed line) = absence of bacteria, outlined in blue dashed line = enlarged area.

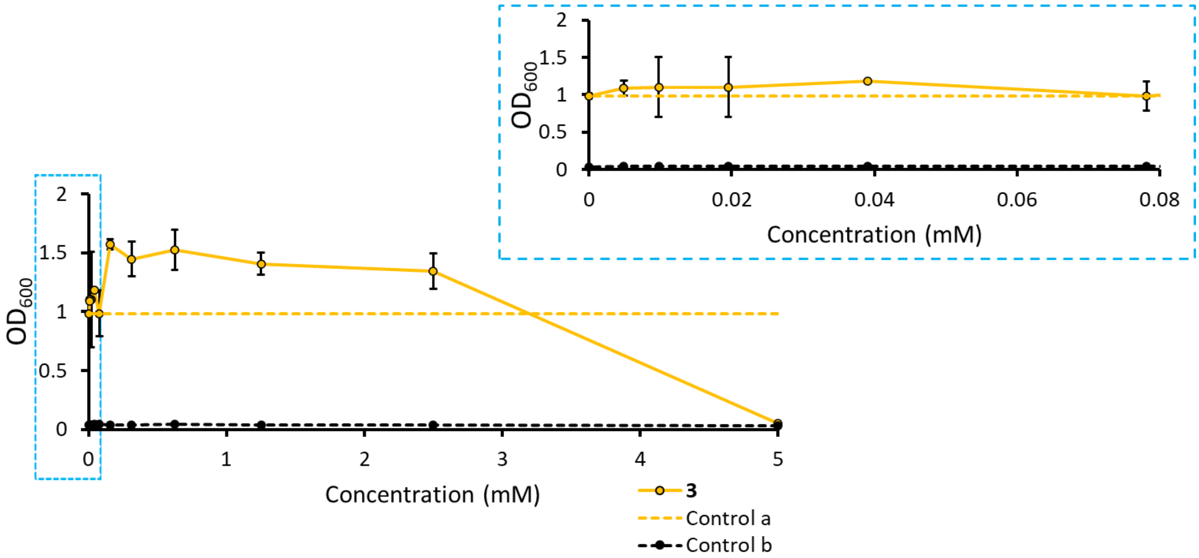

Figure S133 – OD_{600} readings of **3** (solid yellow line) at increasing concentrations in the presence of ATCC 17978 (*A. baumannii*), created from an average of two biological repeats, each containing three technical repeats. Control a (yellow dashed line) = absence of SSA, Control b (black dashed line) = absence of bacteria, outlined in blue dashed line = enlarged area.

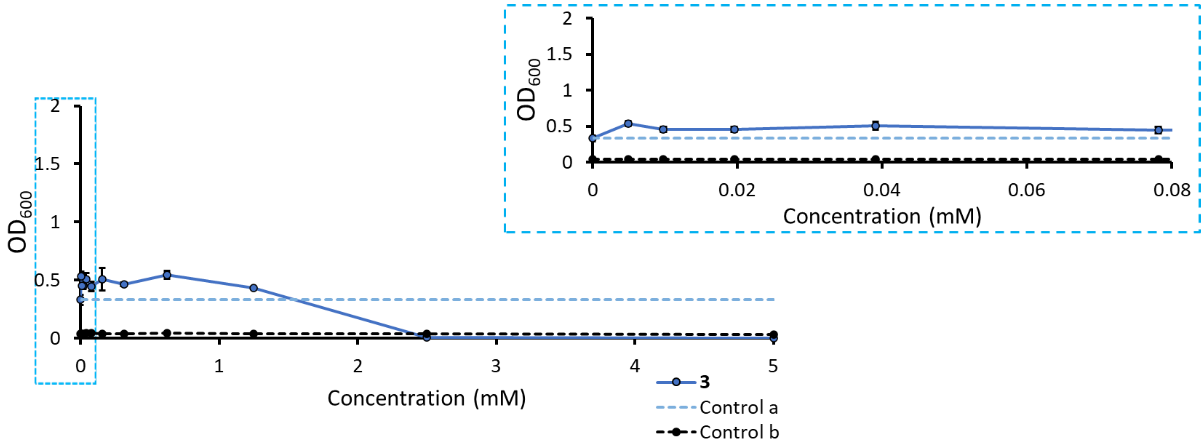

Figure S134 – OD_{600} readings of **3** (solid blue line) at increasing concentrations in the presence of ATCC 9144 (*S. aureus*), created from an average of two biological repeats, each containing three technical repeats. Control a (blue dashed line) = absence of SSA, Control b (black dashed line) = absence of bacteria, outlined in light blue dashed line = enlarged area.

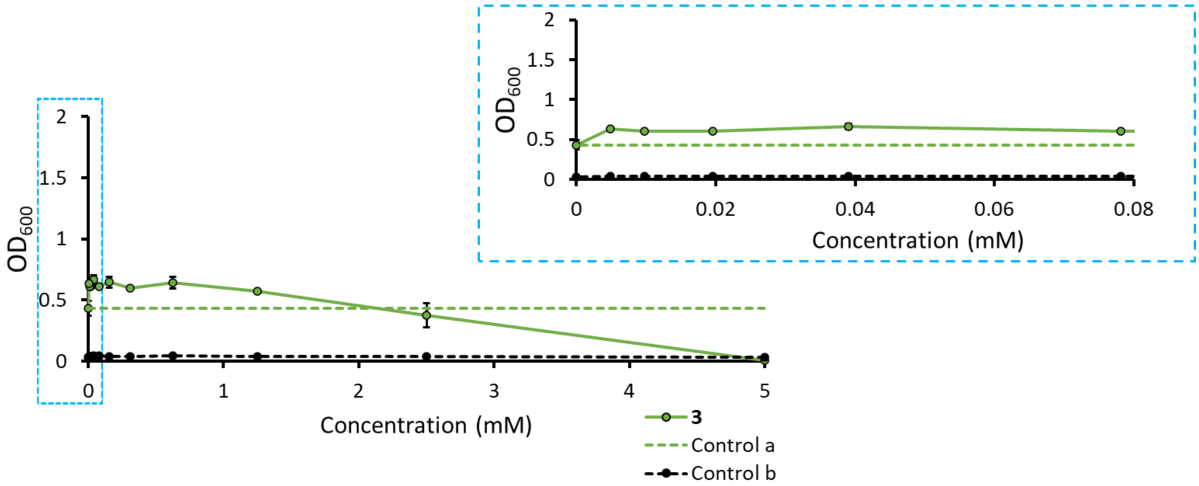

Figure S135 – OD_{600} readings of **3** (solid green line) at increasing concentrations in the presence of NCTC 775 (*E. faecalis*), created from an average of two biological repeats, each containing three technical repeats. Control a (green dashed line) = absence of SSA, Control b (black dashed line) = absence of bacteria, outlined in blue dashed line = enlarged area.

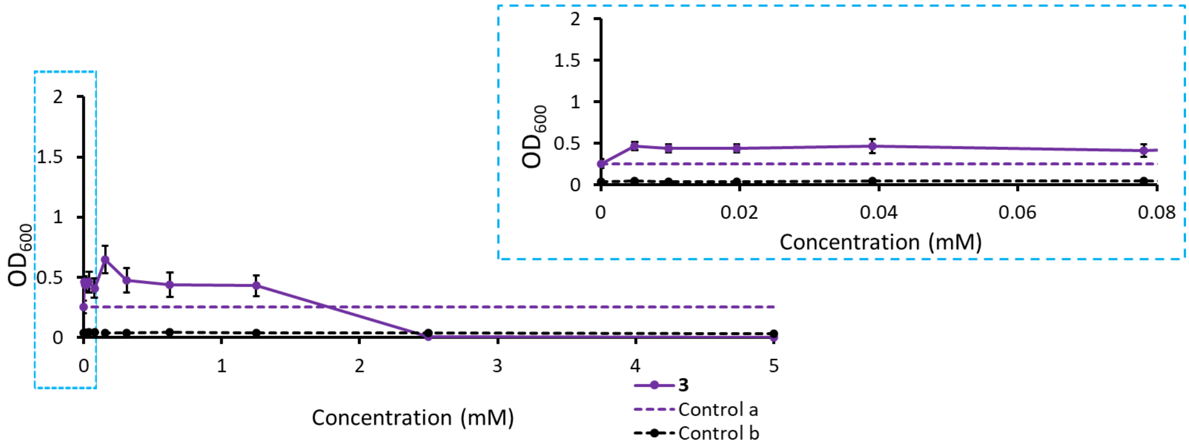

Figure S136 – OD_{600} readings of **3** (solid purple line) at increasing concentrations in the presence of NCTC 12204 (*E. faecium*), created from an average of two biological repeats, each containing three technical repeats. Control a (purple dashed line) = absence of SSA, Control b (black dashed line) = absence of bacteria, outlined in blue dashed line = enlarged area.

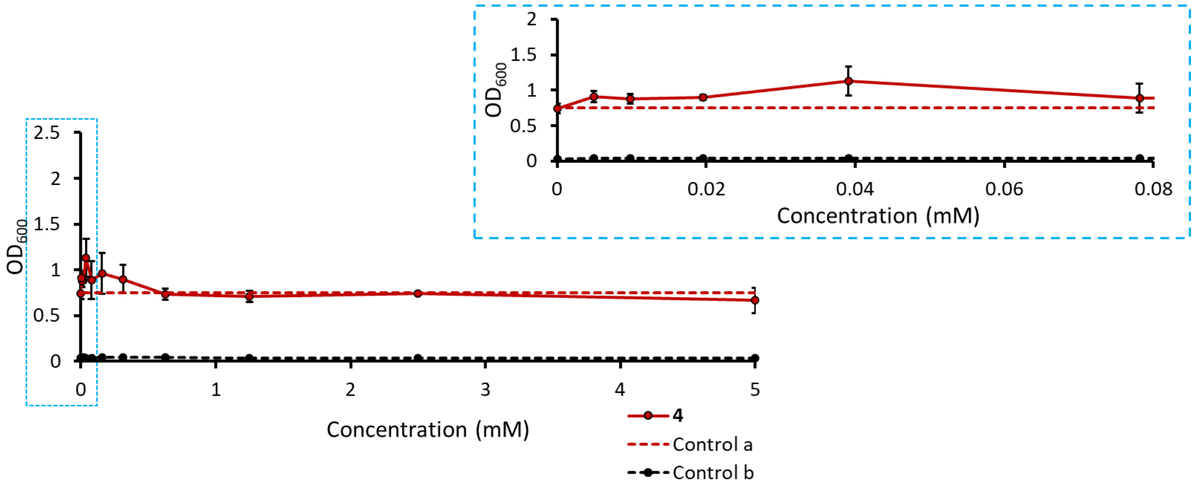

Figure S137 – OD_{600} readings of **4** (solid red line) at increasing concentrations in the presence of PAO1 (*P.aeruginosa*), created from an average of two biological repeats, each containing three technical repeats. Control a (red dashed line) = absence of SSA, Control b (black dashed line) = absence of bacteria, outlined in blue dashed line = enlarged area.

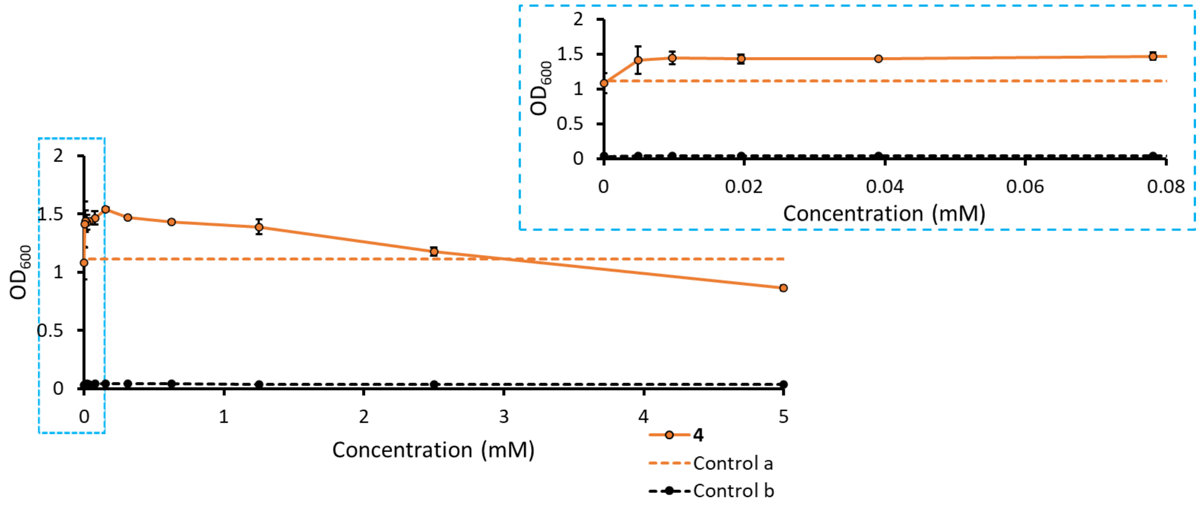

Figure S138 – OD_{600} readings of **4** (solid orange line) at increasing concentrations in the presence of M6 (*K. pneumoniae*), created from an average of two biological repeats, each containing three technical repeats. Control a (orange dashed line) = absence of SSA, Control b (black dashed line) = absence of bacteria, outlined in blue dashed line = enlarged area.

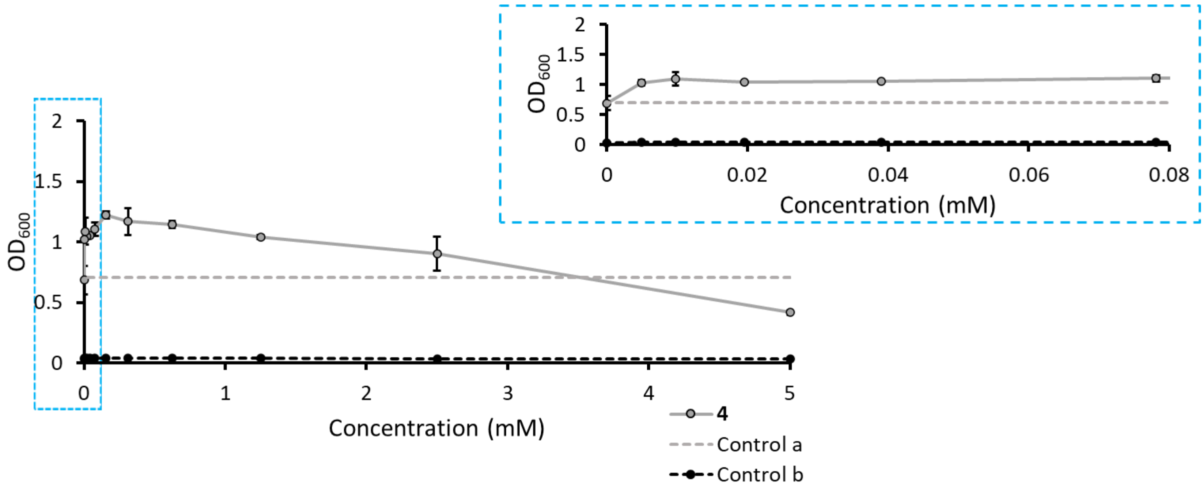

Figure S139 – OD_{600} readings of **4** (solid grey line) at increasing concentrations in the presence of NCTC 12923 (*E. coli*), created from an average of two biological repeats, each containing three technical repeats. Control a (grey dashed line) = absence of SSA, Control b (black dashed line) = absence of bacteria, outlined in blue dashed line = enlarged area.

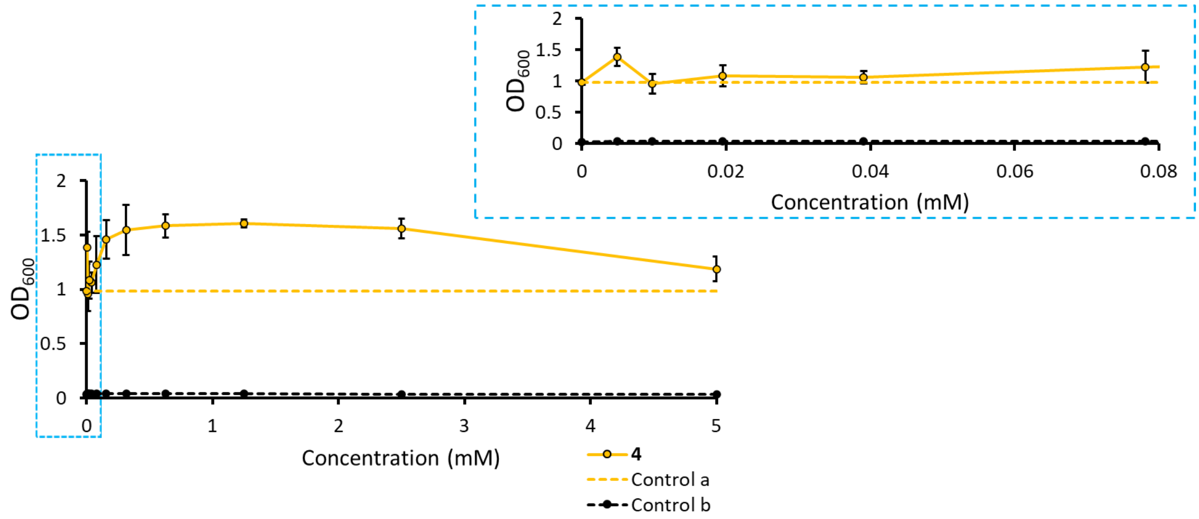

Figure S140 – OD_{600} readings of **4** (solid yellow line) at increasing concentrations in the presence of ATCC 17978 (*A. baumannii*), created from an average of two biological repeats, each containing three technical repeats. Control a (yellow dashed line) = absence of SSA, Control b (black dashed line) = absence of bacteria, outlined in blue dashed line = enlarged area.

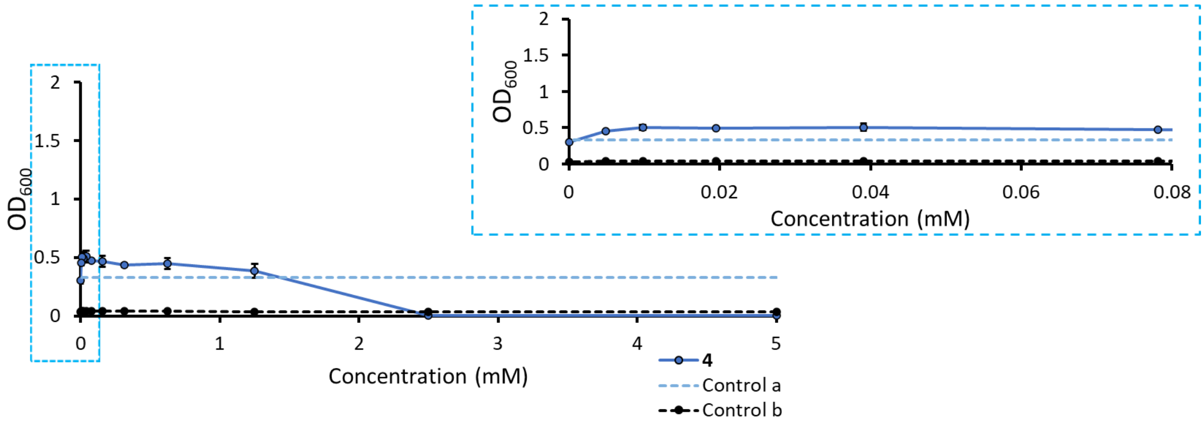

Figure S141 – OD_{600} readings of **4** (solid blue line) at increasing concentrations in the presence of ATCC 9144 (*S. aureus*), created from an average of two biological repeats, each containing three technical repeats. Control a (blue dashed line) = absence of SSA, Control b (black dashed line) = absence of bacteria, outlined in light blue dashed line = enlarged area.

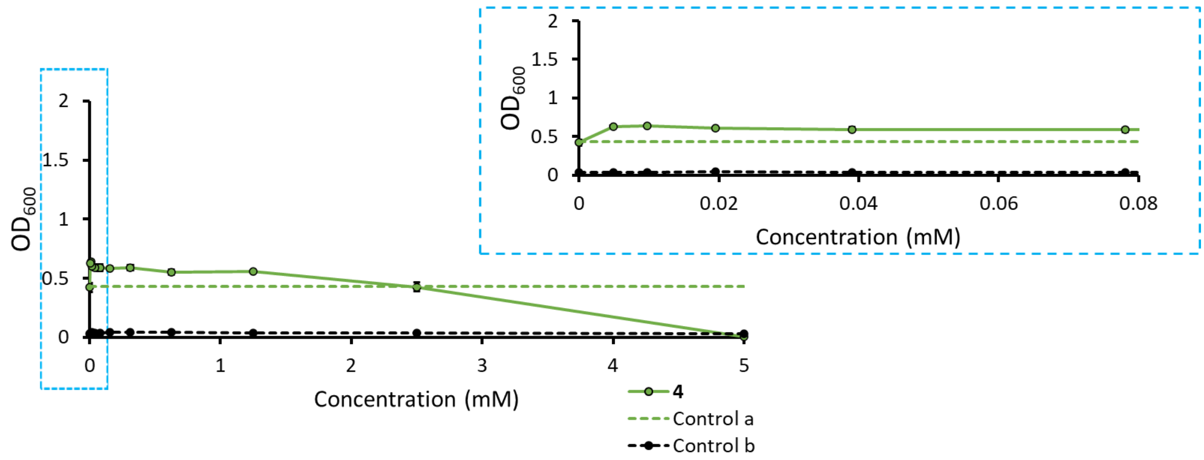

Figure S142 – OD600 readings of **4** (solid green line) at increasing concentrations in the presence of NCTC 775 (*E. faecalis*), created from an average of two biological repeats, each containing three technical repeats. Control a (green dashed line) = absence of SSA, Control b (black dashed line) = absence of bacteria, outlined in blue dashed line = enlarged area.

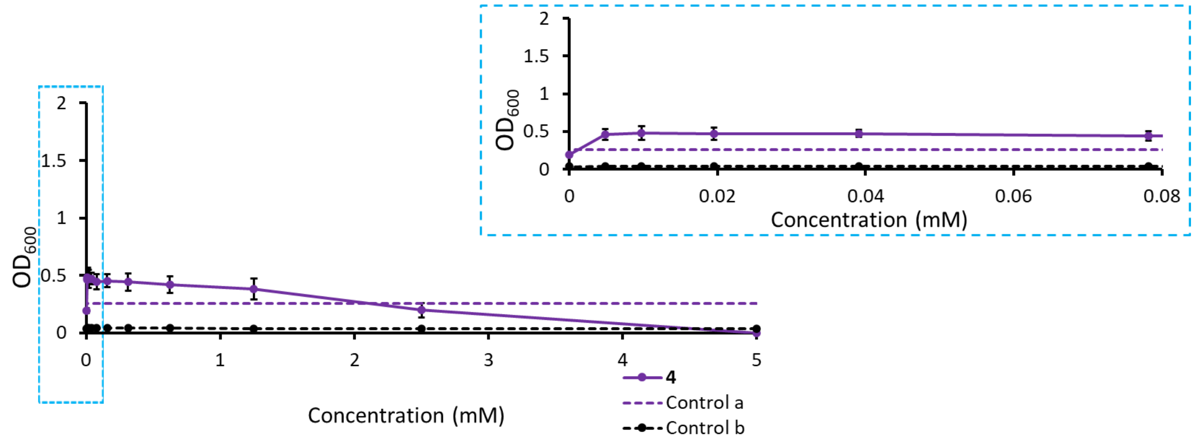

Figure S143 – OD600 readings of **4** (solid purple line) at increasing concentrations in the presence of NCTC 12204 (*E. faecium*), created from an average of two biological repeats, each containing three technical repeats. Control a (purple dashed line) = absence of SSA, Control b (black dashed line) = absence of bacteria, outlined in blue dashed line = enlarged area.

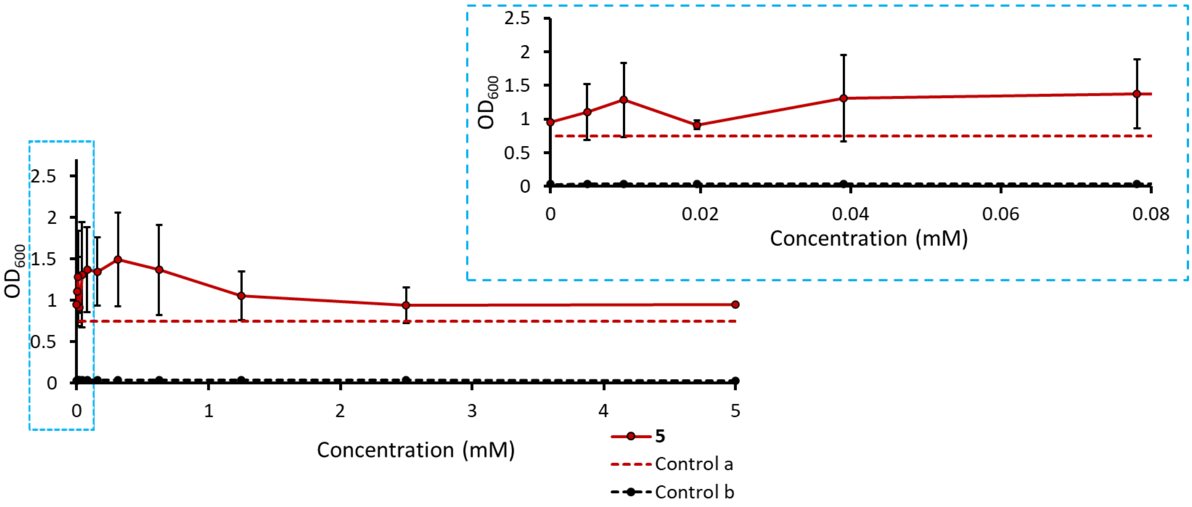

Figure S144 – OD_{600} readings of **5** (solid red line) at increasing concentrations in the presence of PAO1 (*P.aeruginosa*), created from an average of two biological repeats, each containing three technical repeats. Control a (red dashed line) = absence of SSA, Control b (black dashed line) = absence of bacteria, outlined in blue dashed line = enlarged area.

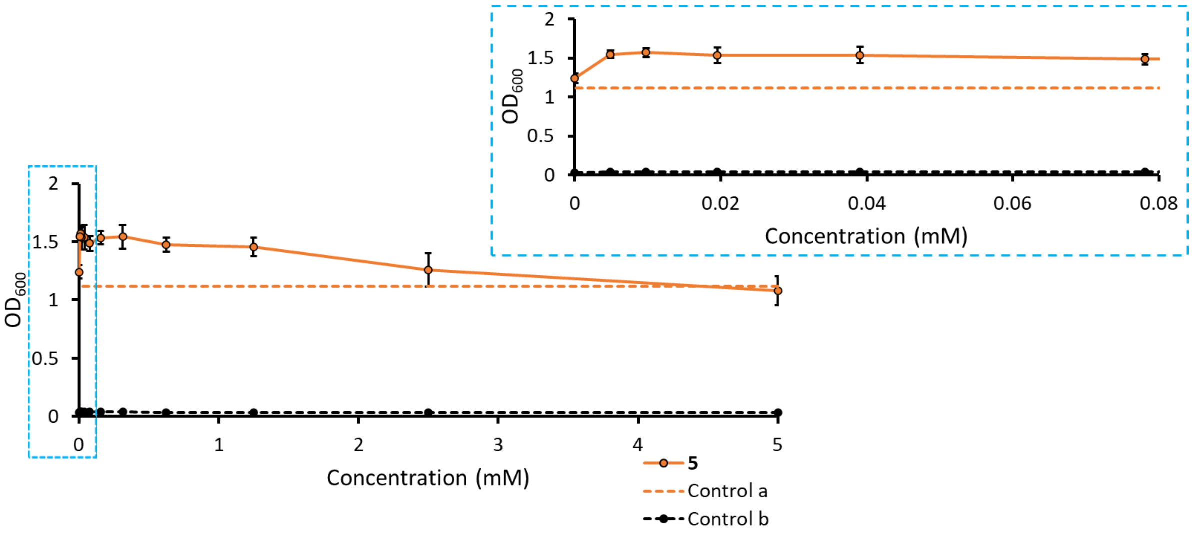

Figure S145 – OD_{600} readings of **5** (solid orange line) at increasing concentrations in the presence of M6 (*K. pneumoniae*), created from an average of two biological repeats, each containing three technical repeats. Control a (orange dashed line) = absence of SSA, Control b (black dashed line) = absence of bacteria, outlined in blue dashed line = enlarged area.

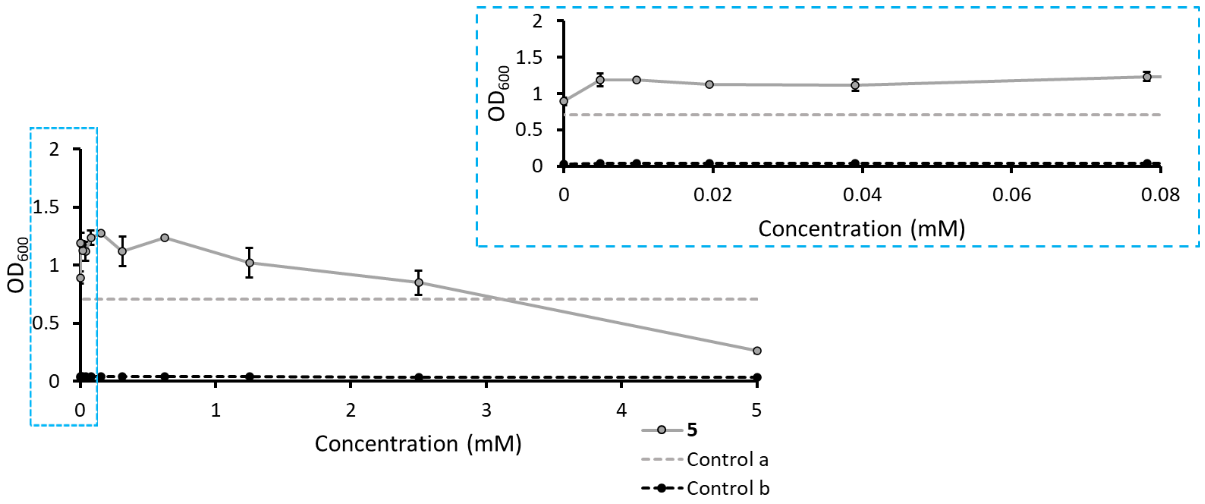

Figure S146 – OD_{600} readings of **5** (solid grey line) at increasing concentrations in the presence of NCTC 12923 (*E. coli*), created from an average of two biological repeats, each containing three technical repeats. Control a (grey dashed line) = absence of SSA, Control b (black dashed line) = absence of bacteria, outlined in blue dashed line = enlarged area.

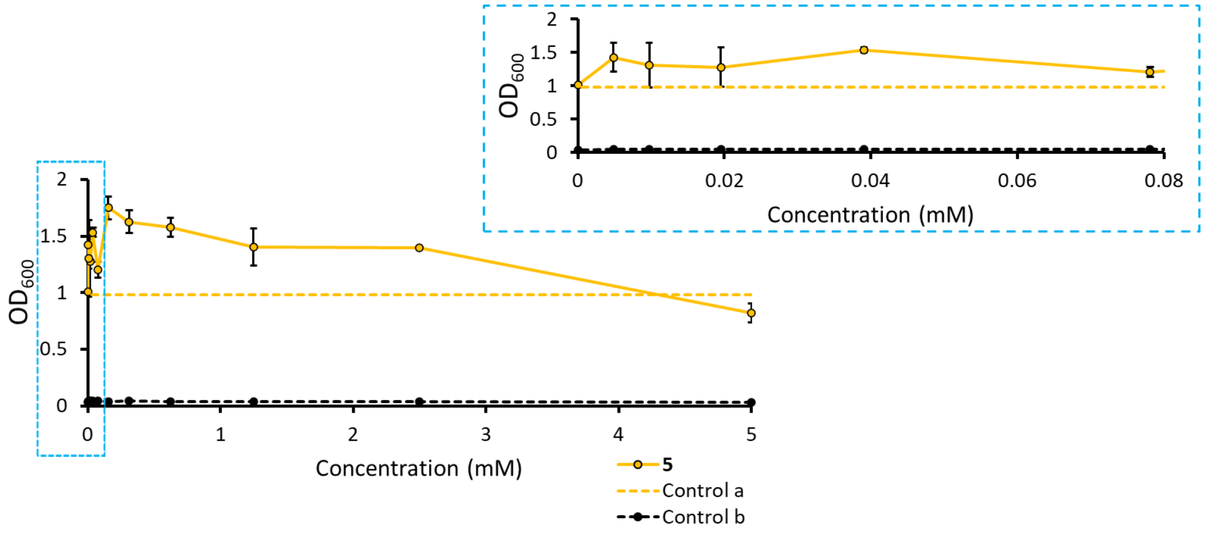

Figure S147 – OD_{600} readings of **5** (solid yellow line) at increasing concentrations in the presence of ATCC 17978 (*A. baumannii*), created from an average of two biological repeats, each containing three technical repeats. Control a (yellow dashed line) = absence of SSA, Control b (black dashed line) = absence of bacteria, outlined in blue dashed line = enlarged area.

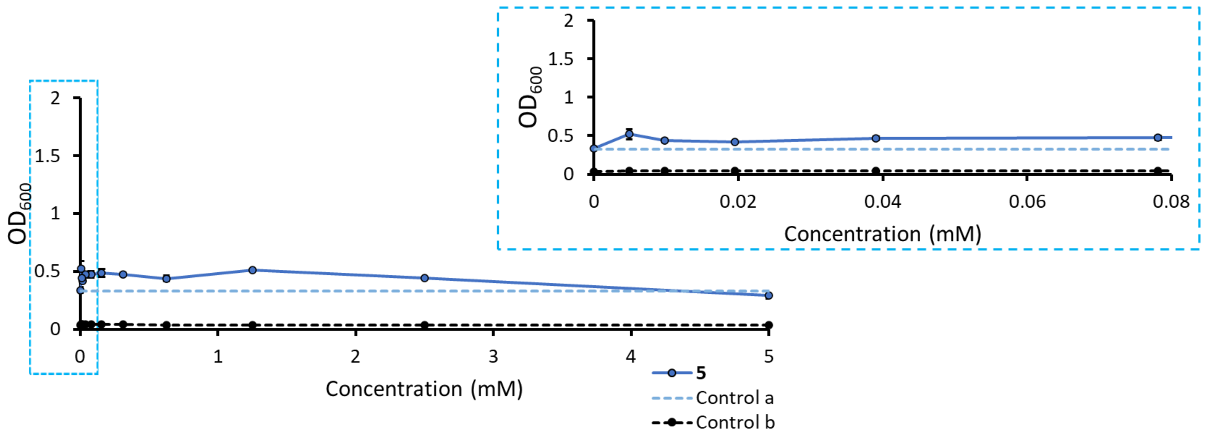

Figure S148 – OD_{600} readings of **5** (solid blue line) at increasing concentrations in the presence of ATCC 9144 (*S. aureus*), created from an average of two biological repeats, each containing three technical repeats. Control a (blue dashed line) = absence of SSA, Control b (black dashed line) = absence of bacteria, outlined in light blue dashed line = enlarged area.

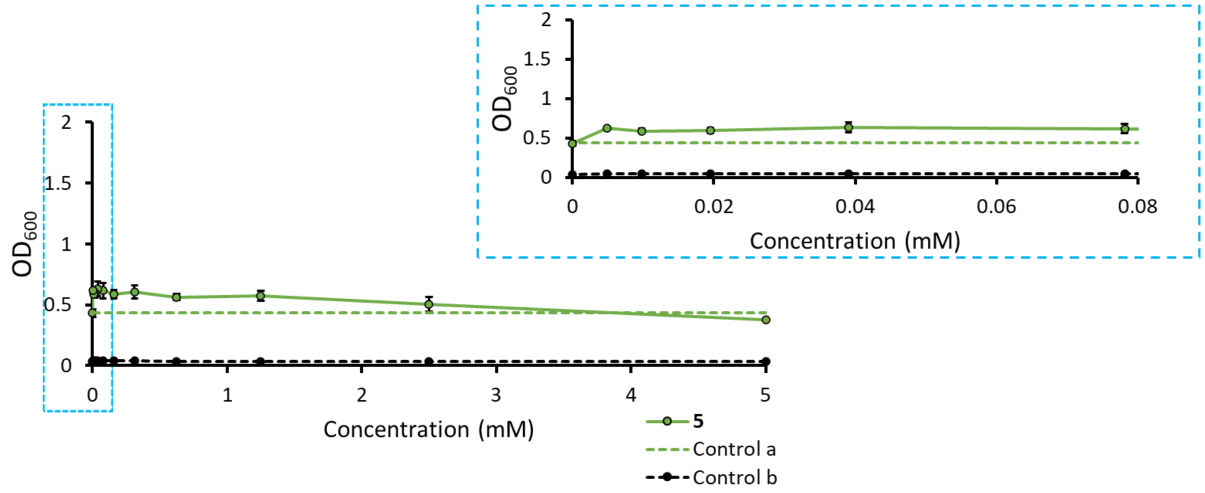

Figure S149 – OD_{600} readings of **5** (solid green line) at increasing concentrations in the presence of NCTC 775 (*E. faecalis*), created from an average of two biological repeats, each containing three technical repeats. Control a (green dashed line) = absence of SSA, Control b (black dashed line) = absence of bacteria, outlined in blue dashed line = enlarged area.

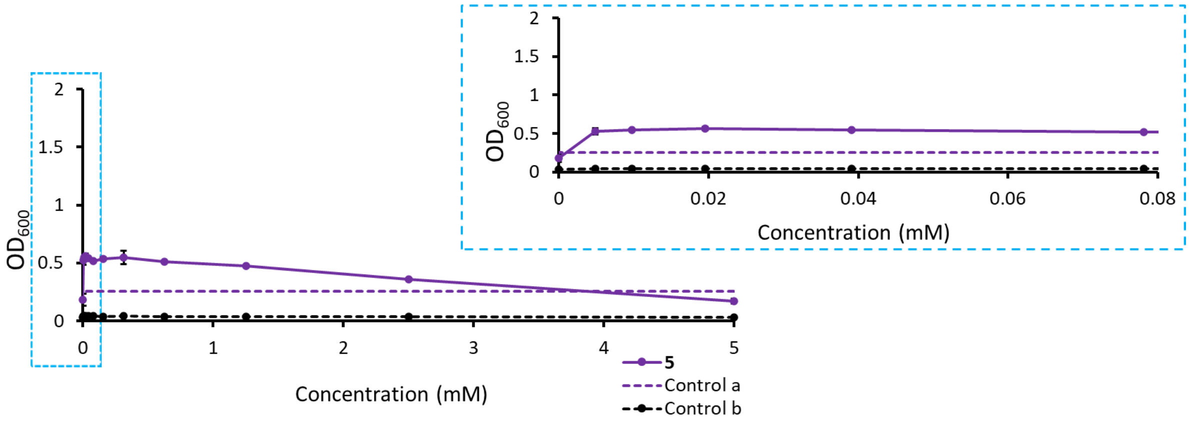

Figure S150 – OD_{600} readings of **5** (solid purple line) at increasing concentrations in the presence of NCTC 12204 (*E. faecium*), created from an average of two biological repeats, each containing three technical repeats. Control a (purple dashed line) = absence of SSA, Control b (black dashed line) = absence of bacteria, outlined in blue dashed line = enlarged area.

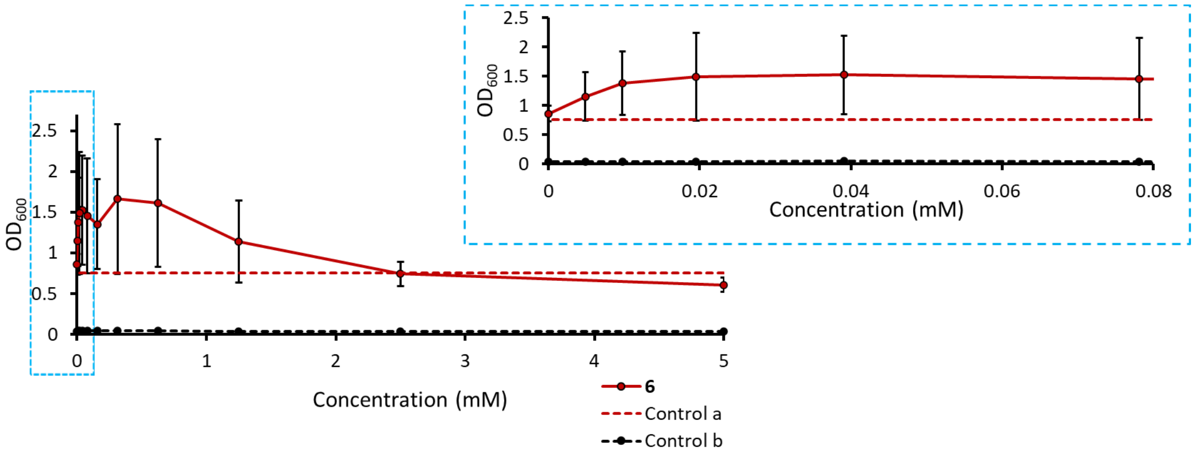

Figure S151 – OD_{600} readings of **6** (solid red line) at increasing concentrations in the presence of PAO1 (*P.aeruginosa*), created from an average of two biological repeats, each containing three technical repeats. Control a (red dashed line) = absence of SSA, Control b (black dashed line) = absence of bacteria, outlined in blue dashed line = enlarged area.

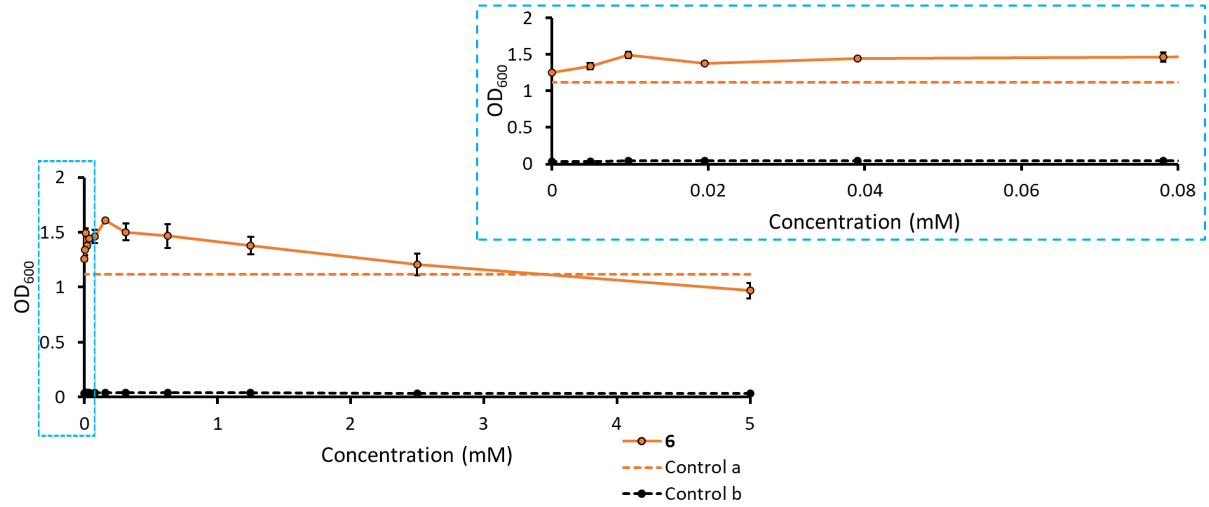

Figure S152 – OD_{600} readings of **6** (solid orange line) at increasing concentrations in the presence of M6 (*K. pneumoniae*), created from an average of two biological repeats, each containing three technical repeats. Control a (orange dashed line) = absence of SSA, Control b (black dashed line) = absence of bacteria, outlined in blue dashed line = enlarged area.

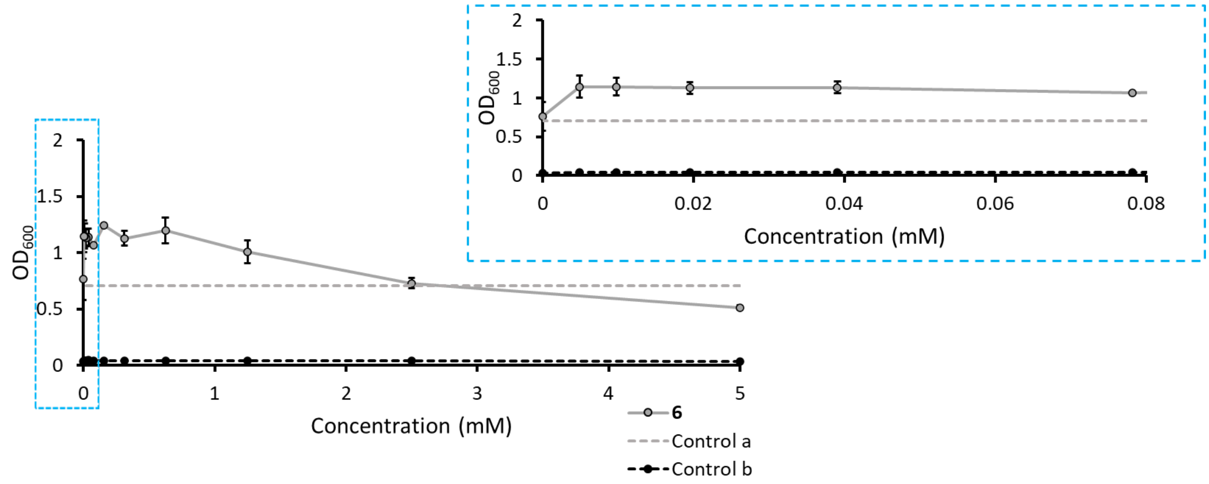

Figure S153 – OD_{600} readings of **6** (solid grey line) at increasing concentrations in the presence of NCTC 12923 (*E. coli*), created from an average of two biological repeats, each containing three technical repeats. Control a (grey dashed line) = absence of SSA, Control b (black dashed line) = absence of bacteria, outlined in blue dashed line = enlarged area.

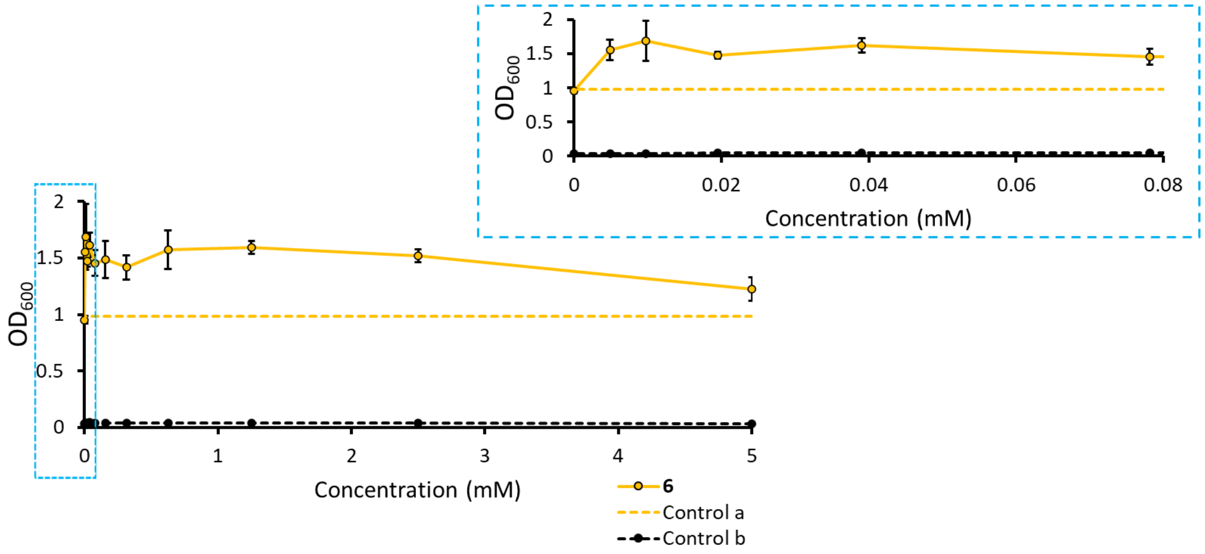

Figure S154 – OD_{600} readings of **6** (solid yellow line) at increasing concentrations in the presence of ATCC 17978 (*A. baumannii*), created from an average of two biological repeats, each containing three technical repeats. Control a (yellow dashed line) = absence of SSA, Control b (black dashed line) = absence of bacteria, outlined in blue dashed line = enlarged area.

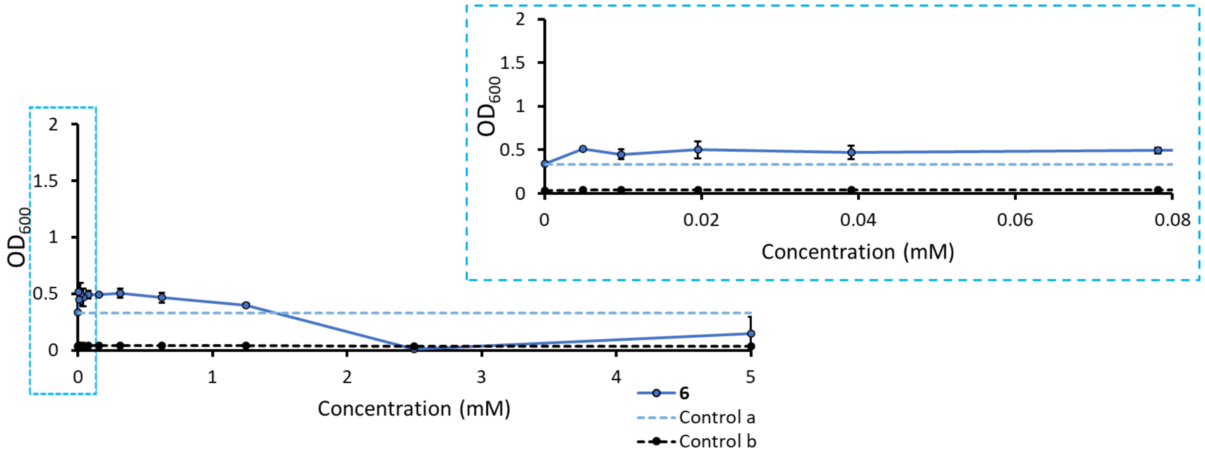

Figure S155 – OD_{600} readings of **6** (solid blue line) at increasing concentrations in the presence of ATCC 9144 (*S. aureus*), created from an average of two biological repeats, each containing three technical repeats. Control a (blue dashed line) = absence of SSA, Control b (black dashed line) = absence of bacteria, outlined in light blue dashed line = enlarged area.

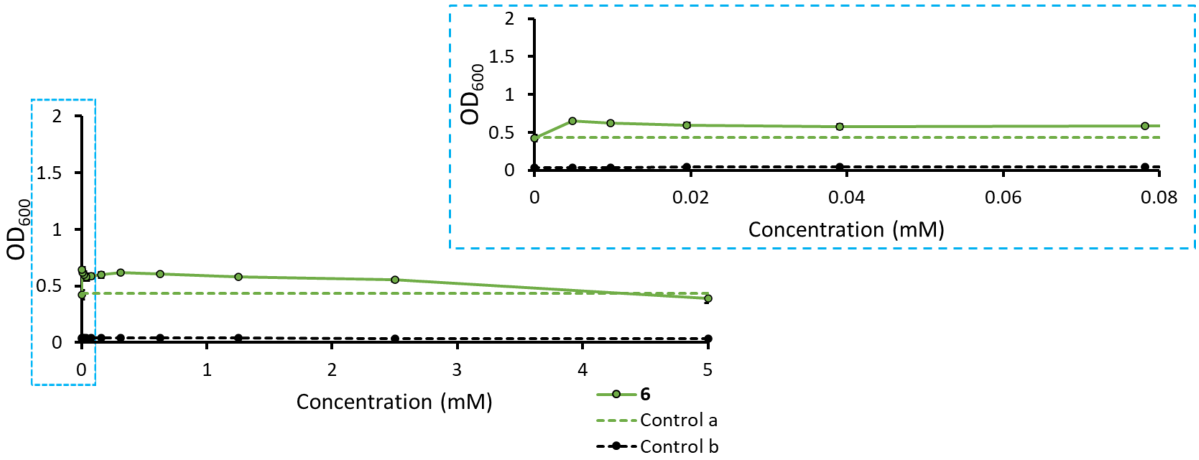

Figure S156 – OD_{600} readings of **6** (solid green line) at increasing concentrations in the presence of NCTC 775 (*E. faecalis*), created from an average of two biological repeats, each containing three technical repeats. Control a (green dashed line) = absence of SSA, Control b (black dashed line) = absence of bacteria, outlined in blue dashed line = enlarged area.

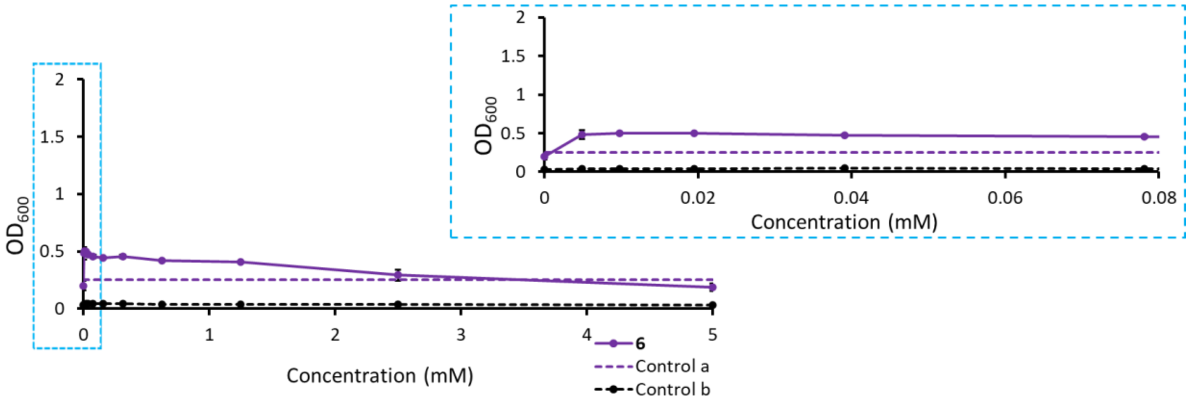

Figure S157 – OD_{600} readings of **6** (solid purple line) at increasing concentrations in the presence of NCTC 12204 (*E. faecium*), created from an average of two biological repeats, each containing three technical repeats. Control a (purple dashed line) = absence of SSA, Control b (black dashed line) = absence of bacteria, outlined in blue dashed line = enlarged area.

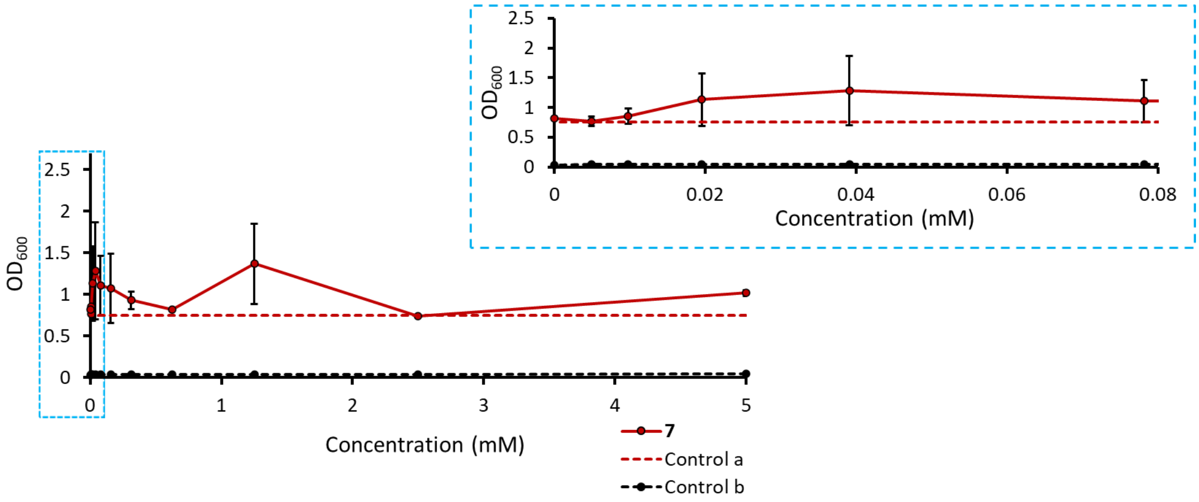

Figure S158 – OD_{600} readings of **7** (solid red line) at increasing concentrations in the presence of PAO1 (*P.aeruginosa*), created from an average of two biological repeats, each containing three technical repeats. Control a (red dashed line) = absence of SSA, Control b (black dashed line) = absence of bacteria, outlined in blue dashed line = enlarged area.

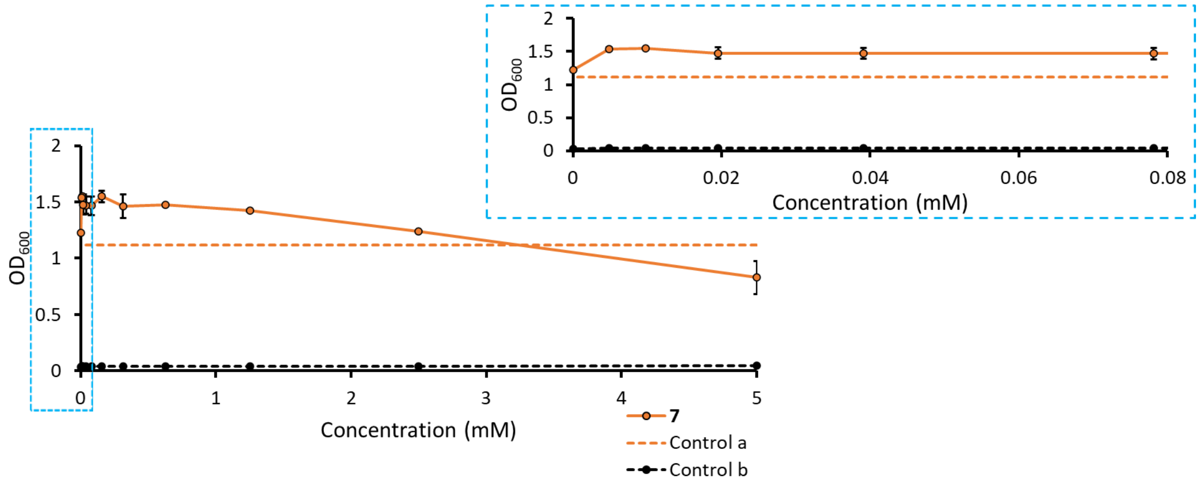

Figure S159 – OD_{600} readings of **7** (solid orange line) at increasing concentrations in the presence of M6 (*K. pneumoniae*), created from an average of two biological repeats, each containing three technical repeats. Control a (orange dashed line) = absence of SSA, Control b (black dashed line) = absence of bacteria, outlined in blue dashed line = enlarged area.

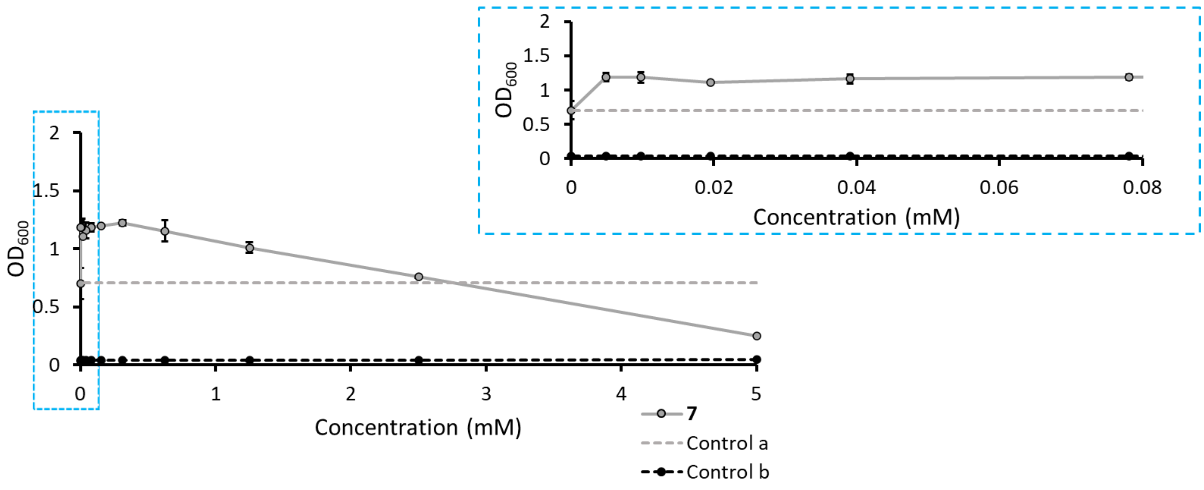

Figure S160 – OD_{600} readings of **7** (solid grey line) at increasing concentrations in the presence of NCTC 12923 (*E. coli*), created from an average of two biological repeats, each containing three technical repeats. Control a (grey dashed line) = absence of SSA, Control b (black dashed line) = absence of bacteria, outlined in blue dashed line = enlarged area.

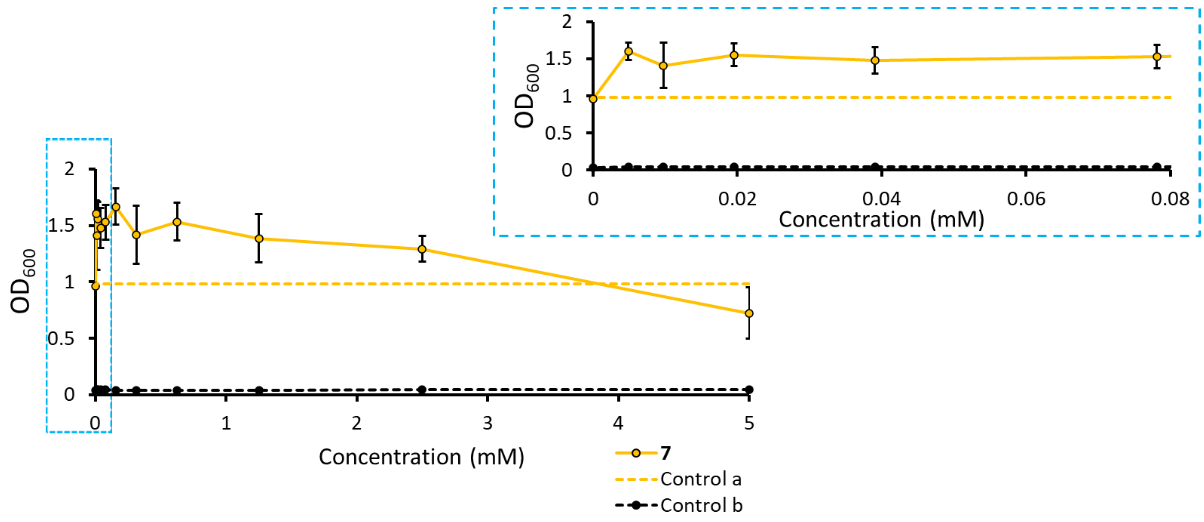

Figure S161 – OD_{600} readings of **7** (solid yellow line) at increasing concentrations in the presence of ATCC 17978 (*A. baumannii*), created from an average of two biological repeats, each containing three technical repeats. Control a (yellow dashed line) = absence of SSA, Control b (black dashed line) = absence of bacteria, outlined in blue dashed line = enlarged area.

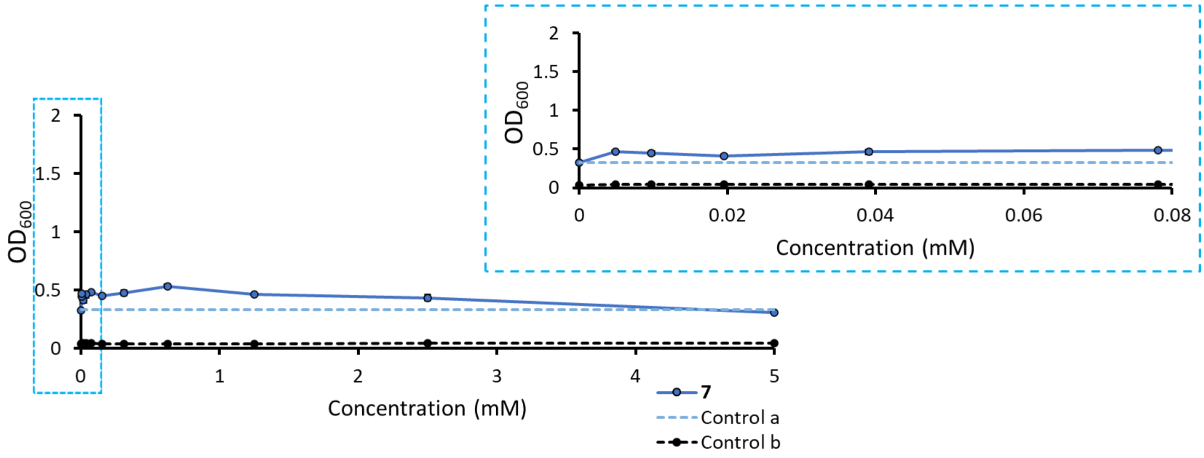

Figure S162 – OD_{600} readings of **7** (solid blue line) at increasing concentrations in the presence of ATCC 9144 (*S. aureus*), created from an average of two biological repeats, each containing three technical repeats. Control a (blue dashed line) = absence of SSA, Control b (black dashed line) = absence of bacteria, outlined in light blue dashed line = enlarged area.

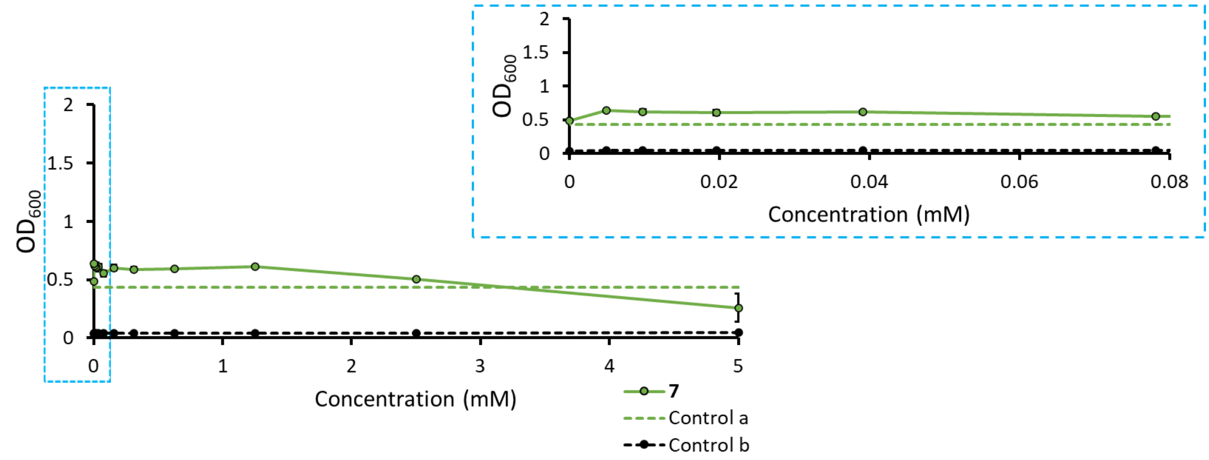

Figure S163 – OD_{600} readings of **7** (solid green line) at increasing concentrations in the presence of NCTC 775 (*E. faecalis*), created from an average of two biological repeats, each containing three technical repeats. Control a (green dashed line) = absence of SSA, Control b (black dashed line) = absence of bacteria, outlined in blue dashed line = enlarged area.

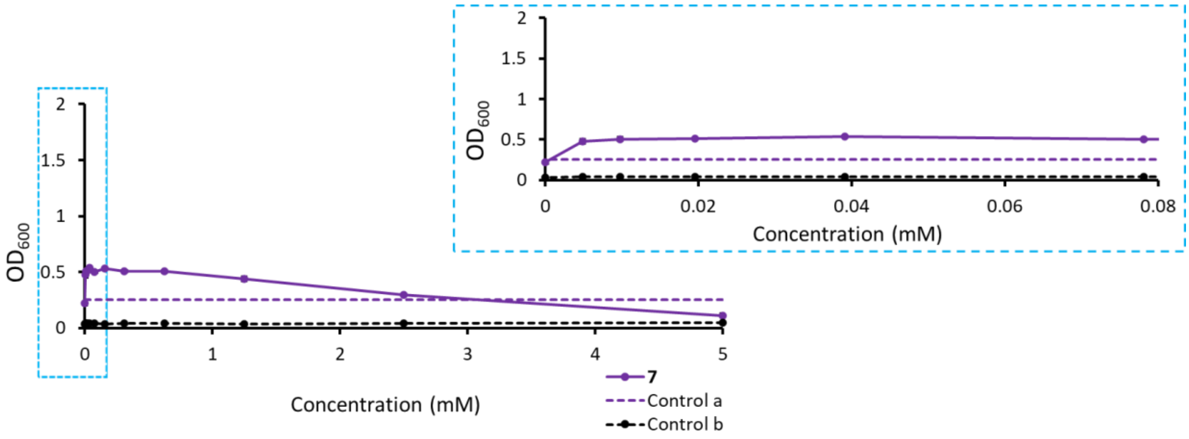

Figure S164 – OD_{600} readings of **7** (solid purple line) at increasing concentrations in the presence of NCTC 12204 (*E. faecium*), created from an average of two biological repeats, each containing three technical repeats. Control a (purple dashed line) = absence of SSA, Control b (black dashed line) = absence of bacteria, outlined in blue dashed line = enlarged area.

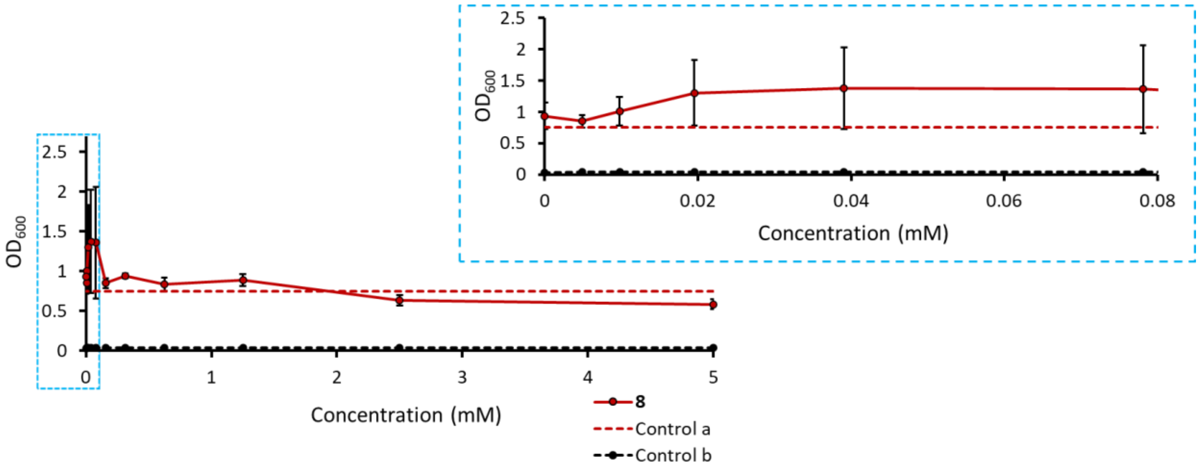

Figure S165 – OD_{600} readings of **8** (solid red line) at increasing concentrations in the presence of PAO1 (*P.aeruginosa*), created from an average of two biological repeats, each containing three technical repeats. Control a (red dashed line) = absence of SSA, Control b (black dashed line) = absence of bacteria, outlined in blue dashed line = enlarged area.

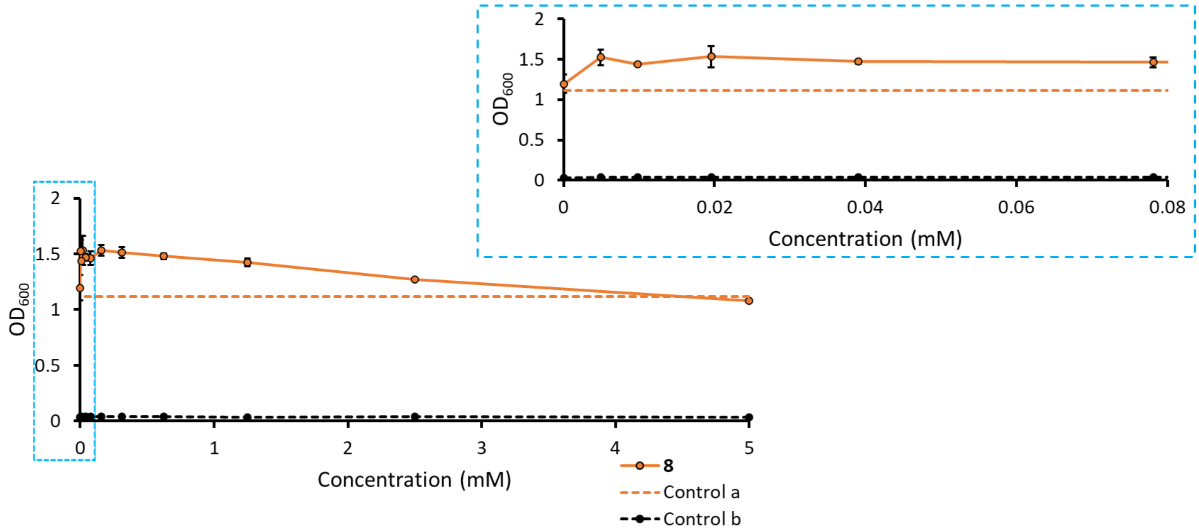

Figure S166 – OD600 readings of **8** (solid orange line) at increasing concentrations in the presence of M6 (*K. pneumoniae*), created from an average of two biological repeats, each containing three technical repeats. Control a (orange dashed line) = absence of SSA, Control b (black dashed line) = absence of bacteria, outlined in blue dashed line = enlarged area.

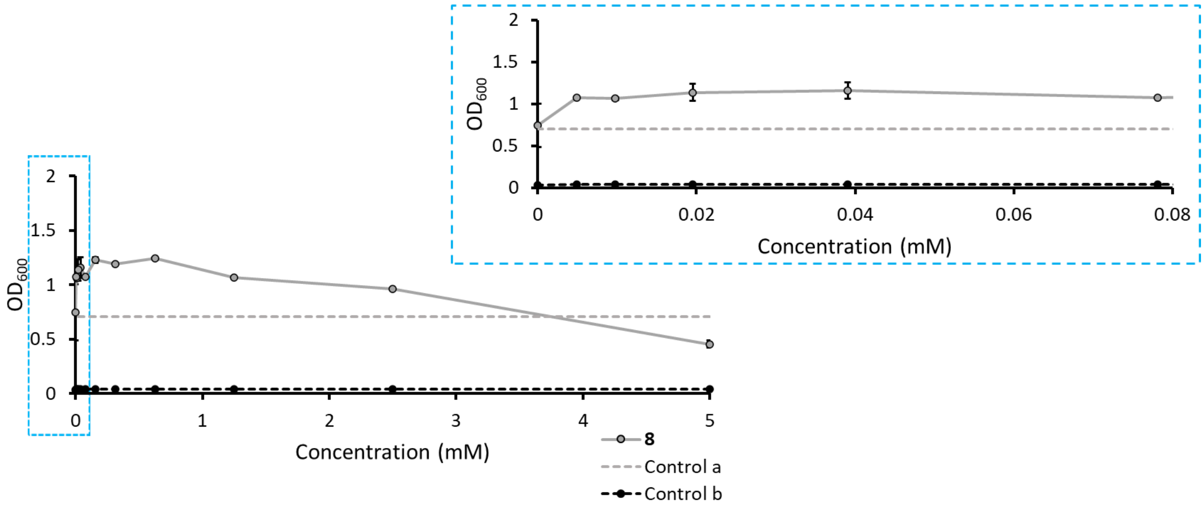

Figure S167 – OD600 readings of **8** (solid grey line) at increasing concentrations in the presence of NCTC 12923 (*E. coli*), created from an average of two biological repeats, each containing three technical repeats. Control a (grey dashed line) = absence of SSA, Control b (black dashed line) = absence of bacteria, outlined in blue dashed line = enlarged area.

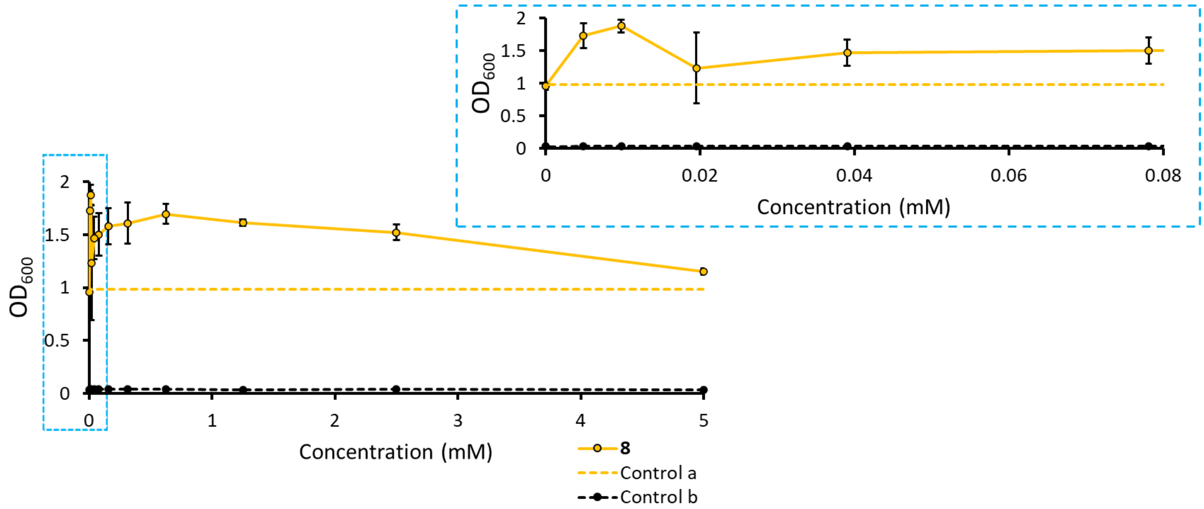

Figure S168 – OD600 readings of **8** (solid yellow line) at increasing concentrations in the presence of ATCC 17978 (*A. baumannii*), created from an average of two biological repeats, each containing three technical repeats. Control a (yellow dashed line) = absence of SSA, Control b (black dashed line) = absence of bacteria, outlined in blue dashed line = enlarged area.

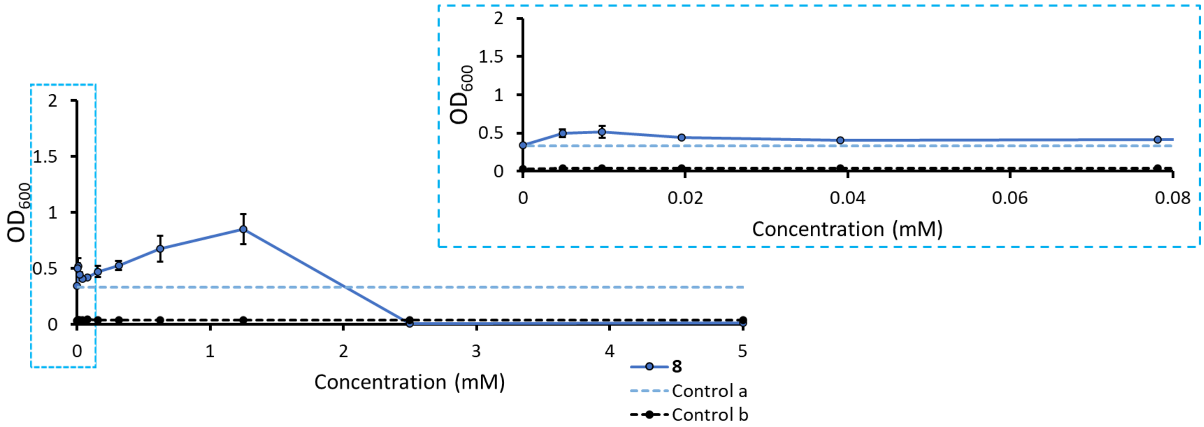

Figure S169 – OD600 readings of **8** (solid blue line) at increasing concentrations in the presence of ATCC 9144 (*S. aureus*), created from an average of two biological repeats, each containing three technical repeats. Control a (blue dashed line) = absence of SSA, Control b (black dashed line) = absence of bacteria, outlined in light blue dashed line = enlarged area.

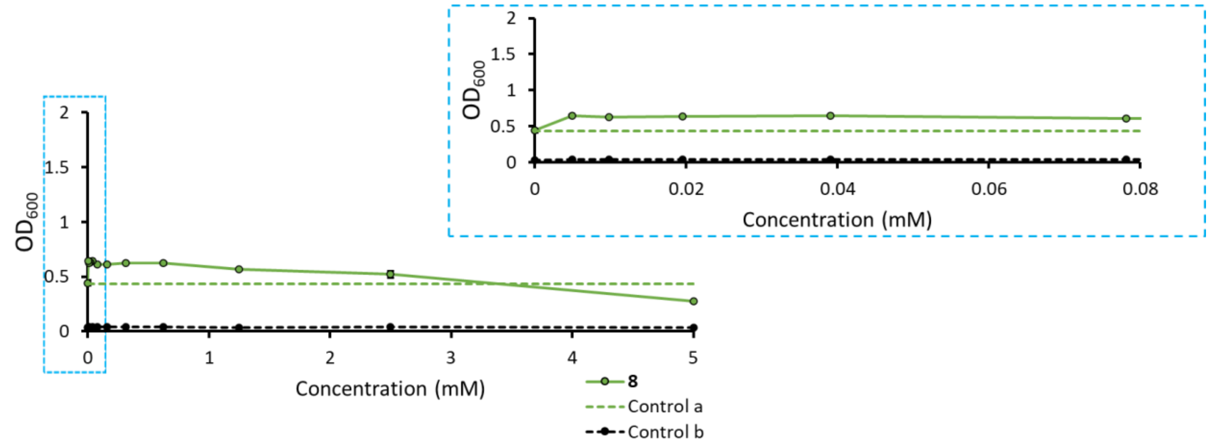

Figure S170 – OD_{600} readings of **8** (solid green line) at increasing concentrations in the presence of NCTC 775 (*E. faecalis*), created from an average of two biological repeats, each containing three technical repeats. Control a (green dashed line) = absence of SSA, Control b (black dashed line) = absence of bacteria, outlined in blue dashed line = enlarged area.

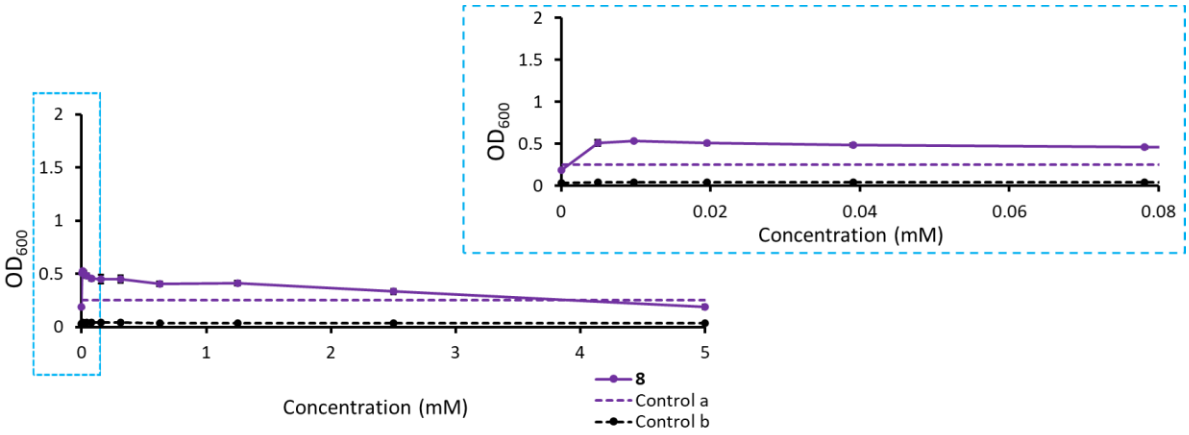

Figure S171 – OD_{600} readings of **8** (solid purple line) at increasing concentrations in the presence of NCTC 12204 (*E. faecium*), created from an average of two biological repeats, each containing three technical repeats. Control a (purple dashed line) = absence of SSA, Control b (black dashed line) = absence of bacteria, outlined in blue dashed line = enlarged area.

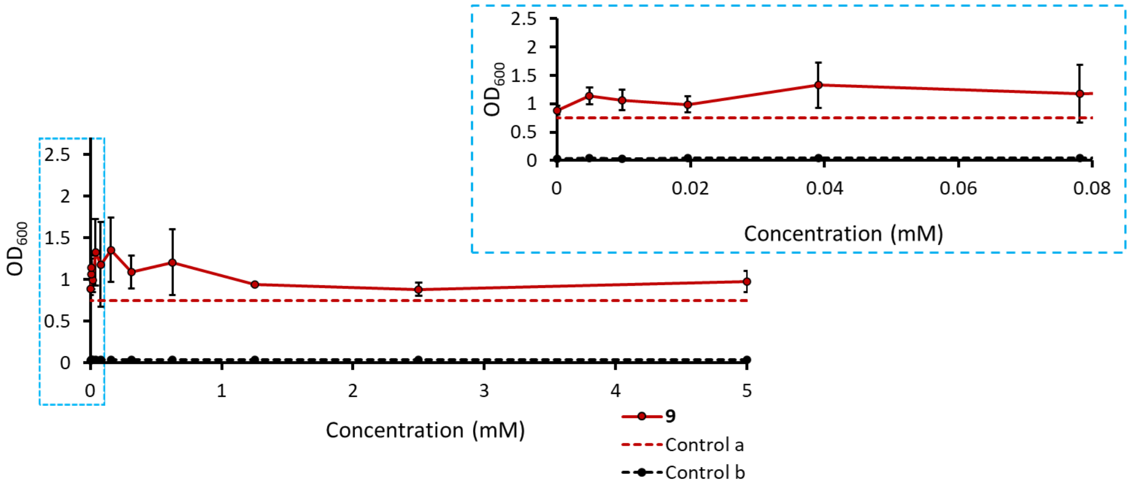

Figure S172 – OD600 readings of **9** (solid red line) at increasing concentrations in the presence of PAO1 (*P.aeruginosa*), created from an average of two biological repeats, each containing three technical repeats. Control a (red dashed line) = absence of SSA, Control b (black dashed line) = absence of bacteria, outlined in blue dashed line = enlarged area.

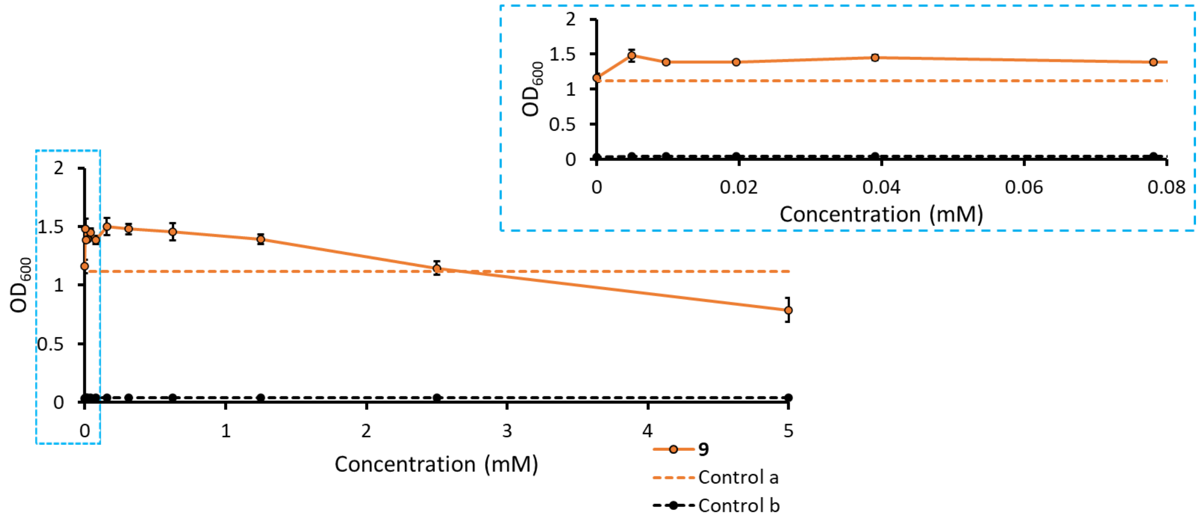

Figure S173 – OD600 readings of **9** (solid orange line) at increasing concentrations in the presence of M6 (*K. pneumoniae*), created from an average of two biological repeats, each containing three technical repeats. Control a (orange dashed line) = absence of SSA, Control b (black dashed line) = absence of bacteria, outlined in blue dashed line = enlarged area.

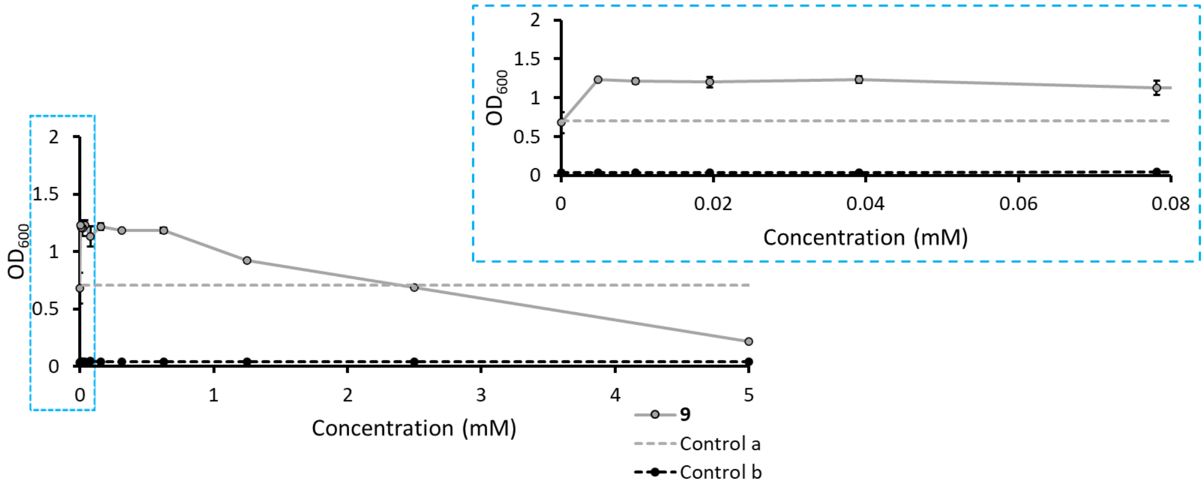

Figure S174 – OD600 readings of **9** (solid grey line) at increasing concentrations in the presence of NCTC 12923 (*E. coli*), created from an average of two biological repeats, each containing three technical repeats. Control a (grey dashed line) = absence of SSA, Control b (black dashed line) = absence of bacteria, outlined in blue dashed line = enlarged area.

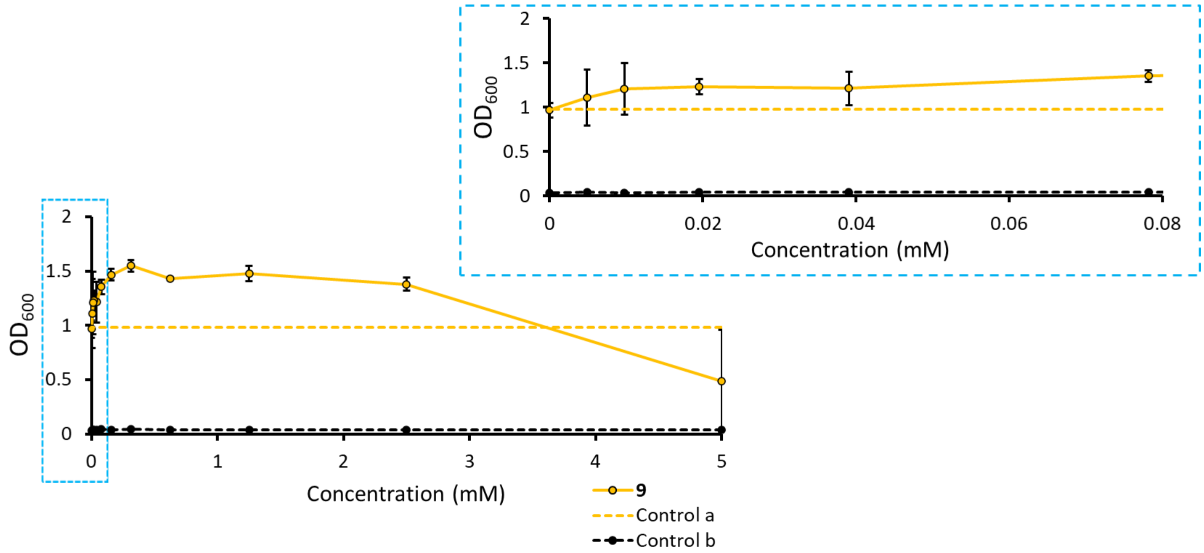

Figure S175 – OD600 readings of **9** (solid yellow line) at increasing concentrations in the presence of ATCC 17978 (*A. baumannii*), created from an average of two biological repeats, each containing three technical repeats. Control a (yellow dashed line) = absence of SSA, Control b (black dashed line) = absence of bacteria, outlined in blue dashed line = enlarged area.

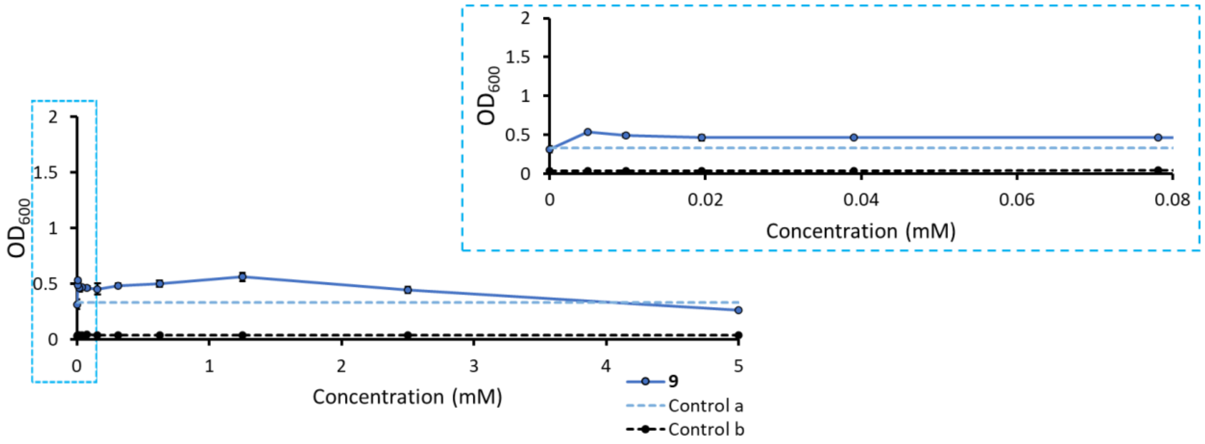

Figure S176 – OD_{600} readings of **9** (solid blue line) at increasing concentrations in the presence of ATCC 9144 (*S. aureus*), created from an average of two biological repeats, each containing three technical repeats. Control a (blue dashed line) = absence of SSA, Control b (black dashed line) = absence of bacteria, outlined in light blue dashed line = enlarged area.

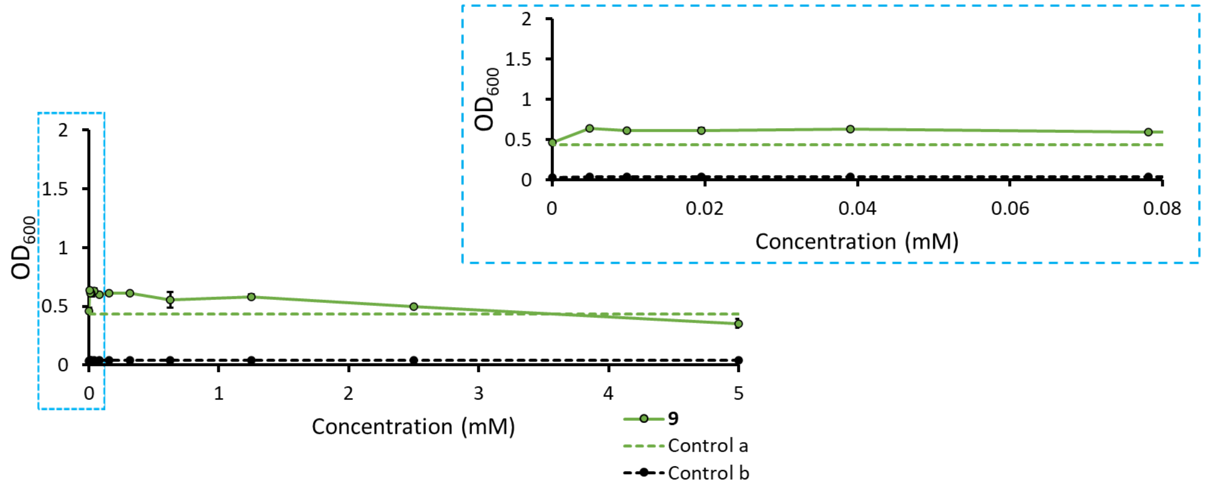

Figure S177 – OD_{600} readings of **9** (solid green line) at increasing concentrations in the presence of NCTC 775 (*E. faecalis*), created from an average of two biological repeats, each containing three technical repeats. Control a (green dashed line) = absence of SSA, Control b (black dashed line) = absence of bacteria, outlined in blue dashed line = enlarged area.

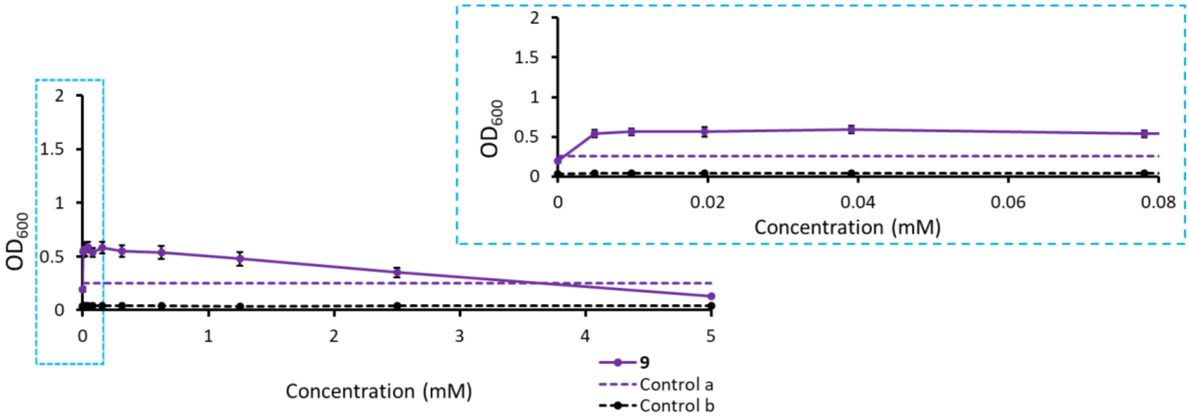

Figure S178 – OD_{600} readings of **9** (solid purple line) at increasing concentrations in the presence of NCTC 12204 (*E. faecium*), created from an average of two biological repeats, each containing three technical repeats. Control a (purple dashed line) = absence of SSA, Control b (black dashed line) = absence of bacteria, outlined in blue dashed line = enlarged area.

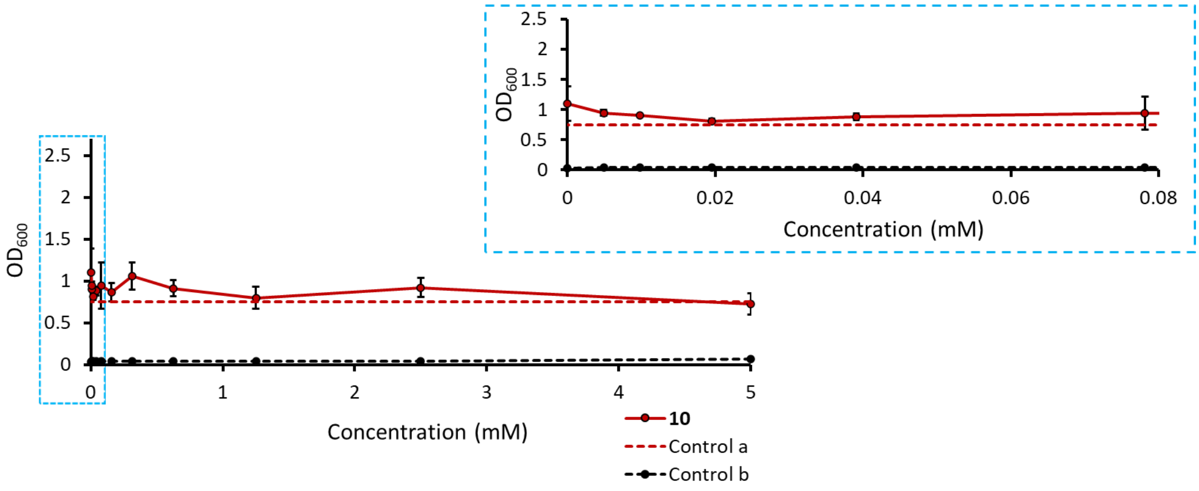

Figure S179 – OD_{600} readings of **10** (solid red line) at increasing concentrations in the presence of PAO1 (*P.aeruginosa*), created from an average of two biological repeats, each containing three technical repeats. Control a (red dashed line) = absence of SSA, Control b (black dashed line) = absence of bacteria, outlined in blue dashed line = enlarged area.

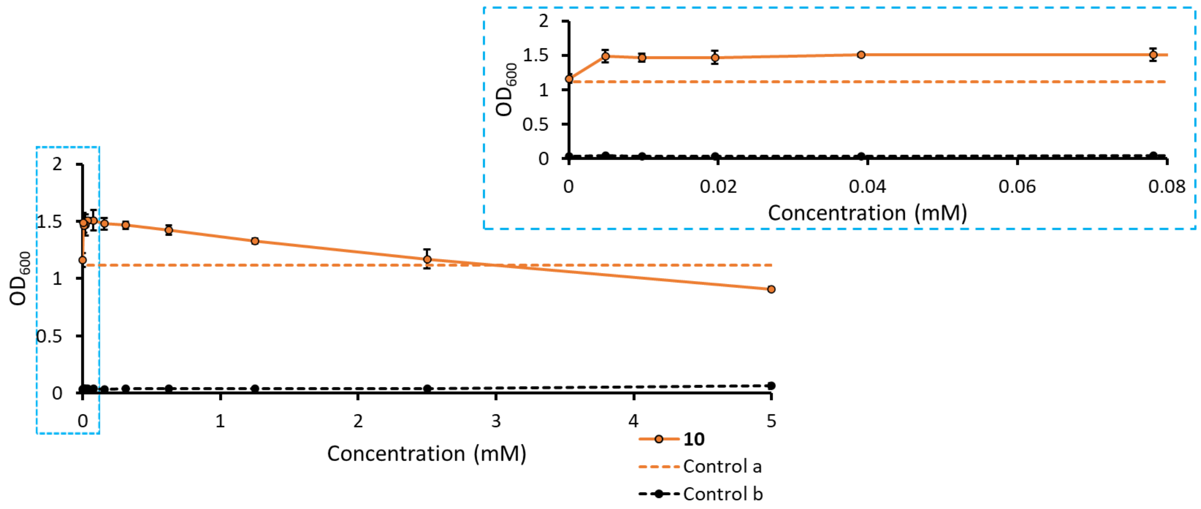

Figure S180 – OD_{600} readings of **10** (solid orange line) at increasing concentrations in the presence of M6 (*K. pneumoniae*), created from an average of two biological repeats, each containing three technical repeats. Control a (orange dashed line) = absence of SSA, Control b (black dashed line) = absence of bacteria, outlined in blue dashed line = enlarged area.

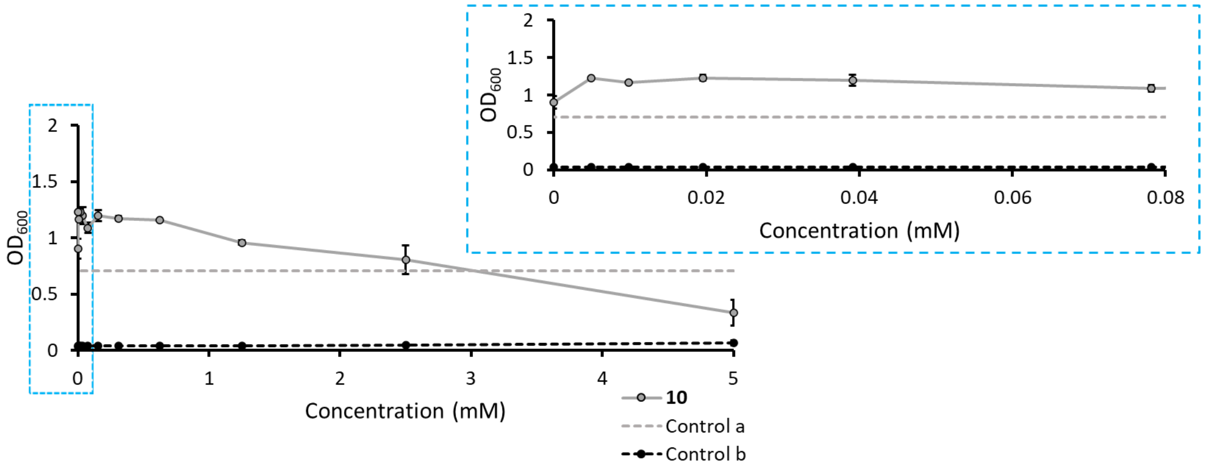

Figure S181 – OD_{600} readings of **10** (solid grey line) at increasing concentrations in the presence of NCTC 12923 (*E. coli*), created from an average of two biological repeats, each containing three technical repeats. Control a (grey dashed line) = absence of SSA, Control b (black dashed line) = absence of bacteria, outlined in blue dashed line = enlarged area.

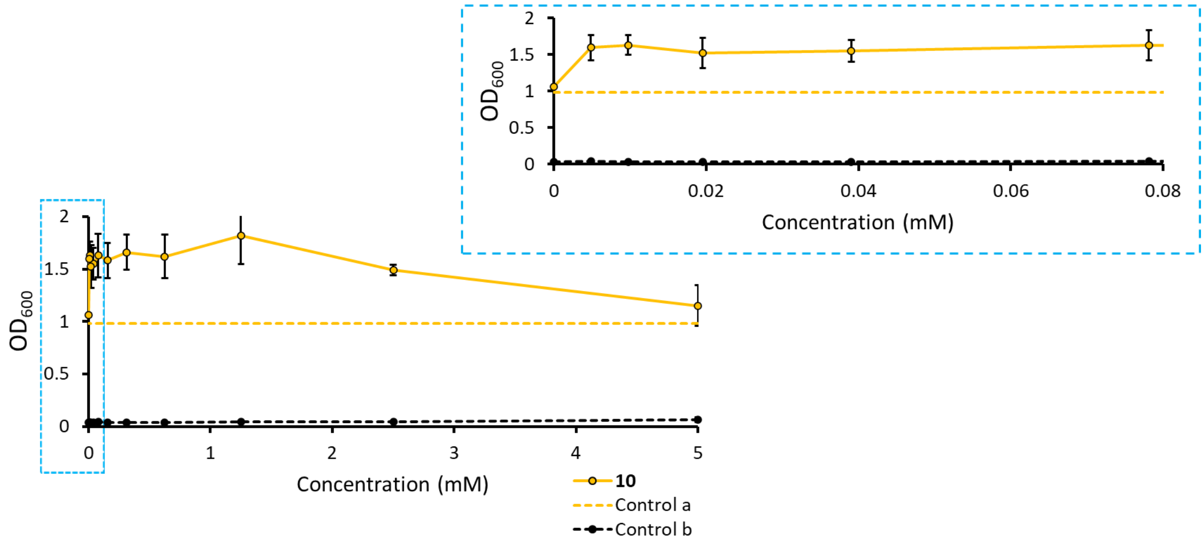

Figure S182 – OD_{600} readings of **10** (solid yellow line) at increasing concentrations in the presence of ATCC 17978 (*A. baumannii*), created from an average of two biological repeats, each containing three technical repeats. Control a (yellow dashed line) = absence of SSA, Control b (black dashed line) = absence of bacteria, outlined in blue dashed line = enlarged area.

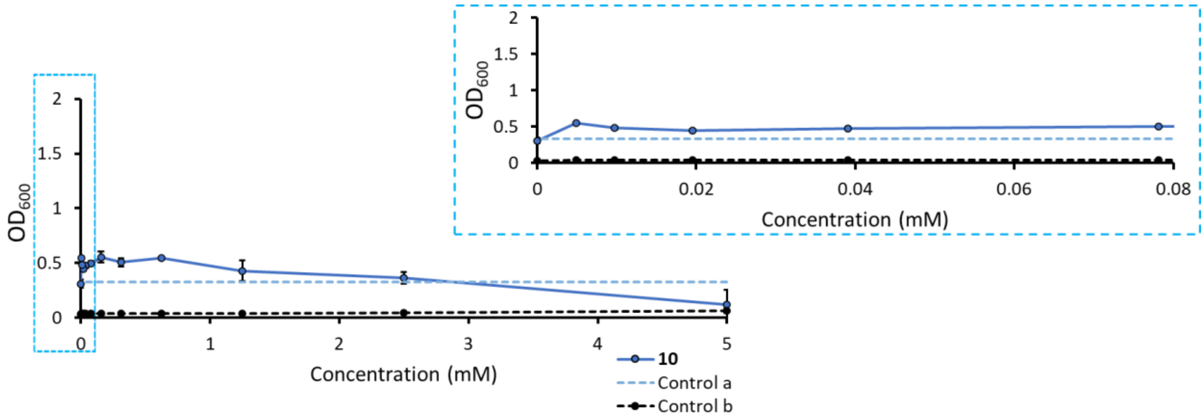

Figure S183 – OD_{600} readings of **10** (solid blue line) at increasing concentrations in the presence of ATCC 9144 (*S. aureus*), created from an average of two biological repeats, each containing three technical repeats. Control a (blue dashed line) = absence of SSA, Control b (black dashed line) = absence of bacteria, outlined in light blue dashed line = enlarged area.

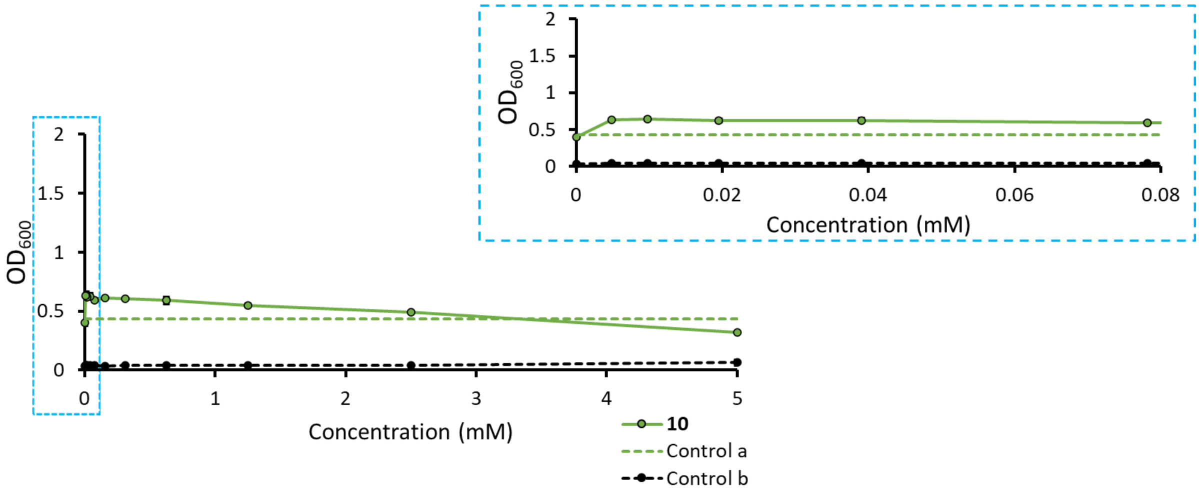

Figure S184 – OD600 readings of **10** (solid green line) at increasing concentrations in the presence of NCTC 775 (*E. faecalis*), created from an average of two biological repeats, each containing three technical repeats. Control a (green dashed line) = absence of SSA, Control b (black dashed line) = absence of bacteria, outlined in blue dashed line = enlarged area.

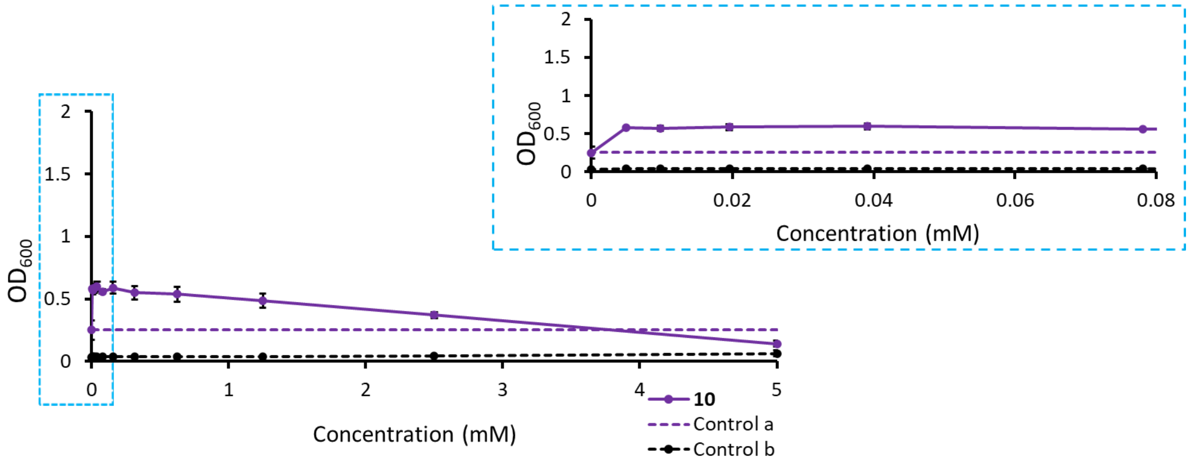

Figure S185 – OD600 readings of **10** (solid purple line) at increasing concentrations in the presence of NCTC 12204 (*E. faecium*), created from an average of two biological repeats, each containing three technical repeats. Control a (purple dashed line) = absence of SSA, Control b (black dashed line) = absence of bacteria, outlined in blue dashed line = enlarged area.

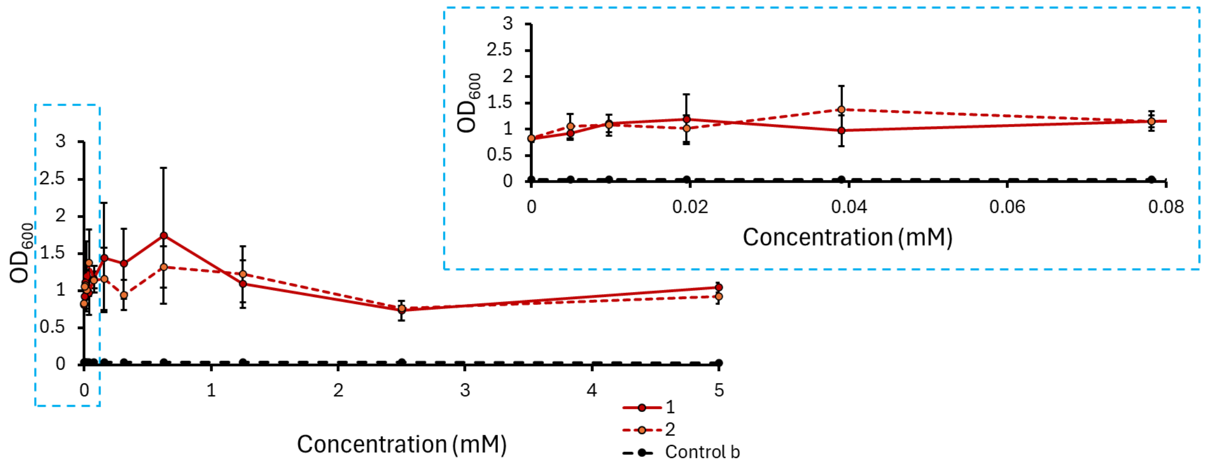

Figure S186 – Comparison of OD_{600} readings of **1** (solid red line) and **2** (dashed red line) at increasing concentrations in the presence of PAO1 (*P.aeruginosa*), created from an average of two biological repeats, each containing three technical repeats. Control b (black dashed line) = absence of bacteria, outlined in blue dashed line = enlarged area.

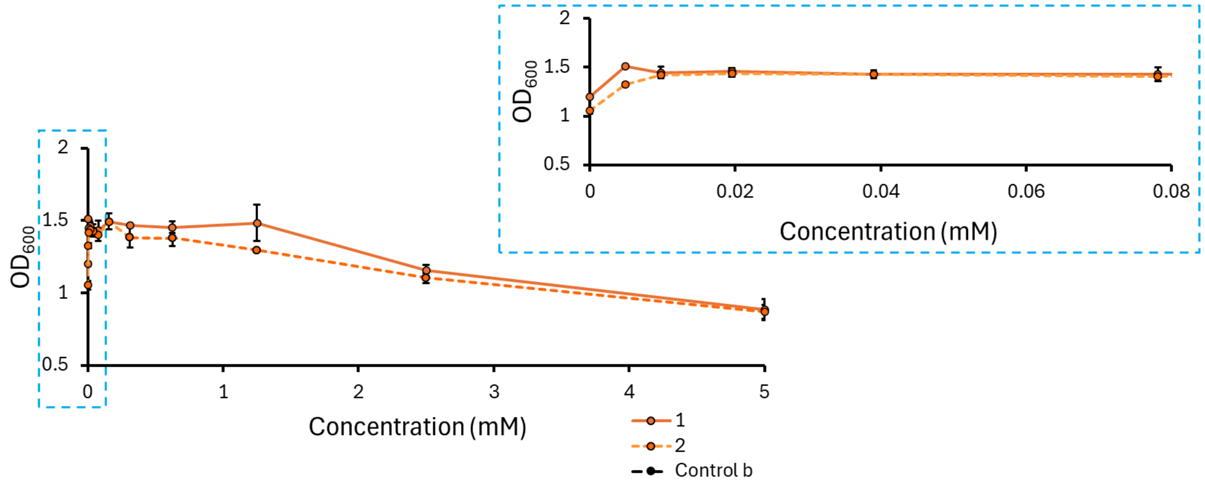

Figure S187 – Comparison of OD_{600} readings of **1** (solid orange line) and **2** (dashed orange line) at increasing concentrations in the presence of M6 (*K. pneumoniae*), created from an average of two biological repeats, each containing three technical repeats. Control b (black dashed line) = absence of bacteria, outlined in blue dashed line = enlarged area.

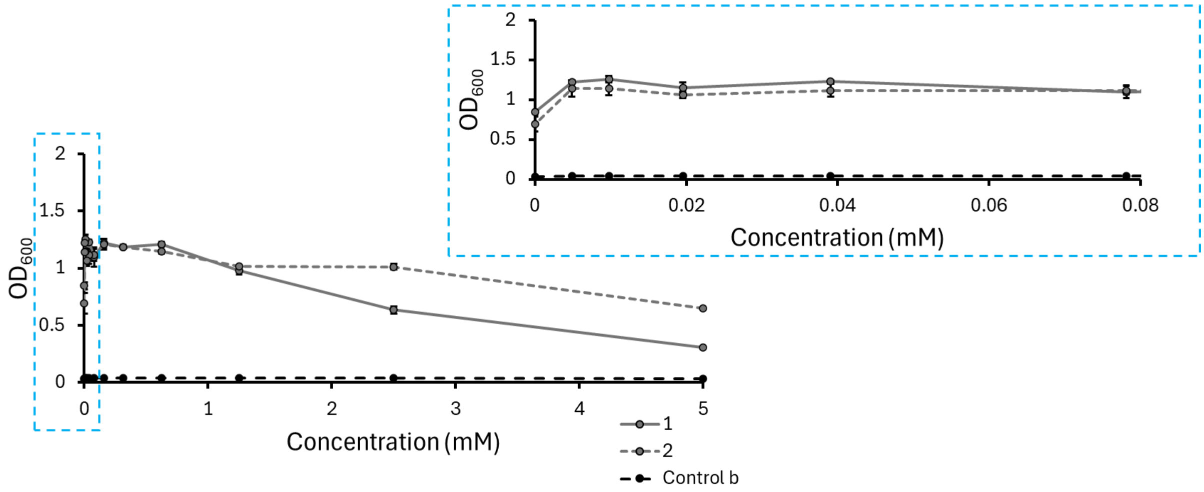

Figure S188 – Comparison of OD_{600} readings of **1** (solid grey line) and **2** (dashed grey line) at increasing concentrations in the presence of NCTC 12923 (*E. coli*), created from an average of two biological repeats, each containing three technical repeats. Control b (black dashed line) = absence of bacteria, outlined in blue dashed line = enlarged area.

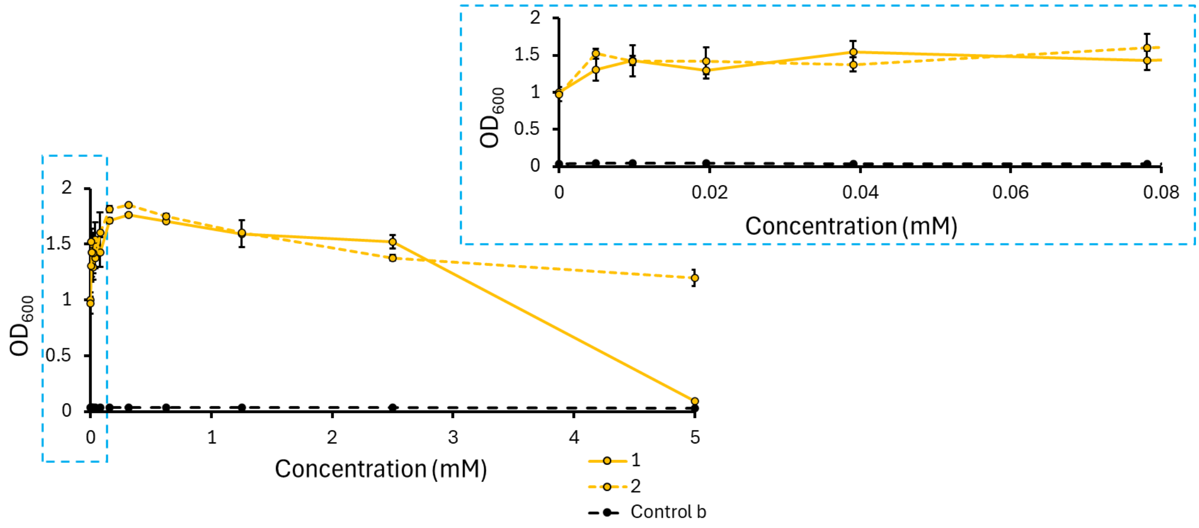

Figure S189 – Comparison of OD_{600} readings of **1** (solid yellow line) and **2** (dashed yellow line) at increasing concentrations in the presence of ATCC 17978 (*A. baumannii*), created from an average of two biological repeats, each containing three technical repeats. Control b (black dashed line) = absence of bacteria, outlined in blue dashed line = enlarged area.

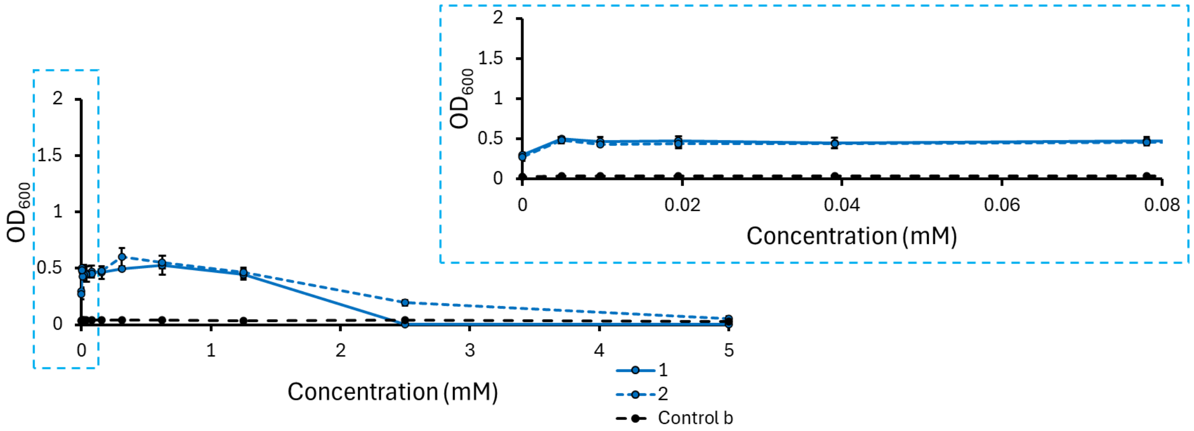

Figure S190 – Comparison of OD_{600} readings of **1** (solid blue line) and **2** (dashed blue line) at increasing concentrations in the presence of ATCC 9144 (*S. aureus*), created from an average of two biological repeats, each containing three technical repeats. Control b (black dashed line) = absence of bacteria, outlined in light blue dashed line = enlarged area.

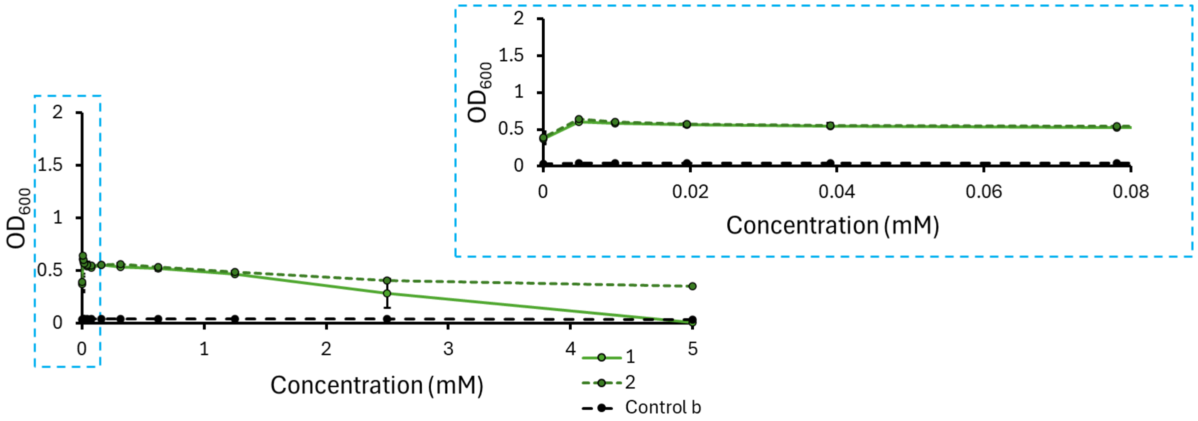

Figure S191 – Comparison of OD_{600} readings of **1** (solid green line) and **2** (dashed green line) at increasing concentrations in the presence of NCTC 775 (*E. faecalis*), created from an average of two biological repeats, each containing three technical repeats. Control b (black dashed line) = absence of bacteria, outlined in blue dashed line = enlarged area.

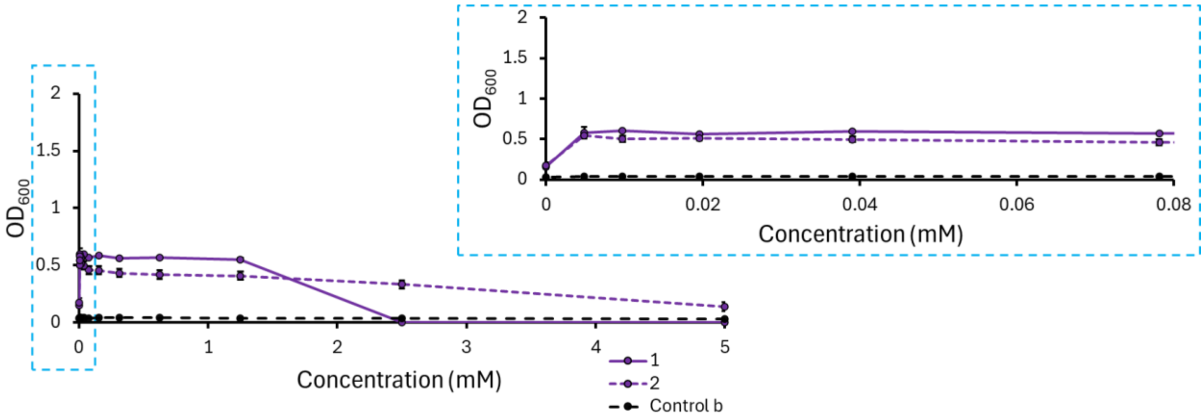

Figure S192 – Comparison of OD_{600} readings of **1** (solid purple line) and **2** (dashed purple line) at increasing concentrations in the presence of NCTC 12204 (*E. faecium*), created from an average of two biological repeats, each containing three technical repeats. Control b (black dashed line) = absence of bacteria, outlined in blue dashed line = enlarged area.

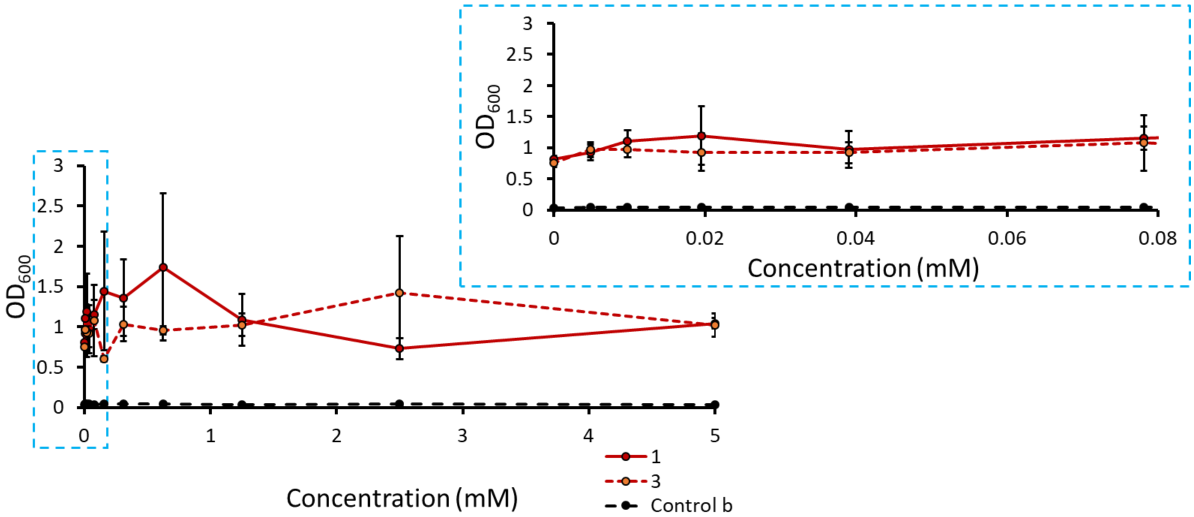

Figure S193 – Comparison of OD_{600} readings of **1** (solid red line) and **3** (dashed red line) at increasing concentrations in the presence of PAO1 (*P.aeruginosa*), created from an average of two biological repeats, each containing three technical repeats. Control b (black dashed line) = absence of bacteria, outlined in blue dashed line = enlarged area.

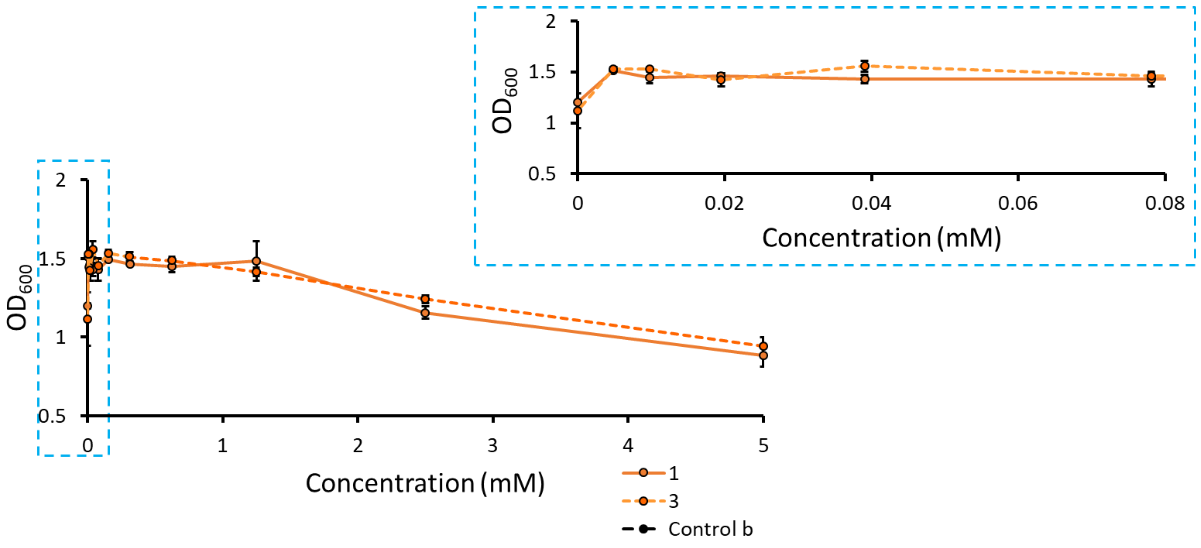

Figure S194 – Comparison of OD_{600} readings of **1** (solid orange line) and **3** (dashed orange line) at increasing concentrations in the presence of M6 (*K. pneumoniae*), created from an average of two biological repeats, each containing three technical repeats. Control b (black dashed line) = absence of bacteria, outlined in blue dashed line = enlarged area.

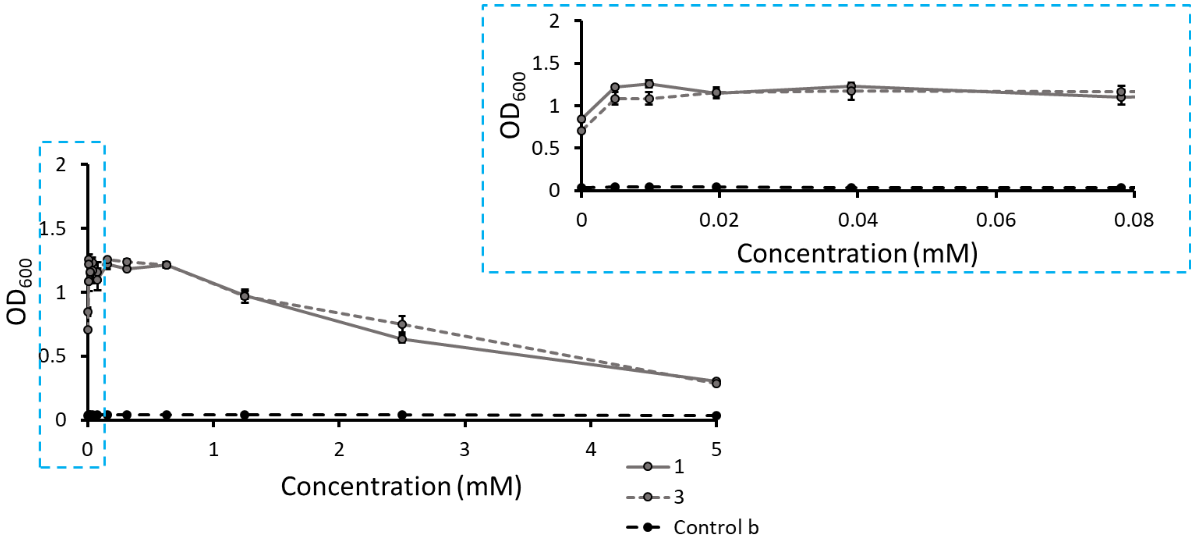

Figure S195 – Comparison of OD_{600} readings of **1** (solid grey line) and **3** (dashed grey line) at increasing concentrations in the presence of NCTC 12923 (*E. coli*), created from an average of two biological repeats, each containing three technical repeats. Control b (black dashed line) = absence of bacteria, outlined in blue dashed line = enlarged area.

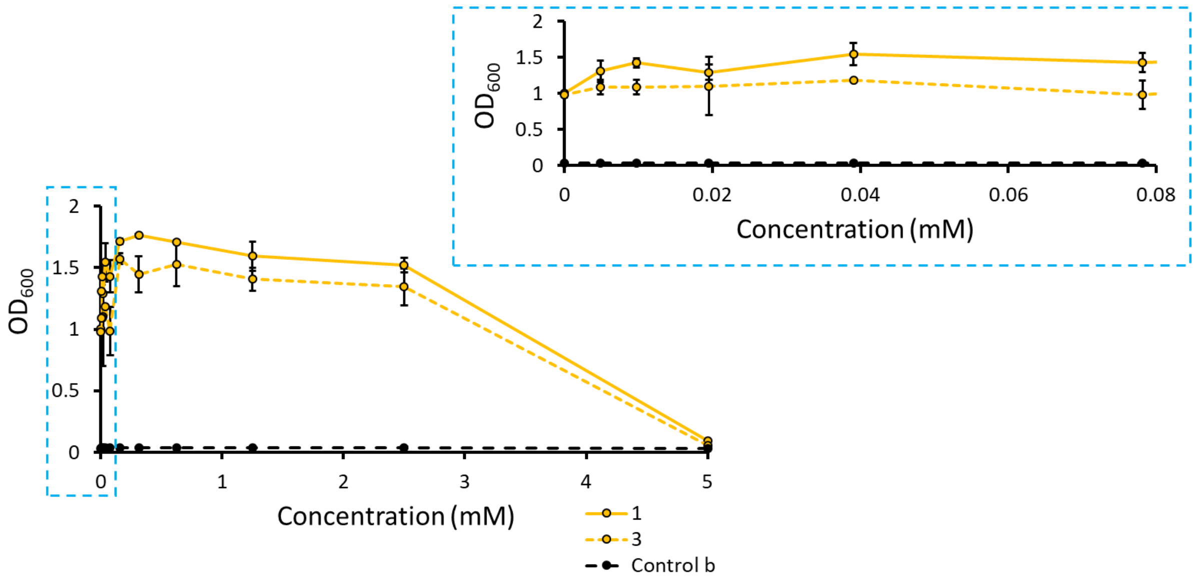

Figure S196 – Comparison of OD_{600} readings of **1** (solid yellow line) and **3** (dashed yellow line) at increasing concentrations in the presence of ATCC 17978 (*A. baumannii*), created from an average of two biological repeats, each containing three technical repeats. Control b (black dashed line) = absence of bacteria, outlined in blue dashed line = enlarged area.

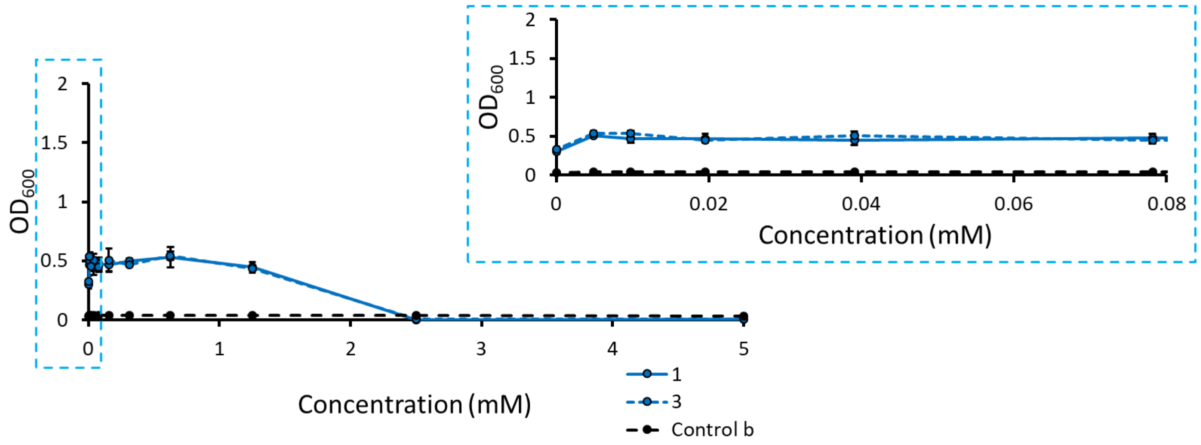

Figure S197 – Comparison of OD_{600} readings of **1** (solid blue line) and **3** (dashed blue line) at increasing concentrations in the presence of ATCC 9144 (*S. aureus*), created from an average of two biological repeats, each containing three technical repeats. Control b (black dashed line) = absence of bacteria, outlined in light blue dashed line = enlarged area.

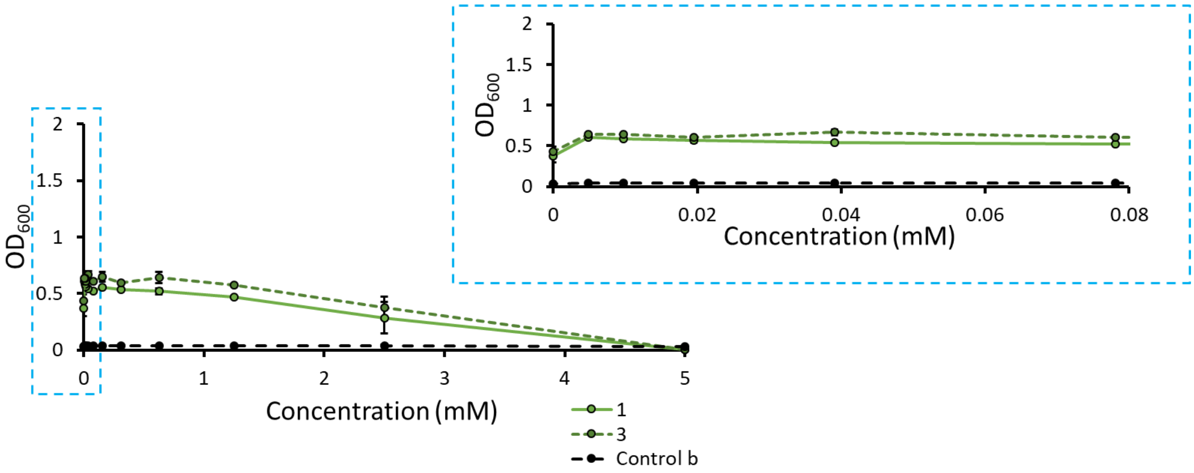

Figure S198 – Comparison of OD$_{600}$ readings of **1** (solid green line) and **3** (dashed green line) at increasing concentrations in the presence of NCTC 775 (*E. faecalis*), created from an average of two biological repeats, each containing three technical repeats. Control b (black dashed line) = absence of bacteria, outlined in blue dashed line = enlarged area.

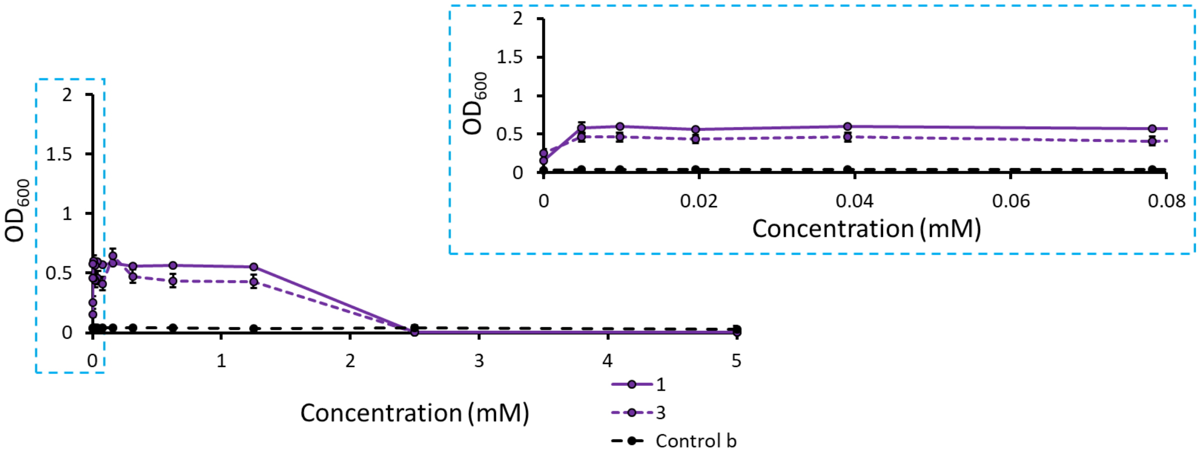

Figure S199 – Comparison of OD$_{600}$ readings of **1** (solid purple line) and **3** (dashed purple line) at increasing concentrations in the presence of NCTC 12204 (*E. faecium*), created from an average of two biological repeats, each containing three technical repeats. Control b (black dashed line) = absence of bacteria, outlined in blue dashed line = enlarged area.

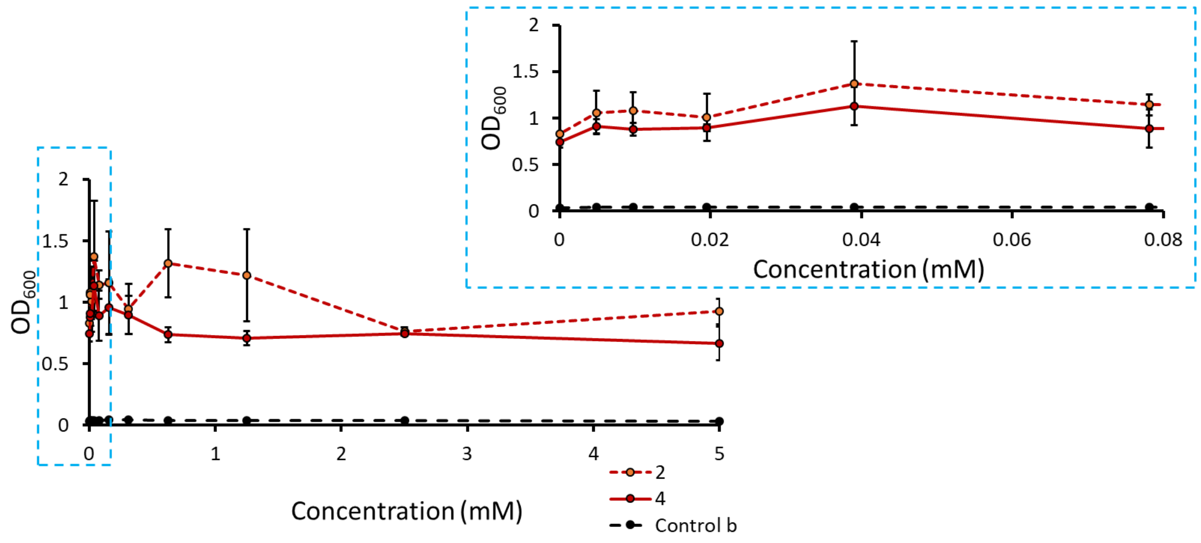

Figure S200 – Comparison of OD_{600} readings of **2** (dashed red line) and **4** (solid red line) at increasing concentrations in the presence of PAO1 (*P.aeruginosa*), created from an average of two biological repeats, each containing three technical repeats. Control b (black dashed line) = absence of bacteria, outlined in blue dashed line = enlarged area.

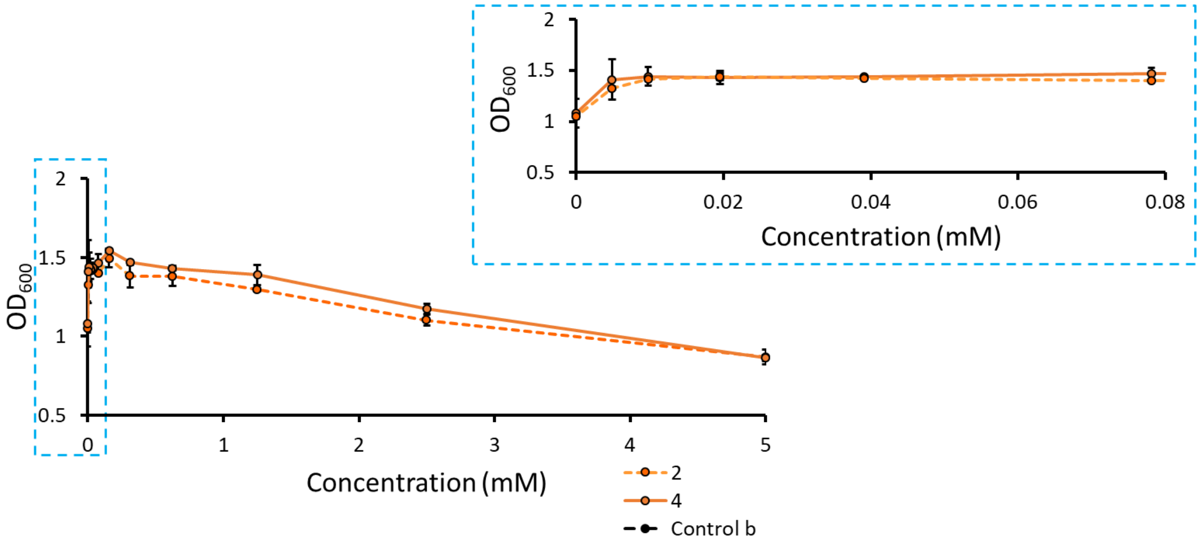

Figure S201 – Comparison of OD_{600} readings of **2** (dashed orange line) and **4** (solid orange line) at increasing concentrations in the presence of M6 (*K. pneumoniae*), created from an average of two biological repeats, each containing three technical repeats. Control b (black dashed line) = absence of bacteria, outlined in blue dashed line = enlarged area.

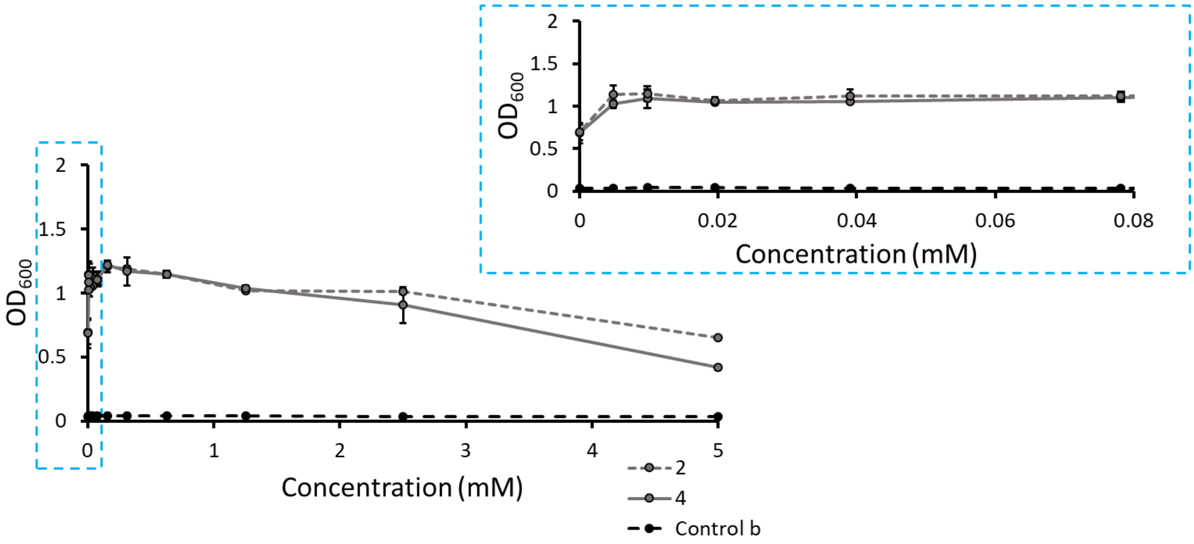

Figure S202 – Comparison of OD_{600} readings of **2** (dashed grey line) and **4** (solid grey line) at increasing concentrations in the presence of NCTC 12923 (*E. coli*), created from an average of two biological repeats, each containing three technical repeats. Control b (black dashed line) = absence of bacteria, outlined in blue dashed line = enlarged area.

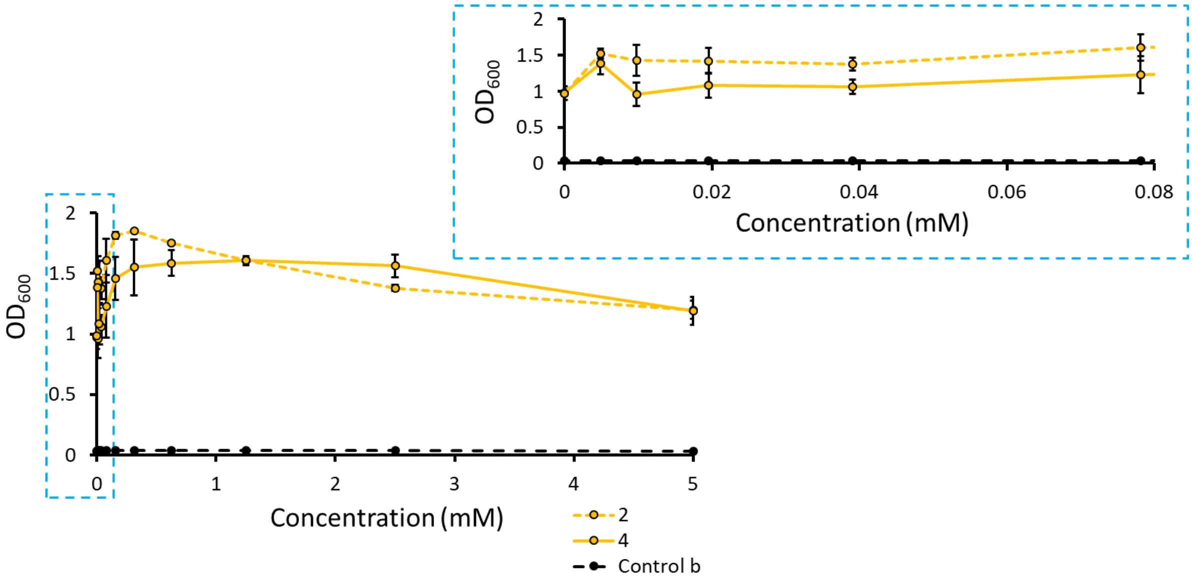

Figure S203 – Comparison of OD_{600} readings of **2** (dashed yellow line) and **4** (solid yellow line) at increasing concentrations in the presence of ATCC 17978 (*A. baumannii*), created from an average of two biological repeats, each containing three technical repeats. Control b (black dashed line) = absence of bacteria, outlined in blue dashed line = enlarged area.

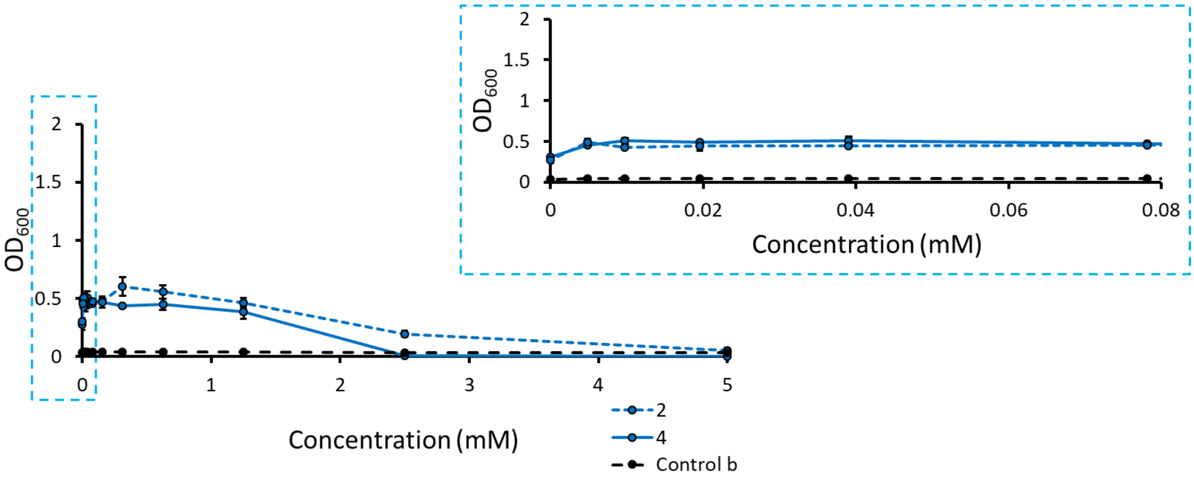

Figure S204 – Comparison of OD_{600} readings of **2** (dashed blue line) and **4** (solid blue line) at increasing concentrations in the presence of ATCC 9144 (*S. aureus*), created from an average of two biological repeats, each containing three technical repeats. Control b (black dashed line) = absence of bacteria, outlined in light blue dashed line = enlarged area.

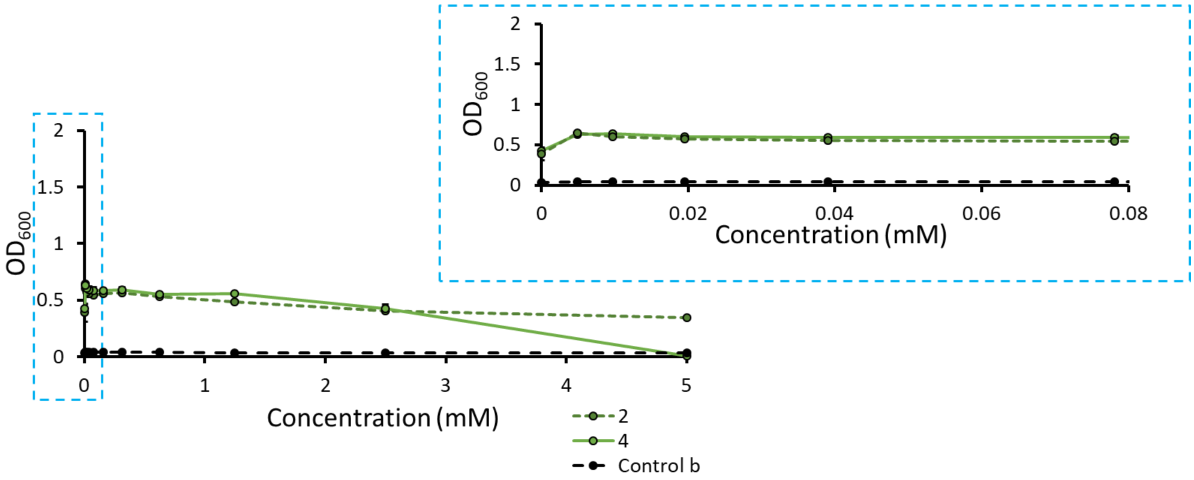

Figure S205 – Comparison of OD_{600} readings of **2** (solid green line) and **4** (dashed green line) at increasing concentrations in the presence of NCTC 775 (*E. faecalis*), created from an average of two biological repeats, each containing three technical repeats. Control b (black dashed line) = absence of bacteria, outlined in blue dashed line = enlarged area.

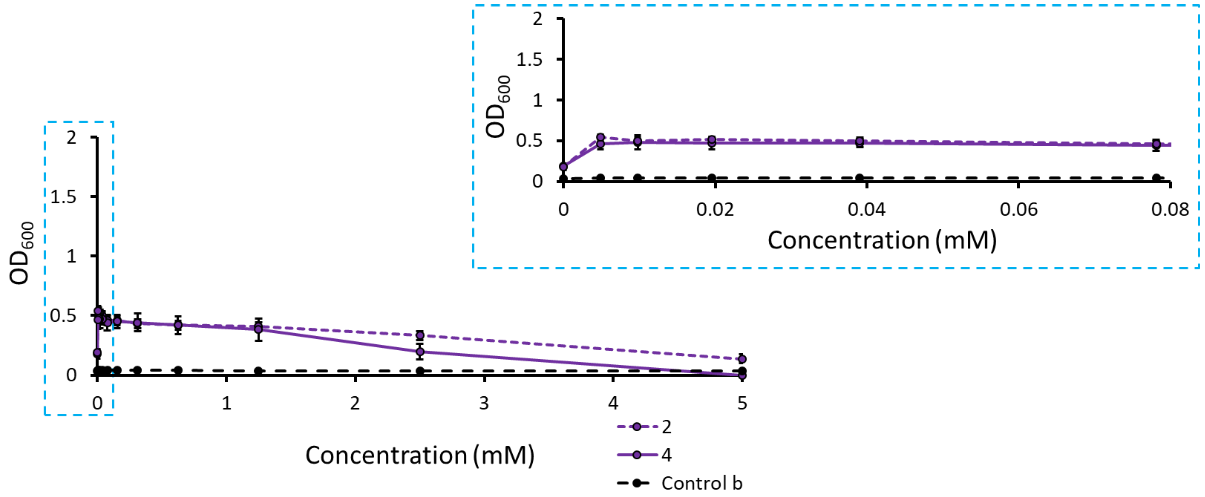

Figure S206 – Comparison of OD_{600} readings of **2** (dashed purple line) and **4** (solid purple line) at increasing concentrations in the presence of NCTC 12204 (*E. faecium*), created from an average of two biological repeats, each containing three technical repeats. Control b (black dashed line) = absence of bacteria, outlined in blue dashed line = enlarged area.

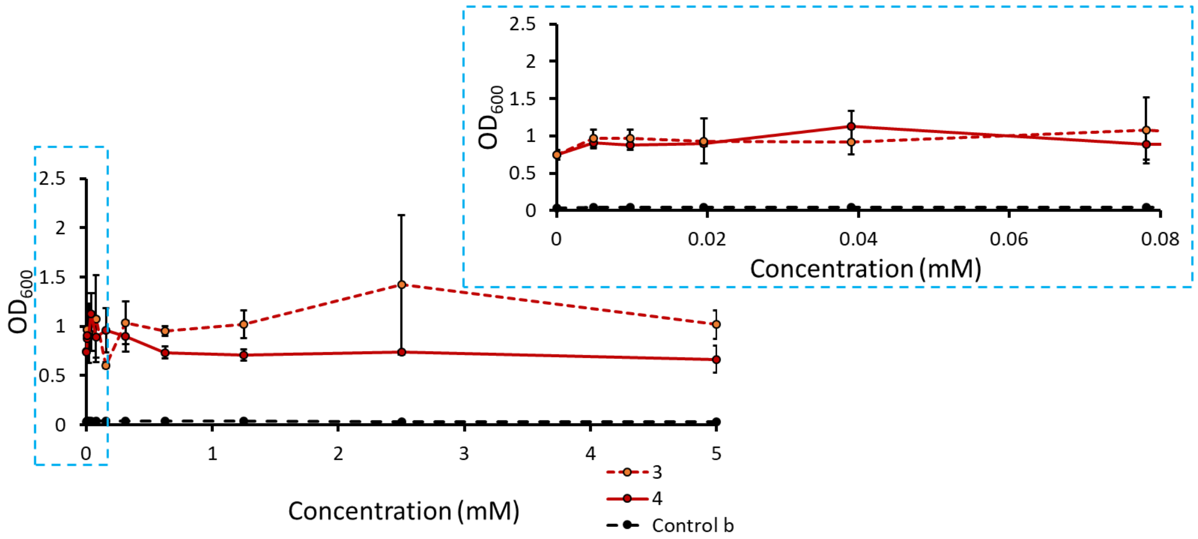

Figure S207 – Comparison of OD_{600} readings of **2** (dashed red line) and **4** (solid red line) at increasing concentrations in the presence of PAO1 (*P.aeruginosa*), created from an average of two biological repeats, each containing three technical repeats. Control b (black dashed line) = absence of bacteria, outlined in blue dashed line = enlarged area.

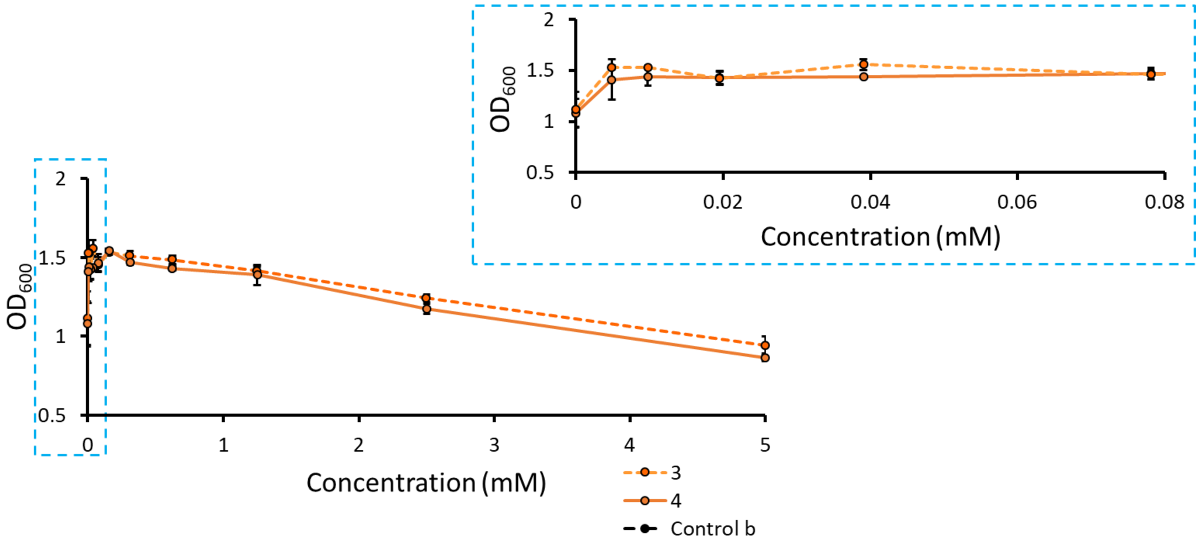

Figure S208 – Comparison of OD_{600} readings of **3** (dashed orange line) and **4** (solid orange line) at increasing concentrations in the presence of M6 (*K. pneumoniae*), created from an average of two biological repeats, each containing three technical repeats. Control b (black dashed line) = absence of bacteria, outlined in blue dashed line = enlarged area.

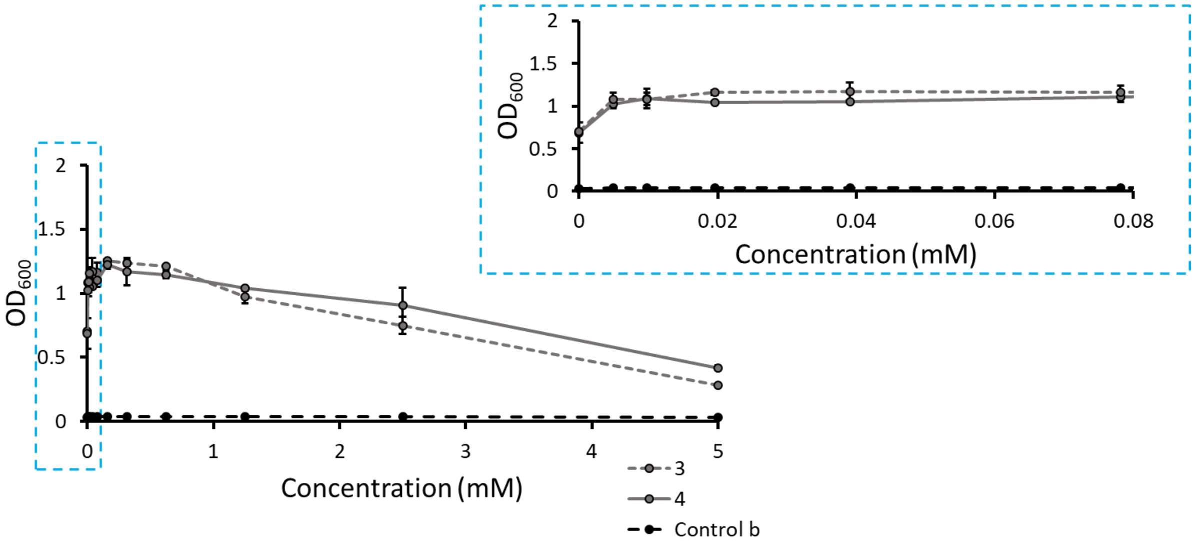

Figure S209 – Comparison of OD_{600} readings of **3** (dashed grey line) and **4** (solid grey line) at increasing concentrations in the presence of NCTC 12923 (*E. coli*), created from an average of two biological repeats, each containing three technical repeats. Control b (black dashed line) = absence of bacteria, outlined in blue dashed line = enlarged area.

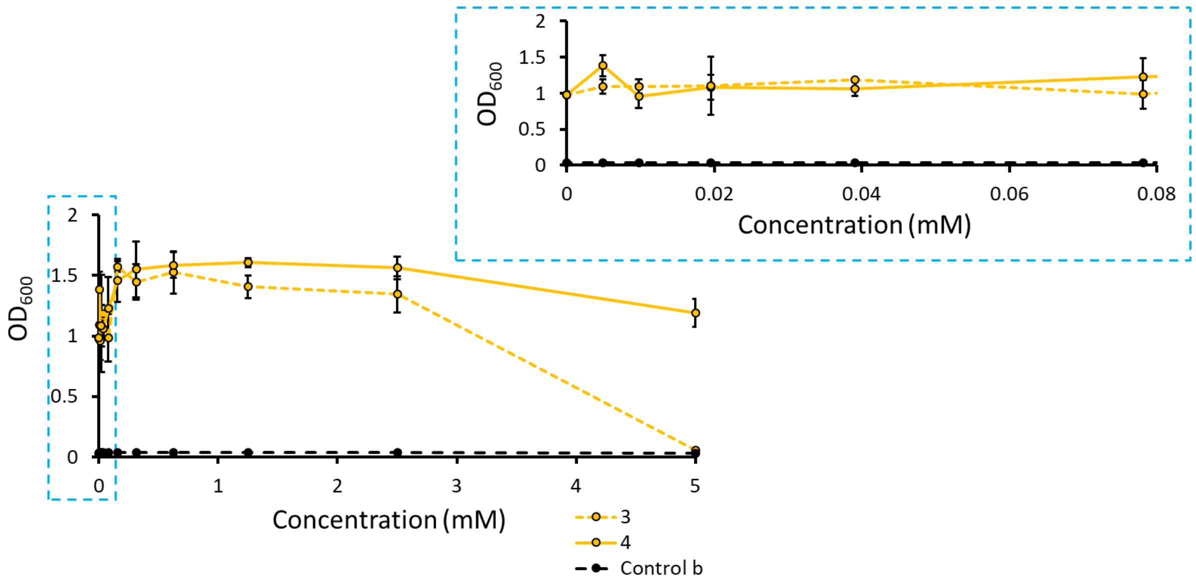

Figure S210 – Comparison of OD_{600} readings of **3** (dashed yellow line) and **4** (solid yellow line) at increasing concentrations in the presence of ATCC 17978 (*A. baumannii*), created from an average of two biological repeats, each containing three technical repeats. Control b (black dashed line) = absence of bacteria, outlined in blue dashed line = enlarged area.

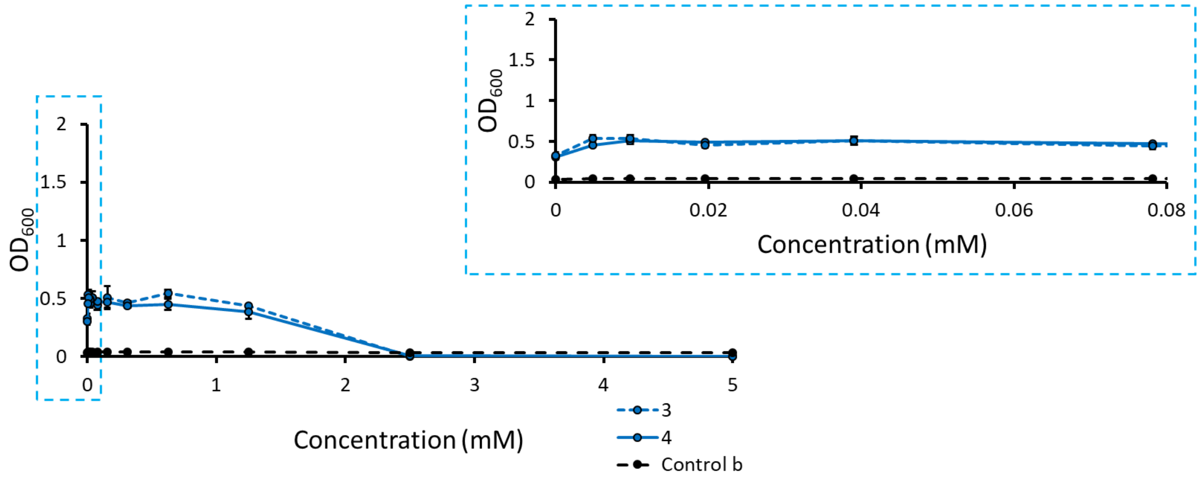

Figure S211 – Comparison of OD_{600} readings of **3** (dashed blue line) and **4** (solid blue line) at increasing concentrations in the presence of ATCC 9144 (*S. aureus*), created from an average of two biological repeats, each containing three technical repeats. Control b (black dashed line) = absence of bacteria, outlined in light blue dashed line = enlarged area.

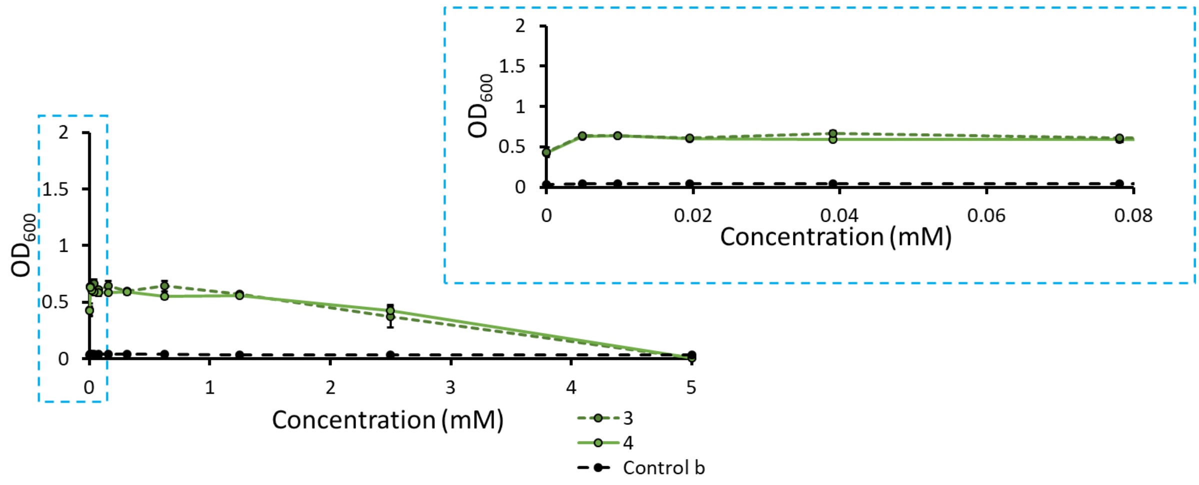

Figure S212 – Comparison of OD_{600} readings of **3** (solid green line) and **4** (dashed green line) at increasing concentrations in the presence of NCTC 775 (*E. faecalis*), created from an average of two biological repeats, each containing three technical repeats. Control b (black dashed line) = absence of bacteria, outlined in blue dashed line = enlarged area.

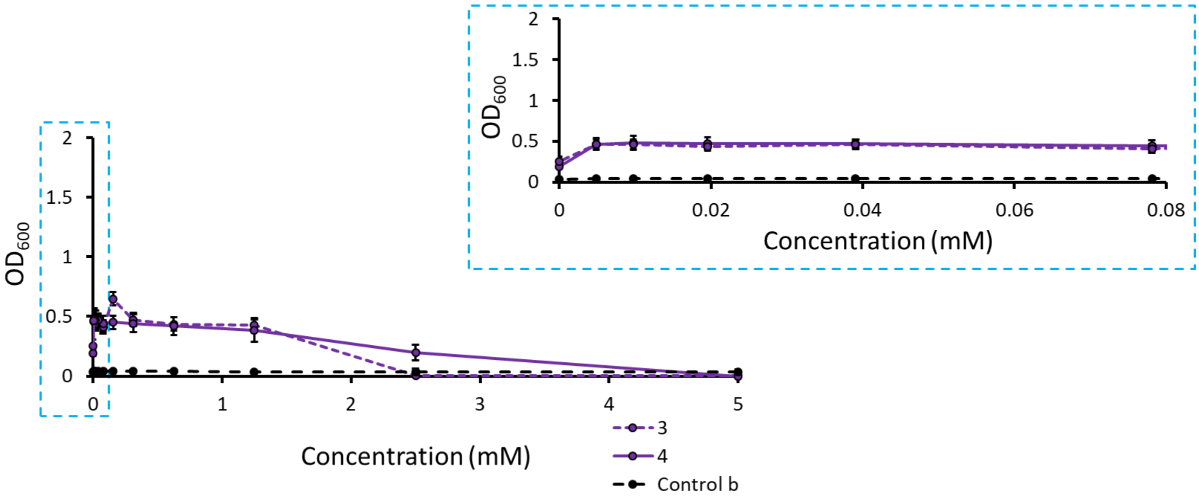

Figure S213 – Comparison of OD_{600} readings of **3** (dashed purple line) and **4** (solid purple line) at increasing concentrations in the presence of NCTC 12204 (*E. faecium*), created from an average of two biological repeats, each containing three technical repeats. Control b (black dashed line) = absence of bacteria, outlined in blue dashed line = enlarged area.

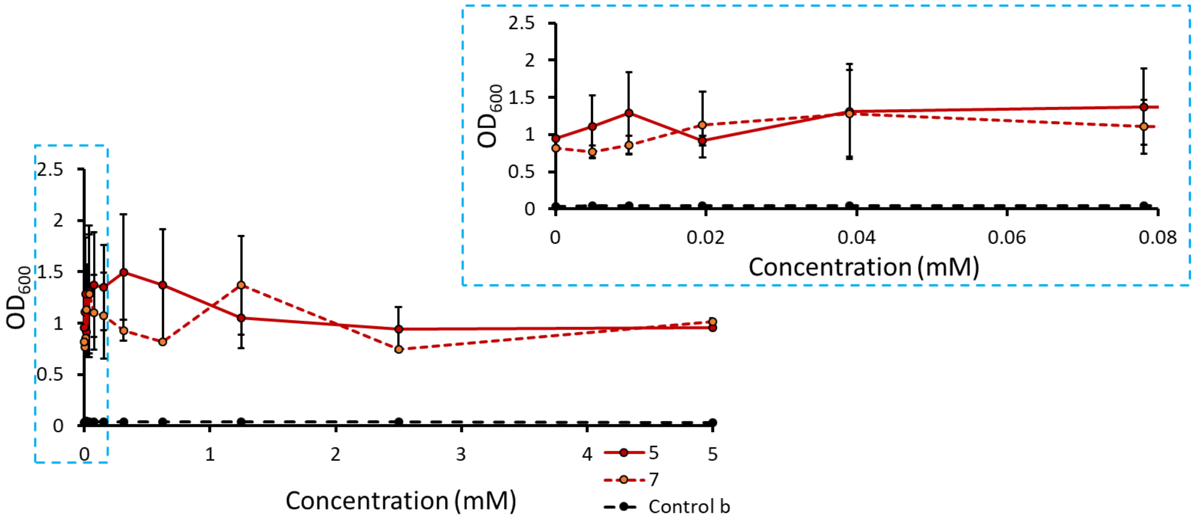

Figure S214 – Comparison of OD_{600} readings of **5** (solid red line) and **7** (dashed red line) at increasing concentrations in the presence of PAO1 (*P.aeruginosa*), created from an average of two biological repeats, each containing three technical repeats. Control b (black dashed line) = absence of bacteria, outlined in blue dashed line = enlarged area.

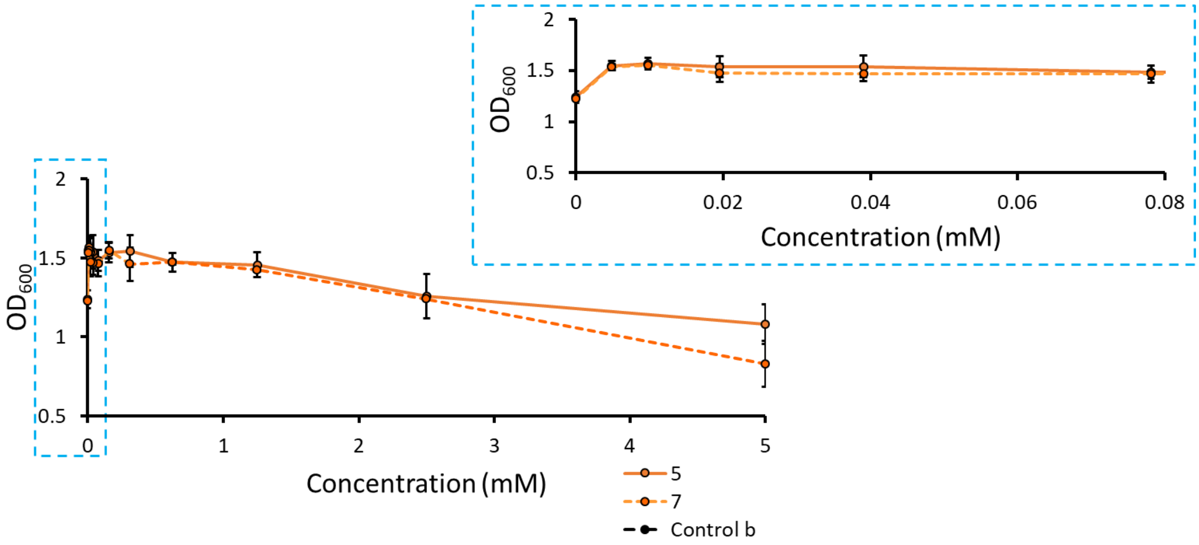

Figure S215 – Comparison of OD_{600} readings of **5** (solid orange line) and **7** (dashed orange line) at increasing concentrations in the presence of M6 (*K. pneumoniae*), created from an average of two biological repeats, each containing three technical repeats. Control b (black dashed line) = absence of bacteria, outlined in blue dashed line = enlarged area.

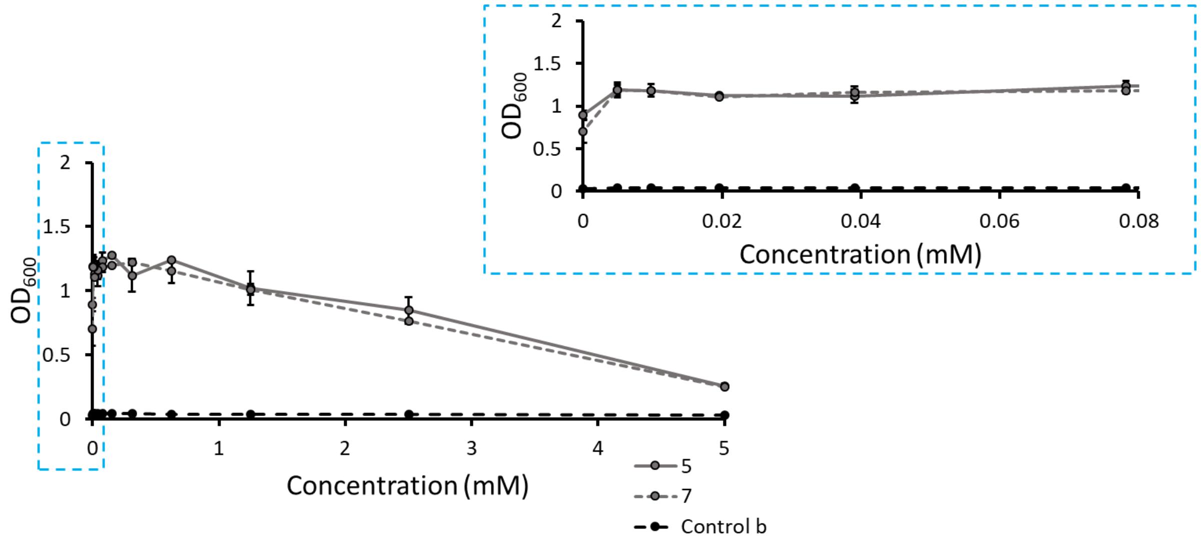

Figure S216 – Comparison of OD_{600} readings of **5** (solid grey line) and **7** (dashed grey line) at increasing concentrations in the presence of NCTC 12923 (*E. coli*), created from an average of two biological repeats, each containing three technical repeats. Control b (black dashed line) = absence of bacteria, outlined in blue dashed line = enlarged area.

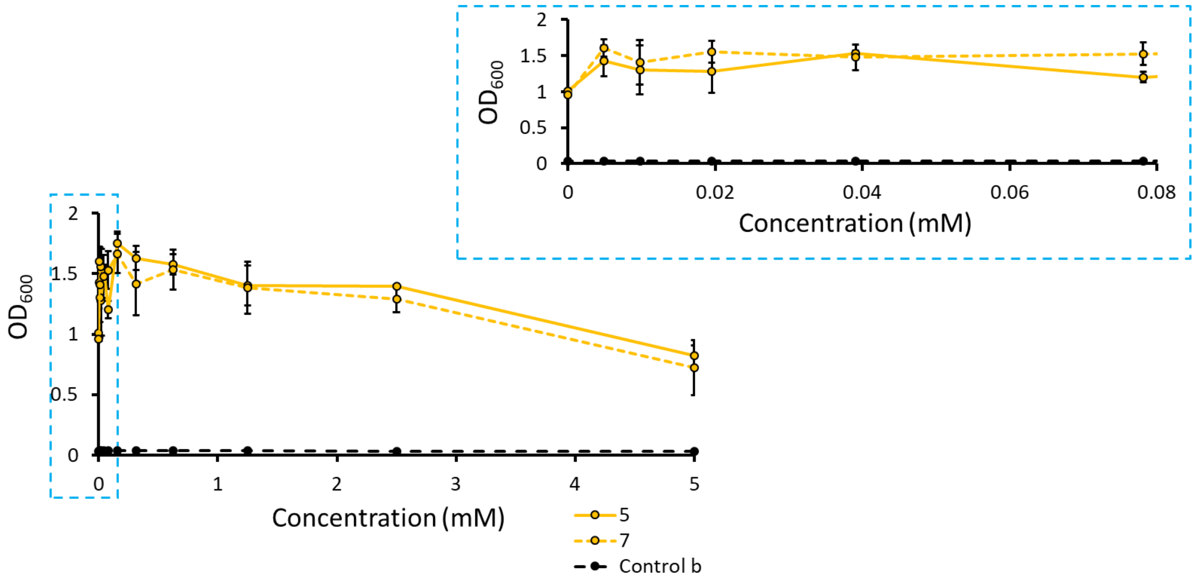

Figure S217 – Comparison of OD_{600} readings of **5** (solid yellow line) and **7** (dashed yellow line) at increasing concentrations in the presence of ATCC 17978 (*A. baumannii*), created from an average of two biological repeats, each containing three technical repeats. Control b (black dashed line) = absence of bacteria, outlined in blue dashed line = enlarged area.

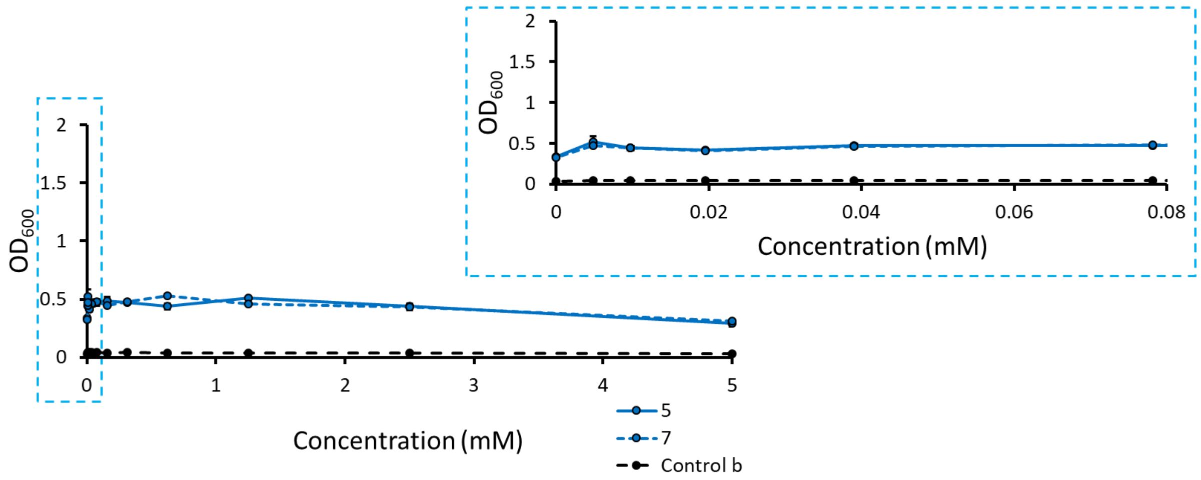

Figure S218 – Comparison of OD_{600} readings of **5** (solid blue line) and **7** (dashed blue line) at increasing concentrations in the presence of ATCC 9144 (*S. aureus*), created from an average of two biological repeats, each containing three technical repeats. Control b (black dashed line) = absence of bacteria, outlined in light blue dashed line = enlarged area.

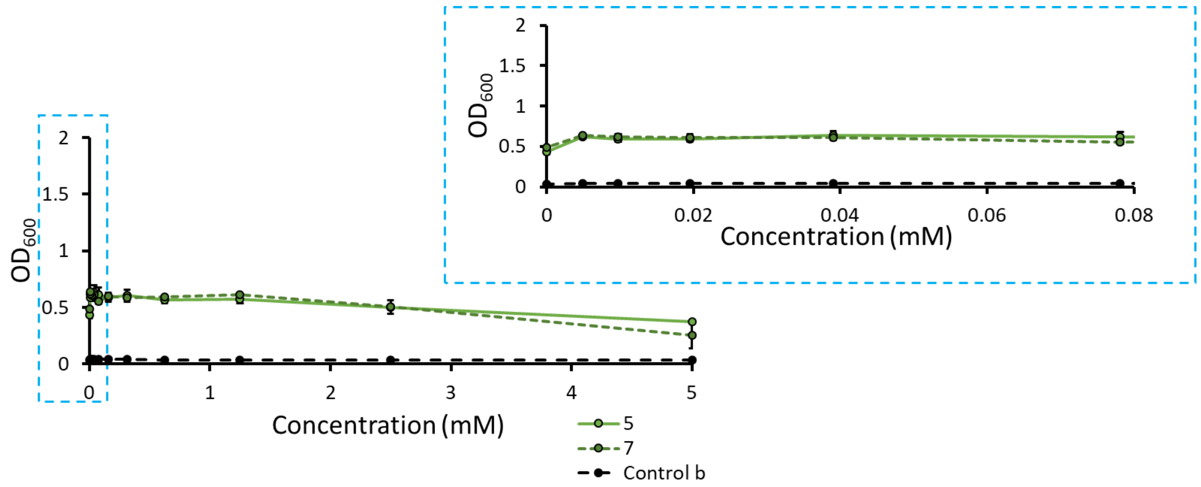

Figure S219 – Comparison of OD_{600} readings of **5** (solid green line) and **7** (dashed green line) at increasing concentrations in the presence of NCTC 775 (*E. faecalis*), created from an average of two biological repeats, each containing three technical repeats. Control b (black dashed line) = absence of bacteria, outlined in blue dashed line = enlarged area.

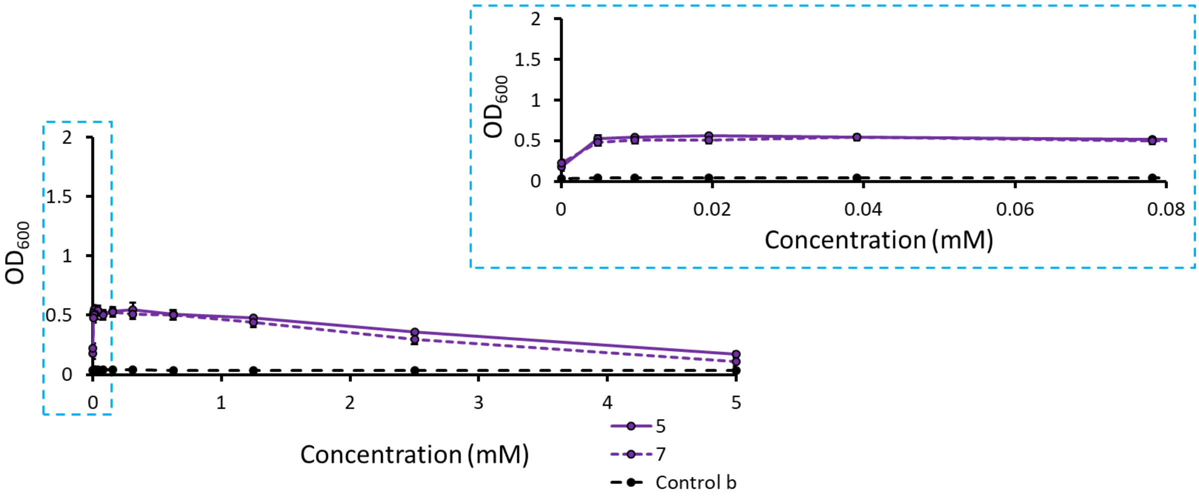

Figure S220 – Comparison of OD_{600} readings of **5** (solid purple line) and **7** (dashed purple line) at increasing concentrations in the presence of NCTC 12204 (*E. faecium*), created from an average of two biological repeats, each containing three technical repeats. Control b (black dashed line) = absence of bacteria, outlined in blue dashed line = enlarged area.

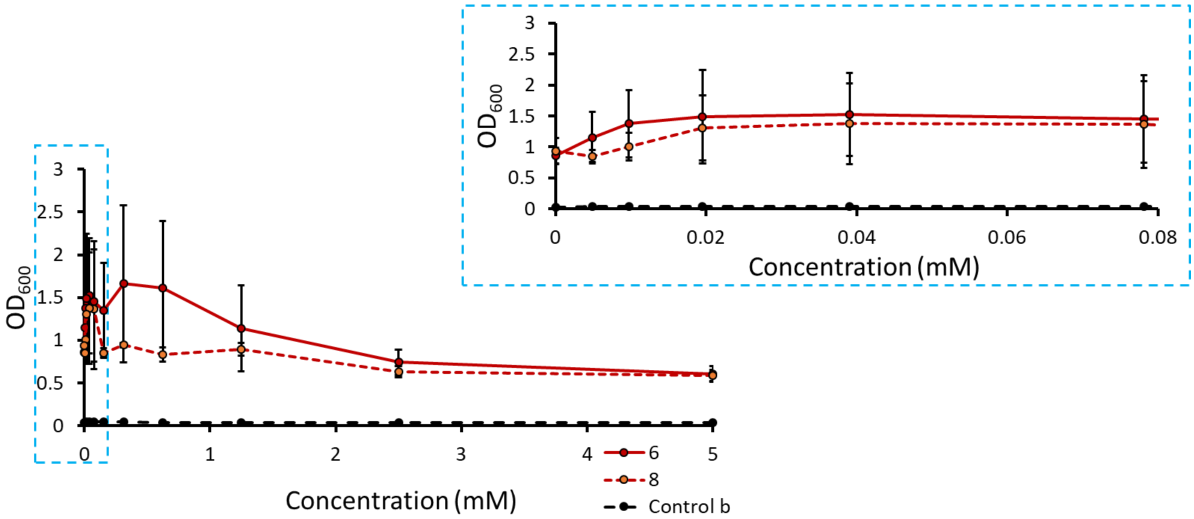

Figure S221 – Comparison of OD_{600} readings of **6** (solid red line) and **8** (dashed red line) at increasing concentrations in the presence of PAO1 (*P.aeruginosa*), created from an average of two biological repeats, each containing three technical repeats. Control b (black dashed line) = absence of bacteria, outlined in blue dashed line = enlarged area.

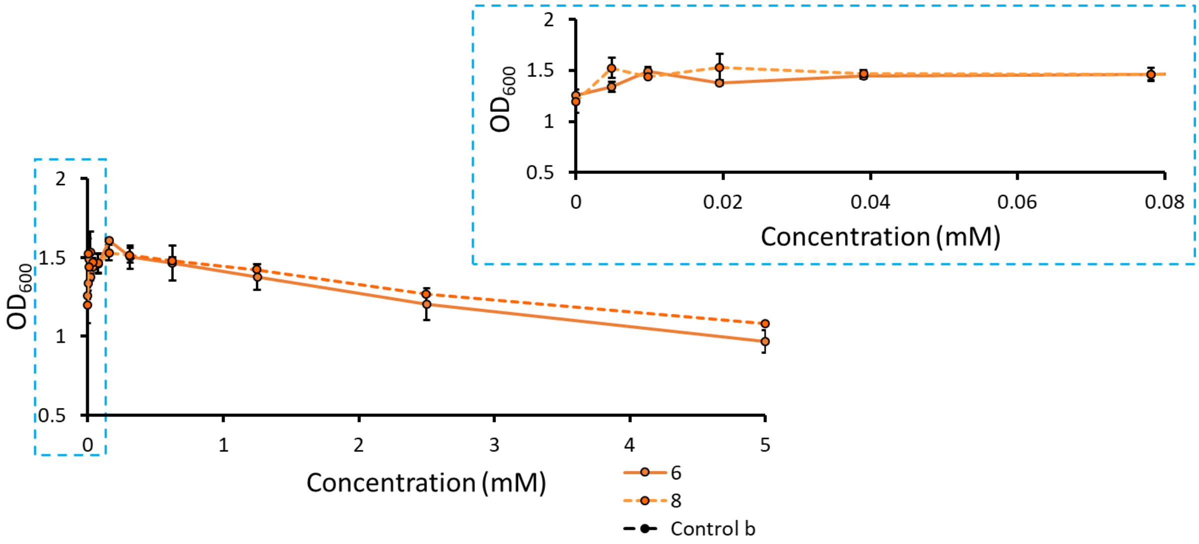

Figure S222 – Comparison of OD_{600} readings of **6** (solid orange line) and **8** (dashed orange line) at increasing concentrations in the presence of M6 (*K. pneumoniae*), created from an average of two biological repeats, each containing three technical repeats. Control b (black dashed line) = absence of bacteria, outlined in blue dashed line = enlarged area.

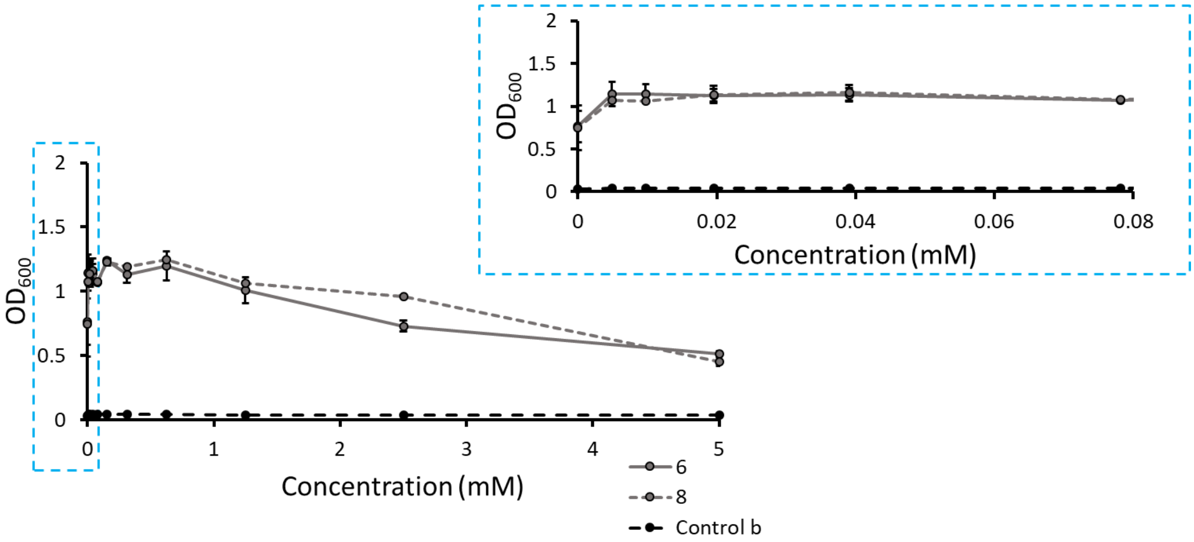

Figure S223 – Comparison of OD_{600} readings of **6** (solid grey line) and **8** (dashed grey line) at increasing concentrations in the presence of NCTC 12923 (*E. coli*), created from an average of two biological repeats, each containing three technical repeats. Control b (black dashed line) = absence of bacteria, outlined in blue dashed line = enlarged area.

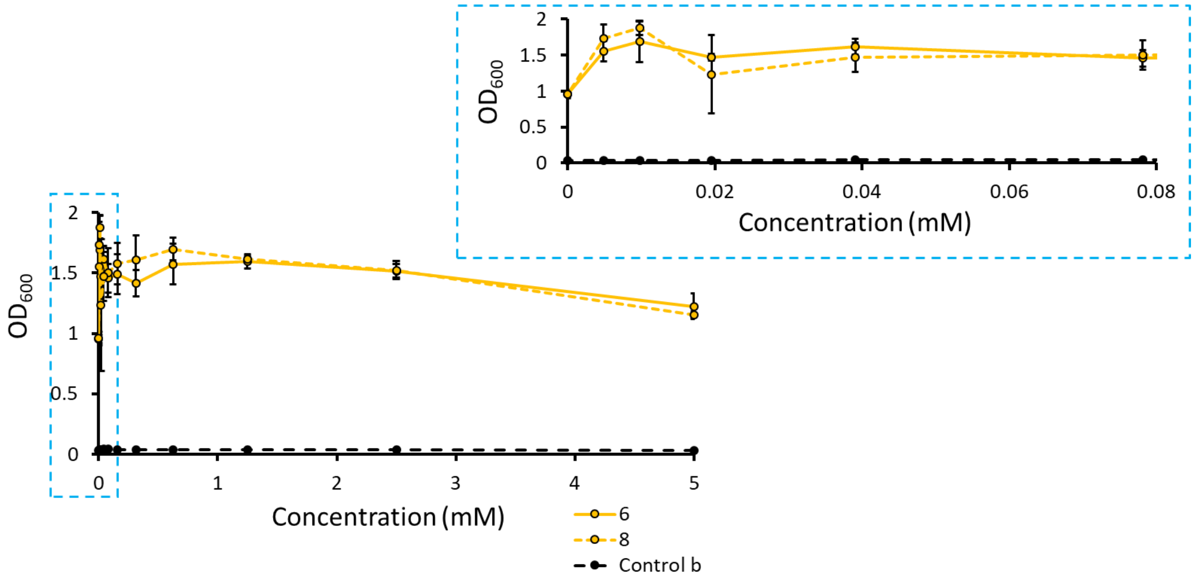

Figure S224 – Comparison of OD_{600} readings of **6** (solid yellow line) and **8** (dashed yellow line) at increasing concentrations in the presence of ATCC 17978 (*A. baumannii*), created from an average of two biological repeats, each containing three technical repeats. Control b (black dashed line) = absence of bacteria, outlined in blue dashed line = enlarged area.

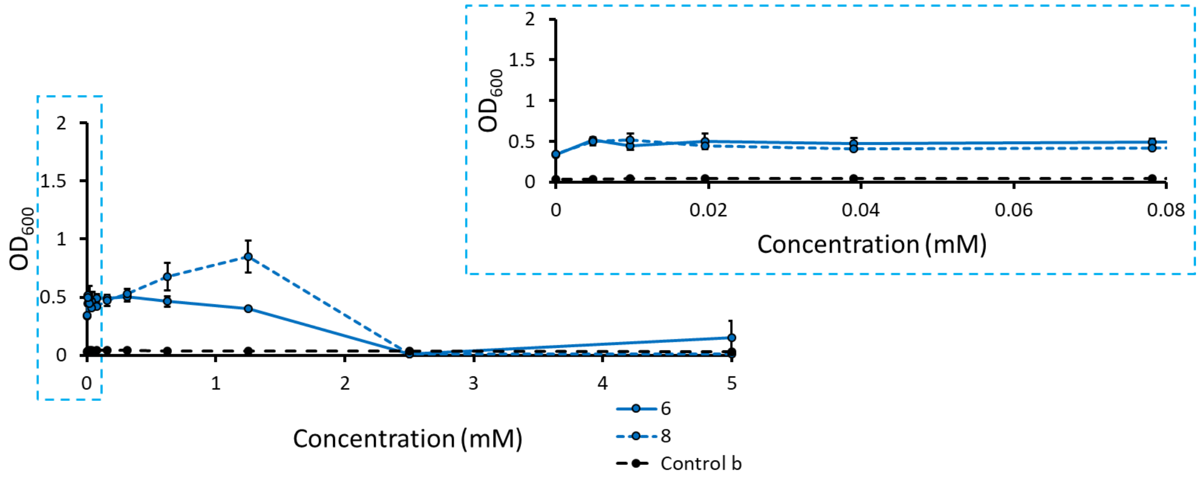

Figure S225 – Comparison of OD_{600} readings of **6** (solid blue line) and **8** (dashed blue line) at increasing concentrations in the presence of ATCC 9144 (*S. aureus*), created from an average of two biological repeats, each containing three technical repeats. Control b (black dashed line) = absence of bacteria, outlined in light blue dashed line = enlarged area.

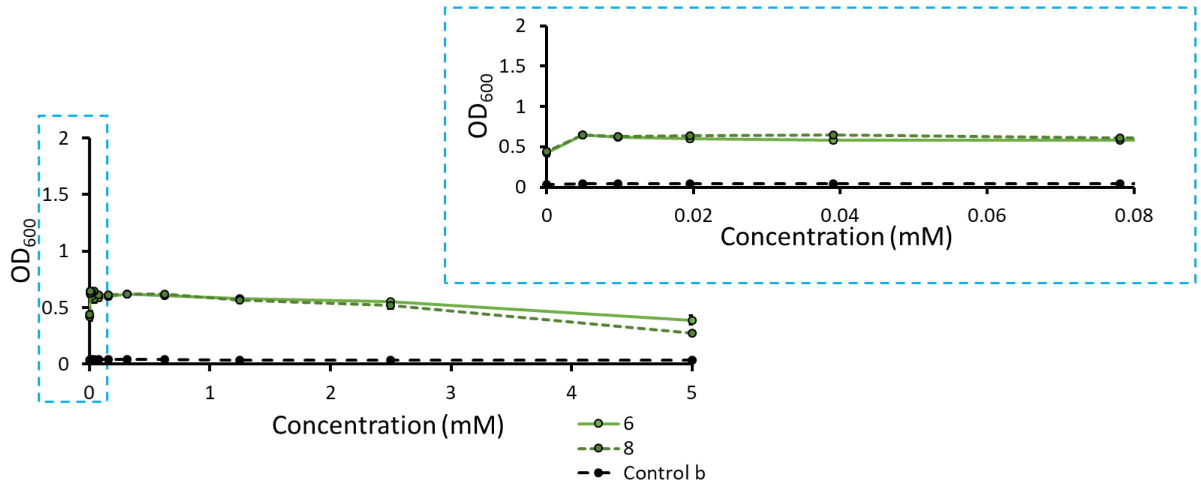

Figure S226 – Comparison of OD_{600} readings of **6** (solid green line) and **8** (dashed green line) at increasing concentrations in the presence of NCTC 775 (*E. faecalis*), created from an average of two biological repeats, each containing three technical repeats. Control b (black dashed line) = absence of bacteria, outlined in blue dashed line = enlarged area.

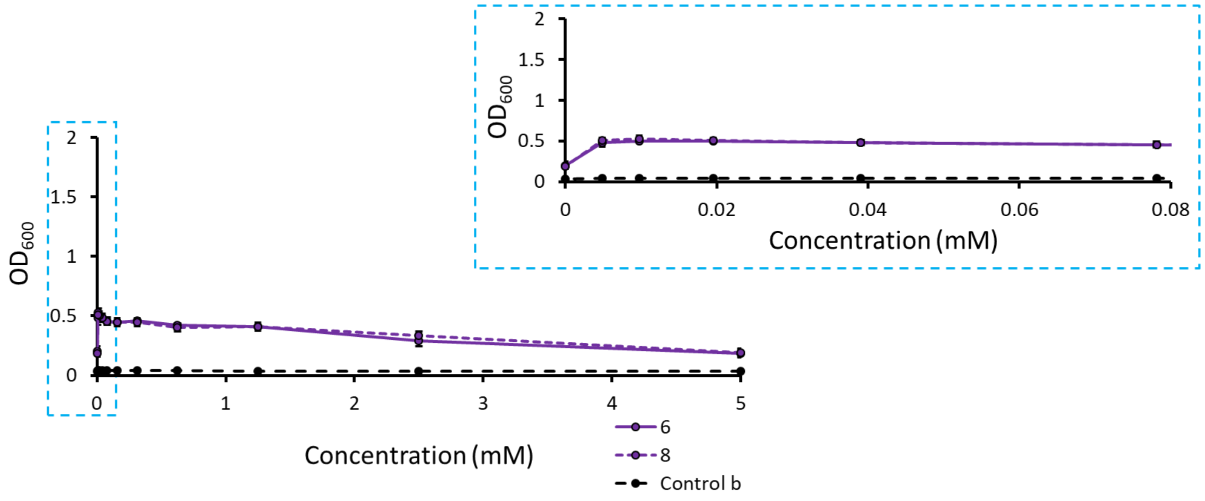

Figure S227 – Comparison of OD_{600} readings of **6** (solid purple line) and **8** (dashed purple line) at increasing concentrations in the presence of NCTC 12204 (*E. faecium*), created from an average of two biological repeats, each containing three technical repeats. Control b (black dashed line) = absence of bacteria, outlined in blue dashed line = enlarged area.

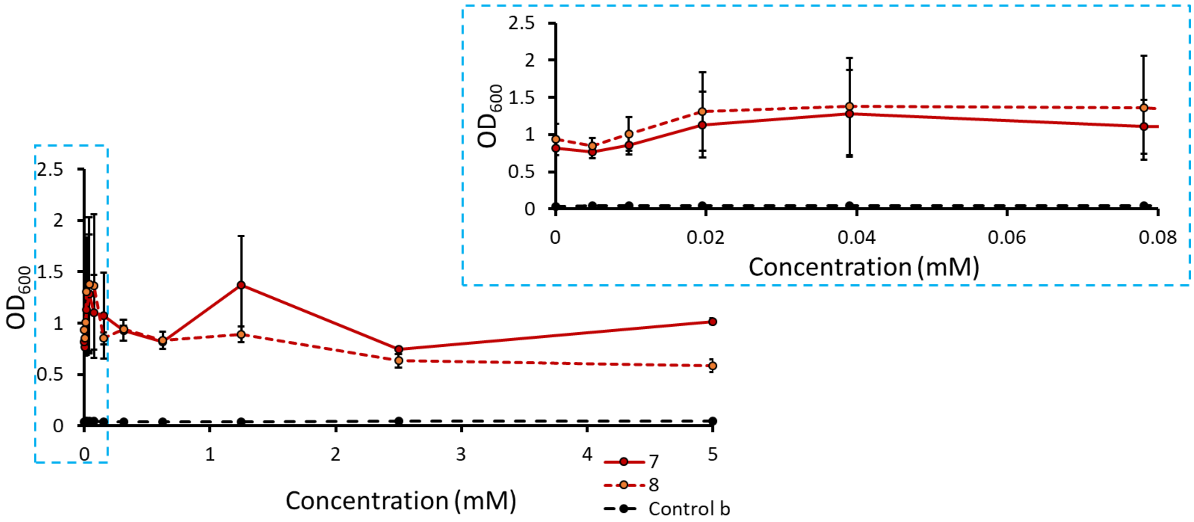

Figure S228 – Comparison of OD_{600} readings of **7** (solid red line) and **8** (dashed red line) at increasing concentrations in the presence of PAO1 (*P.aeruginosa*), created from an average of two biological repeats, each containing three technical repeats. Control b (black dashed line) = absence of bacteria, outlined in blue dashed line = enlarged area.

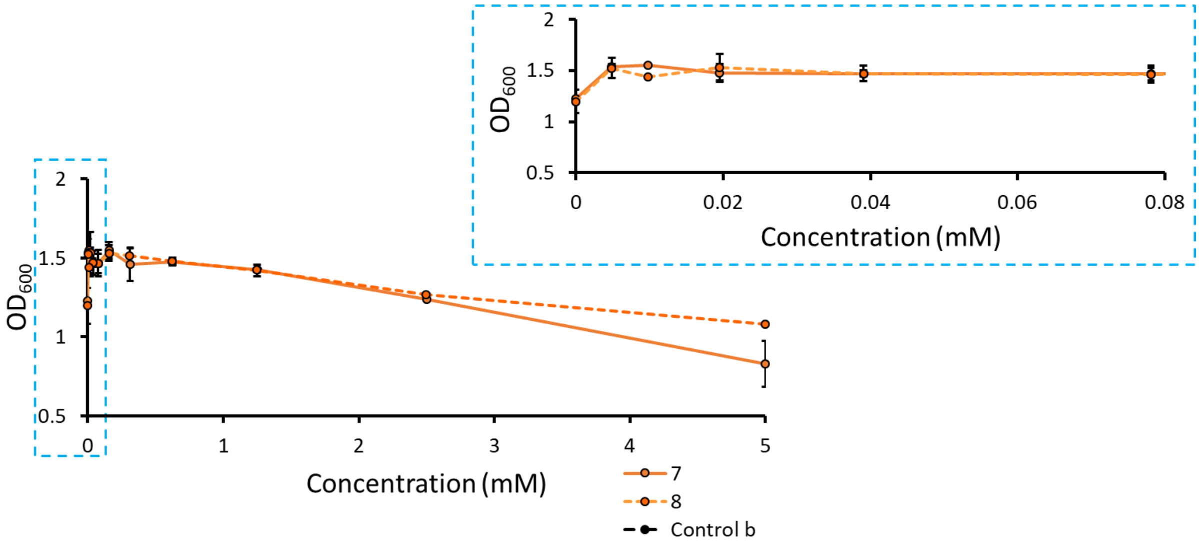

Figure S229 – Comparison of OD_{600} readings of **7** (solid orange line) and **8** (dashed orange line) at increasing concentrations in the presence of M6 (*K. pneumoniae*), created from an average of two biological repeats, each containing three technical repeats. Control b (black dashed line) = absence of bacteria, outlined in blue dashed line = enlarged area.

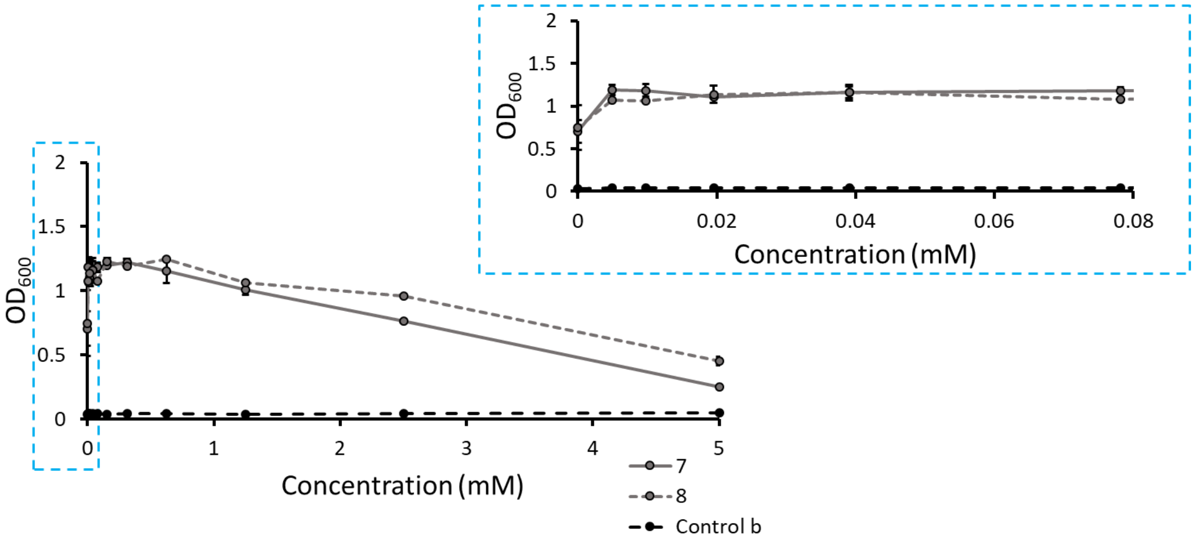

Figure S230 – Comparison of OD_{600} readings of **7** (solid grey line) and **8** (dashed grey line) at increasing concentrations in the presence of NCTC 12923 (*E. coli*), created from an average of two biological repeats, each containing three technical repeats. Control b (black dashed line) = absence of bacteria, outlined in blue dashed line = enlarged area.

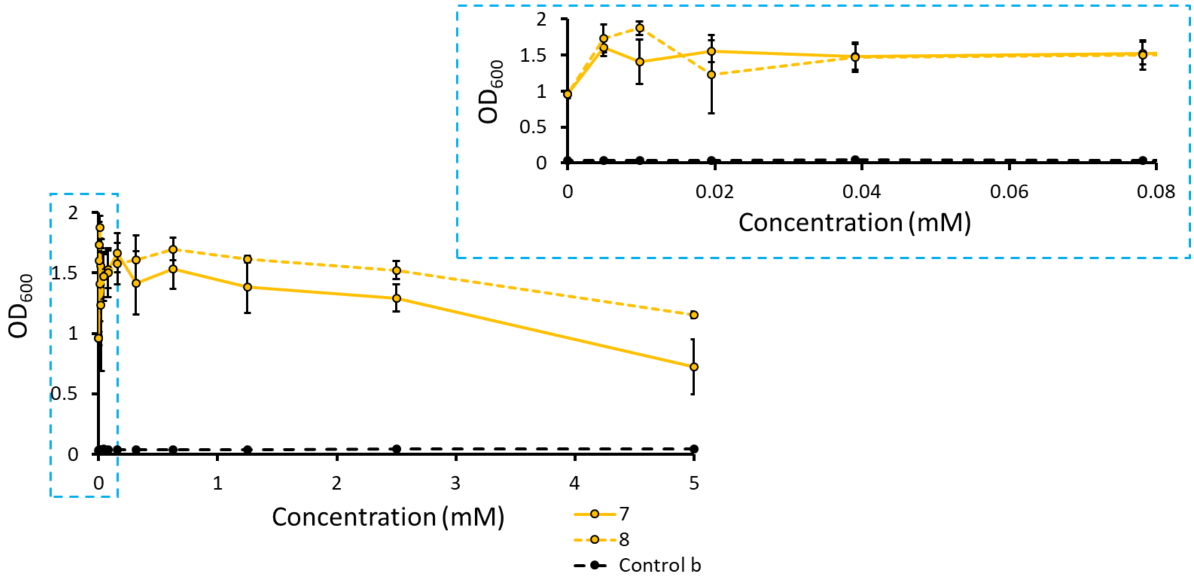

Figure S231 – Comparison of OD_{600} readings of **7** (solid yellow line) and **8** (dashed yellow line) at increasing concentrations in the presence of ATCC 17978 (*A. baumannii*), created from an average of two biological repeats, each containing three technical repeats. Control b (black dashed line) = absence of bacteria, outlined in blue dashed line = enlarged area.

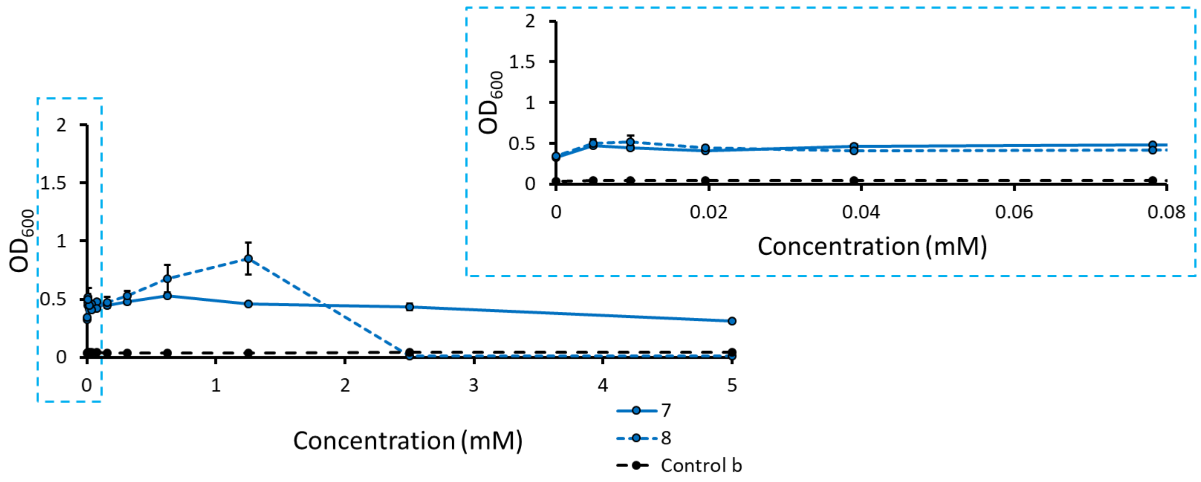

Figure S232 – Comparison of OD_{600} readings of **7** (solid blue line) and **8** (dashed blue line) at increasing concentrations in the presence of ATCC 9144 (*S. aureus*), created from an average of two biological repeats, each containing three technical repeats. Control b (black dashed line) = absence of bacteria, outlined in light blue dashed line = enlarged area.

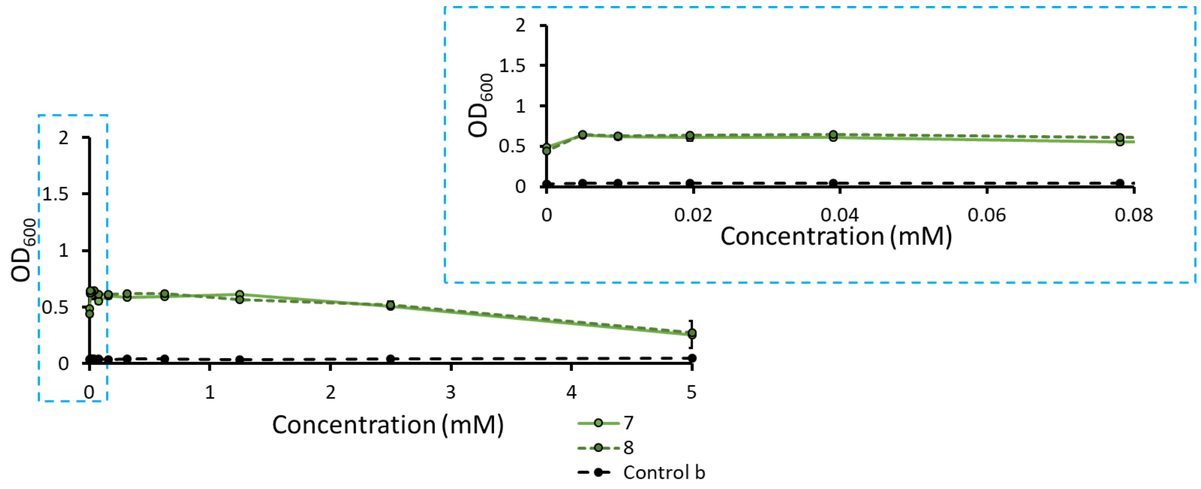

Figure S233 – Comparison of OD_{600} readings of **7** (solid green line) and **8** (dashed green line) at increasing concentrations in the presence of NCTC 775 (*E. faecalis*), created from an average of two biological repeats, each containing three technical repeats. Control b (black dashed line) = absence of bacteria, outlined in blue dashed line = enlarged area.

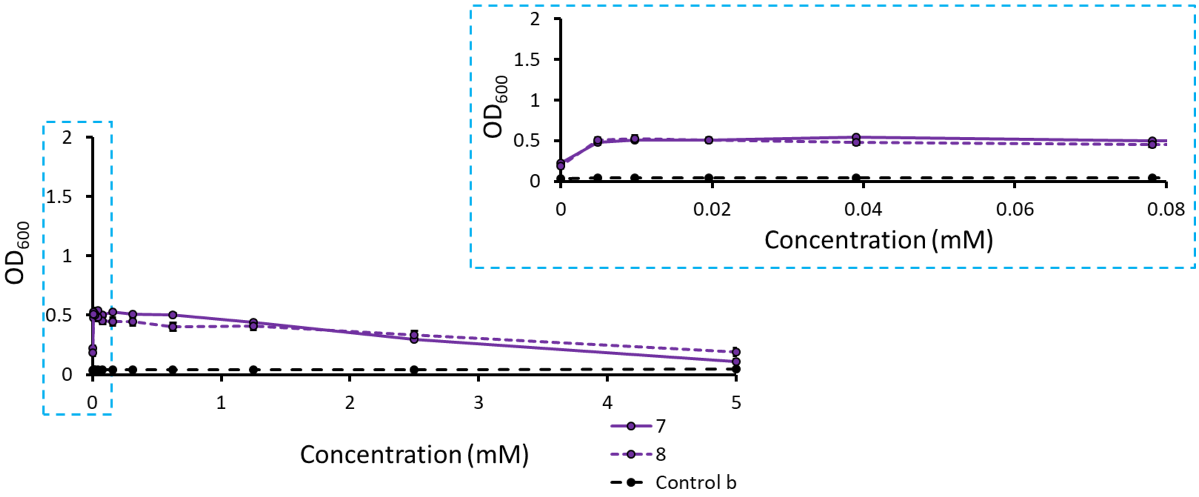

Figure S234 – Comparison of OD_{600} readings of **7** (solid purple line) and **8** (dashed purple line) at increasing concentrations in the presence of NCTC 12204 (*E. faecium*), created from an average of two biological repeats, each containing three technical repeats. Control b (black dashed line) = absence of bacteria, outlined in blue dashed line = enlarged area.

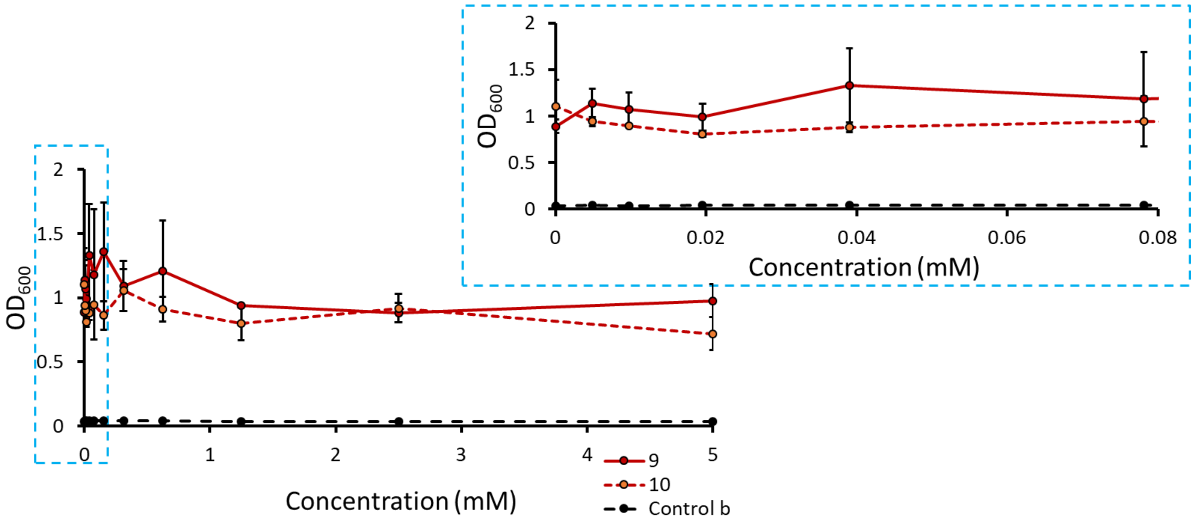

Figure S235 – Comparison of OD_{600} readings of **9** (solid red line) and **10** (dashed red line) at increasing concentrations in the presence of PAO1 (*P.aeruginosa*), created from an average of two biological repeats, each containing three technical repeats. Control b (black dashed line) = absence of bacteria, outlined in blue dashed line = enlarged area.

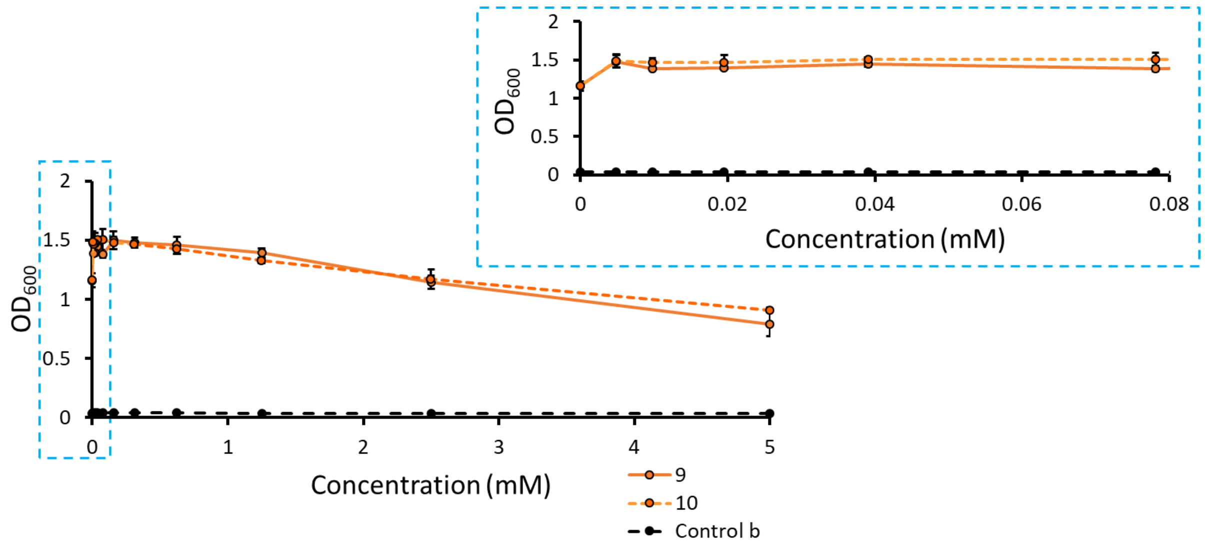

Figure S236 – Comparison of OD_{600} readings of **9** (solid orange line) and **10** (dashed orange line) at increasing concentrations in the presence of M6 (*K. pneumoniae*), created from an average of two biological repeats, each containing three technical repeats. Control b (black dashed line) = absence of bacteria, outlined in blue dashed line = enlarged area.

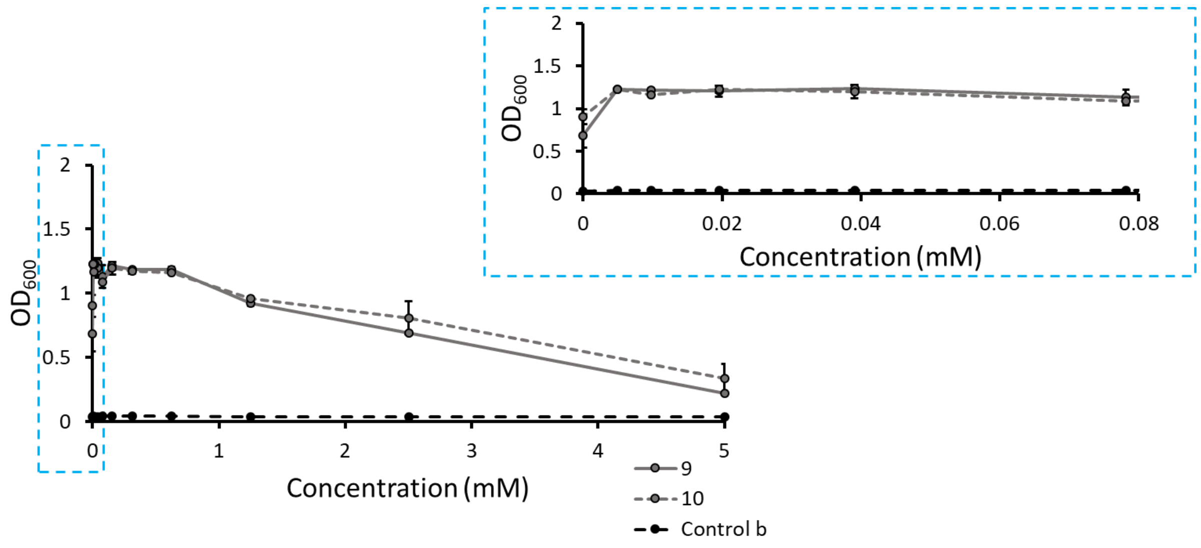

Figure S237 – Comparison of OD_{600} readings of **9** (solid grey line) and **10** (dashed grey line) at increasing concentrations in the presence of NCTC 12923 (*E. coli*), created from an average of two biological repeats, each containing three technical repeats. Control b (black dashed line) = absence of bacteria, outlined in blue dashed line = enlarged area.

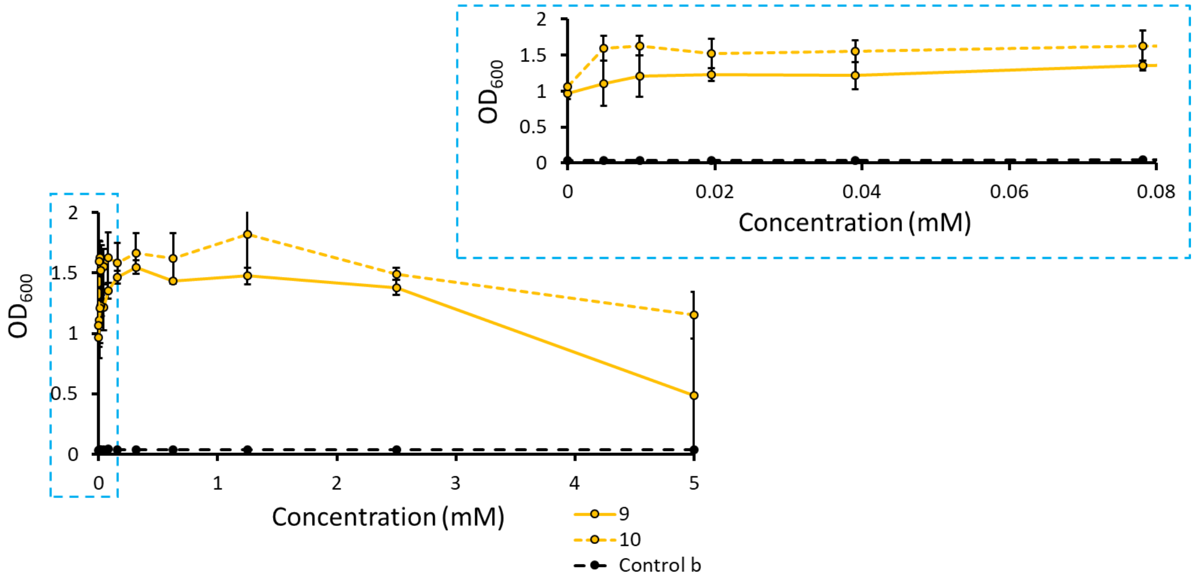

Figure S238 – Comparison of OD_{600} readings of **9** (solid yellow line) and **10** (dashed yellow line) at increasing concentrations in the presence of ATCC 17978 (*A. baumannii*), created from an average of two biological repeats, each containing three technical repeats. Control b (black dashed line) = absence of bacteria, outlined in blue dashed line = enlarged area.

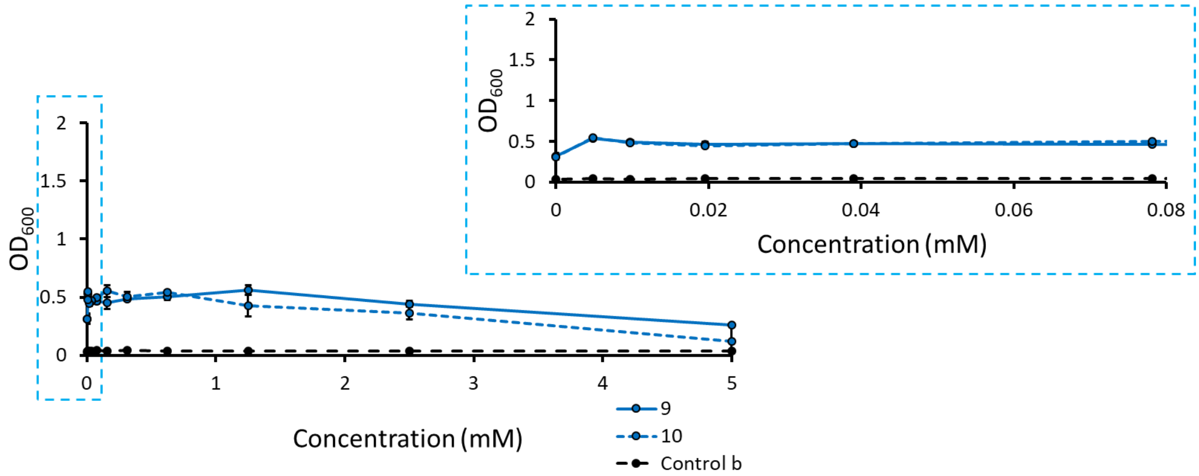

Figure S239 – Comparison of OD_{600} readings of **9** (solid blue line) and **10** (dashed blue line) at increasing concentrations in the presence of ATCC 9144 (*S. aureus*), created from an average of two biological repeats, each containing three technical repeats. Control b (black dashed line) = absence of bacteria, outlined in light blue dashed line = enlarged area.

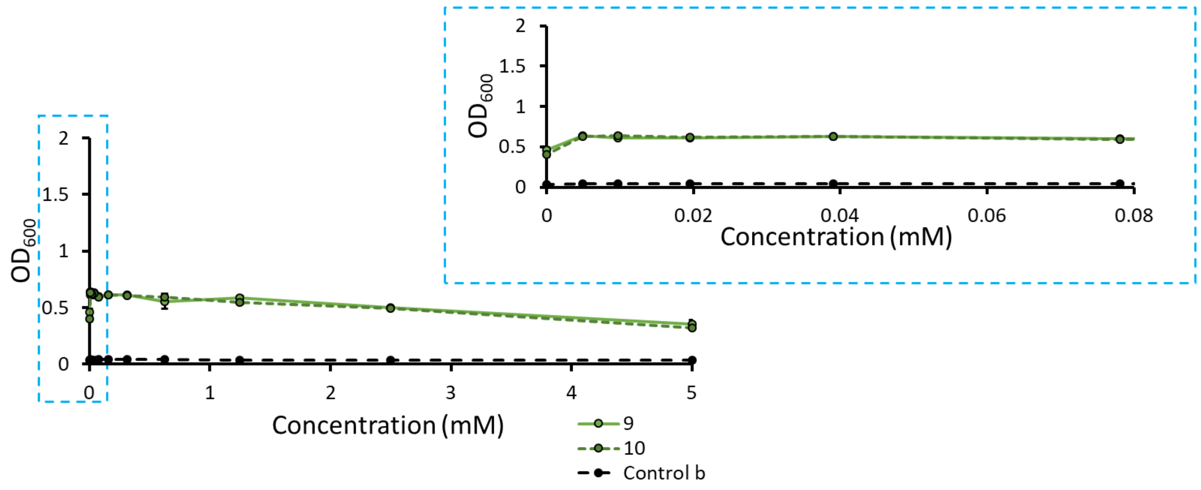

Figure S240 – Comparison of OD_{600} readings of **9** (solid green line) and **10** (dashed green line) at increasing concentrations in the presence of NCTC 775 (*E. faecalis*), created from an average of two biological repeats, each containing three technical repeats. Control b (black dashed line) = absence of bacteria, outlined in blue dashed line = enlarged area.

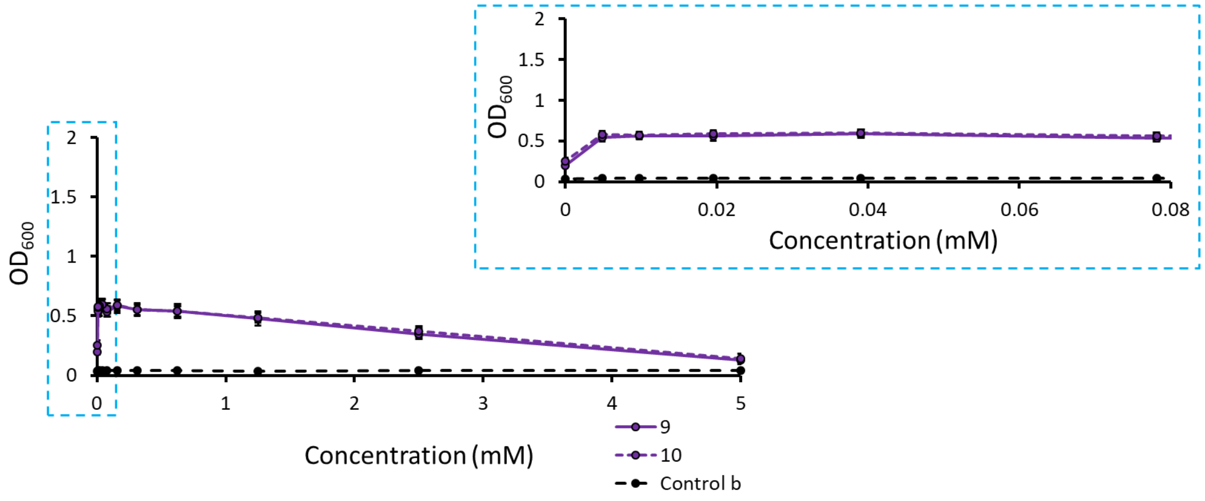

Figure S241 – Comparison of OD_{600} readings of **9** (solid purple line) and **10** (dashed purple line) at increasing concentrations in the presence of NCTC 12204 (*E. faecium*), created from an average of two biological repeats, each containing three technical repeats. Control b (black dashed line) = absence of bacteria, outlined in blue dashed line = enlarged area.

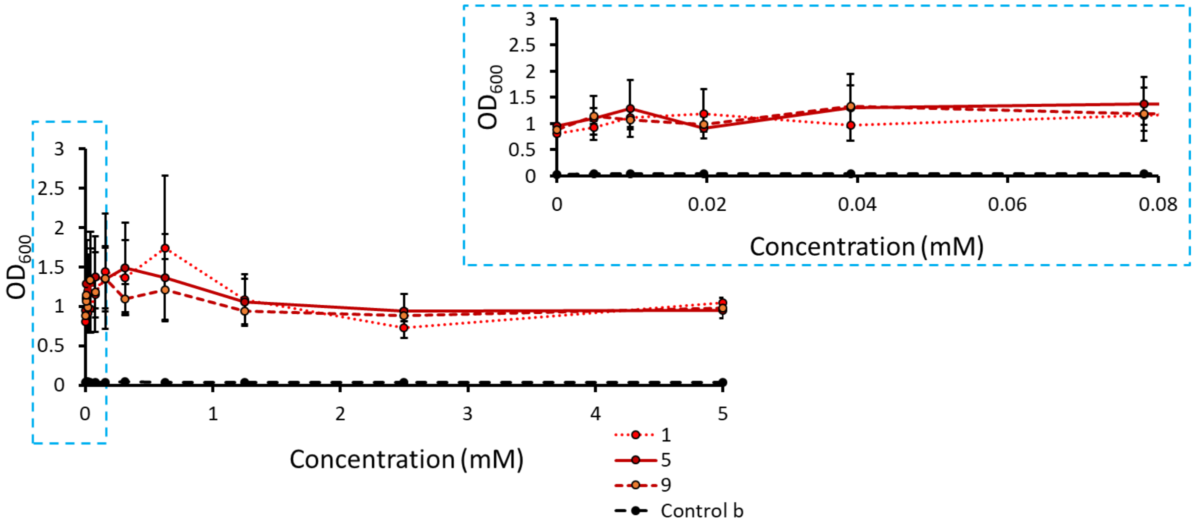

Figure S242 – Comparison of OD_{600} readings of **1** (dotted red line), **5** (solid red line) and **9** (dashed red line) at increasing concentrations in the presence of PAO1 (*P.aeruginosa*), created from an average of two biological repeats, each containing three technical repeats. Control b (black dashed line) = absence of bacteria, outlined in blue dashed line = enlarged area.

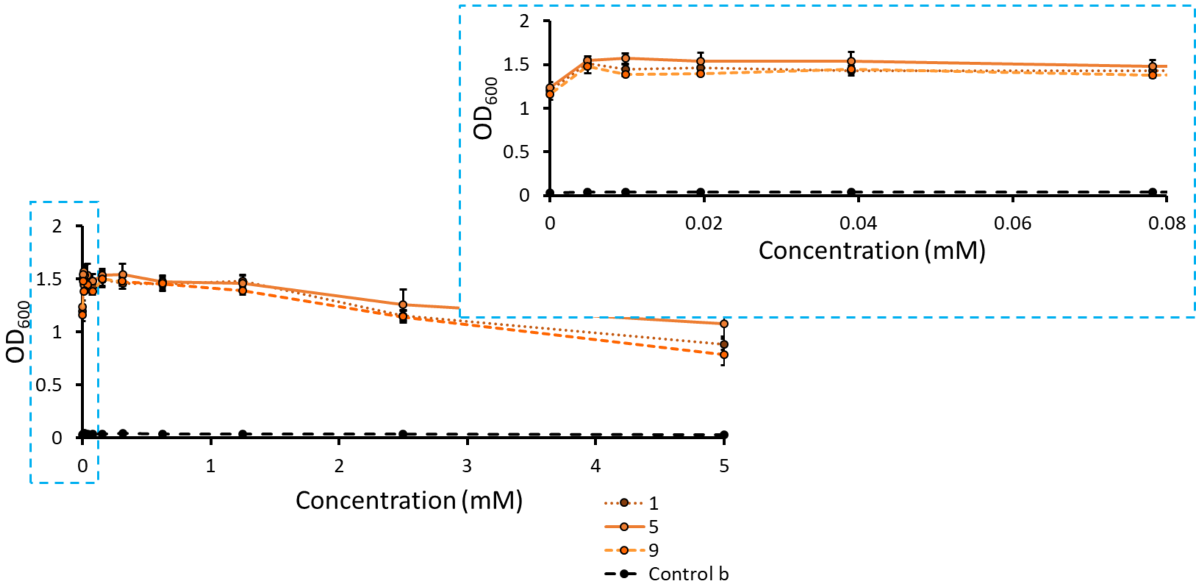

Figure S243 – Comparison of OD_{600} readings **1** (dotted orange line), **5** (solid orange line) and **9** (dashed orange line) at increasing concentrations in the presence of M6 (*K. pneumoniae*), created from an average of two biological repeats, each containing three technical repeats. Control b (black dashed line) = absence of bacteria, outlined in blue dashed line = enlarged area.

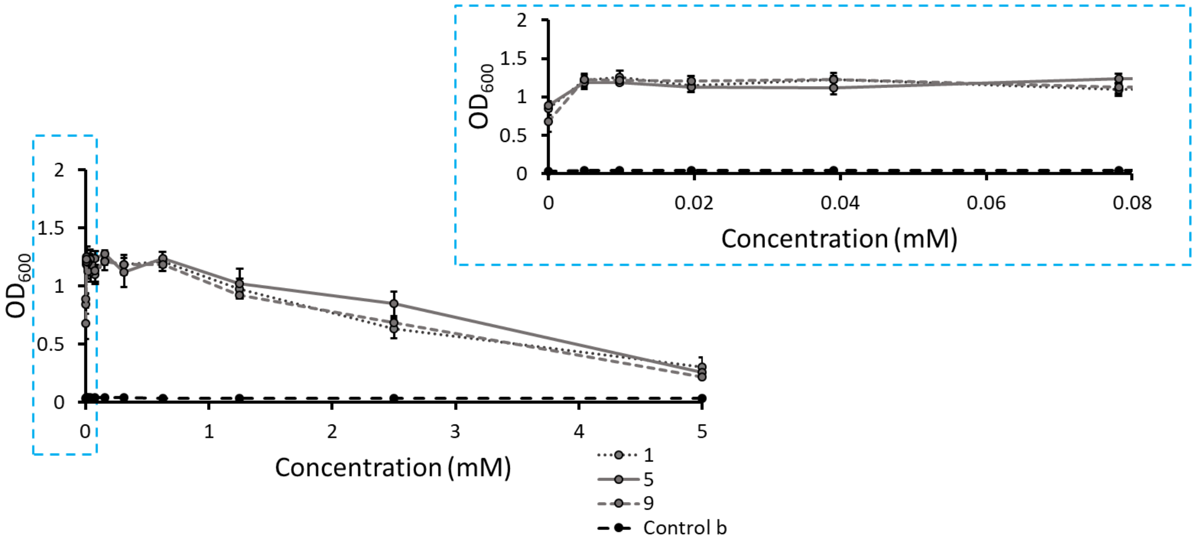

Figure S244 – Comparison of OD_{600} readings **1** (dotted grey line), **5** (solid grey line) and **9** (dashed grey line) at increasing concentrations in the presence of NCTC 12923 (*E. coli*), created from an average of two biological repeats, each containing three technical repeats. Control b (black dashed line) = absence of bacteria, outlined in blue dashed line = enlarged area.

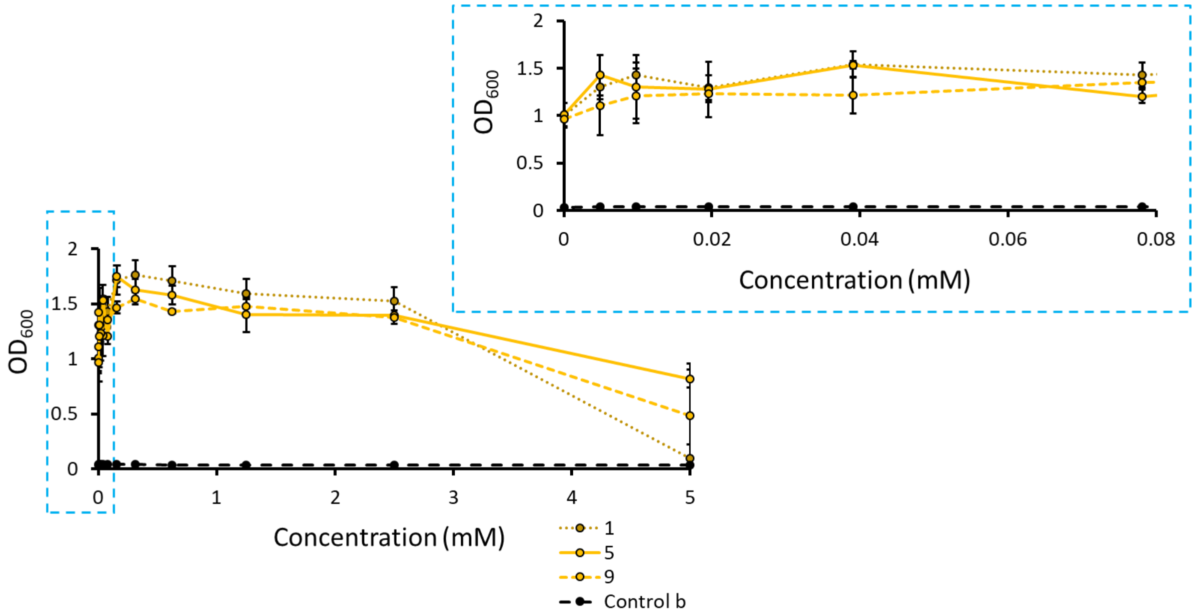

Figure S245 – Comparison of OD_{600} readings of **1** (dotted yellow line), **5** (solid yellow line) and **9** (dashed yellow line) at increasing concentrations in the presence of ATCC 17978 (*A. baumannii*), created from an average of two biological repeats, each containing three technical repeats. Control b (black dashed line) = absence of bacteria, outlined in blue dashed line = enlarged area.

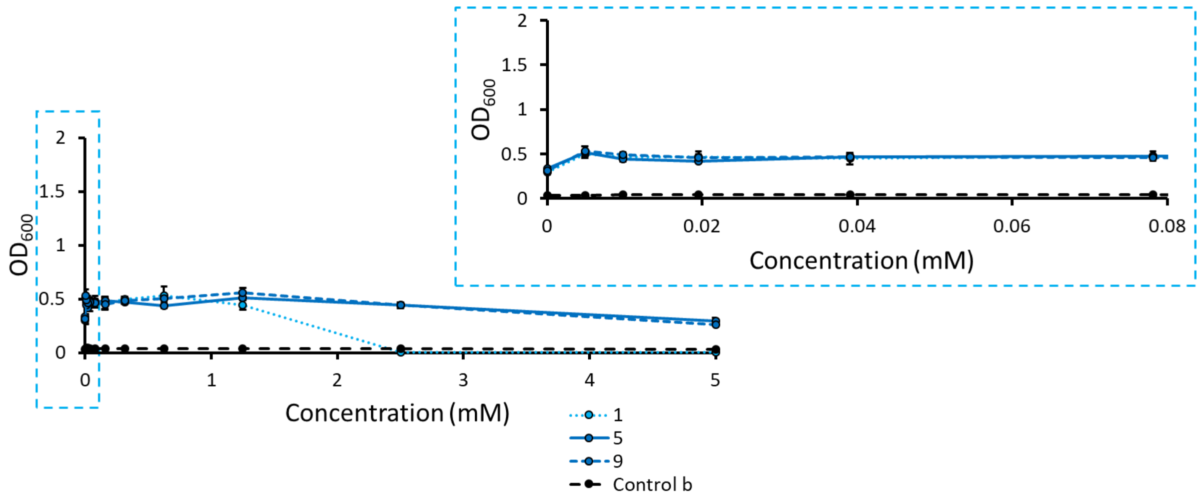

Figure S246 – Comparison of OD_{600} readings of **1** (dotted blue line), **5** (solid blue line) and **9** (dashed blue line) at increasing concentrations in the presence of ATCC 9144 (*S. aureus*), created from an average of two biological repeats, each containing three technical repeats. Control b (black dashed line) = absence of bacteria, outlined in light blue dashed line = enlarged area.

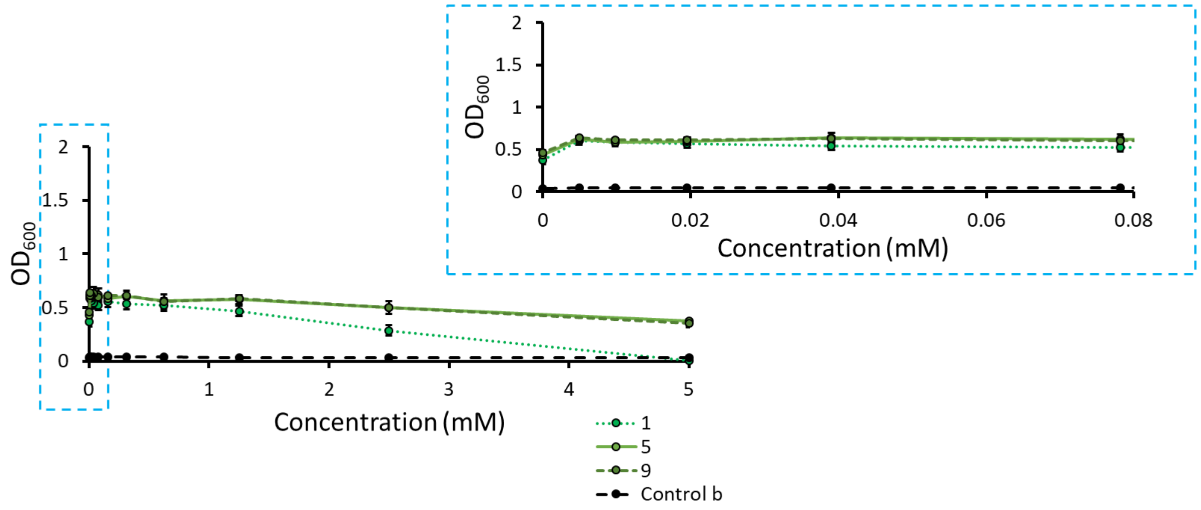

Figure S247 – Comparison of OD_{600} readings of **1** (dotted green line), **5** (solid green line) and **9** (dashed green line) at increasing concentrations in the presence of NCTC 775 (*E. faecalis*), created from an average of two biological repeats, each containing three technical repeats. Control b (black dashed line) = absence of bacteria, outlined in blue dashed line = enlarged area.

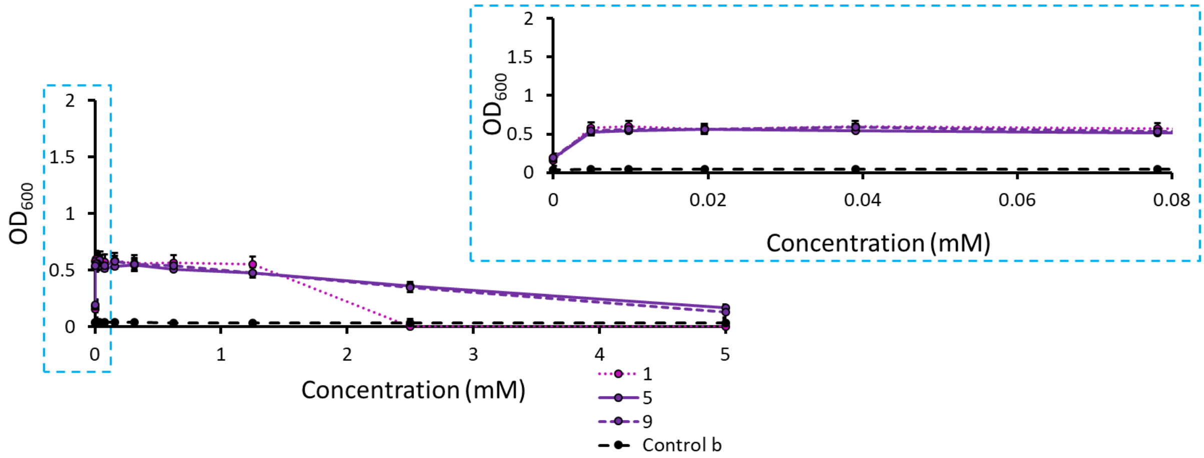

Figure S248 – Comparison of OD_{600} readings of **1** (dotted purple line), **5** (solid purple line) and **9** (dashed purple line) at increasing concentrations in the presence of NCTC 12204 (*E. faecium*), created from an average of two biological repeats, each containing three technical repeats. Control b (black dashed line) = absence of bacteria, outlined in blue dashed line = enlarged area.

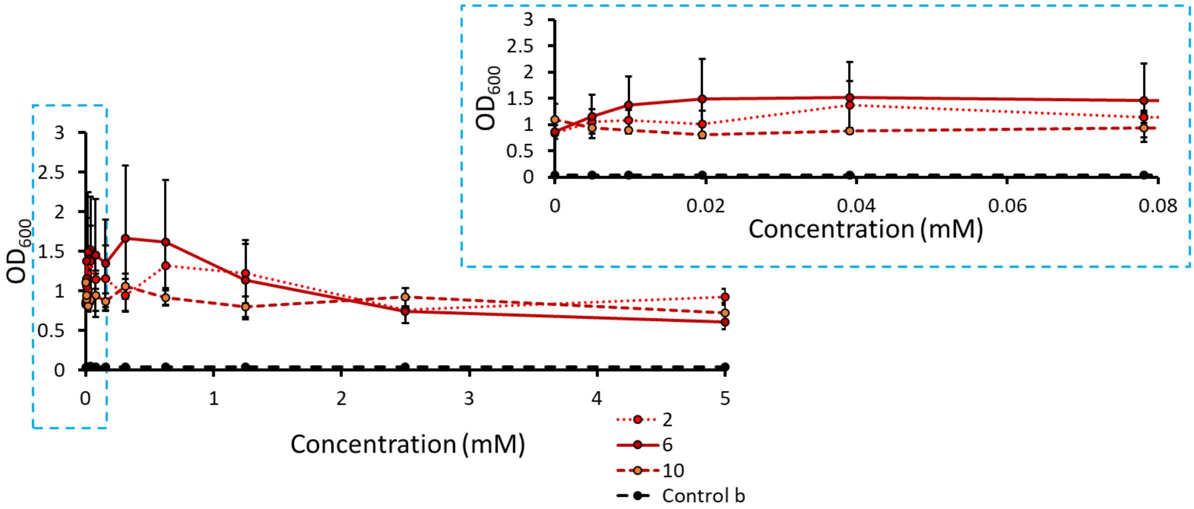

Figure S249 – Comparison of OD_{600} readings of **2** (dotted red line), **6** (solid red line) and **10** (dashed red line) at increasing concentrations in the presence of PAO1 (*P.aeruginosa*), created from an average of two biological repeats, each containing three technical repeats. Control b (black dashed line) = absence of bacteria, outlined in blue dashed line = enlarged area.

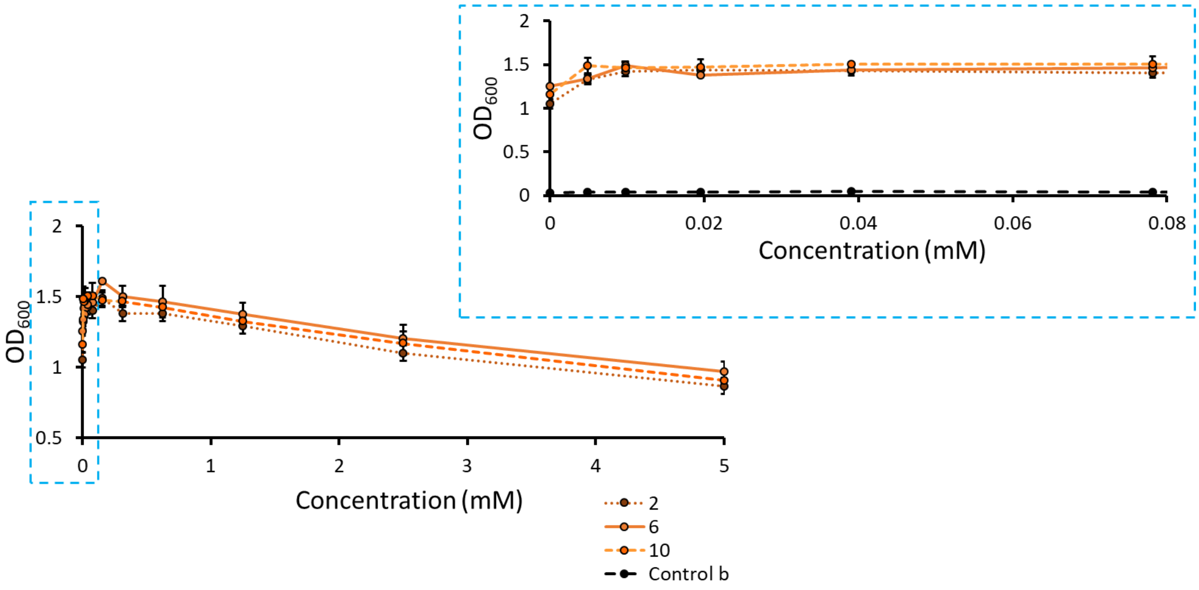

Figure S250 – Comparison of OD_{600} readings **2** (dotted orange line), **6** (solid orange line) and **10** (dashed orange line) at increasing concentrations in the presence of M6 (*K. pneumoniae*), created from an average of two biological repeats, each containing three technical repeats. Control b (black dashed line) = absence of bacteria, outlined in blue dashed line = enlarged area.

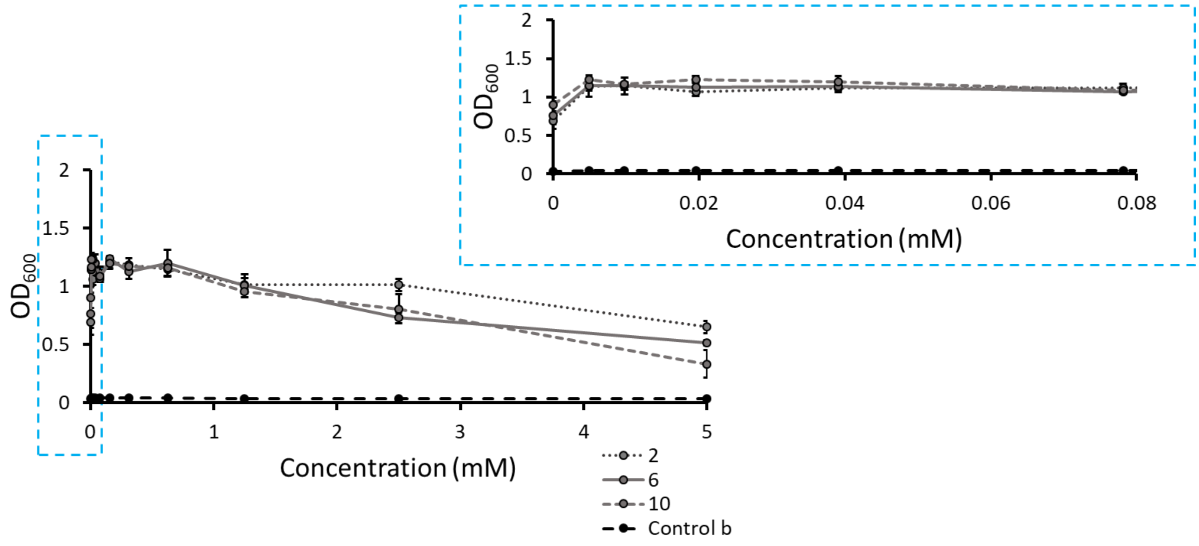

Figure S251 – Comparison of OD_{600} readings **2** (dotted grey line), **6** (solid grey line) and **10** (dashed grey line) at increasing concentrations in the presence of NCTC 12923 (*E. coli*), created from an average of two biological repeats, each containing three technical repeats. Control b (black dashed line) = absence of bacteria, outlined in blue dashed line = enlarged area.

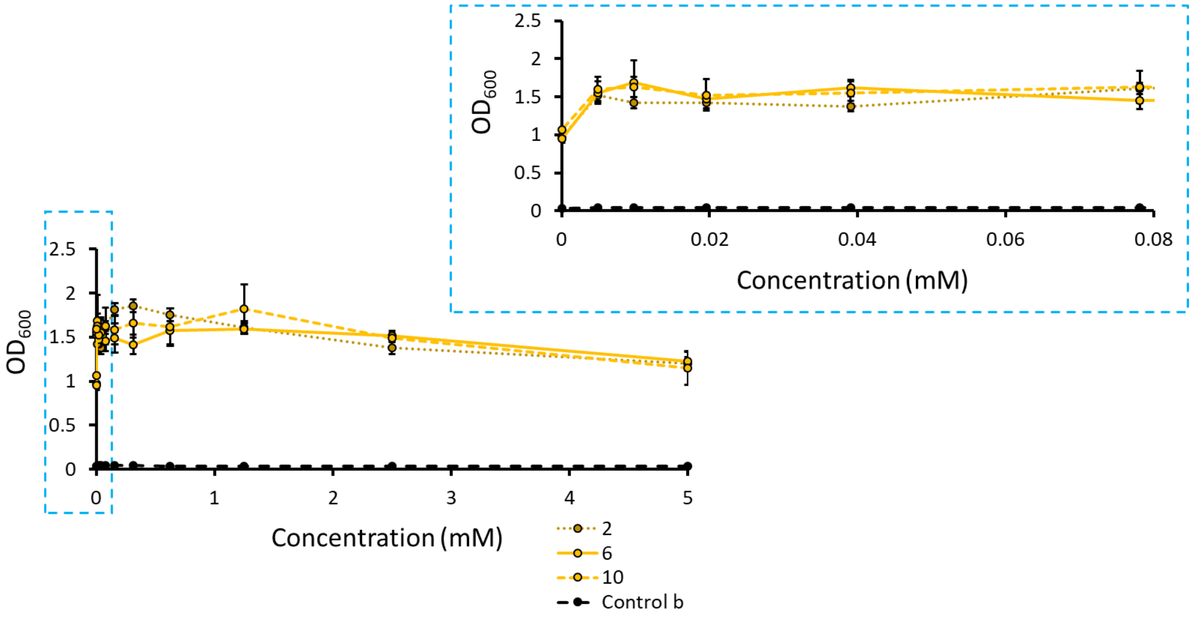

Figure S252 – Comparison of OD_{600} readings of **2** (dotted yellow line), **6** (solid yellow line) and **10** (dashed yellow line) at increasing concentrations in the presence of ATCC 17978 (*A. baumannii*), created from an average of two biological repeats, each containing three technical repeats. Control b (black dashed line) = absence of bacteria, outlined in blue dashed line = enlarged area.

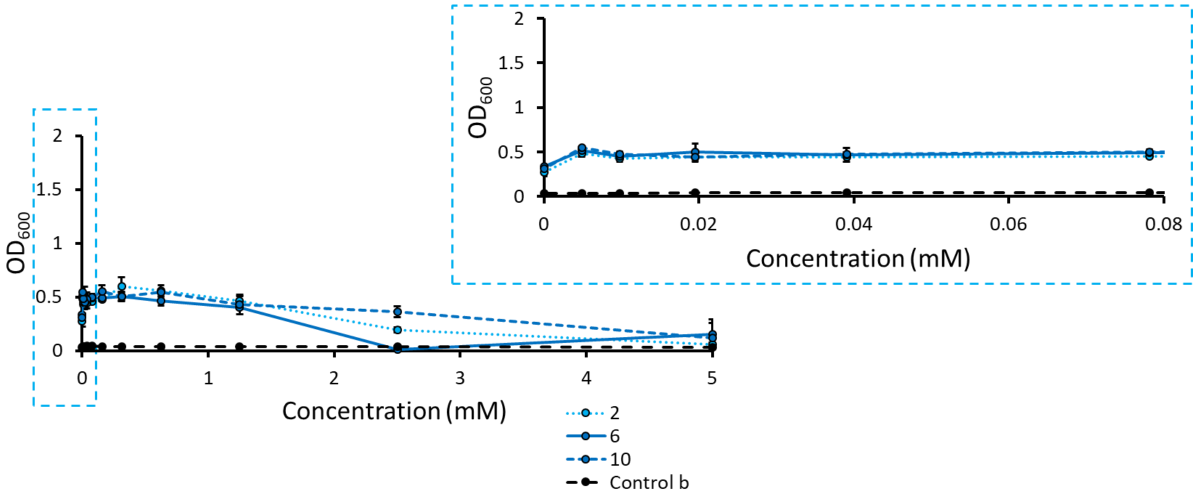

Figure S253 – Comparison of OD_{600} readings of **2** (dotted blue line), **6** (solid blue line) and **10** (dashed blue line) at increasing concentrations in the presence of ATCC 9144 (*S. aureus*), created from an average of two biological repeats, each containing three technical repeats. Control b (black dashed line) = absence of bacteria, outlined in light blue dashed line = enlarged area.

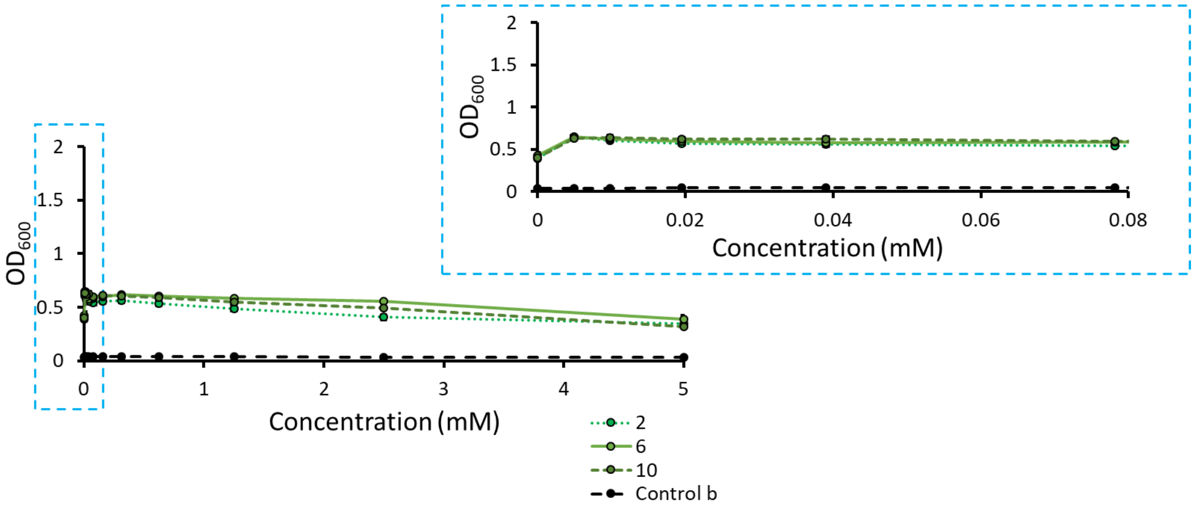

Figure S254 – Comparison of OD_{600} readings of **2** (dotted green line), **6** (solid green line) and **10** (dashed green line) at increasing concentrations in the presence of NCTC 775 (*E. faecalis*), created from an average of two biological repeats, each containing three technical repeats. Control b (black dashed line) = absence of bacteria, outlined in blue dashed line = enlarged area.

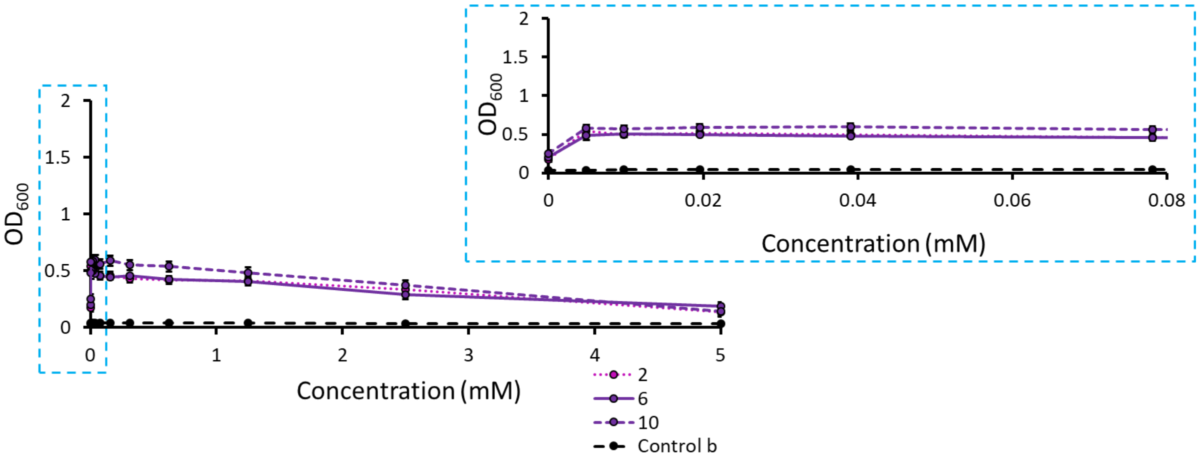

Figure S255 – Comparison of OD_{600} readings of **2** (dotted purple line), **6** (solid purple line) and **10** (dashed purple line) at increasing concentrations in the presence of NCTC 12204 (*E. faecium*), created from an average of two biological repeats, each containing three technical repeats. Control b (black dashed line) = absence of bacteria, outlined in blue dashed line = enlarged area.

Summary

Table S21 – Percentage inhibition of bacterial growth after 20 hours incubation with the appropriate SSA, supplied as a 10.0 mM in an H_2O/ 5.0 % EtOH solution to give a final top concentration of 5.0 mM

Red = ≤ 0 %, orange = > 0 % - ≤ 24 %, blue = > 24 % – ≤ 49 %, purple = > 49 % – ≤ 74 % and green = > 74 % – 100 %

	Gram-negative				Gram-positive		
	Bacterial strain						
SSA	PAO1 (*P.aeruginosa*)	M6 (*K. pneumoniae*)	NCTC 12923 (*E. coli*)	ATCC 17978 (*A. baumannii*)	ATCC 9144 (*S. aureus*)	NCTC 775 (*E. faecalis*)	NCTC 12204 (*E. faecium*)
**1**	-28.56	26.19	64.03	90.67	98.67	98.79	99.02
**2**	-11.25	17.56	6.06	-23.34	79.71	21.86	66.25
**3**	-35.71	15.37	59.91	94.45	99.09	98.85	99.41
**4**	10.30	19.93	39.02	-20.93	98.85	98.82	99.74
**5**	-0.05	12.79	70.96	18.61	12.67	12.98	5.80
**6**	29.42	22.92	32.77	-28.50	55.64	7.71	7.44
**7**	-24.25	32.42	64.22	24.84	5.65	47.23	50.11
**8**	37.24	9.69	39.33	-20.26	96.79	38.33	-1.33
**9**	34.57	21.69	62.96	-8.08	60.74	20.00	44.44
**10**	-10.14	31.98	67.91	49.97	17.46	23.13	34.10

Section 17: References

1 G. M. Sheldrick, *Acta Crystallogr A Found Adv*, 2015, **71**, 3–8.

2 G. M. Sheldrick, *Acta Crystallogr C Struct Chem*, 2015, **71**, 3–8.

3 G. M. Sheldrick, *Acta Crystallogr A*, 2015, **71**, 3–8.

4 O. V Dolomanov, L. J. Bourhis, R. J. Gildea, J. A. K. Howard and H. Puschmann, *J Appl Crystallogr*, 2009, **42**, 339–341.
